# Stereoselective radical C–H alkylation with acceptor/acceptor-substituted diazo reagents *via* Co(ii)-based metalloradical catalysis†
†Electronic supplementary information (ESI) available. CCDC 1020629–1020631. For ESI and crystallographic data in CIF or other electronic format see DOI: 10.1039/c4sc02610a



**DOI:** 10.1039/c4sc02610a

**Published:** 2014-11-12

**Authors:** Xin Cui, Xue Xu, Li-Mei Jin, Lukasz Wojtas, X. Peter Zhang

**Affiliations:** a Department of Chemistry , University of South Florida Tampa , FL 33620-5250 , USA . Email: xpzhang@usf.edu ; Fax: +1 813-974-1733 ; Tel: +1 813-974-7249

## Abstract

Co(ii)-based metalloradical catalysis has, for the first time, been successfully applied for asymmetric intramolecular C–H alkylation of acceptor/acceptor-substituted diazo reagents. Through the design and synthesis of a new *D*_2_-symmetric chiral amidoporphyrin as the supporting ligand, the Co(ii)-based metalloradical system, which operates at room temperature, is capable of 1,5-C–H alkylation of α-methoxycarbonyl-α-diazosulfones with a broad range of electronic properties, providing the 5-membered sulfolane derivatives in high yields with excellent diastereoselectivities and enantioselectivities. In addition to complete chemoselectivity toward allylic and allenic C–H bonds, the Co(ii)-based metalloradical catalysis for asymmetric C–H alkylation features a remarkable degree of functional group tolerance.

## Abstract 2

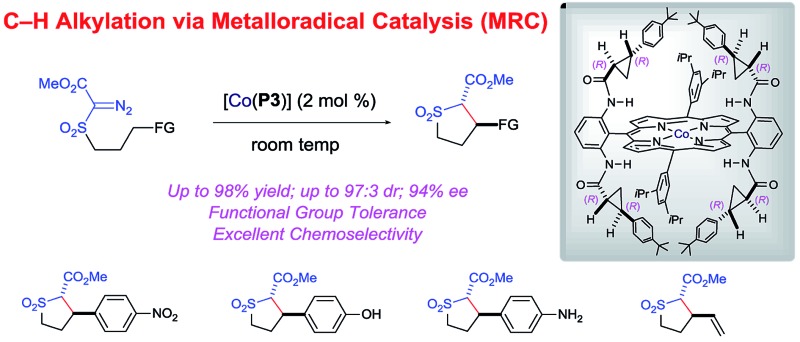
Co(ii)-based metalloradical catalysis has, for the first time, been successfully applied for asymmetric intramolecular C–H alkylation of acceptor/acceptor-substituted diazo reagents.

## Introduction

**Scheme 1:**
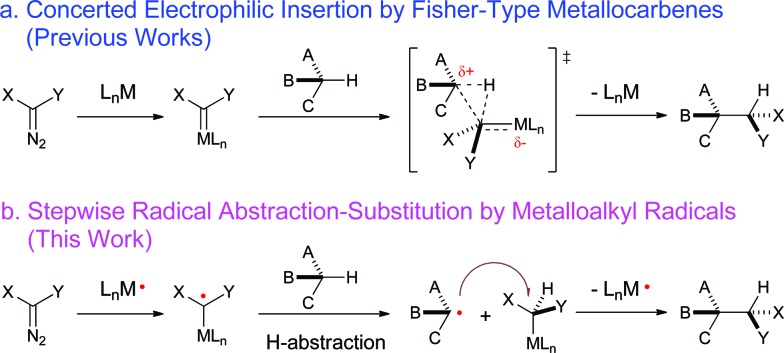
C–H functionalization by a) electrophilic metallocarbene insertion and b) radical C–H alkylation *via* MRC.

Direct C–H bond functionalization lies at the heart of modern organic chemistry and has attracted growing attention from synthetic chemists.^1^ The development of catalytic asymmetric systems for C–H functionalization will allow for the construction of optically active compounds directly from ubiquitous C–H bonds while installing various functionalities. Such a type of catalytic transformation is inherently challenging as it requires the catalyst to be sufficiently reactive to activate normally inert C–H bonds while demanding high controllability in order to achieve chemo-, regio- and stereoselectivity. Among different approaches, asymmetric C–H alkylation *via* metal-catalyzed carbene insertion represents one of the most effective methods for the enantioselective functionalization of C–H bonds (Scheme 1a).^2^ A number of metal catalysts, including Rh_2_,^2b,2f–k,3^ Cu,^2b,2f–k^ Ir,^4^ and Fe^5^ complexes, have been successfully developed to catalyze enantioselective C–H alkylation with diazo reagents as the carbene sources. In fact, asymmetric C–H alkylation *via* catalytic carbene insertion has already been applied as a key strategy for the enantioselective syntheses of natural products and pharmaceutically important molecules.^2b,2f,2g,2i,6^ While the existing metal catalysts were shown to be highly effective with the use of acceptor- and donor/acceptor-substituted diazo reagents, acceptor/acceptor (A/A)-substituted diazo reagents, which bear two electron-withdrawing groups at the α-carbon, have proven to be highly challenging with respect to serving as carbene precursors for asymmetric C–H insertions.^2f–h,7^ This challenge is closely related to the electronic nature of the existing Lewis acidic metal catalysts as well as the Fischer-type metallocarbene intermediates of these catalytic systems. Since the presence of the two electron-withdrawing groups results in significant decreases in the electron density at the α-carbon centers, A/A-substituted diazo reagents are generally less reactive toward Lewis acidic metal catalysts for carbene insertion processes. Once formed, the A/A-substituted metallocarbenes would be intrinsically too electrophilic to be controlled in subsequent C–H insertion steps, leading to poor regio- and enantioselectivities. Moreover, the high electrophilicity of the metallocarbenes would render a catalytic insertion system based on the use of A/A-substituted diazo reagents limited, with a substrate scope of only electron-rich C–H substrates and without the capability of functionalizing electron-deficient C–H bonds.

Among previous efforts toward enantioselective C–H alkylation with A/A-substituted diazo reagents,^7^ the most notable example is the Cu-based intramolecular system recently reported by Maguire and coworkers.^8^ Supported by chiral bisoxazoline ligands, this Cu-catalyzed asymmetric system was shown to enable intramolecular C–H insertion with α-alkoxycarbonyl-α-diazosulfones, affording the corresponding six-membered thiopyrans in high enantioselectivities.^8^ However, the yields of the desired products were generally low to moderate (30–68%) as the Cu-catalyzed reactions typically gave a complex mixture of products. Furthermore, it was reported that the efficiency of the catalytic system was further reduced for C–H substrates with decreased electron richness. For example, the insertion reaction was completely inhibited for benzylic C–H bonds with an electron-withdrawing NO_2_ group substituted at the *para*-position of the phenyl ring.^8*a*^ Evidently, general and effective catalytic systems for asymmetric C–H alkylation *via* metal-mediated carbene insertion with A/A-substituted diazo reagents remain to be developed, despite extensive efforts undertaken.^2*f*^ Besides seeking further improvements on existing catalytic systems, exploration of fundamentally different pathways involving intermediates other than Fischer-type electrophilic metallocarbenes may provide new opportunities for addressing this and related challenges in asymmetric C–H alkylation.

As stable low-spin 15e-metalloradicals, cobalt(ii) porphyrin complexes, [Co(Por)], have been disclosed to activate diazo reagents to form α-Co(iii)-alkyl radicals (also known as Co(iii)-carbene radicals), which serve as key intermediates in Co(ii)-based metalloradical catalysis (MRC).^9^ Unlike the electrophilic Fischer-type carbene intermediates, α-Co(iii)-alkyl radicals have been demonstrated to undergo radical addition to alkenes and alkynes, followed by radical cyclization, leading to the development of catalytic radical cyclopropanation,^3e,10^ cyclopropenation^11^ and furanylation reactions.^12^ Considering the genuine radical nature of the metalloalkyl radical intermediates, we envisioned the possibility of a new C–H alkylation process (Scheme 1b) if (i) the α-Co(iii)-alkyl radical is capable of abstracting a C–H bond hydrogen atom and (ii) the subsequent radical substitution reaction between the resulting alkyl radical and Co(iii)-alkyl complex could proceed effectively. This type of metalloradical alkylation would be both fundamentally interesting and practically attractive as the radical pathway would be much less dependent on the electronic properties of the diazo reagents and C–H substrates, potentially leading to the development of a general catalytic system for C–H alkylation, including with A/A-substituted diazo reagents and for electron-deficient C–H bonds. Moreover, as another notable feature of radical reactions, this type of C–H functionalization would be expected to have a high degree of functional group tolerance.^13^

As the outcome of our efforts toward the development of radical-type C–H alkylation, we report herein the first Co(ii)-based metalloradical system that is highly effective for asymmetric intramolecular C–H alkylation with α-methoxycarbonyl-α-diazosulfones, a class of A/A-substituted diazo reagents. The new Co(ii)-catalyzed system can proceed at room temperature and is capable of alkylating C–H bonds with wide-ranging electronic properties, including challenging electron-deficient C–H bonds. In addition to high diastereo- and enantioselectivity, the metalloradical process features a remarkable degree of tolerance toward various functionalities, including unprotected OH and NH_2_ groups, as well as excellent chemoselectivity for allylic/allenic C–H alkylation.

## Results and discussion

Initial experiments were performed to examine the possibility of Co(ii)-based metalloradical catalysis for 1,5-C–H alkylation with α-methoxycarbonyl-α-diazosulfones **1**, a class of A/A-substituted diazo reagents that has not been previously demonstrated to undergo highly asymmetric C–H alkylation.^8*a*^ Reaction screening started with a challenging C–H substrate with a 4-nitrophenyl group, **1a** (Table 1), which was shown to be ineffective for the Cu-based C–H insertion, presumably due to its electron-deficiency.^8*a*^ The common [Co(TPP)] (TPP = 5,10,15,20-tetraphenylporphyrin) was shown to be incapable of activating **1a** for the expected C–H alkylation reaction, even when it was used in a stoichiometric amount (entry 1). We then turned our attention to the use of Co(ii) complexes of *D*_2_-symmetric chiral amidoporphyrins [Co(*D*_2_-Por*)] as potential catalysts.^14^ Remarkably, when [Co(**P1**)] (**P1** = 3,5-Di^*t*^Bu-ChenPhyrin), a known metalloradical catalyst for radical cyclopropanation,^10,14^ was employed at only 2 mol% catalyst loading, effective intramolecular alkylation of the benzylic C–H bonds was observed even at room temperature, affording the desired *trans*-sulfolane **2a** in an 83% yield with a 90% dr, although with low enantioselectivity (entry 2). This dramatic ligand-accelerated catalysis is rationalized to be as a result of double N–H···O hydrogen bonding interactions between two of the amide N–H units on the ligand as donors and the S=O (SO_2_ group) and C=O (CO_2_Me group) units of the substrate moiety as acceptors,^10a,10b,10d^ which may facilitate the activation of **1a** through stabilization of the resulting α-Co(iii)-alkyl radical **A** (Table 1). To improve enantioselectivity, a new *D*_2_-symmetric chiral amidoporphyrin, 3,5-Di^*t*^Bu-(4′-^*t*^Bu)XuPhyrin (**P2**), was modularly constructed from the chiral cyclopropanecarboxyamide containing two stereogenic centers (see ESI†). Under the same conditions, the Co(ii) complex of this second-generation catalyst [Co(**P2**)] (Table 1) was shown to catalyze the C–H alkylation reaction with significantly improved enantioselectivity and similarly high diastereoselectivity, but with a reduced product yield (entry 3). In an effort to increase the reaction yield without affecting its high stereoselectivities, replacement of 3,5-di-*tert*-butyl groups with 3,5-diisopropyl groups in two of the *meso*-positions of **P2**, without changing the chiral building blocks, led to the design and synthesis of the less-hindered chiral porphyrin 3,5-DiiPr-(4′-^*t*^Bu)XuPhyrin (**P3**) (see ESI†). The Co(ii) complex of **P3**, [Co(**P3**)], was shown to efficiently catalyze the room temperature C–H alkylation of **1a**, producing *trans*-sulfolane **2a** in 92% yield with 92% de and 92% ee (entry 4).

**Table 1:** Effect of the porphyrin ligand on the stereoselective metalloradical C–H alkylation of α-methoxycarbonyl-α-diazosulfone **1a** catalyzed by [Co(*D*_2_-Por*)]^*a*^

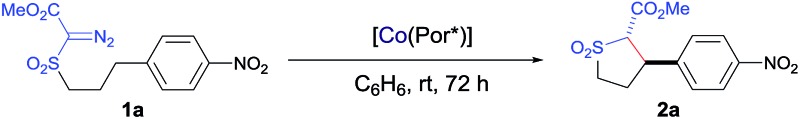				
Entry	Catalyst (loading)	Yield^*b*^ (%)	dr^*c*^	ee^*d*^ (%)
1	[Co(TPP)] (120 mol%)	NR^*e*^	—	—
2	[Co(**P1**)] (2 mol%)	83	95 : 5	–24
3	[Co(**P2**)] (2 mol%)	63	96 : 4	91
4	[Co(**P3**)] (2 mol%)	92	96 : 4	92
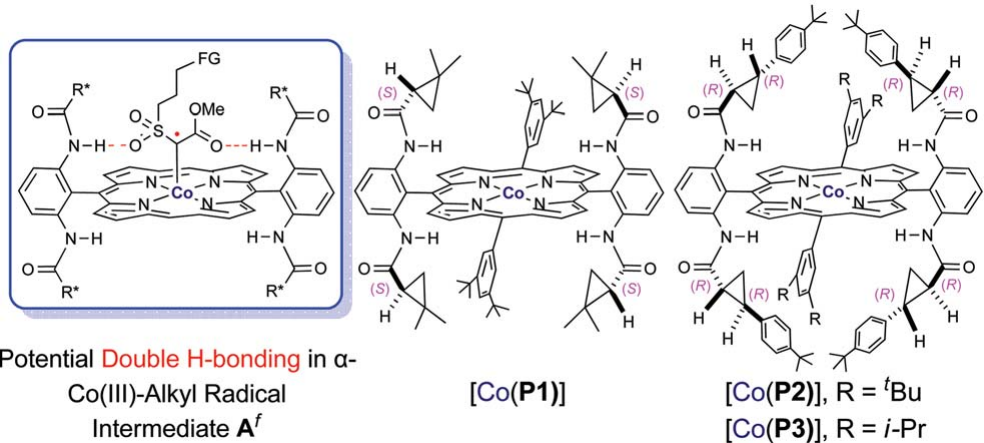				

^*a*^Reactions were carried out at room temperature for 72 h in a one-time fashion without slow addition of the diazo reagent using [Co(Por)] under N_2_.

^*b*^Isolated yields.

^*c*^The trans : cis diastereomeric ratio determined by ^1^H-NMR.

^*d*^Enantiomeric excess determined by chiral HPLC.

^*e*^No reaction.

^*f*^For clarity, the other two *meso*-groups of the porphyrin are omitted.

The [Co(**P3**)]-catalyzed intramolecular C–H alkylation was demonstrated to be applicable to A/A-substituted diazo reagents, α-methoxycarbonyl-α-diazosulfones **1**, containing different types of C–H bonds with varied electronic properties and substituents, leading to the stereoselective formation of *trans*-sulfolane derivatives **2** (Table 2). In addition to **1a** bearing the electron-withdrawing NO_2_ group, diazo reagents **1b–f** with various halogen substituents such as CF_3_, F, Cl, and Br could also be transformed by [Co(**P3**)] to the corresponding sulfolanes **2b–f** in high yields with high stereoselectivities (entries 1–6). As expected, α-diazosulfones with electron-neutral aryl units such as non-substituted phenyl (**1g**) and *para*-methylphenyl groups (**1h**) were also suitable substrates for the Co(ii)-based system, providing the desired C–H alkylation products **2g** and **2h** in similarly high yields and stereoselectivities (entries 7 and 8). The relative and absolute configurations of the two contiguous chiral centers in **2g** were established as [2*S*,3*R*] by X-ray crystal structural analysis (see ESI†). Likewise, electron-rich benzylic C–H bonds could also be effectively alkylated by the Co(ii)-based catalytic system, as demonstrated with the high-yielding and highly selective reactions of diazo reagents **1i** and **1j** containing electron-donating 4-alkoxyphenyl groups (entries 9 and 10). These results indicate that the Co(ii)-catalyzed asymmetric alkylation is insensitive to the electronics of the C–H substrates, which is in line with the envisioned radical mechanism (Scheme 1b).

**Table 2:** [Co(**P3**)]-catalyzed asymmetric C–H alkylation of α-methoxycarbonyl-α-diazosulfone compounds^*a*^

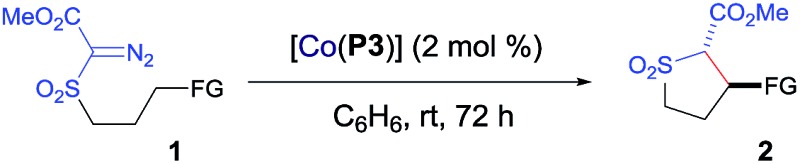
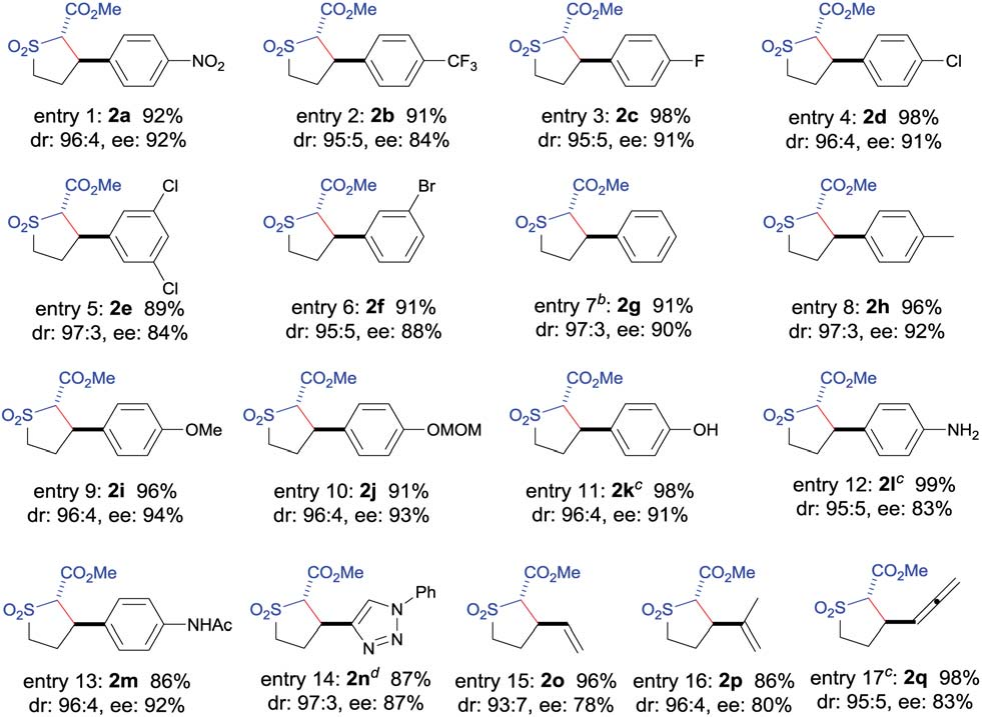

^*a*^Syntheses of catalysts and diazo compounds are summarized in ESI;^15^ reactions were carried out at room temperature for 72 h using [Co(**P3**)] under N_2_; isolated yields; the trans : cis diastereomeric ratios were determined by ^1^H-NMR; enantiomeric excesses were determined by chiral HPLC.

^*b*^[2*S*,3*R*] absolute configuration determined by anomalous-dispersion effects in X-ray diffraction measurements on a crystal.

^*c*^5 mol% catalyst used.

^*d*^PhF used as solvent.

The [Co(**P3**)]-based catalytic system was further shown to display other attractive features that are unique for radical processes. Firstly, the metalloradical C–H alkylation was found to tolerate well various functional groups. For example, C–H substrates containing unprotected hydroxyl (**1k**) and amino (**1l**) groups as well as amido (**1m**) and triazole (**1n**) functionalities could undergo catalytic intramolecular alkylation reactions without affecting these usually reactive functional groups, providing highly functionalized *trans*-sulfolanes **2k–n** in excellent yields with high stereoselectivities (entries 11–14). Secondly, excellent chemoselectivity for intramolecular allylic C–H alkylation to form 5-membered sulfolanes *versus* C=C cyclopropanation to form bicyclo[4.1.0] structures was observed for this Co(ii)-based metalloradical catalysis. Allylic C–H substrates such as **1o** and **1p** were chemoselectively alkylated to form sulfolanes **2o** and **2p** exclusively (entries 15 and 16), without any complications from the competitive cyclopropanations of the neighboring C=C bonds.^16^ Besides allylic C–H bonds, chemoselective alkylation of allenic C–H bonds could also be achieved by [Co(**P3**)], as exemplified with substrate **1q**, affording the corresponding sulfolane **2q** in an excellent yield without any side reactions (entry 17). The remarkable chemoselectivity as well as functional group tolerance, together with the observed electronic insensitivity, highlight the unique features of this Co(ii)-based metalloradical alkylation system.^17^

The demonstrated reactivity and selectivity profile of the Co(ii)-catalyzed C–H alkylation is in good agreement with the anticipated radical pathway of metalloradical catalysis (MRC) (Scheme 1b). To directly probe the radical mechanism, we investigated potential *E*–*Z* olefin isomerization of Co(ii)-catalyzed allylic C–H alkylation. Different from the concerted insertion pathway (Scheme 1a), the radical allylic alkylation would involve formation of allylic radical intermediates as a result of H-atom abstraction of allylic C–H bonds by the initial α-Co(iii)-alkyl radicals. In view of the facile *E*–*Z* interconversion of allylic radicals,^10b,18^ the catalytic reaction of isomerically pure allylic C–H substrates could lead to the formation of a mixture of (*E*)- and (*Z*)-alkylation products. To this end, α-methoxycarbonyl-α-diazosulfones **1r** and **1s**, which were derived from the (*E*)- and (*Z*)-isomers of 2-hexene, respectively, were employed as radical probe substrates for Co(ii)-based metalloradical alkylation. As expected, *E*–*Z* isomerization was observed in the alkylation reactions of both **1r** and **1s**, producing isomeric mixtures of products **2r** and **2s** in high combined yields (Table 3). Interestingly, the degree of the isomerization could be controlled by Co(ii) catalysts with different ligand environments. With use of the sterically encumbered [Co(**P3**)] catalyst, both **1r** and **1s** tended to mostly retain their olefin configuration with only slight isomerization observed (entries 1 and 4). When the less sterically hindered [Co(**P4**)] (**P4** = 3,5-Di^*t*^Bu-IbuPhyrin) was used as the catalyst, an increase in the degree of isomerization was observed for both alkylation reactions (entries 2 and 5). These results indicate that the degree of isomerization of the allylic radicals is kinetically controlled by the ligand sterics. Accordingly, by using even less sterically hindered [Co(**P5**)] (**P5** = *meso-^n^* Bu-IbuPhyrin) as the catalyst, further increases in isomerization were observed in both reactions (entries 3 and 6). In fact, [Co(**P5**)]-catalyzed alkylation reactions of both **1r** and **1s** generated mixtures of **2r** and **2s** with similar ratios (entries 3 and 6), suggesting near equilibrium distributions of the two isomeric products. The results from these isomerization experiments provide further support of the proposed radical mechanism for the Co(ii)-catalyzed alkylation.

**Table 3:** Catalyst-controlled olefin isomerizations to probe the radical mechanism of Co(ii)-catalyzed C–H alkylation^*a*^

Entry	Diazo	[Co(**P**)]	Yield^*b*^ (%)	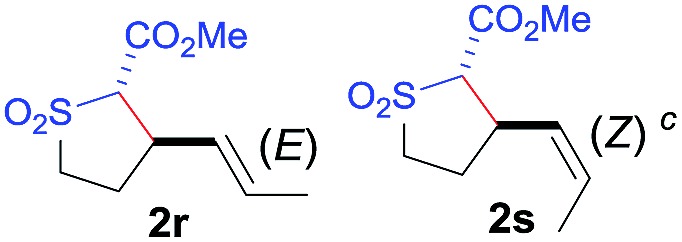
1	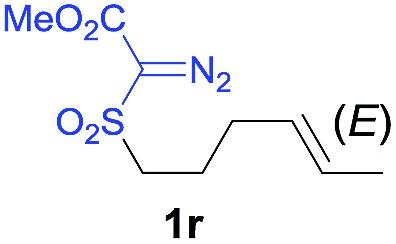	[Co(**P3**)]	94	95 : 5
2		[Co(**P4**)]	96	89 : 11
3		[Co(**P5**)]	96	82 : 18
4	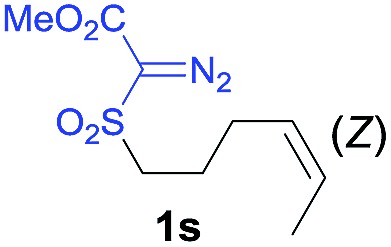	[Co(**P3**)]	92	18 : 82
5		[Co(**P4**)]	94	49 : 51
6		[Co(**P5**)]	95	77 : 23
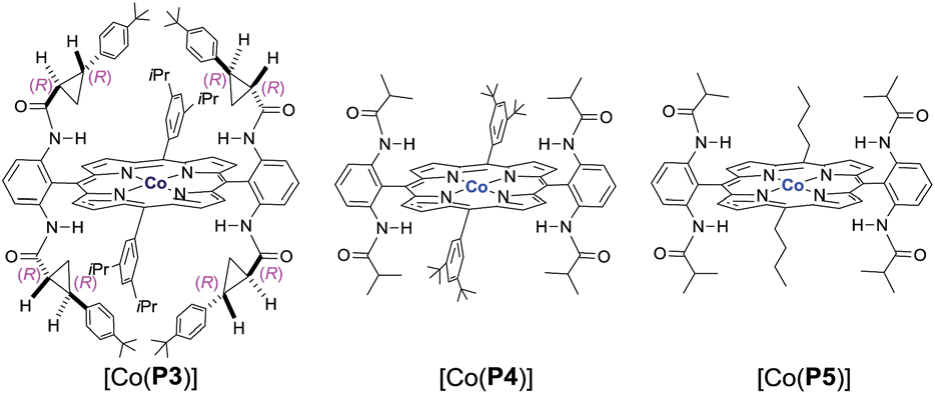				

^*a*^Reactions were carried out in benzene with 2 mol % catalyst at 40 °C for 72 h under N_2_.

^*b*^Isolated yields.

^*c*^The *E*–*Z* ratio determined by ^1^H-NMR.

The Co(ii)-catalyzed asymmetric C–H alkylation allowed for stereoselective construction of 5-membered sulfolane structures with concurrent creation of two contiguous stereogenic centers. By taking advantage of the acidity of the chiral methine unit between the two electron-withdrawing groups, sulfolanes **2** could be further transformed to produce more densely functionalized derivatives **3** (Table 4), which may find interesting biomedical applications.^19^ For example, enantioenriched sulfolanes **2i** and **2e** could be selectively fluorinated with selectfluor after facile deprotonation of the acidic chiral center, affording compounds **3ia** and **3ea**, respectively, in high yields with excellent diastereoselectivities and without affecting the original enantiopurities (entries 1 and 2). The absolute configuration of the two contiguous stereocenters in **3ea**, including the newly-created quaternary chiral center, was established as [2*R*,3*R*] by X-ray crystal structural analysis (see ESI†). Highly stereoselective chlorination and methylation could be similarly achieved as demonstrated with the high-yielding production of compounds **3ib** (entry 3) and **3ic** (entry 4), respectively, from **2i**. Besides nucleophilic substitution reactions, the resulting carbanion from the acidic chiral center in **2** could also be employed for Michael addition as exemplified by the reaction of **2i** with ethyl acrylate, affording multi-functional sulfolane **3id** while retaining the original optical purity (entry 5).

**Table 4:** Diastereoselective transformations of sulfolanes with construction of quaternary carbon stereocenters^*a*^

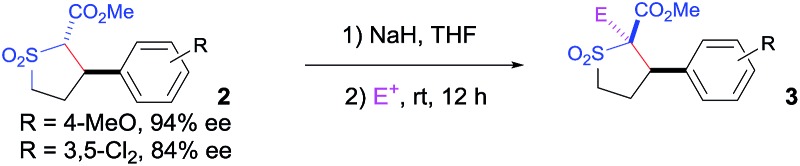					
Entry	Electrophile	Product	Yield (%)	dr	ee (%)
1^*b*^	Selectfluor	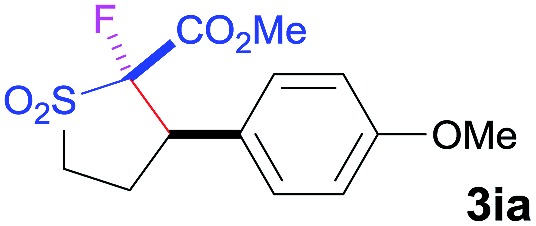	92	96 : 4	94
2^*b*^	Selectfluor	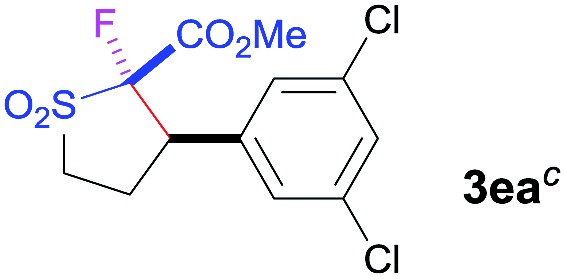	89	96 : 4	84
3	NCS	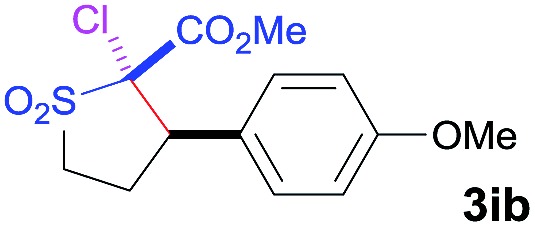	92	97 : 3	93
4	MeI	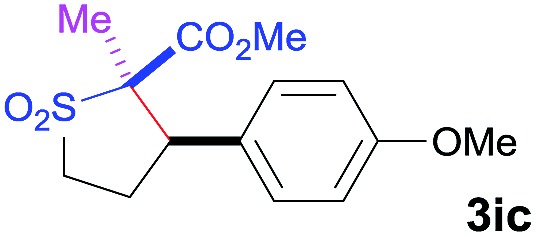	91	8 : 92	93
5^*d*^	Ethyl acrylate	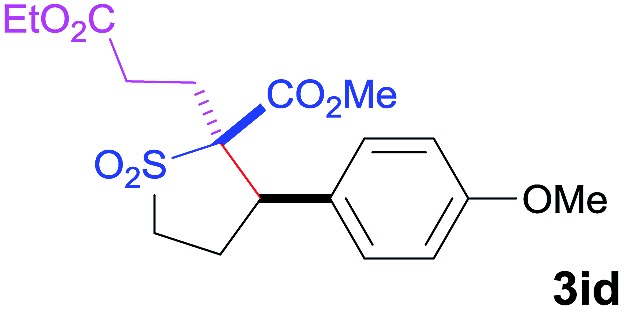	60	4 : 96	93

^*a*^Compound **2** was treated with 1.2 equiv. of NaH in THF at room temperature, followed by the addition of 1.1 equiv. of electrophile and the subsequent stirring of the reaction mixture for 12 h; isolated yields; the trans : cis diastereomeric ratios were determined by ^1^H-NMR; enantiomeric excesses were determined by chiral HPLC.

^*b*^THF/DMF (2 : 1) used as solvent.

^*c*^[2*R*,3*R*] absolute configuration determined by anomalous-dispersion effects in X-ray diffraction measurements on a crystal.

^*d*^The reaction was stirred for 3 h.

## Conclusions

In summary, we have demonstrated a fundamentally new approach based on the concept of metalloradical catalysis (MRC) for addressing asymmetric C–H alkylation with challenging acceptor/acceptor-substituted diazo reagents, such as α-methoxycarbonyl-α-diazosulfones. With the development of the new *D*_2_-symmetric chiral amidoporphyrin 3,5-DiiPr-(4′-*t*Bu)XuPhyrin (**P3**) as the supporting ligand, we have shown that its Co(ii) complex [Co(**P3**)] is an effective metalloradical catalyst for asymmetric intramolecular 1,5-C–H alkylation of α-methoxycarbonyl-α-diazosulfones, producing 5-membered sulfolane derivatives in high yields with excellent stereoselectivities. In addition to its room temperature operation, the Co(ii)-based metalloradical alkylation system demonstrates several salient features, such as unusual insensitivity to the electronics of C–H substrates, excellent chemoselectivity toward allylic/allenic C–H bonds, and outstanding tolerance to functional groups. Our preliminary results suggest that the unique reactivity and selectivity profile of the Co(ii)-catalyzed C–H alkylation likely originates from the underlying radical mechanism. Efforts are underway to expand the application of Co(ii)-MRC for asymmetric C–H alkylation as well as to further its mechanistic understanding.
