# Supplementary material for: Stereoselective radical C–H alkylation with acceptor/acceptor-substituted diazo reagents via Co(ii)-based metalloradical catalysis
Source: Chem Sci. 2014 Nov 12;6(2):1219–24. doi: 10.1039/c4sc02610a (PMC4324598; doi:10.1039/c4sc02610a)
Supplement: Supplementary file 1 [file SC-006-C4SC02610A-s001.pdf]

Electronic Supplementary Material (ESI) for Chemical Science.
This journal is © The Royal Society of Chemistry 2014

Supporting Information

Stereoselective Radical C–H Alkylation with Acceptor/Acceptor-Substituted Diazo Reagents via Co(II)-Based Metalloradical Catalysis

Xin Cui, Xue Xu, Li-Mei Jin, Lukasz Wojtas, and X. Peter Zhang*
Department of Chemistry, University of South Florida, Tampa, Florida 33620-5250

General Considerations. All C-H alkylation reactions were carried out under a nitrogen atmosphere in oven-dried glassware following standard Schlenk techniques. Anhydrous benzene and other reagents were direct used as purchased from Aldrich. Thin layer chromatography was performed on Merck TLC plates (silica gel 60 F254). Flash column chromatography was performed with ICN silica gel (60 Å, 230-400 mesh, 32-63 μm). Proton and carbon nuclear magnetic resonance spectra (^{1}H NMR and ^{13}C NMR) were recorded on a Bruker250 (250 MHz) or Varian Inova600 (600 MHz) instruments with chemical shifts reported relative to residual solvent. HPLC measurements were carried out on a Shimadzu HPLC system with Whelk-O1,Chiralcel OD-H, OJ-H, and AD-H columns. HRMS data was obtained on an Agilent 1100 LC/MS ESI/TOF mass spectrometer with electrospray ionization.

Catalyst Synthesis:

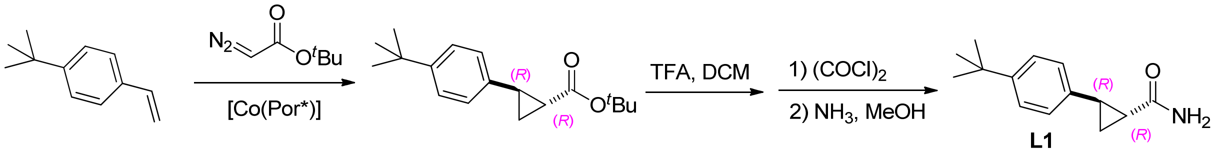

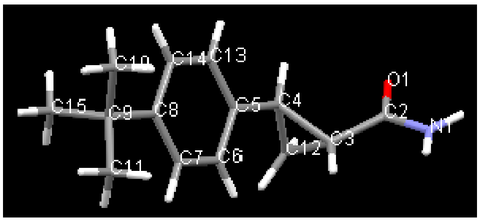

(1*R*,2*R*)-2-(4-(tert-butyl)phenyl)cyclopropanecarboxamide (L1) were synthesized according to our previous reported procedure with 97% ee.[1,2] After recrystallization the ee was improved to no less than 99%.[2] [1*R*,2*R*] absolute configuration was determined by anomalous-dispersion effects in X-ray diffraction measurements on crystal.

$[\alpha]^{20}_D$= -26.115 (c = 1, $CHCl_3$). ^{1}H NMR (500 MHz, $CDCl_3$) δ 7.32 (d, J = 8.34 Hz, 2H), 7.06 (d, J = 8.33 Hz, 2H), 5.73 (br, 2H), 2.54-2.45 (m, 1H), 1.70-1.65 (m, 1H), 1.64-1.60 (m, 1H), 1.31 (s, 9H), 1.30-1.28 (m, 1H). ^{13}C NMR (125 MHz, $CDCl_3$) δ 174.609 (C), 149.414(C), 137.457(C), 125.735(CH), 125.394(CH), 34.418(C), 31.342(CH3), 25.871(CH), 25.353(CH), 16.219(CH2). HRMS (ESI) ($[M+H]^+$) Calcd. for $C_{14}H_{20}NO$: 218.1539, Found 218.1531. IR (neat, cm^{-1}): 3403.60, 1645.76, 1423.86, 822.32, 562.68. HPLC analysis: ee = 99%. OD-H (90% hexanes: 10% isopropanol, 1.0 mL/min): t_{major} = 8.8 min, t_{minor} = 13.7 min.

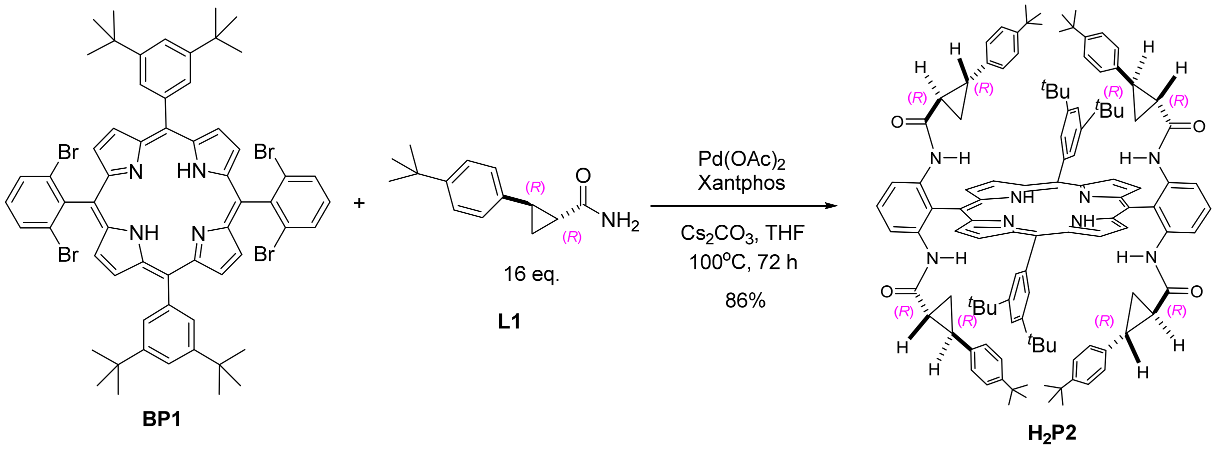

[H_2(**P2**)] were synthesized according to our previous reported procedure[3] with 86% yield. The 5,15-bis(2,6-dibromophenyl)-10,20-bis(3,5-di-*tert*-butylphenyl)-porphyrin **BP1**[3] (0.1 mmol), chiral amide **L1** (1.6 mmol), $Pd(OAc)_2$ (0.04 mmol), Xantphos (0.08 mmol), and Cs_2CO_3 (1.6 mmol) were placed in an oven-dried, resealable Schlenk tube. The tube was capped with a Teflon screwcap, evacuated, and backfilled with nitrogen. The screwcap was replaced with a rubber septum, and THF was added via syringe. The tube was purged with nitrogen for 2 min, and then the septum was replaced with the Teflon screwcap. The tube was sealed, and its contents were heated with stirring. The resulting mixture was cooled to room temperature, taken up in ethyl acetate and concentrated *in vacuo*. The crude product was then purified by flash chromatography with hexanes/EtOAc (4:1) as an eluent (Rf = 0.3). ^{1}H NMR (250 MHz, $CDC1_3$): δ 9.06 (d, J = 4.61 Hz, 4H), 8.90 (d, J = 4.37 Hz, 4H), 8.63 (br, 4H), 8.05 (s, 4H), 7.91 (t, J = 8.25 Hz, 2H), 7.86 (s, 2H), 6.64 (br, 8H), 6.59 (s, 4H), 6.25 (br, 8H), 2.29-2.22 (m, 4H), 1.53 (s, 36H), 1.34-1.25 (m, 4H), 0.95 (s, 36H), 0.59 (br, 4H), 0.18 (br, 4H), -2.49 (s, 2H). ^{13}C NMR (62.5 MHz, $CDC1_3$): δ 170.290, 149.346, 149.049, 139.752, 139.266, 136.530, 133.845, 130.482, 130.224, 125.629, 124.968, 124.762, 123.125, 121.755, 117.457, 107.859, 35.130, 34.053, 31.757, 31.070, 29.793, 26.500, 25.591, 15.773. HRMS (ESI) ($[M+H]^+$) Calcd. for C116H131N8O4: 1701.0320**,** Found 1701.0375. UV–vis (CH_2Cl_2), λ_{max} nm (log ε): 423(5.25), 518(4.17), 552(3.74), 591(3.66), 649(3.74).

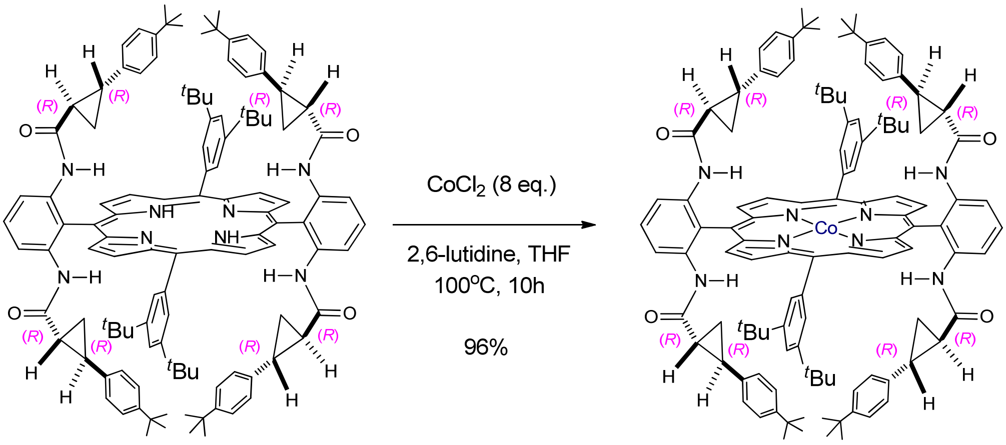

[Co(**P2**)] were synthesized according to our previous reported procedure[3] with 96% yield. Free base porphyrin [H_2(**P2**)] and anhydrous $CoCl_2$ (8 equiv) were placed in an oven-dried, resealable Schlenk tube. The tube was capped with a Teflon screwcap, evacuated, and backfilled with nitrogen. The screwcap was replaced with a rubber septum, 2,6-lutidine (8 equiv) and anhydrous THF were added via syringe. The tube was purged with nitrogen for 2 minutes, and then the septum was replaced with the Teflon screwcap. The tube was sealed, and its contents were heated with stirring. The resulting mixture was cooled to room temperature, taken up in ethyl acetate, and transferred to a separatory funnel. The mixture was washed with water 3 times and product was then purified by flash chromatography with hexanes/EtOAc (2:1) as an eluent (Rf = 0.6). HRMS (ESI) (M^{*+}) Calcd. for C116H128CoN8O4: 1756.9418**,** Found 1756.9430**.** UV–vis (CH_2Cl_2), λ_{max} nm (log ε): 415(5.17), 531(4.06).

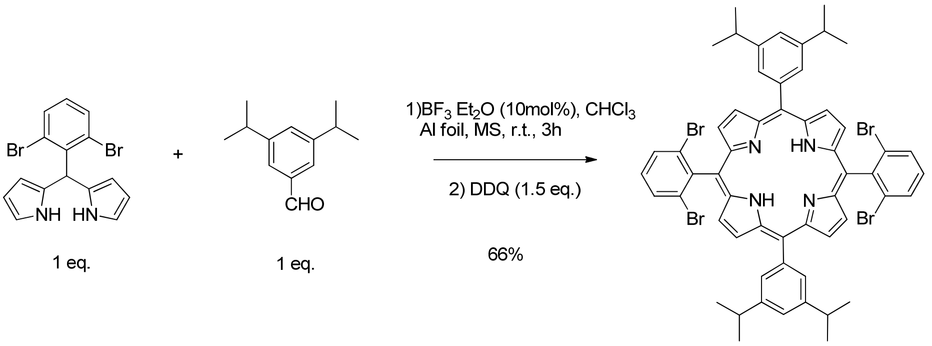

3,5-diisopropylbenzaldehyde was synthesized according to previous reported procedure.[4]

5,15-Bis(2,6-dibromophenyl)-10,20-bis(3,5-diisopropylphenyl)porphyrin were synthesized according to our previous reported procedure[3] with 66% yield. A mixture of meso-(2,6-dibromophenyl)dipyrromethane[3] (5 mmol), 3,5-diisopropylbenzaldehyde (5 mmol) in chloroform (1L) was purged with nitrogen for 10 min. Boron trifluoride diethyl etherate (0.5 mL) was added dropwise via a syringe and the flask was wrapped with aluminum foil to shield it from light. The solution was stirred under a nitrogen atmosphere at room temperature for 3 h, and 2,3-dichloro-5,6-dicyano-1,4-benzoquinone (DDQ) (6 mmol) was added as powder at one time. After 1h, 10 mL of triethylamine was added. The reaction solution was then directly poured on the top of a silica gel column that was packed with dichloromethane. The column was eluted with dichloromethane. The fractions containing product were collected and concentrated on a rotary evaporator. The residue was washed several times with hexanes to afford the pure compound.^{1}H NMR (250 MHz, $CDCl_3$): δ 8.93 (d, *J* = 4.75 Hz, 4H), 8.67 (d, *J* = 4.82 Hz, 4H), 8.06 (d, *J* = 8.06 Hz, 4H), 7.96 (d, *J* = 1.44 Hz, 4H), 7.56 (t, *J* = 8.06 Hz, 2H), 7.49 (s, 2H), 3.25-3.12 (m, 4H), 1.49 (d, *J* = 6.90 Hz, 24H), -2.52 (s, 2H). ^{13}C NMR (62.5 MHz, $CDCl_3$): δ 147.051, 143.591, 141.398, 131.492, 131.075, 130.995, 130.776, 128.518, 123.995, 121.210, 118.190, 34.359, 24.383. HRMS (ESI) ($[M+H]^+$) Calcd. for C56H51Br4N4: 1099.0811**,** Found 1099.0836**.** UV–vis (CH_2Cl_2), λ_{max} nm (log ε): 422(5.31), 516(4.42), 550(3.92), 594(3.95), 648(3.65).

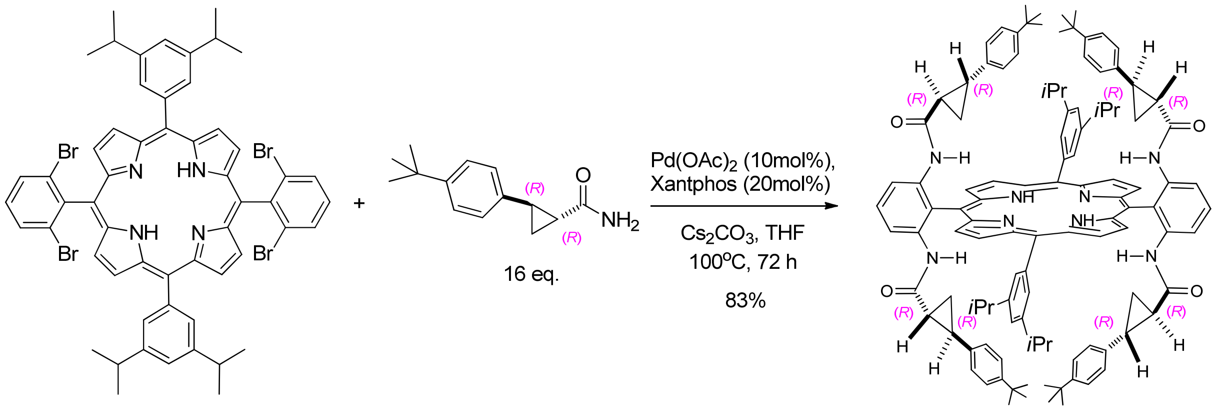

[H_2(**P3**)] were synthesized according to the above procedure for [H_2(**P2**)] with 83% yield.[3] Product was purified by flash chromatography with hexanes/EtOAc (4:1) as an eluent (Rf = 0.3). ^{1}H NMR (250 MHz, $CDCl_3$): δ 9.17 (d, *J* = 4.75 Hz, 4H), 9.01 (d, *J* = 4.68 Hz, 4H), 8.72 (br, 4H), 8.11-7.90 (m, 6H), 7.66 (s, 2H), 6.81 (d, *J* = 6.54 Hz, 8H), 6.71 (s, 4H), 6.39 (d, *J* = 5.45 Hz,

8H), 3.36-3.26 (m, 4H), 2.48-2.25 (m, 4H), 1.68-1.49 (m, 24H), 1.43-1.39 (m, 4H), 1.09 (s, 36H), 0.70 (br, 4H), 0.34 (br, 4H), -2.39 (s, 2H). ^{13}C NMR (62.5 MHz, CDCl$_3$): δ 170.358, 149.167, 147.576, 140.566, 139.381, 136.692, 133.898, 131.327, 130.598, 130.462, 125.709, 124.885, 124.691, 122.803, 117.548, 107.983, 34.397, 34.179, 31.192, 31.035, 26.569, 25.659, 24.538, 24.376, 16.023. HRMS (ESI) ([M+H]$^+$) Calcd. for C112H23N8O4: 1644.9694**,** Found 1644.9727**.** UV–vis (CH_2Cl_2), λ_{max} nm (log ε): 423(5.23), 515(4.06), 550(3.65), 593(3.62), 642(3.50).

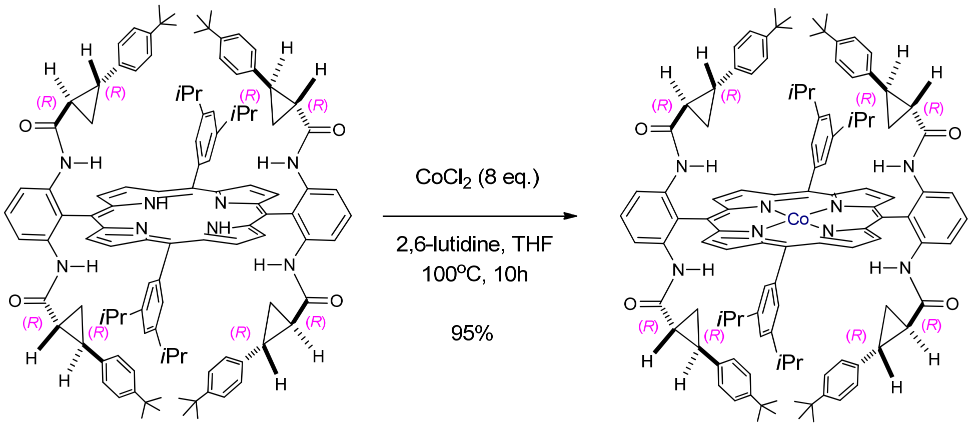

[Co(**P3**)] were synthesized according to the above general procedure for [Co(**P2**)] with 98% yield.[3] Product was purified by flash chromatography with hexanes/EtOAc (2:1) as an eluent (Rf = 0.6). HRMS (ESI) ([M+H]$^+$) Calcd. for C112H121CoN8O4: 1701.8870**,** Found 1701.8860**.** UV–vis (CH_2Cl_2), λ_{max} nm (log ε): 415(5.26), 530(4.19).

Non-chiral cobalt porphyrin complex [Co(**P4**)] were synthesized according to our previous reported procedure.[3]

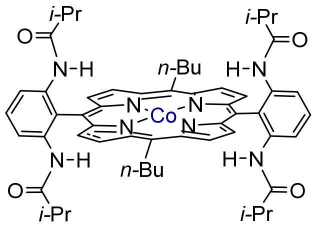

[Co(**P5**)]

Non-chiral cobalt porphyrin complex [Co(**P5**)] were also synthesized according to our previous reported procedure.[3] Product was purified by flash chromatography with hexanes/EtOAc (2:1) as an eluent (Rf = 0.3). HRMS (ESI) ([M+H]$^+$) Calcd. for C56H65CoN8O4: 972.4455**,** Found 972.4479**.** UV–vis (CH_2Cl_2), λ_{max} nm (log ε): 415(5.14), 532(4.01).

Typical Procedure for the Preparation of the Methyl 2-diazo-2-sulfonylacetates (1).

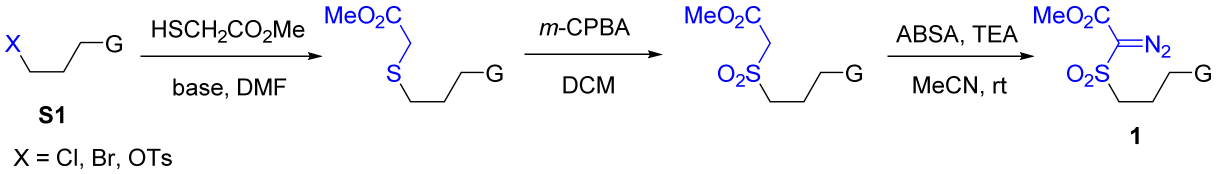

X = Cl, Br, OTs

G = Ar, alkenyl

The starting materials **S1**, including halides or tosylated alcohols are either commercially available or synthesized according to known procedures: 3-(4-nitrophenyl)propyl bromide (**S1a**)[6], 3-(4-trifluoromethyl)benzenepropanol (**S1b**)[7] were synthesized according to the known procedures. 3-phenylpropyl bromide (**S1c**), 3-(4-fluorophenyl)propyl bromide (**S1d**), 3-(4-chloro)benzenepropanol (**S1e**) were used as purchased. 3-(3-Bromo)benzenepropanol (**S1f**)[8], 3-(3,5-dichloro)benzenepropanol (**S1g**)[8], 3-(4-methyl)benzenepropanol (**S1h**)[9], were synthesized according to the known procedures. 3-(4-methoxy)benzenepropanol (**S1i**) were used as purchased. 3-(4-Hydroxyphenyl)propanol (**S1k**)[10] were synthesized according to the known procedure and 3-(4-methoxymethoxyphenyl)propanol (**S1j**) was synthesized through a typical MOM protection of **S1k**. 3-(4-Aminophenyl)propanol (**S1m**)[11] were synthesized according to the known procedure and 3-(4-acetamidophenyl)propanol (**S1l**) was synthesized through a typical acyl protection from **S1m**. 3-(1-Phenyl-1*H*-1,2,3-triazol-4-yl)propan-1-ol (**S1n**)[12] was synthesized according to the known procedure. 5-Bromo-1-pentene (**S1o**) was used as purchased. 5-Bromo-2-methyl-1-pentene (**S1p**)[13] was synthesized according to the known procedure. 4,5-Hexadien-1-ol (**S1q**)[14] was synthesized according to the known procedure. Trans-4-hexenol (**S1r**) and cis-4-hexenol (**S1s**) were used as purchased. The Diazo precursor methyl 2-sulfonylacetates were synthesized according to the known procedure.[5] Using these precursors, methyl 2-diazo-2-sulfonylacetates (**1**) were synthesized by the following diazo transfer procedure:

To a stirred solution of methyl 2-sulfonylacetate (10 mmol) in acetonitrile (10 mL) was added p-acetamidobenzenesulfonyl azide (ABSA, 12mmol). Triethylamine (TEA, 15mmol) was then added dropwise under room temperature. The reaction mixture was stirred at room temperature until the complete consumption of methyl 2-sulfonylacetate (monitored by TLC). Purification of the crude residue by flash chromatography on silica gel afforded methyl 2-diazo-2-sulfonylacetates (**1**) in typically 60-95% yields:

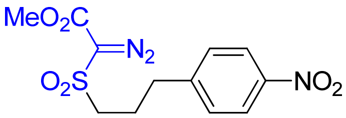

methyl 2-diazo-2-((3-(4-nitrophenyl)propyl)sulfonyl)acetate (1a):

Product was purified by flash chromatography with hexanes/EtOAc (2:1) as an eluent (Rf = 0.3).

^{1}H NMR (250 MHz, $CDCl_3$): δ 8.12 (d, *J* = 8.61 Hz, 2H), 7.36 (d, *J* = 8.56 Hz, 2H), 3.83 (s, 3H), 3.45-3.36 (m, 2H), 2.90 (t, *J* = 7.63 Hz, 2H), 2.26-2.14 (m, 2H). ^{13}C NMR (62.5 MHz, CDCl3): δ 160.342(C), 147.752(C), 146.753(C), 129.401(CH), 123.887(CH), 73.119(C), 55.612(CH2), 53.155(CH3), 33.665(CH2), 23.768(CH2). HRMS (ESI) ($[M+Na]^+$) Calcd. for $C_{12}H_{13}N_3NaO_6S$: 350.0418**,** Found 350.0424**.** IR (neat, cm^{-1}): 2144.03, 1714.82, 1508.77, 1345.42, 1214.38, 736.88.

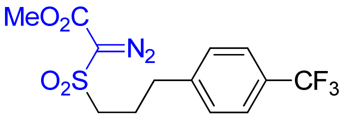

methyl 2-diazo-2-((3-(4-(trifluoromethyl)phenyl)propyl)sulfonyl)acetate (1b):

Product was purified by flash chromatography with hexanes/EtOAc (3:1) as an eluent (Rf = 0.4).

^{1}H NMR (250 MHz, $CDCl_3$): δ 7.49 (d, *J* = 8.09 Hz, 2H), 7.23 (d, *J* = 8.01 Hz, 2H), 3.76 (s, 3H), 3.35-3.26 (m, 2H), 2.78 (t, *J* = 7.52 Hz, 2H), 2.20-2.04 (m, 2H). ^{13}C NMR (62.5 MHz, CDCl3): δ 160.395(C), 143.869(C), 130.241 (d, *J* = 56 Hz; C), 128.831(CH), 125.700(CH), 123.873 (q, *J* = 250 Hz; CF3), 73.127(C), 55.656(CH2), 53.124(CH3), 33.667(CH2), 23.398(CH2). HRMS (ESI) ($[M+Na]^+$) Calcd. for $C_{13}H_{13}F_3N_2NaO_4S$: 373.0439**,** Found 373.0444**.** IR (neat, cm^{-1}): 1241.78, 1708.69, 1329.28, 1148.75, 1093.22, 797.39.

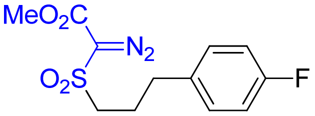

methyl 2-diazo-2-((3-(4-fluorophenyl)propyl)sulfonyl)acetate (1c):

Product was purified by flash chromatography with hexanes/EtOAc (3:1) as an eluent (Rf = 0.5).

^{1}H NMR (250 MHz, $CDCl_3$): δ 6.92 (tt, *J* = 4.80, 2.29 Hz, 2H), 6.83-6.72 (m, 2H), 3.62 (s, 3H), 3.20-3.08 (m, 2H), 2.54 (t, *J* = 7.45 Hz, 2H), 1.93 (m, 2H). ^{13}C NMR (62.5 MHz, CDC13): δ 161.667 (d, *J* = 243 Hz; CF), 160.434(C), 135.408 (d, *J* = 3.2 Hz; C), 129.969 (d, *J* = 7.9 Hz; CH), 115.704 (d, *J* = 21 Hz; CH), 55.720(CH2), 53.137(CH3), 33.081(CH2), 24.408(CH2). HRMS (ESI) ($[M+H]^+$) Calcd. for $C_{12}H_{14}FN_2O_4S$: 301.0652, Found 301.0660. IR (neat, cm^{-1}): 2128.04, 1715.01, 1509.29, 1217.86, 742.27.

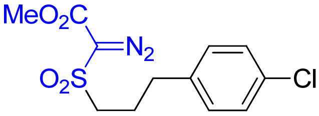

methyl 2-((3-(4-chlorophenyl)propyl)sulfonyl)-2-diazoacetate (1d):

Product was purified by flash chromatography with hexanes/EtOAc (3:1) as an eluent (Rf = 0.5).

^{1}H NMR (250 MHz, $CDCl_3$): δ 7.17 (d, *J* = 8.24 Hz, 3H), 7.03 (d, *J* = 8.30 Hz, 2H), 3.73 (s, 3H), 3.30-3.24 (m, 2H), 2.66 (t, *J* = 7.44 Hz, 2H), 2.10-1.98 (m, 2H). ^{13}C NMR (62.5 MHz, CDC13): δ 160.395(C), 138.323(C), 132.275(C), 129.898(CH), 128.800(CH), 73.042(C), 55.627(CH2), 53.163(CH3), 33.168(CH2), 24.178(CH2). HRMS (ESI) ($[M+H]^+$) Calcd. for $C_{12}H_{14}ClN_2O_4S$: 317.0356, Found 317.0367. IR (neat, cm^{-1}): 2134.71, 1721.13, 1297.30, 1149.01, 630.67.

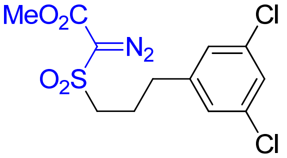

methyl 2-diazo-2-((3-(3,5-dichlorophenyl)propyl)sulfonyl)acetate (1e):

Product was purified by flash chromatography with hexanes/EtOAc (3:1) as an eluent (Rf = 0.5).

^{1}H NMR (250 MHz, $CDCl_3$): δ 7.14 (t, *J* = 1.80 Hz, 1H), 7.00 (d, *J* = 1.79 Hz, 2H), 3.77 (s, 3H), 3.33-3.27 (m, 2H), 2.67 (t, *J* = 7.56 Hz, 2H), 2.06 (m, 2H). ^{13}C NMR (62.5 MHz, CDC13): δ 160.346(C), 143.255(C), 135.106(C), 127.028(CH), 126.851(CH), 73.128(C), 55.530(CH2), 53.173(CH3), 33.290(CH2), 23.871(CH2). HRMS (ESI) ($[M+Na]^+$) Calcd. for $C_{12}H_{12}Cl_2N_2NaO_4S$: 372.9787, Found 372.9798. IR (neat, cm^{-1}): 2130.51, 1718.44, 1336.61,

1217.59, 626.34.

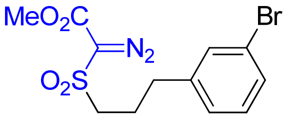

methyl 2-((3-(3-bromophenyl)propyl)sulfonyl)-2-diazoacetate (1f):

Product was purified by flash chromatography with hexanes/EtOAc (3:1) as an eluent (Rf = 0.5). ^{1}H NMR (250 MHz, $CDCl_3$): δ 7.15 (m,5H), 3.74 (s,3H), 3.34-3.22 (m,2H), 2.66 (t, *J* = 7.46 Hz,2H), 2.14-1.96 (m,2H). ^{13}C NMR (62.5 MHz, CDCl3): δ 160.385(C), 142.211(C), 131.484(C), 130.352(CH), 129.738(CH), 127.213(CH), 122.703(CH), 73.107(C), 55.675(CH2), 53.179(CH3), 33.475(CH2), 24.117(CH2). HRMS (ESI) ($[M+H]^+$) Calcd. for $C_{12}H_{14}BrN_2O_4S$: 360.9851**,** Found 360.9852**.** IR (neat, cm^{-1}): 2129.64, 1718.89, 1337.57, 1217.17, 625.98.

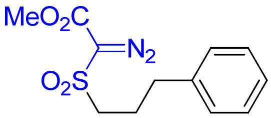

methyl 2-diazo-2-((3-phenylpropyl)sulfonyl)acetate (1g):

Product was purified by flash chromatography with hexanes/EtOAc (3:1) as an eluent (Rf = 0.5). ^{1}H NMR (250 MHz, $CDCl_3$): δ 7.39-6.99 (m, 5H), 3.75 (s, 3H), 3.36-3.18 (m, 2H), 2.70 (t, *J* = 7.42 Hz, 2H), 2.09 (m, 2H). ^{13}C NMR (62.5 MHz, CDCl3): δ 160.365(C), 140.013(C), 128.674(CH), 128.496(CH), 126.504(CH), 73.008(C), 55.709(CH2), 52.992(CH3), 33.729(CH2), 24.262(CH2). HRMS (ESI) ($[M+H]^+$) Calcd. for $C_{12}H_{15}N_2O_4S$: 283.0747**,** Found 283.0753**.** IR (neat, cm^{-1}): 2127.69, 1713.09, 1294.79, 1145.68, 740.11.

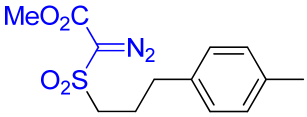

methyl 2-diazo-2-((3-(p-tolyl)propyl)sulfonyl)acetate (1h):

Product was purified by flash chromatography with hexanes/EtOAc (3:1) as an eluent (Rf = 0.5). ^{1}H NMR (250 MHz, $CDCl_3$): δ 7.05-6.85 (m, 4H), 3.68 (s, 3H), 3.27-3.21 (m, 2H), 2.60 (t, *J* = 7.39 Hz, 2H), 2.20 (s, 3H), 2.01 (m, 2H). ^{13}C NMR (62.5 MHz, CDCl3): δ 160.445(C), 136.768(C), 136.029(C), 129.401(CH), 128.381(CH), 73.064(C), 55.849(CH2), 53.069(CH3), 33.416(CH2), 24.419(CH2), 21.060(CH3). HRMS (ESI) ($[M+H]^+$) Calcd. for $C_{13}H_{17}N_2O_4S$: 297.0903**,** Found 297.0912**.** IR (neat, cm^{-1}): 2131.10, 1716.78, 1335.60, 1296.09, 738.47.

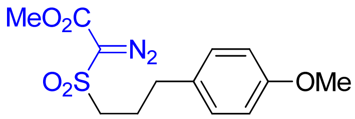

methyl 2-diazo-2-((3-(4-methoxyphenyl)propyl)sulfonyl)acetate (1i):

Product was purified by flash chromatography with hexanes/EtOAc (3:1) as an eluent (Rf = 0.4). ^{1}H NMR (250 MHz, $CDCl_3$): δ 7.10 (d, *J* = 8.65 Hz, 2H), 6.85 (d, *J* = 8.66 Hz, 2H), 3.82 (s, 3H), 3.78 (s, 3H), 3.42-3.31 (m, 2H), 2.72 (t, *J* = 7.40 Hz, 2H), 2.12 (m, 2H). ^{13}C NMR (62.5 MHz, CDCl3): δ 160.432(C), 158.331(C), 131.740(C), 129.422(CH), 114.118(CH), 73.041(C), 55.795(CH2), 55.292(CH3), 53.082(CH3), 32.951(CH2), 24.520(CH2). HRMS (ESI) ($[M+H]^+$) Calcd. for $C_{13}H_{17}N_2O_5S$: 313.0852, Found 313.0862. IR (neat, cm^{-1}): 2134.89, 1711.77, 1335.39, 1148.52, 741.66.

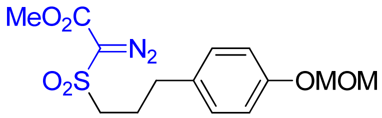

methyl 2-diazo-2-((3-(4-(methoxymethoxy)phenyl)propyl)sulfonyl)acetate (1j):

Product was purified by flash chromatography with hexanes/EtOAc (2:1) as an eluent (Rf = 0.4). ^{1}H NMR (250 MHz, $CDCl_3$): δ 7.01 (d, *J* = 8.61 Hz, 2H), 6.89 (d, *J* = 8.65 Hz, 2H), 5.06 (s, 2H), 3.74 (s, 3H), 3.38 (s, 3H), 3.33-3.20 (m, 2H), 2.63 (t, *J* = 7.37 Hz, 2H), 2.04 (m, 2H). ^{13}C NMR (62.5 MHz, CDCl3): δ 160.455(C), 155.978(C), 133.054(C), 129.458(CH), 116.567(CH), 94.546(CH2), 73.087(C), 55.990(CH3), 55.809(CH2), 53.105(CH3), 33.040(CH2), 24.481(CH2). HRMS (ESI) ($[M+H]^+$) Calcd. for $C_{14}H_{18}N_2O_6SNa$: 365.0778, Found 365.0786. IR (neat, cm^{-1}): 2129.07, 1715.22, 1295.81, 1147.29, 740.72.

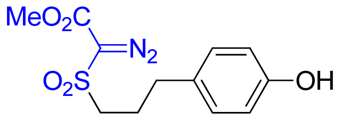

methyl 2-diazo-2-((3-(4-hydroxyphenyl)propyl)sulfonyl)acetate (1k):

Product was purified by flash chromatography with hexanes/EtOAc (2:1) as an eluent (Rf = 0.3). ^{1}H NMR (250 MHz, $CDCl_3$): δ 6.94 (d, *J* = 8.42 Hz, 2H), 6.70 (d, *J* = 8.46 Hz, 2H), 5.49 (s, 1H), 3.75 (s, 3H), 3.35-3.21 (m, 2H), 2.61 (t, *J* = 7.33 Hz, 2H), 2.10-1.98 (m, 2H). ^{13}C NMR (62.5 MHz, CDCl3): δ 160.594(C), 154.482(C), 131.548(C), 129.583(CH), 115.612(CH), 55.856(CH2), 53.248(CH3), 32.974(CH2), 24.490(CH2). HRMS (ESI) ($[M+H]^+$) Calcd. for

$C_{12}H_{15}N_2O_5S$: 299.0696**,** Found 299.0702**.** IR (neat, cm^{-1}): 3450.00, 2137.39, 1727.07, 1293.04, 1217.25, 832.44.

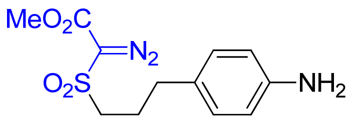

methyl 2-((3-(4-aminophenyl)propyl)sulfonyl)-2-diazoacetate (1l):

Product was purified by flash chromatography with hexanes/EtOAc (1:1) as an eluent (Rf = 0.3).

^{1}H NMR (250 MHz, $CDCl_3$): δ 6.86 (d, *J* = 8.22 Hz, 2H), 6.54 (d, *J* = 8.27 Hz, 2H), 3.74 (s, 3H), 3.64-3.34 (br, 2H), 3.34-3.18 (m, 2H), 2.56 (t, *J* = 7.33 Hz, 2H), 2.07-1.94 (m, 2H). ^{13}C NMR (62.5 MHz, CDCl3): δ 160.498(C), 145.137(C), 129.269(C), 121.904(CH), 115.384(CH), 73.068(C), 55.868(CH2), 53.139(CH3), 33.007(CH2), 24.576(CH2). HRMS (ESI) ([M+H]$^+$) Calcd. for $C_{12}H_{16}N_3O_4S$: 298.0856**,** Found 298.0863**.** IR (neat, cm^{-1}): 2124.92, 1715.57, 1299.03, 1142.19, 622.93.

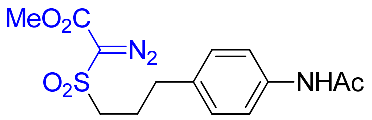

methyl 2-((3-(4-acetamidophenyl)propyl)sulfonyl)-2-diazoacetate (1m):

Product was purified by flash chromatography with hexanes/EtOAc (1:1.5) as an eluent (Rf = 0.3).

^{1}H NMR (250 MHz, $CDCl_3$): δ 8.00 (s, 1H), 7.38 (d, *J* = 8.39 Hz, 2H), 7.01 (d, *J* = 8.37 Hz, 2H), 3.74 (s, 3H), 3.34-3.20 (m, 2H), 2.65 (t, *J* = 7.33 Hz, 2H), 2.03 (m, 5H). ^{13}C NMR (62.5 MHz, CDCl3): δ 168.924(C), 160.412(C), 136.723(C), 135.442(C), 128.849(CH), 120.426(CH), 73.062(C), 55.822(CH2), 53.162(CH3), 33.227(CH2), 24.404(CH2), 24.221(CH3). HRMS (ESI) ([M+H]$^+$) Calcd. for $C_{14}H_{18}N_3O_5S$: 340.0961**,** Found 340.0966**.** IR (neat, cm^{-1}): 2133.04, 1711.39, 1325.48, 1224.19, 736.59.

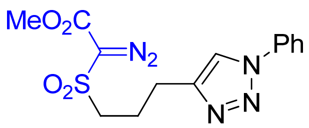

methyl 2-diazo-2-((3-(1-phenyl-1H-1,2,3-triazol-4-yl)propyl)sulfonyl)acetate (1n):

Product was purified by flash chromatography with hexanes/EtOAc (1:2) as an eluent (Rf = 0.5).

^{1}H NMR (250 MHz, $CDCl_3$): δ 7.79 (s, 1H), 7.63 (m, 2H), 7.48-7.28 (m, 3H), 3.74 (s, 3H), 3.49-3.39 (m, 2H), 2.89 (t, *J* = 7.22 Hz, 2H), 2.28-2.16 (m, 2H). ^{13}C NMR (62.5 MHz, CDCl3): δ

160.380(C), 146.383(C), 137.029(C), 129.772(CH), 128.707(CH), 120.409(CH), 119.767(CH), 73.046(C), 55.637(CH2), 53.126(CH3), 23.739(CH2), 22.482(CH2). HRMS (ESI) ($[M+Na]^+$) Calcd. for $C_{14}H_{15}N_5NaO_4S$: 372.0736, Found 372.0749. IR (neat, cm^{-1}): 2147.09, 1728.70, 1335.78, 1140.71, 629.10.

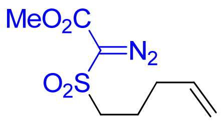

methyl 2-diazo-2-(pent-4-en-1-ylsulfonyl)acetate (1o):

Product was purified by flash chromatography with hexanes/EtOAc (3:1) as an eluent (Rf = 0.5). ^1H NMR (250 MHz, $CDCl_3$): δ 5.69 (tdd, J = 17.02, 10.32, 6.65 Hz, 1H), 5.03 (dd, J = 7.79, 1.26 Hz, 1H), 4.97 (s, 1H), 3.80 (s, 3H), 3.35-3.29 (m, 2H), 2.23-2.04 (m, 2H), 1.99-1.72 (m, 2H). ^{13}C NMR (62.5 MHz, CDCl3): δ 160.458(C), 136.112(CH), 116.699(CH2), 72.941(C), 55.809(CH2), 53.117(CH3), 31.754(CH2), 21.750(CH2). HRMS (ESI) ($[M+H]^+$) Calcd. for $C_8H_{13}N_2O_4S$: 233.0590, Found 233.0592. IR (neat, cm^{-1}): 2127.20, 1713.21, 1294.51, 1143.75, 740.59.

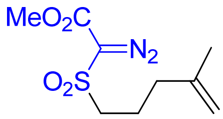

methyl 2-diazo-2-((4-methylpent-4-en-1-yl)sulfonyl)acetate (1p):

Product was purified by flash chromatography with hexanes/EtOAc (3:1) as an eluent (Rf = 0.5). ^1H NMR (250 MHz, $CDCl_3$): δ 4.73 (d, J = 19.79 Hz, 2H), 3.83 (s, 3H), 3.39-3.24 (m, 2H), 2.14 (t, J = 7.25 Hz, 2H), 1.95 (td, J = 18.75, 7.51 Hz, 2H), 1.69 (s, 3H). ^{13}C NMR (62.5 MHz, CDCl3): δ 160.450(C), 143.160(C), 111.819(CH2), 72.973(C), 55.921(CH2), 53.054(CH3), 35.662(CH2), 21.951(CH2), 20.362(CH3). HRMS (ESI) ($[M+Na]^+$) Calcd. for $C_9H_{14}N_2O_4NaS$: 269.0566, Found 269.0578. IR (neat, cm^{-1}): 2127.24, 1713.81, 1294.58, 1147.20, 739.77.

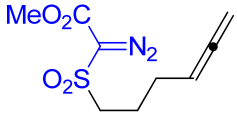

methyl 2-diazo-2-(hexa-4,5-dien-1-ylsulfonyl)acetate (1q):

Product was purified by flash chromatography with hexanes/EtOAc (3:1) as an eluent (Rf = 0.5). ^1H NMR (500 MHz, $CDCl_3$): δ 5.09 (p, J = 6.58 Hz, 1H), 4.73 (td, J = 6.63, 3.29 Hz, 2H), 3.86

(s, 3H), 3.47-3.38 (m, 2H), 2.16 (m, 2H), 1.96 (m, 2H) ^{13}C NMR (125 MHz, CDCl3): δ 208.587(C), 160.441(C), 88.050(CH), 76.054(CH2), 55.886(CH2), 53.089(CH3), 26.231(CH2), 21.895(CH2). HRMS (ESI) ([M+H]$^+$) Calcd. for $C_9H_{13}N_2O_4S$: 245.0590**,** Found 245.0596**.** IR (neat, cm^{-1}): 2126.04, 1712.55, 1330.54, 1293.43, 1083.18, 740.07.

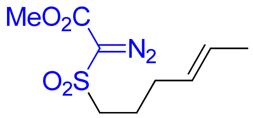

(*E*)-methyl 2-diazo-2-(hex-4-en-1-ylsulfonyl)acetate (1r) (Note: contains 5% *Z*-isomer):
Product was purified by flash chromatography with hexanes/EtOAc (3:1) as an eluent (Rf = 0.5).
^{1}H NMR (500 MHz, $CDCl_3$): δ 5.49 (m, 1H), 5.35 (m, 1H), 3.87 (s, 3H), 3.39-3.34 (m, 2H), 2.14 (dt, *J* = 7.5, 6.5 Hz, 2H), 1.93-1.85 (m, 2H), 1.66 (d, *J* = 6.43 Hz, 3H) ^{13}C NMR (125 MHz, CDCl3): δ 160.495(C), 128.504(CH), 127.519(CH), 55.964(CH2), 53.077(CH3), 30.676(CH2), 22.457(CH2), 17.881(CH3). HRMS (ESI) ([M+Na]$^+$) Calcd. for $C_9H_{14}N_2O_4NaS$: 269.0566**,** Found 269.0571**.** IR (neat, cm^{-1}): 2125.19, 1713.43, 1292.97, 1142.38, 1082.38, 739.37.

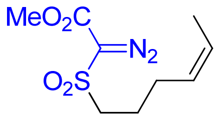

(*Z*)-methyl 2-diazo-2-(hex-4-en-1-ylsulfonyl)acetate (1s) (Note: contains 6% *E*-isomer):
Product was purified by flash chromatography with hexanes/EtOAc (3:1) as an eluent (Rf = 0.5).
^{1}H NMR (500 MHz, $CDCl_3$): δ 5.62-5.54 (m, 1H), 5.36-5.31 (m, 1H), 3.89 (s, 3H), 3.42-3.37 (m, 2H), 2.22 (dt, J = 7.0, 7.5 Hz, 2H), 1.95-1.88 (m, 2H), 1.62 (d, J = 6.84 Hz, 3H). ^{13}C NMR (125 MHz, CDCl3): δ 160.467(C), 127.621(CH), 126.370(CH), 55.994(CH2), 53.050(CH3), 24.983(CH2), 22.490(CH2), 12.822(CH3). HRMS (ESI) ([M+Na]$^+$) Calcd. for $C_9H_{14}N_2O_4NaS$: 269.0566**,** Found 269.0571**.** IR (neat, cm^{-1}): 2125.42, 1713.52, 1293.95, 1141.76, 740.29.

General Procedure for [Co(Por)]-catalyzed Intramolecular C-H Alkylation Reaction. An oven dried Schlenk tube was charged with catalyst (2 mol %). The Schlenk tube was then evacuated and back filled with nitrogen. The Teflon screw cap was replaced with a rubber septum and methyl 2-diazo-2-sulfonylacetate (**1**, 0.1 mmol) was added followed by 0.5 ml solvent. The Schlenk tube was then purged with nitrogen for 1 minute and the rubber septum was replaced with a Teflon screw cap. The reaction mixture was then stirred at room temperature. After 72 h, the reaction mixture was purified by flash chromatography. The fractions containing the product were collected and concentrated by rotary evaporation to afford the compound.

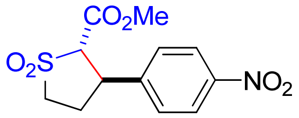

methyl 3-(4-nitrophenyl)tetrahydrothiophene-2-carboxylate 1,1-dioxide (2a): Product was purified by flash chromatography with hexanes/EtOAc (1:1) as an eluent (Rf = 0.5). $[\alpha]^{20}_{D}$ = -42.451 (*c* = 0.2, $CHCl_3$). ^{1}H NMR (250 MHz, $CDC1_3$) δ 8.22-8.11 (m, 2H), 7.49-7.38 (m, 2H), 3.93-4.09 (m, 2H), 3.762 (s, 3H), 3.45 (ddd, J = 12.91, 7.09, 1.52 Hz, 1H), 3.22 (ddd, *J* = 12.93, 6.96, 6.96 Hz, 1H), 2.43-2.20 (m, 1H), 2.63-2.45 (m, 1H). ^{13}C NMR (62.5 MHz, $CDC1_3$) δ 164.915(C), 147.742(C), 145.966(C), 128.392(CH), 124.478(CH), 71.000(CH), 53.811(CH3), 52.839(CH2), 44.498(CH), 28.076(CH2). HRMS (ESI) ($[M+Na]^+$) Calcd. for $C_{12}H_{13}NO_6SNa$: 322.0356**,** Found 322.0363**.** IR (neat, cm^{-1}): 1742.38, 1519.50, 1349.40, 1323.39, 1279.11. HPLC analysis: ee (*trans*) = 92%. Whelk(90% hexanes: 10% isopropanol, 1.0 mL/min) *trans*-isomer: t_{major} = 61.4 min, t_{minor} = 74.7 min.

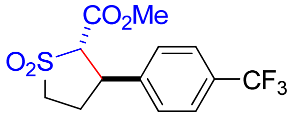

methyl 3-(4-(trifluoromethyl)phenyl)tetrahydrothiophene-2-carboxylate 1,1-dioxide (2b): Product was purified by flash chromatography with hexanes/EtOAc (3:1) as an eluent (Rf = 0.4). $[\alpha]^{20}_{D}$ = -25.626 (*c* = 0.2, $CHCl_3$). ^{1}H NMR (250 MHz, $CDC1_3$) δ 7.56 (d, *J* = 8.17 Hz, 2H), 7.37 (d, *J* = 8.14 Hz, 2H), 4.02-3.89 (m, 2H), 3.75 (s, 3H), 3.42 (ddd, *J* = 12.96, 6.97, 1.60 Hz, 1H), 3.19 (ddd, *J* = 12.92, 6.93, 6.93 Hz, 1H), 2.58-2.42 (m, 1H), 2.38-2.24 (m, 1H). ^{13}C NMR (62.5

MHz, $CDCl_3$) δ 165.051(C), 142.747(C), 130.519 (q, J = 32.5 Hz, C), 127.737(CH), 126.251 (q, J = 3.7 Hz, CH), 124.053 (q, J = 245 Hz; CF3), 71.210(CH), 53.696(CH3), 52.977(CH2), 44.658(CH), 28.224(CH2). HRMS (ESI) ($[M+Na]^+$) Calcd. for $C_{13}H_{13}F_3NaO_4S$: 345.0379, Found 345.0379. IR (neat, cm^{-1}): 1734.95, 1319.50, 1283.61, 1151.53, 1116.38. HPLC analysis: ee (*trans*) = 84%. Whelk(90% hexanes: 10% isopropanol, 1.0 mL/min) *trans*-isomer: t_{major} = 24.6 min, t_{minor} = 32.8 min.

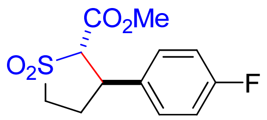

methyl 3-(4-fluorophenyl)tetrahydrothiophene-2-carboxylate 1,1-dioxide (2c): Product was purified by flash chromatography with hexanes/EtOAc (3:1) as an eluent (Rf = 0.4). $[\alpha]^{20}_D$ = -32.653 (c = 0.2, $CHCl_3$). ^1H NMR (250 MHz, $CDCl_3$) δ 7.27-7.18 (m, 2H), 7.05-6.92 (m, 2H), 3.94-3.84 (m, 2H), 3.74 (s, 3H), 3.40 (ddd, J = 12.92, 6.91, 1.50 Hz, 1H), 3.16 (ddd, J = 12.91, 6.92, 6.92 Hz, 1H), 2.54-2.38 (m, 1H), 2.34-2.16 (m, 1H). ^{13}C NMR (62.5 MHz, $CDCl_3$) δ 165.213(C), 162.491 (d, J = 246 Hz, CF), 134.488 (d, J = 3.2 Hz, C), 128.873(d, J = 8 Hz, CH), 116.197 (d, J = 21.4 Hz, CH), 71.622(CH), 53.627(CH3), 53.153(CH2), 44.359(CH), 28.510(CH2). HRMS (ESI) ($[M+H]^+$) Calcd. for $C_{12}H_{14}FO_4S$: 273.0591, Found 273.0593. IR (neat, cm^{-1}): 1737.03, 1512.97, 1320.24, 1280.39, 1226.37. HPLC analysis: ee (*trans*) = 91%. Whelk(90% hexanes: 10% isopropanol, 1.0 mL/min) *trans*-isomer: t_{major} = 25.6 min, t_{minor} = 33.2 min.

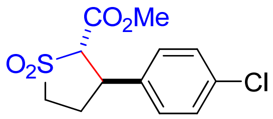

methyl 3-(4-chlorophenyl)tetrahydrothiophene-2-carboxylate 1,1-dioxide (2d): Product was purified by flash chromatography with hexanes/EtOAc (3:1) as an eluent (Rf = 0.4). $[\alpha]^{20}_D$ = -19.185 (c = 0.2, $CHCl_3$). ^1H NMR (250 MHz, $CDCl_3$) δ 7.31-7.23 (m, 2H), 7.19-7.15 (m, 2H), 3.95-3.79 (m, 2H), 3.73 (s, 3H), 3.40 (ddd, J = 12.88, 6.95, 1.60 Hz, 1H), 3.16 (ddd, J = 12.92, 6.92, 6.92 Hz, 1H), 2.53-2.38 (m, 1H), 2.35-2.14 (m, 1H). ^{13}C NMR (62.5 MHz, $CDCl_3$) δ 165.037(C), 137.135(C), 133.937(C), 129.325(CH), 128.553(CH), 71.339(CH), 53.547(CH3), 53.039(CH2), 44.326(CH), 28.238(CH2). HRMS (ESI) ($[M+H]^+$) Calcd. for $C_{12}H_{14}ClO_4S$:

289.0296, Found 289.0298. IR (neat, cm^{-1}): 1737.61, 1494.26, 1310.39, 1297.91, 1278.27. HPLC analysis: ee (*trans*) = 91%. Whelk(90% hexanes: 10% isopropanol, 1.0 mL/min) *trans*-isomer: t_{major} = 28.3 min, t_{minor} = 45.0 min.

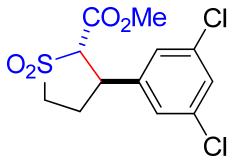

methyl 3-(3,5-dichlorophenyl)tetrahydrothiophene-2-carboxylate 1,1-dioxide (2e): Product was purified by flash chromatography with hexanes/EtOAc (3:1) as an eluent (Rf = 0.4). $[\alpha]^{20}_D$ = -26.960 (*c* = 0.2, $CHCl_3$). ^{1}H NMR (250 MHz, $CDC1_3$) δ 7.24 (t, *J* = 1.81 Hz, 1H), 7.13 (d, *J* = 1.81 Hz, 2H), 3.94-3.81 (m, 2H), 3.77 (s, 3H), 3.40 (ddd, *J* = 13.01, 6.93, 1.41 Hz, 1H), 3.16 (ddd, *J* = 12.97, 6.90, 6.90 Hz, 1H), 2.50-2.42 (m, 1H), 2.33-2.18 (m, 1H). ^{13}C NMR (62.5 MHz, $CDC1_3$) δ 165.030(C), 142.104(C), 135.832(C), 128.497(CH), 128.378(CH), 125.940(CH), 70.990(CH), 53.806(CH3), 52.861(CH2), 44.240(CH), 28.116(CH2). HRMS (ESI) ($[M+H]^+$) Calcd. for $C_{12}H_{13}Cl_2O_4S$: 322.9906, Found 322.9909. IR (neat, cm^{-1}): 1722.66, 1566.31, 1435.11, 1308.25, 1292.81, 1261.14. HPLC analysis: ee (*trans*) = 84%. Whelk(90% hexanes: 10% isopropanol, 1.0 mL/min) *trans*-isomer: t_{major} = 40.1 min, t_{minor} = 54.8 min.

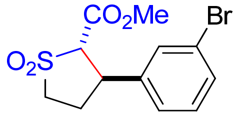

methyl 3-(3-bromophenyl)tetrahydrothiophene-2-carboxylate 1,1-dioxide (2f): Product was purified by flash chromatography with hexanes/EtOAc (3:1) as an eluent (Rf = 0.4). $[\alpha]^{20}_D$ = -25.369 (*c* = 0.2, $CHCl_3$). ^{1}H NMR (250 MHz, $CDC1_3$) δ 7.43-7.32 (m, 2H), 7.19-7.16 (m, 2H), 3.94-3.84 (m, 2H), 3.76 (s, 3H), 3.40 (ddd, *J* = 12.80, 6.81, 1.29 Hz, 1H), 3.16 (ddd, *J* = 12.95, 6.88, 6.88 Hz, 1H), 2.50-2.42 (m, 1H), 2.35-2.22 (m, 1H). ^{13}C NMR (62.5 MHz, $CDC1_3$) δ 165.118(C), 141.001(C), 131.390(C), 130.850(CH), 130.365(CH), 125.984(CH), 123.242(CH), 71.279(CH), 53.750(CH3), 53.043(CH2), 44.576(CH), 28.339(CH2). HRMS (ESI) ($[M+NH_4]^+$) Calcd. for $C_{12}H_{17}BrNO_4S$: 350.0056, Found 350.0055. IR (neat, cm^{-1}): 1741.54, 1321.61, 1277.79, 1174.73, 1119.48. HPLC analysis: ee (*trans*) = 88%. Whelk(90% hexanes: 10% isopropanol, 1.0 mL/min) *trans*-isomer: t_{major} = 27.8 min, t_{minor} = 38.5 min.

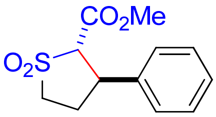

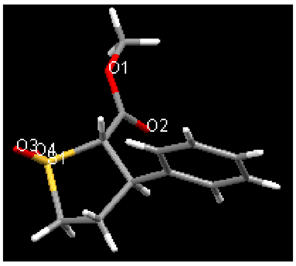

(2S,3R)-methyl 3-phenyltetrahydrothiophene-2-carboxylate 1,1-dioxide (2g): Product was purified by flash chromatography with hexanes/EtOAc (3:1) as an eluent (Rf = 0.4). $[\alpha]^{20}_{D}$ = -22.201 (*c* = 0.2, $CHCl_3$). ^1H NMR (250 MHz, $CDCl_3$) δ 7.28 (m, 5H), 4.00-3.82 (m, 2H), 3.74 (s, 3H), 3.40 (ddd, *J* = 12.91, 6.90, 1.46 Hz, 1H), 3.17 (ddd, *J* = 12.90, 6.90, 6.90 Hz, 1H), 2.54-2.40 (m, 1H), 2.39-2.21 (m, 1H). ^{13}C NMR (62.5 MHz, $CDCl_3$) δ 165.293(C), 138.717(C), 129.246(CH), 128.148(CH), 127.197(CH), 71.545(CH), 53.586(CH3), 53.200(CH2), 45.067(CH), 28.472(CH2). HRMS (ESI) ($[M+Na]^+$) Calcd Calcd. for $C_{12}H_{14}NaO_4S$: 277.0505**,** Found 277.0503. IR (neat, cm^{-1}): 1731.58, 1321.27, 1304.82, 1281.99, 1267.05. HPLC analysis: ee (*trans*) = 90%. Whelk(90% hexanes: 10% isopropanol, 1.0 mL/min) *trans*-isomer: t_{major} = 27.7 min, t_{minor} = 35.8 min.

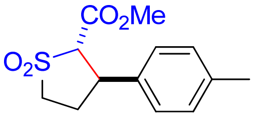

methyl 3-(p-tolyl)tetrahydrothiophene-2-carboxylate 1,1-dioxide (2h): Product was purified by flash chromatography with hexanes/EtOAc (3:1) as an eluent (Rf = 0.4). $[\alpha]^{20}_{D}$ = -37.175 (*c* = 0.2, $CHCl_3$). ^1H NMR (250 MHz, $CDCl_3$) δ 7.17-7.04 (m, 4H), 3.97-3.77 (m, 2H), 3.73 (s, 3H), 3.38 (ddd, *J* = 12.86, 6.89, 1.61 Hz, 1H), 3.15 (ddd, *J* = 12.88, 6.93, 6.93 Hz, 1H), 2.53-2.36 (m, 1H), 2.36-2.13 (m, 1H), 2.26 (s, 3H). ^{13}C NMR (62.5 MHz, $CDCl_3$) δ 165.316(C), 137.960(C), 135.726(C), 129.885(CH), 127.052(CH), 71.654(CH), 53.511(CH3), 53.285(CH2), 44.807(CH), 28.548(CH2), 21.100(CH3). HRMS (ESI) ($[M+H]^+$) Calcd. for $C_{13}H_{17}O_4S$: 269.0842, Found 269.0841. IR (neat, cm^{-1}): 1742.43, 1516.45, 1308.91, 1284.14, 1253.43. HPLC analysis: ee (*trans*) = 92%. Whelk(90% hexanes: 10% isopropanol, 1.0 mL/min) *trans*-isomer: t_{major} = 28.6 min, t_{minor} = 40.9 min.

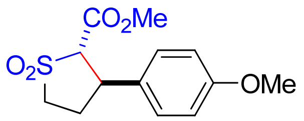

methyl 3-(4-methoxyphenyl)tetrahydrothiophene-2-carboxylate 1,1-dioxide (2i): Product was purified by flash chromatography with hexanes/EtOAc (2:1) as an eluent (Rf = 0.4). $[\alpha]^{20}_{D}$ = -83.267 (*c* = 0.2, $CHCl_3$). ^1H NMR (250 MHz, $CDC1_3$) δ 7.18-7.10 (m, 2H), 6.84-6.79 (m, 2H), 3.92-3.80 (m, 2H), 3.72 (s, 3H), 3.72 (s, 3H), 3.38 (ddd, *J* = 12.80, 6.81, 1.29 Hz, 1H), 3.15 (ddd, *J* = 12.89, 6.93, 6.93 Hz, 1H), 2.48-2.38 (m, 1H), 2.33-2.21 (m, 1H). ^{13}C NMR (62.5 MHz, $CDC1_3$) δ 165.323(C), 159.365(C), 130.652(C), 128.273(CH), 114.577(CH), 71.759(CH), 55.372(CH3), 53.503(CH2), 53.318(CH3), 44.472(CH), 28.576(CH2). HRMS (ESI) ($[M+Na]^+$) Calcd. for $C_{13}H_{16}NaO_5S$: 307.0611, Found 306.0604. IR (neat, cm^{-1}): 1728.44, 1515.67, 1336.49, 1318.35, 1282.93, 1269.83. HPLC analysis: ee (*trans*) = 94%. Whelk(80% hexanes: 20% isopropanol, 1.0 mL/min) *trans*-isomer: t_{major} = 25.2 min, t_{minor} = 41.4 min.

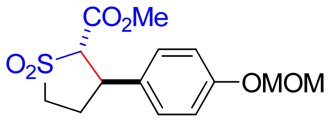

methyl 3-(4-(methoxymethoxy)phenyl)tetrahydrothiophene-2-carboxylate 1,1-dioxide (2j): Product was purified by flash chromatography with hexanes/EtOAc (1:1) as an eluent (Rf = 0.6). $[\alpha]^{20}_{D}$ = -27.858 (*c* = 0.2, $CHCl_3$). ^1H NMR (250 MHz, $CDC1_3$) δ 7.18-7.08 (m, 2H), 7.01-6.89 (m, 2H), 5.09 (s, 2H), 3.95-3.80 (m, 2H), 3.74 (s, 3H), 3.47-3.31 (m, 1H), 3.40 (s, 3H), 3.15 (ddd, *J* = 12.90, 6.95, 6.95 Hz, 1H), 2.51-2.35 (m, 1H), 2.35-2.18 (m, 1H). ^{13}C NMR (62.5 MHz, $CDC1_3$) δ 165.300(C), 156.992(C), 131.945(C), 128.320(CH), 116.921(CH), 94.429(CH2), 71.710(CH), 56.112(CH3), 53.528(CH3), 53.294(CH2), 44.481(CH), 28.559(CH2). HRMS (ESI) ([M+Na]+) Calcd. for $C_{14}H_{18}NaO_6S$: 337.0716, Found 337.0721. IR (neat, cm^{-1}): 1734.84, 1513.88, 1313.63, 1279.90, 1190.92. HPLC analysis: ee (*trans*) = 93%. Whelk(90% hexanes: 10% isopropanol, 1.0 mL/min) *trans*-isomer: t_{major} = 40.9 min, t_{minor} = 62.2 min.

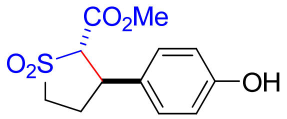

methyl 3-(4-hydroxyphenyl)tetrahydrothiophene-2-carboxylate 1,1-dioxide (2k): Product

was purified by flash chromatography with hexanes/EtOAc (1:1) as an eluent (Rf = 0.3). $[\alpha]^{20}_{D}$ = -50.214 (c = 0.2, $CHCl_3$). ^1H NMR (250 MHz, $CDC1_3$) δ 7.14-7.02 (m, 2H), 6.81-6.66 (m, 2H), 5.16 (s, 1H), 3.96-3.78 (m, 2H), 3.73 (s, 3H), 3.38 (ddd, J = 12.87, 6.91, 1.48 Hz, 1H), 3.15 (ddd, J = 12.92, 6.92, 6.92 Hz, 1H), 2.51-2.35 (m, 1H), 2.35-2.16 (m, 1H). ^{13}C NMR (62.5 MHz, $CDC1_3$) δ 165.386(C), 155.521(C), 130.686(C), 128.482(CH), 116.059(CH), 71.782(CH), 53.599(CH3), 53.322(CH2), 44.479(CH), 28.579(CH2). HRMS (ESI) ($[M+Na]^+$) Calcd. for C12H14NaO5S: 293.0454, Found 293.0458. IR (neat, cm^{-1}): 3450.00, 1735.32, 1516.57, 1308.31, 1278.99, 1259.24. HPLC analysis: ee (*trans*) = 91%. Whelk(90% hexanes: 10% isopropanol, 1.0 mL/min) *trans*-isomer: t_{major} = 53.5 min, t_{minor} = 99.2 min.

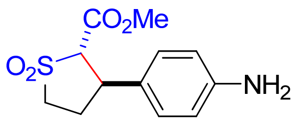

methyl 3-(4-aminophenyl)tetrahydrothiophene-2-carboxylate 1,1-dioxide (2l): Product was purified by flash chromatography with hexanes/EtOAc (1:1.3) as an eluent (Rf = 0.3). $[\alpha]^{20}_{D}$ = -6.312 (c = 0.2, $CHCl_3$). ^1H NMR (250 MHz, $CDC1_3$) δ 6.99 (d, J = 8.11 Hz, 2H), 6.56 (d, J = 8.12 Hz, 2H), 3.89-3.75 (m, 2H), 3.72 (s, 3H), 3.52 (br, 2H), 3.41-3.31 (m, 1H), 3.13 (ddd, J = 12.88, 6.93, 6.93 Hz, 1H), 2.47-2.32 (m, 1H), 2.32-2.15 (m, 1H). ^{13}C NMR (62.5 MHz, $CDC1_3$) δ 165.513(C), 146.186(C), 128.298(C), 128.079(CH), 115.586(CH), 71.896(CH), 53.480(CH3), 53.417(CH2), 44.597(CH), 28.625(CH2). HRMS (ESI) ($[M+H]^+$) Calcd. for $C_{12}H_{16}NO_4S$: 270.0795, Found 270.0794. IR (neat, cm^{-1}): 1753.38, 1316.66, 1268.81, 1174.44, 1117.46. HPLC analysis: ee (*trans*) = 83% Whelk(80% hexanes: 20% isopropanol, 1.0 mL/min) *trans*-isomer: t_{major} = 56.8 min, t_{minor} = 97.0 min.

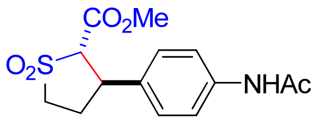

methyl 3-(4-acetamidophenyl)tetrahydrothiophene-2-carboxylate 1,1-dioxide (2m): Product was purified by flash chromatography with hexanes/EtOAc (1:2) as an eluent (Rf = 0.5). $[\alpha]^{20}_{D}$ = -10.514 (c = 0.2, $CHCl_3$). ^1H NMR (250 MHz, acetone-d6) δ 9.16 (s, 1H), 7.62 (d, J = 8.60 Hz, 2H), 7.39-7.26 (m, 2H), 4.08 (d, J = 11.39 Hz, 1H), 3.95-3.81 (m, 1H), 3.71 (s, 3H), 3.50 (ddd, J = 12.85, 7.26, 1.45 Hz, 1H), 3.28 (ddd, J = 12.75, 7.13, 7.13 Hz, 1H), 2.62-2.46 (m, 1H), 2.42-

2.23 (m, 1H), 2.06 (s, 3H). ^{13}C NMR (62.5 MHz, acetone-d6) δ 168.890(C), 165.993(C), 140.065(C), 134.894(C), 128.568(CH), 120.337(CH), 72.248(CH), 54.236(CH2), 53.386(CH3), 45.519(CH), 24.279(CH2). HRMS (ESI) ([M+H]$^+$) Calcd. for $C_{14}H_{18}NO_5S$: 312.0900, Found 312.0903. IR (neat, cm^{-1}): 1735.40, 1666.60, 1532.49, 1515.06, 1414.01, 1311.41. HPLC analysis: ee (*trans*) = 92%. Whelk(60% hexanes: 40% isopropanol, 1.0 mL/min) *trans*-isomer: t_{major} = 34.7 min, t_{minor} = 49.2 min.

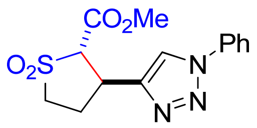

methyl 3-(1-phenyl-1H-1,2,3-triazol-4-yl)tetrahydrothiophene-2-carboxylate 1,1-dioxide (2n): Product was purified by flash chromatography with hexanes/EtOAc (1:2) as an eluent (Rf = 0.6). $[\alpha]^{20}_{D}$ = -23.511 (*c* = 0.2, $CHCl_3$). ^{1}H NMR (250 MHz, $CDCl_3$) δ 7.89 (s, 1H), 7.64 (d, *J* = 7.51 Hz, 2H), 7.49-7.38 (m, 3H), 4.28 (d, *J* = 10.45 Hz, 1H), 4.19-4.08 (m, 1H), 3.77 (s, 3H), 3.50-3.40 (m, 1H), 3.30-3.15 (m, 1H), 2.68-2.53 (m, 2H). ^{13}C NMR (62.5 MHz, $CDCl_3$) δ 163.972(C), 144.934(C), 135.796(C), 128.858(CH), 128.060(CH), 119.549(CH), 119.055(CH), 68.945(CH), 52.636(CH3), 51.735(CH2), 35.174(CH), 25.553(CH2). HRMS (ESI) ([M+H]$^+$) Calcd. for $C_{14}H_{16}N_3O_4S$: 322.0856**,** Found 322.0861. IR (neat, cm^{-1}): 1747.40, 1314.99, 1265.19, 1231.85, 1172.84. HPLC analysis: ee (*trans*) = 87%. Whelk(80% hexanes: 20% isopropanol, 1.0 mL/min) *trans*-isomer: t_{major} = 65.4 min, t_{minor} = 102.2 min.

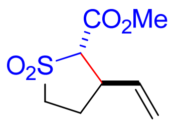

methyl 3-vinyltetrahydrothiophene-2-carboxylate 1,1-dioxide (2o): Product was purified by flash chromatography with hexanes/EtOAc (3:1) as an eluent (Rf = 0.4). $[\alpha]^{20}_{D}$ = -48.060 (*c* = 0.2, $CHCl_3$). ^{1}H NMR (250 MHz, $CDCl_3$) δ 5.70 (ddd, *J* = 17.22, 10.24, 7.06 Hz, 1H), 5.22-5.09 (m, 2H), 3.79 (s, 3H), 3.67 (d, *J* = 10.25 Hz, 1H), 3.41-3.22 (m, 2H), 3.07 (dt, *J* = 12.61, 7.01 Hz, 1H), 2.41-2.23 (m, 1H), 2.05-1.87 (m, 1H). ^{13}C NMR (62.5 MHz, $CDCl_3$) δ 165.038(C), 135.561(CH), 118.085(CH2), 69.816(CH), 53.498(CH3), 52.621(CH2), 43.232(CH), 26.547(CH2). HRMS (ESI([M+Na]$^+$) Calcd. for $C_8H_{12}NaO_4S$: 277.0349**,** Found 277.0352. IR (neat, cm^{-1}): 1732.54, 1357.71, 1314.60, 1285.74, 1264.76. HPLC analysis: ee (*trans*) = 78%.

AD-H(97% hexanes: 3% isopropanol, 1.0 mL/min) *trans*-isomer: t_{major} = 48.3 min, t_{minor} = 25.2 min.

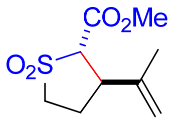

methyl 3-(prop-1-en-2-yl)tetrahydrothiophene-2-carboxylate 1,1-dioxide (2p): Product was purified by flash chromatography with hexanes/EtOAc (3:1) as an eluent (Rf = 0.4). $[\alpha]^{20}_{D}$ = -45.548 (c = 0.2, $CHCl_3$). ^1H NMR (250 MHz, $CDCl_3$) δ 4.87 (s, 1H), 4.83 (s, 1H), 3.80 (d, J = 10.25 Hz, 1H), 3.79 (s, 3H), 3.43-3.22 (m, 2H), 3.06 (dt, J = 12.74, 6.81 Hz, 1H), 2.39-2.23 (m, 1H), 2.12-1.93 (m, 1H), 1.70 (s, 3H). ^{13}C NMR (62.5 MHz, $CDCl_3$) δ 165.502(C), 141.774(C), 113.597(CH2), 68.901(CH), 53.525(CH3), 52.836(CH2), 46.306(CH), 25.769(CH2), 20.083(CH3). HRMS (ESI) ($[M+H]^+$) Calcd. for $C_9H_{15}O_4S$: 219.0686, Found 219.0679. IR (neat, cm^{-1}): 1743.86, 1436.32, 1316.73, 1274.53, 1170.36, 1149.15. HPLC analysis: ee (*trans*) = 80%. AD-H (90% hexanes: 10% isopropanol, 1.0 mL/min) *trans*-isomer: t_{major} = 12.6 min, t_{minor} = 10.0 min.

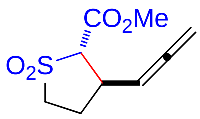

methyl 3-(propa-1,2-dien-1-yl)tetrahydrothiophene-2-carboxylate 1,1-dioxide (2q): Product was purified by flash chromatography with hexanes/EtOAc (3:1) as an eluent (Rf = 0.4). $[\alpha]^{20}_{D}$ = -29.450 (c = 0.2, $CHCl_3$). ^1H NMR (250 MHz, $CDCl_3$) δ 5.24 (dd, J = 12.40, 6.60 Hz, 1H), 4.90 (ddd, J = 6.49, 3.16, 1.02 Hz, 2H), 3.84 (s, 3H), 3.79 (d, J = 9.75 Hz, 1H), 3.44-3.35 (m, 1H), 3.32 (ddd, J = 12.75, 7.42, 3.14 Hz, 1H), 3.13 (ddd, J = 12.91, 11.50, 7.05 Hz, 1H), 2.45-2.37 (m, 1H), 2.07-1.96 (m, 1H). ^{13}C NMR (62.5 MHz, $CDCl_3$) δ 207.490(C), 164.958(C), 89.899(CH), 78.923(CH2), 69.881(CH), 53.467(CH3), 52.634(CH2), 37.853(CH), 26.625(CH2). HRMS (ESI) ($[M+Na]^+$) Calcd. for $C_9H_{12}O_4SNa$: 239.0349, Found 239.0353. IR (neat, cm^{-1}): 1732.62, 1305.28, 1278.21, 1116.17, 868.22. HPLC analysis: ee (*trans*) = 83%. AD-H (95% hexanes: 5% isopropanol, 1.0 mL/min) *trans*-isomer: t_{major} = 22.0 min, t_{minor} = 30.3 min.

Catalyst Controlled Olefin Isomerization to probe the Radical Mechanism of Co(II)-Catalyzed C–H Alkylation

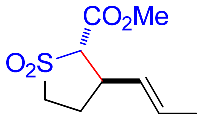

methyl (E)-3-(prop-1-en-1-yl)tetrahydrothiophene-2-carboxylate 1,1-dioxide (2r): Product was purified by flash chromatography with hexanes/EtOAc (3:1) as an eluent (Rf = 0.4). ^{1}H NMR (500 MHz, CDC1$_3$) δ 5.67 (dqd, *J* = 15.0, 6.48, 0.96 Hz, 1H), **5.34 (ddq, *J* = 15.23, 7.53, 1.56 Hz, 1H)**, 3.84 (s, 1H), 3.67 (d, *J* = 10.37 Hz, 1H), 3.40-3.33 (m, 1H), 3.31 (ddd, *J* = 12.99, 7.14, 1.55 Hz, 1H), 2.36-2.30 (m, 1H), 1.97 (ddd, *J* = 25.59, 12.57, 7.21 Hz, 1H), 1.67 (dd, *J* = 6.50, 0.96 Hz, 1H). ^{13}C NMR (125 MHz, CDC1$_3$) δ 165.129(C), 129.300(CH), 128.394(CH), 70.219(CH), 53.405(CH3), 52.735(CH2), 42.707(CH), 27.103(CH2), 17.887(CH3). HRMS (ESI) ([M+Na]$^+$) Calcd. for $C_9H_{14}O_4SNa$: 241.0505, Found 241.0510. IR (neat, cm^{-1}): 1733.29, 1438.38, 1318.28, 1267.95, 1175.48, 1118.27.

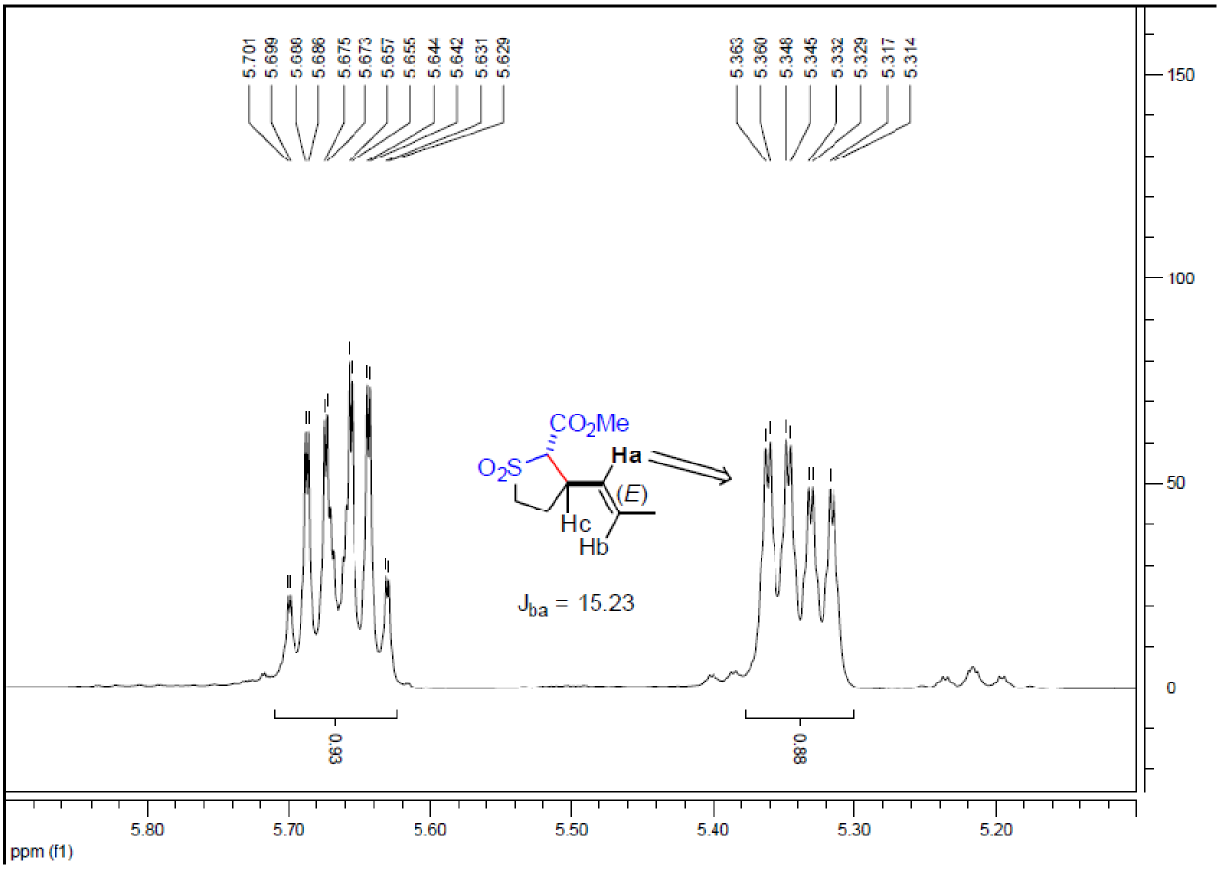

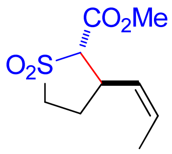

methyl (Z)-3-(prop-1-en-1-yl)tetrahydrothiophene-2-carboxylate 1,1-dioxide (2s): Product was purified by flash chromatography with hexanes/EtOAc (3:1) as an eluent (Rf = 0.4). ^{1}H NMR (500 MHz, CDC1$_3$) δ 5.72-5.60 (m, 1H), **5.22 (ddq, *J* = 10.89, 9.38, 1.78 Hz, 1H)**, 3.84 (s, 1H), 3.79-3.72 (m, 1H), 3.65 (d, *J* = 10.11 Hz, 1H), 3.33 (ddd, *J* = 12.94, 7.28, 1.86 Hz, 1H), 3.15 (dt, *J* = 12.80, 7.13 Hz, 1H), 2.33-2.26 (m, 1H), 1.99-1.89 (m, 1H), 1.72 (dd, *J* = 6.95, 1.80 Hz, 1H). ^{13}C NMR (125 MHz, CDC1$_3$) δ 165.189(C), 129.068(CH), 127.963(CH), 70.355(CH), 53.416(CH3), 52.684(CH2), 37.879(CH), 27.189(CH2), 13.214(CH3). HRMS (ESI) ([M+Na]$^+$) Calcd. for $C_9H_{14}O_4SNa$: 241.0505, Found 241.0511. IR (neat, cm^{-1}): 1733.59, 1438.48, 1312.26, 1268.89, 1172.53, 1117.23.

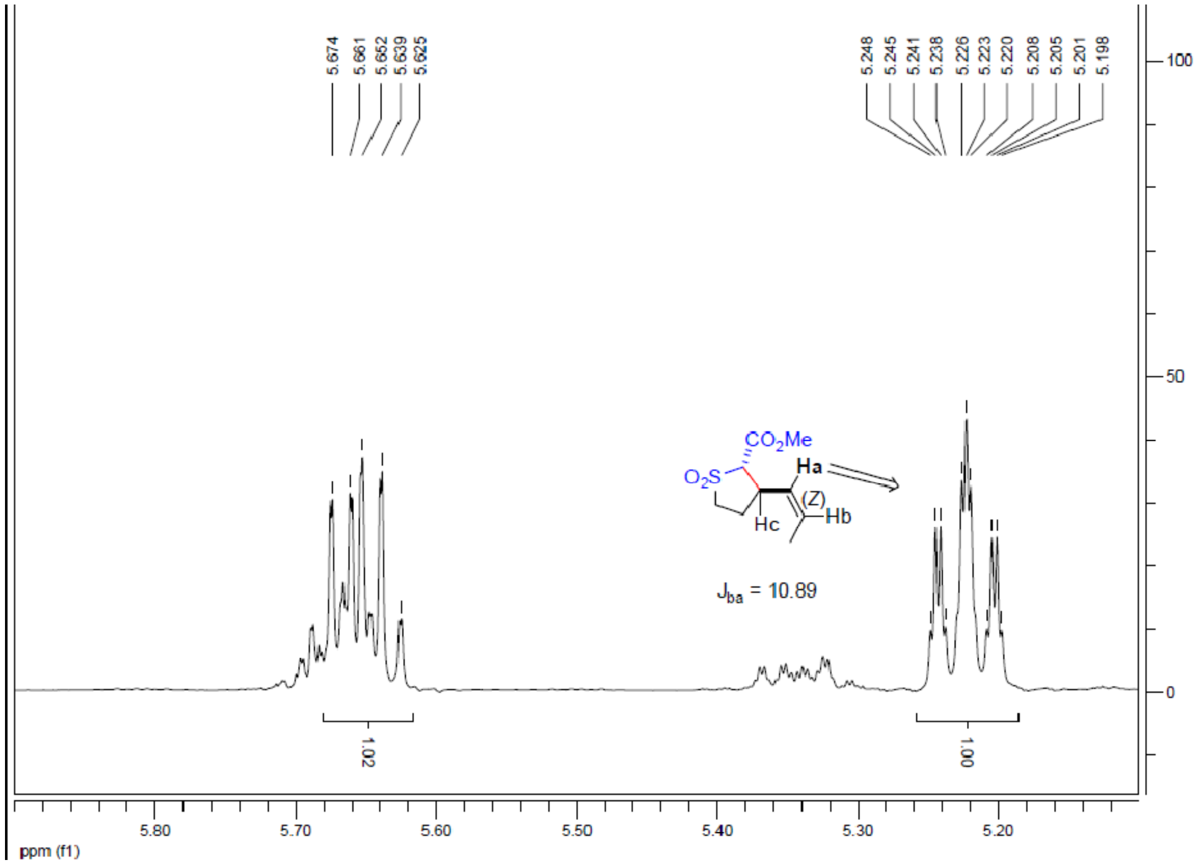

entry	diazo	[Co(P)]	yield (%) [b]	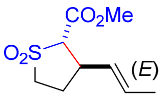 2r	:	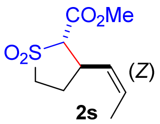 2s
1	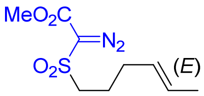 1r	[Co(**P3**)]	94	95	:	5
2		[Co(**P4**)]	96	89	:	11
3		[Co(**P5**)]	96	82	:	18
4	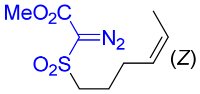 1s	[Co(**P3**)]	92	18	:	82
5		[Co(**P4**)]	94	49	:	51
6		[Co(**P5**)]	95	77	:	23

The ratio of compound **2r** and **2s** in entries 1-6 were determined by ^{1}HNMR of their mixtures. The proton of **2r** at 5.34ppm and proton of **2s** at 5.22ppm were selected for the determination.

Entry 1:

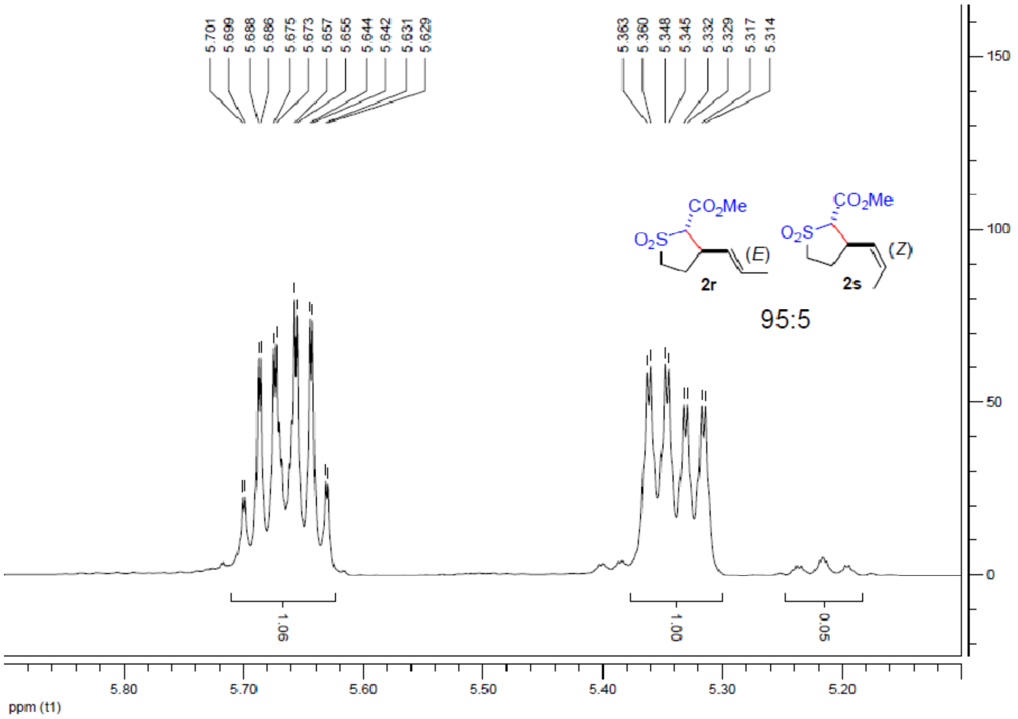

Entry 2:

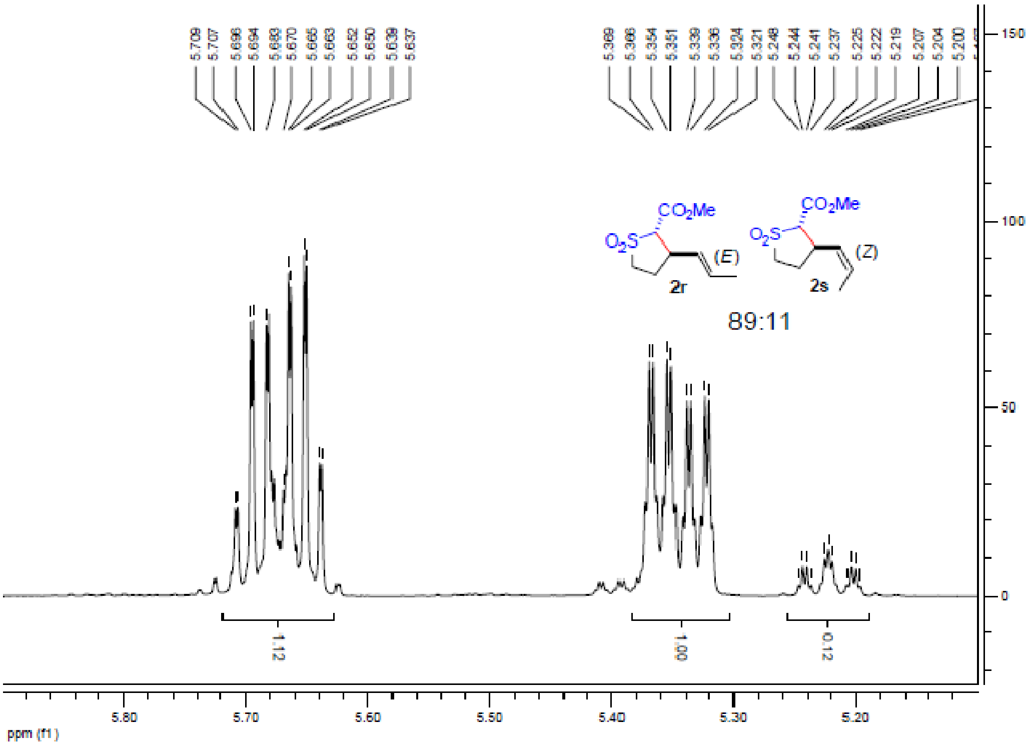

Entry 3:

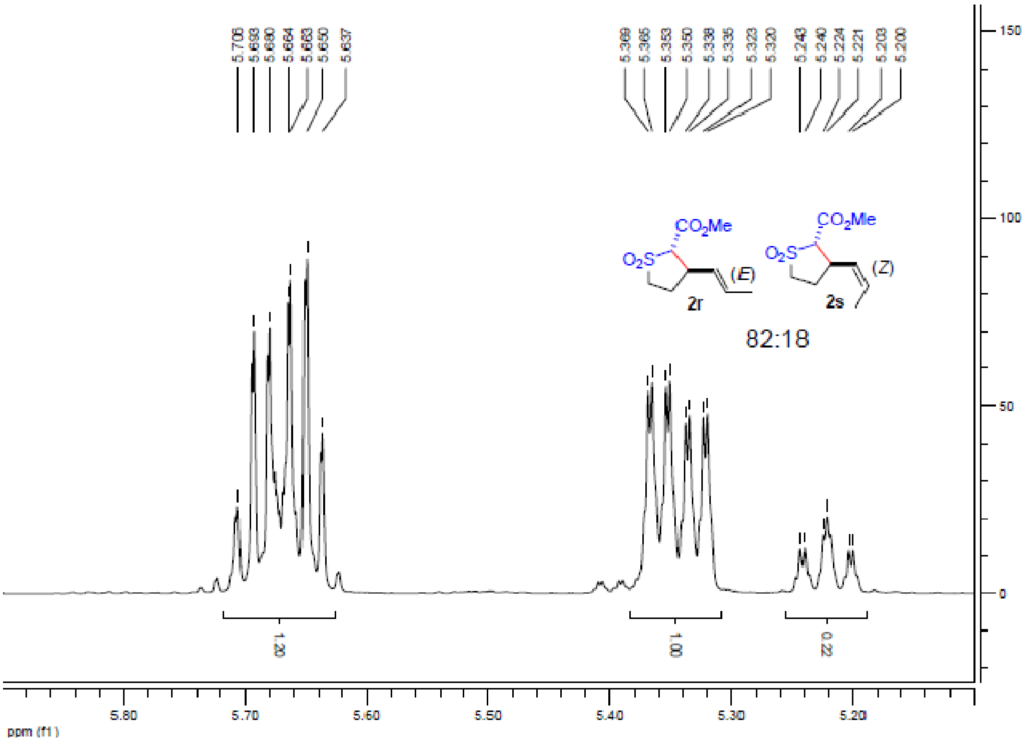

Entry 4:

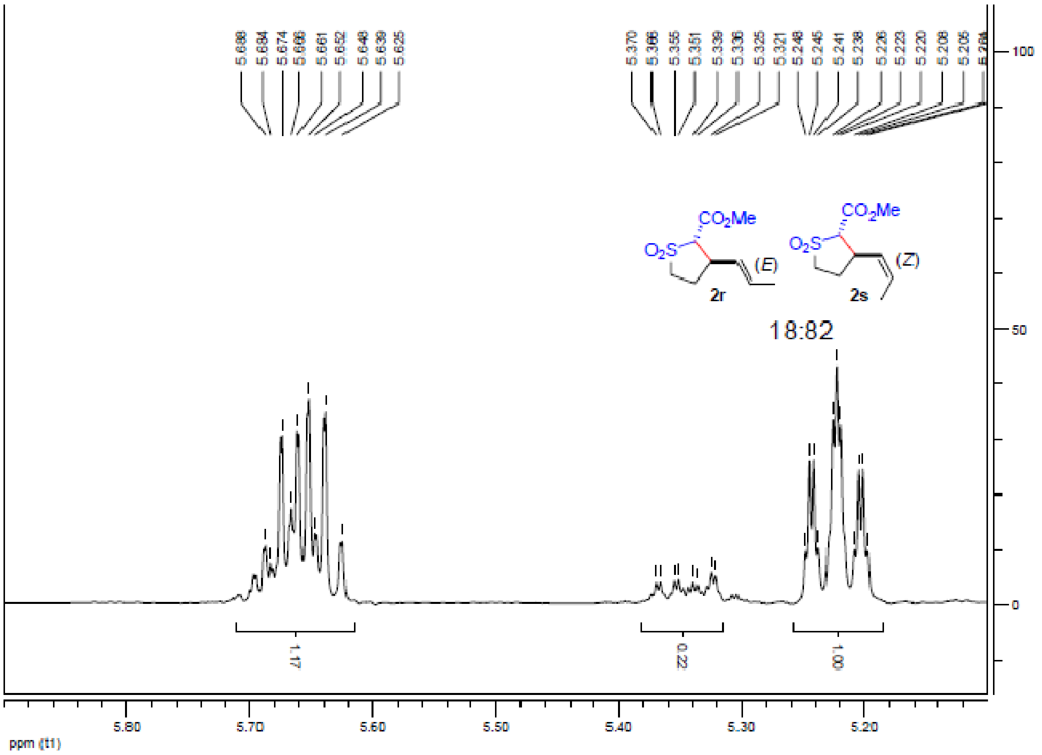

Entry 5:

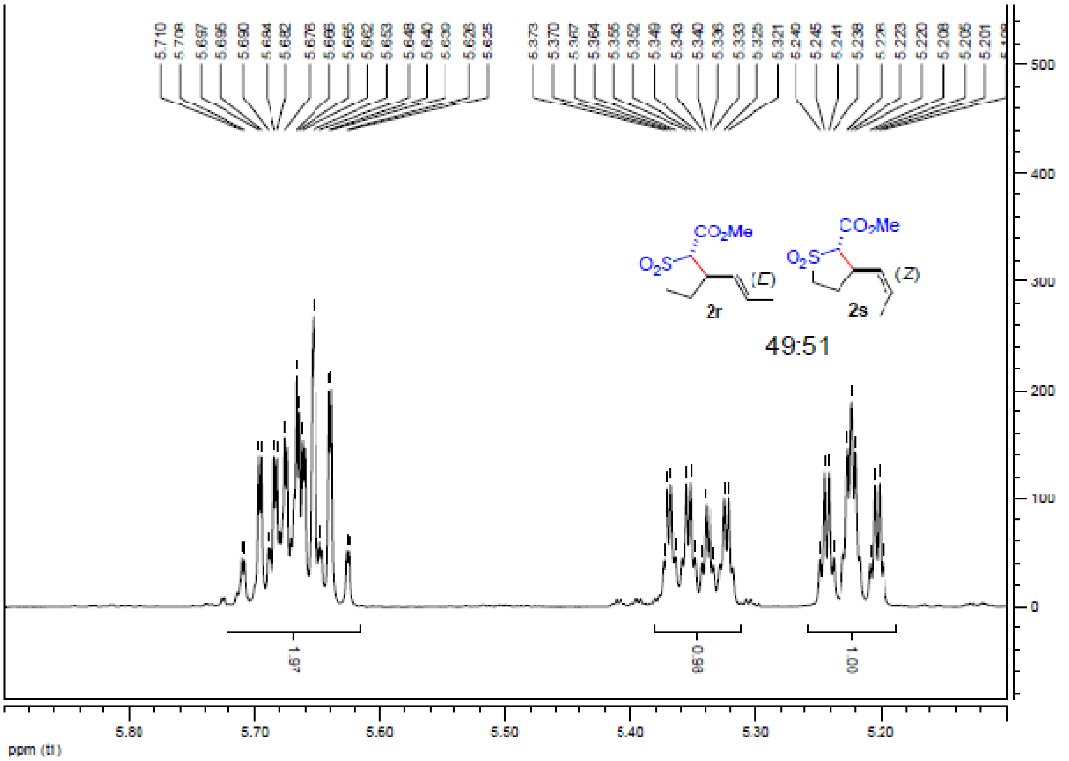

Entry 6:

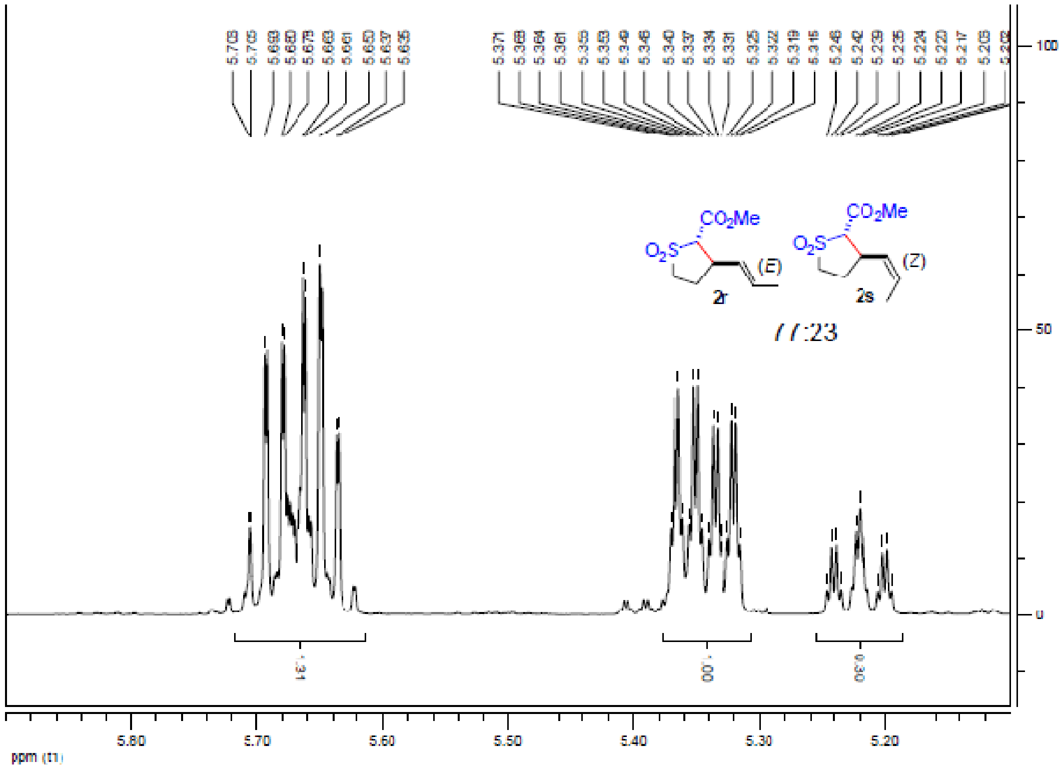

Diastereocontrolled Electrophilic Substitution Reaction of Sulfolanes 2.

Sulfolane **2** (0.1 mmol) in 1 mL of THF was treated with 1.2 equiv of NaH in THF at room temperature for 20 min, then 1.1 equiv of electrophile was added. The reaction mixture was stirred overnight and concentrated to remove THF, followed by purification of the residue by column chromatography to afford the desired product.

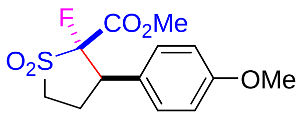

methyl 2-fluoro-3-(4-methoxyphenyl)tetrahydrothiophene-2-carboxylate 1,1-dioxide (3ia): Product was purified by flash chromatography with hexanes/EtOAc (2:1) as an eluent (Rf = 0.3). $[\alpha]^{20}_{D}$ = -45.766 (*c* = 0.2, $CHCl_3$). ^1H NMR (600 MHz, $CDC1_3$) δ 7.15 (d, *J* = 8.46 Hz, 2H), 6.81 (d, *J* = 8.56 Hz, 2H), 3.73 (s, 3H), 3.71-3.67 (m, 1H), 3.59 (s, 3H), 3.55 (dd, *J* = 13.10, 8.84 Hz, 1H), 3.36-3.26 (m, 1H), 2.87-2.75 (m, 1H), 2.44-2.40 (m, 1H). ^{13}C NMR (62.5 MHz, $CDC1_3$) δ 163.219 (d, *J* = 26.6 Hz, C), 159.760(C), 128.916(C), 125.600(CH), 114.308(CH), 105.313 (d, *J* = 243 Hz, CF), 55.346(CH3), 53.449(CH3), 50.526(CH2), 48.371 (d, *J* = 18.2 Hz, CH),

21.145 (d, J = 6.9 Hz, CH2). HRMS (ESI) ($[M+NH_4]^+$) Calcd. for $C_{13}H_{19}FNO_5S$: 320.0962, Found 320.0975. IR (neat, cm^{-1}): 1749.36, 1516.61, 1320.83, 1250.19, 1227.75. HPLC analysis: ee (*trans*) = 95%. Whelk(90% hexanes: 10% isopropanol, 1.0 mL/min) *trans*-isomer: t_{major} = 35.8 min, t_{minor} = 40.9 min.

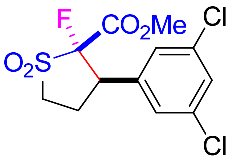

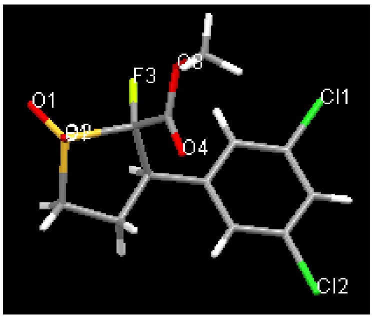

(2R,3R)-methyl 3-(3,5-dichlorophenyl)-2-fluorotetrahydrothiophene-2-carboxylate 1,1-dioxide (3ea): Product was purified by flash chromatography with hexanes/EtOAc (3:1) as an eluent (Rf = 0.4). $[\alpha]^{20}_D$ = -22.375 (c = 0.2, $CHCl_3$). ^1H NMR (600 MHz, $CDC1_3$) δ 7.32 (s, 1H), 7.18 (s, 2H), 3.77-3.72 (m, 1H), 3.70 (s, 3H), 3.63-3.60 (m, 1H), 3.39-3.34 (m, 1H), 2.84-2.80 (m, 1H), 2.54-2.49 (m, 1H). ^{13}C NMR (62.5 MHz, $CDC1_3$) δ 163.780 (d, J = 26.4 Hz, C), 137.356(C), 135.610(C), 128.992(CH), 126.363(CH), 104.562 (d, J = 245 Hz, CF), 53.784(CH3), 50.246(CH2), 48.137 (d, J = 18.3 Hz, CH), 20.951 (d, J = 6.7 Hz, CH2). HRMS (ESI) ($[M+NH_4]^+$) Calcd. for $C_{12}H_{15}Cl_2FNO_4S$: 358.0077, Found 358.0085. IR (neat, cm^{-1}): 1750.32, 1567.36, 1434.67, 1333.53, 1285.06, 1252.27. . HPLC analysis: ee (*trans*) = 84%. AD-H(98% hexanes: 2% isopropanol, 1.0 mL/min) *trans*-isomer: t_{major} = 57.0 min, t_{minor} = 69.4 min.

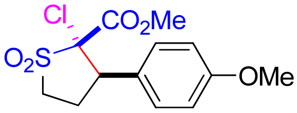

methyl 2-chloro-3-(4-methoxyphenyl)tetrahydrothiophene-2-carboxylate 1,1-dioxide (3ib): Product was purified by flash chromatography with hexanes/EtOAc (2:1) as an eluent (Rf = 0.4). $[\alpha]^{20}_D$ = -62.588 (c = 0.2, $CHCl_3$). ^1H NMR (600 MHz, $CDC1_3$) δ 7.18 (d, J = 8.78 Hz, 2H), 6.82 (d, J = 8.57 Hz, 2H), 3.74 (s, 3H), 3.72-3.66 (m, 2H), 3.65 (s, 3H), 3.40 (ddd, J = 13.29, 9.81, 7.33 Hz, 1H), 2.94-2.87 (m, 1H), 2.44-2.35 (m, 1H). ^{13}C NMR (62.5 MHz, $CDC1_3$) δ 164.186(C), 160.046(C), 129.834(C), 125.669(CH), 114.264(CH), 85.796(C), 55.358(CH3),

54.238(CH3), 52.501(CH2), 49.555(CH), 24.216(CH2). HRMS (ESI) ([M+Na]$^+$) Calcd. for $C_{13}H_{15}ClNaO_5S$: 341.0221, Found 341.0225. IR (neat, cm^{-1}):): 1755.60, 1727.23, 1514.36, 1329.24, 1248.40. HPLC analysis: ee (*trans*) = 93%. OD-H(90% hexanes: 10% isopropanol, 1.0 mL/min) *trans*-isomer: t_{major} = 38.7 min, t_{minor} = 30.9 min.

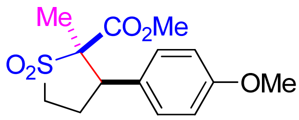

methyl 3-(4-methoxyphenyl)-2-methyltetrahydrothiophene-2-carboxylate 1,1-dioxide (3ic): Product was purified by flash chromatography with hexanes/EtOAc (3:1) as an eluent (Rf = 0.5). $[\alpha]^{20}_D$ = -33.609 (*c* = 0.2, $CHCl_3$). ^{1}H NMR (600 MHz, $CDCl_3$) δ 7.11 (d, *J* = 8.50 Hz, 2H), 6.85 (d, *J* = 8.57 Hz, 2H), 3.78 (s, 3H), 3.69 (dt, *J* = 13.27, 3.27 Hz, 1H), 3.65 (s, 3H), 3.35 (dd, *J* = 12.40, 7.51 Hz, 1H), 3.32-3.28 (m, 1H), 2.93-2.86 (m, 1H), 2.41-2.30 (m, 1H), 1.48 (s, 3H). ^{13}C NMR (62.5 MHz, $CDCl_3$) δ 167.822(C), 159.646(C), 129.589(C), 127.790(CH), 114.259(CH), 70.789(C), 55.347(CH3), 52.940(CH3), 50.806(CH3), 50.185(CH2), 25.043(CH), 14.005(CH2). HRMS (ESI) ([M+H]$^+$) Calcd. for $C_{14}H_{19}O_5S$: 299.0948, Found 299.0962. IR (neat, cm^{-1}):): 1727.50, 1514.10, 1454.10, 1306.83, 1278.19, 1248.58. HPLC analysis: ee (*trans*) = 93%. AD-H(95% hexanes: 5% isopropanol, 1.0 mL/min) *trans*-isomer: t_{major} = 27.0 min, t_{minor} = 22.8 min.

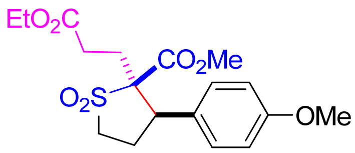

methyl 2-(3-ethoxy-3-oxopropyl)-3-(4-methoxyphenyl)tetrahydrothiophene-2-carboxylate 1,1-dioxide (3id): Product was purified by flash chromatography with hexanes/EtOAc (2:1) as an eluent (Rf = 0.2). $[\alpha]^{20}_D$ = -4.019 (*c* = 0.2, $CHCl_3$). ^{1}H NMR (600 MHz, $CDCl_3$) δ 7.06 (d, *J* = 8.43 Hz, 2H), 6.84 (d, *J* = 8.49 Hz, 2H), 4.11-4.05 (m, 2H), 3.78 (s, 3H), 3.72 (ddd, *J* = 12.80, 2.93, 1.06 Hz, 1H), 3.66 (s, 3H), 3.48 (dd, *J* = 12.30, 7.47 Hz, 1H), 3.35-3.26 (m, 1H), 2.83-2.69 (m, 2H), 2.54-2.41 (m, 1H), 2.38-2.35 (m, 1H), 2.32-2.25 (m, 1H), 2.23-2.17 (m, 1H), 1.20 (t, *J* = 7.13 Hz, 3H),. ^{13}C NMR (62.5 MHz, $CDCl_3$) δ 172.612(C), 167.016(C), 159.814(C),

129.288(C), 127.412(CH), 114.367(CH), 73.254(CH), 60.614(CH2), 55.360(CH3), 52.814(CH3), 51.555(CH2), 50.943(CH), 28.658(CH2), 25.685(CH2), 24.976(CH2), 14.227(CH3). HRMS (ESI) ($[M+NH_4]^+$) Calcd. for $C_{18}H_{25}O_7S$: 385.1316, Found 385.1326. IR (neat, cm^{-1}):): 1738.97, 1675.99, 1264.64, 1190.13, 1029.01. HPLC analysis: ee (*trans*) = 93%. AD-H(90% hexanes: 10% isopropanol, 1.0 mL/min) *trans*-isomer: t_{major} = 36.0 min, t_{minor} = 20.4 min.

References

(1) Chen, Y.; Zhang, X. P. *J. Org. Chem.* **2007**, *72*, 5931.

(2) Xu, X.; Lu, H. J.; Ruppel, J. V.; Cui, X.; de Mesa, S. L.; Wojtas, L.; Zhang, X. P. *J. Am. Chem. Soc.* **2011**, *133*, 15292.

(3) Chen, Y.; Fields, K. B.; Zhang, X. P. *J. Am. Chem. Soc.* **2004**, *126*, 14718.

(4) Casarini, D.; Lunazzi, L.; Mazzanti, A. *J. Org. Chem.* **2008**, *73*, 2811.

(5) Flynn, C. J.; Elcoate, C. J.; Lawrence, S. E.; Maguire, A. R. *J. Am. Chem. Soc.* **2010**, *132*, 1184.

(6) Blankespoor, R. L.; DeVries, T.; Hansen, E.; Kallemeyn, J. M.; Klooster, A. M.; Mulder, J. A.; Smart, R. P.; Griend, D. A. V. *J. Org. Chem.* **2002**, *67*, 2677.

(7) Milczek, E.; Boudet, N.; Blakey, S. *Angew. Chem., Int. Ed.* **2008**, *47*, 6825.

(8) Ranta, J.; Kumpulainen, T.; Lemmetyinen, H.; Efimov, A. *J. Org. Chem.* **2010**, *75*, 5178.

(9) Houjeiry, T. I.; Poe, S. L.; McQuade, D. T. *Org. Lett.* **2012**, *14*, 4394.

(10) Liang, H.; Ciufolini, M. A. *Chemistry-a European Journal* **2010**, *16*, 13262.

(11) Chen, S. PCT Int. Appl., 2008106204, 04 Sep 2008

(12) Crandall, J. K.; Conover, W. W.; Komin, J. B. *J. Org. Chem.* **1975**, *40*, 2042.

(13) Jung, J. C.; Kache, R.; Vines, K. K.; Zheng, Y. S.; Bijoy, P.; Valluri, M.; Avery, M. A. *J. Org. Chem.* **2004**, *69*, 9269.

(14) Trost, B. M.; Pinkerton, A. B.; Seidel, M. *J. Am. Chem. Soc.* **2001**, *123*, 12466.

X-ray Crystallography

The X-ray diffraction data were collected using Bruker-AXS SMART-APEXII CCD diffractometer (CuKα, λ = 1.54178 Å). Indexing was performed using *APEX2* [1] (Difference Vectors method). Data integration and reduction were performed using SaintPlus 6.01 [2]. Absorption correction was performed by multi-scan method implemented in SADABS [3]. Space groups were determined using XPREP implemented in APEX2 [1]. The structure was solved using SHELXS-97 (direct methods) and refined using SHELXL-97 (full-matrix least-squares on F^2) contained in APEX2 [1] and WinGX v1.70.01 [4,5,6,7] programs packages. All non-hydrogen atoms were refined anisotropically. Hydrogen atoms were placed in geometrically calculated positions or found in the Fourier difference map and included in the refinement process using riding model with isotropic thermal parameters: Uiso(H) = 1.5Ueq(-CH3), Uiso(H) = 1.2Ueq(-CH2,-CH) or without constraints (H1A and H1B in **L1**). For **L1** the absolute configuration has been established by the structure determination of a compound containing a chiral reference molecule of known absolute configuration (Chen, Y.; Zhang, X. P. *J. Org. Chem.* **2007**, *72*, 5931.). Crystal data and refinement conditions are shown in Table 1, 2 and 3.

[1] Bruker (2008). *APEX2* (Version 2008.1-0). Bruker AXS Inc., Madison, Wisconsin, USA.
[2] Bruker (2001b). SAINT-V6.28A. Data Reduction Software.
[3] Sheldrick, G. M. (1996). *SADABS. Program for Empirical Absorption Correction.* University of Gottingen, Germany.
[4] Farrugia L.J. Appl. Cryst. (1999). 32, 837±838
[5] Sheldrick, G.M. (1997) SHELXL-97. Program for the Refinement of Crystal
[6] Sheldrick, G.M. (1990) Acta Cryst. A46, 467-473
[7] Sheldrick, G. M. (2008). Acta Cryst. A64, 112-122.

Table 1. Crystal data and structure refinement for compound **L1**	
Identification code	**L1**
Empirical formula	C14 H19 N O
Formula weight	217.30
Temperature	228(2) K
Wavelength	1.54178 A
Crystal system, space group	Monoclinic, C2
Unit cell dimensions	a = 7.0592(2) A alpha = 90 deg.<br>b = 9.6718(3) A beta = 91.179(2) deg.<br>c = 18.5618(6) A gamma = 90 deg.
Volume	1267.04(7) A^3
Z, Calculated density	4, 1.139 Mg/m^3
Absorption coefficient	0.552 mm^-1
F(000)	472
Crystal size	0.20 x 0.02 x 0.02 mm
Theta range for data collection	4.77 to 65.96 deg.
Limiting indices	-8<=h<=8, -10<=k<=8, -21<=l<=21
Reflections collected / unique	6867 / 1913 [R(int) = 0.0270]
Completeness to theta = 65.96	98.3 %
Absorption correction	Semi-empirical from equivalents
Max. and min. transmission	0.9918 and 0.8976
Refinement method	Full-matrix least-squares on F^2
Data / restraints / parameters	1913 / 1 / 156
Goodness-of-fit on F^2	1.082
Final R indices [I>2sigma(I)]	R1 = 0.0383, wR2 = 0.1005
R indices (all data)	R1 = 0.0408, wR2 = 0.1025
Absolute structure parameter	-0.4(3)
Largest diff. peak and hole	0.123 and -0.160 e.A^-3

Table 2. Crystal data and structure refinement for compound **2g**	
Identification code	**2g**
Empirical formula	C12 H14 O4 S
Formula weight	254.29
Temperature	203(2) K
Wavelength	1.54178 A
Crystal system, space group	Monoclinic, P21
Unit cell dimensions	a = 6.70370(10) A alpha = 90 deg.<br>b = 10.0938(2) A,beta = 108.1320(10) deg.<br>c = 9.5452(2) A gamma = 90 deg.
Volume	613.81(2) A^3
Z, Calculated density	2, 1.376 Mg/m^3
Absorption coefficient	2.370 mm^-1
F(000)	268
Crystal size	0.20 x 0.20 x 0.20 mm
Theta range for data collection	4.87 to 68.16 deg.
Limiting indices	-7<=h<=8, -12<=k<=12, -11<=l<=10
Reflections collected / unique	6686 / 2144 [R(int) = 0.0335]
Completeness to theta = 68.16	98.4 %
Absorption correction	Semi-empirical from equivalents
Max. and min. transmission	0.6486 and 0.6486
Refinement method	Full-matrix least-squares on F^2
Data / restraints / parameters	2144 / 1 / 156
Goodness-of-fit on F^2	1.062
Final R indices [I>2sigma(I)]	R1 = 0.0324, wR2 = 0.0785
R indices (all data)	R1 = 0.0341, wR2 = 0.0796
Absolute structure parameter	0.021(19)
Extinction coefficient	0.0073(11)
Largest diff. peak and hole	0.218 and -0.205 e.A^-3

Table 3. Crystal data and structure refinement for compound **3ea**	
Identification code	**3ea**
Empirical formula	C12 H11 Cl2 F O4 S
Formula weight	341.17
Temperature	228(2) K
Wavelength	1.54178 A
Crystal system, space group	Orthorhombic, P212121
Unit cell dimensions	a = 7.7539(3) A alpha = 90 deg.<br>b = 8.4364(3) A beta = 90 deg.<br>c = 22.2865(6) A gamma = 90 deg.
Volume	1457.87(9) A^3
Z, Calculated density	4, 1.554 Mg/m^3
Absorption coefficient	5.556 mm^-1
F(000)	696
Crystal size	0.15 x 0.08 x 0.02 mm
Theta range for data collection	3.97 to 68.23 deg.
Limiting indices	-9<=h<=9, -9<=k<=10, -26<=l<=26
Reflections collected / unique	17397 / 2634 [R(int) = 0.0770]
Completeness to theta = 68.23	99.0 %
Absorption correction	Semi-empirical from equivalents
Max. and min. transmission	0.8970 and 0.4895
Refinement method	Full-matrix least-squares on F^2
Data / restraints / parameters	2634 / 0 / 183
Goodness-of-fit on F^2	1.052
Final R indices [I>2sigma(I)]	R1 = 0.0417, wR2 = 0.0979
R indices (all data)	R1 = 0.0507, wR2 = 0.1036
Absolute structure parameter	0.00(2)
Extinction coefficient	0.0018(3)
Largest diff. peak and hole	0.322 and -0.300 e.A^-3

Supporting Information

Stereoselective Radical C–H Alkylation with Acceptor/Acceptor-Substituted Diazo Reagents via Co(II)-Based Metalloradical Catalysis

Xin Cui, Xue Xu, Li-Mei Jin, Lukasz Wojtas, and X. Peter Zhang*

Department of Chemistry, University of South Florida, Tampa, Florida 33620-5250

2-(4-(tert-butyl)phenyl)cyclopropanecarboxamide (L1)

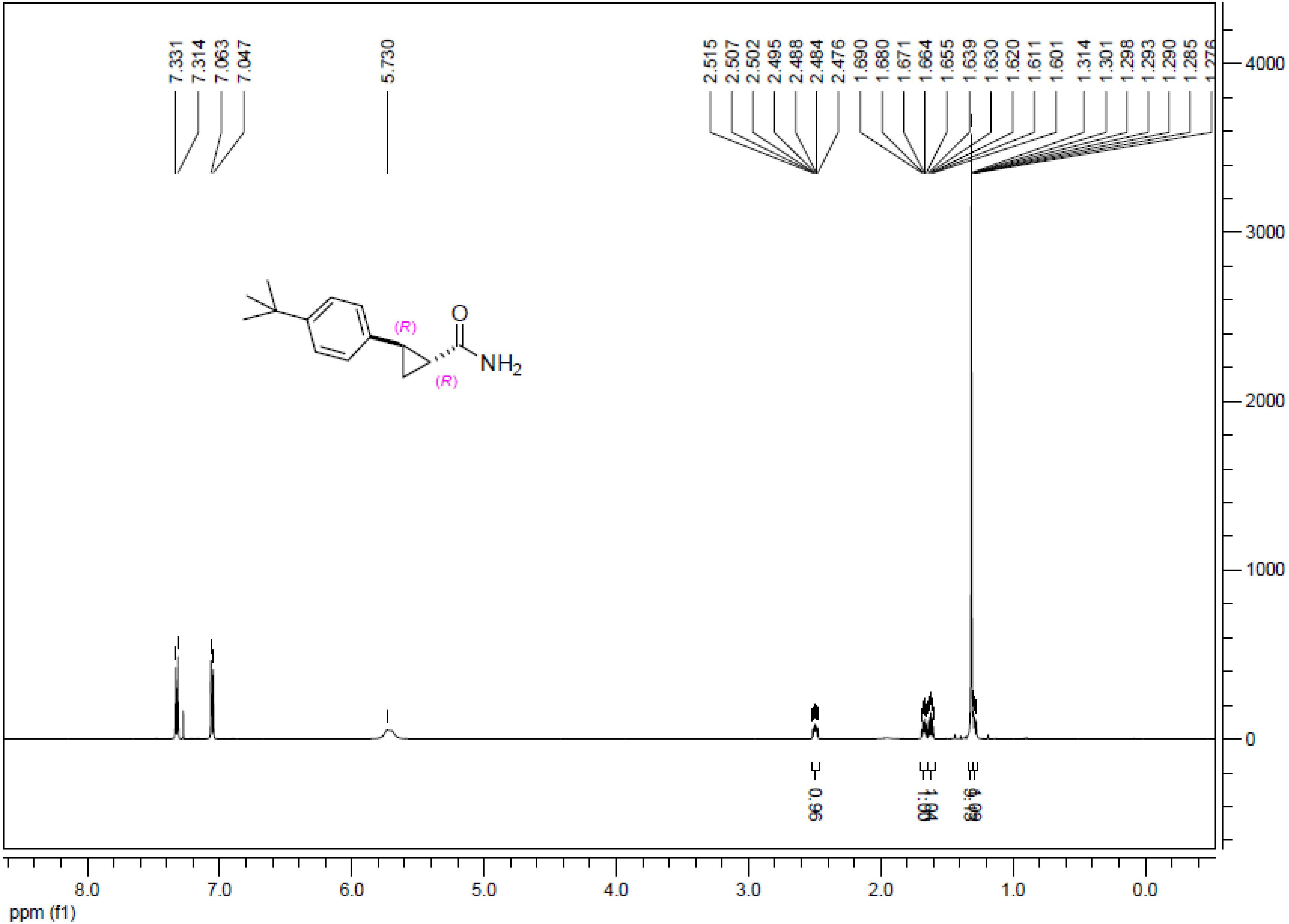

2-(4-(tert-butyl)phenyl)cyclopropanecarboxamide (L1)

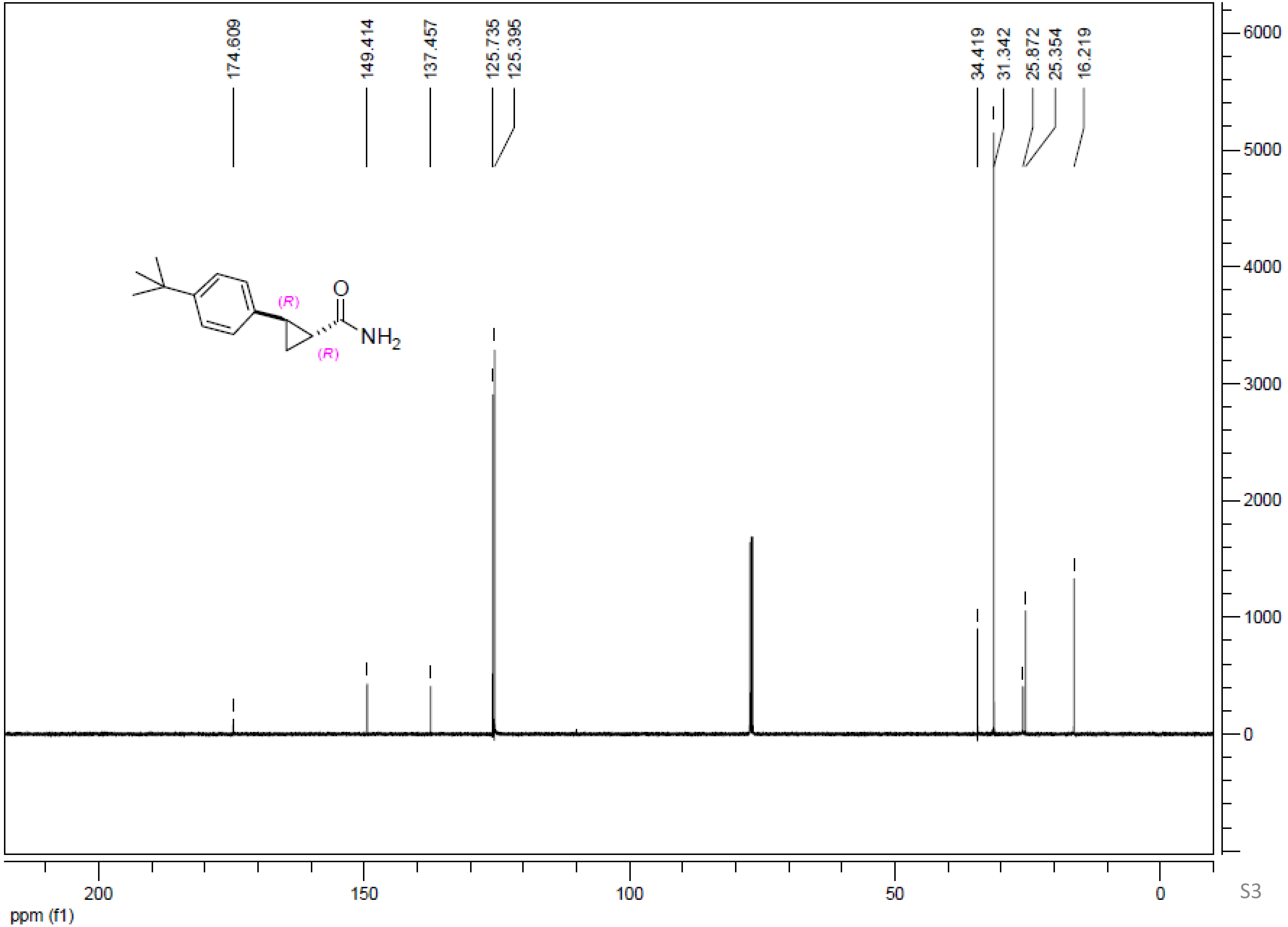

2-(4-(tert-butyl)phenyl)cyclopropanecarboxamide (L1)

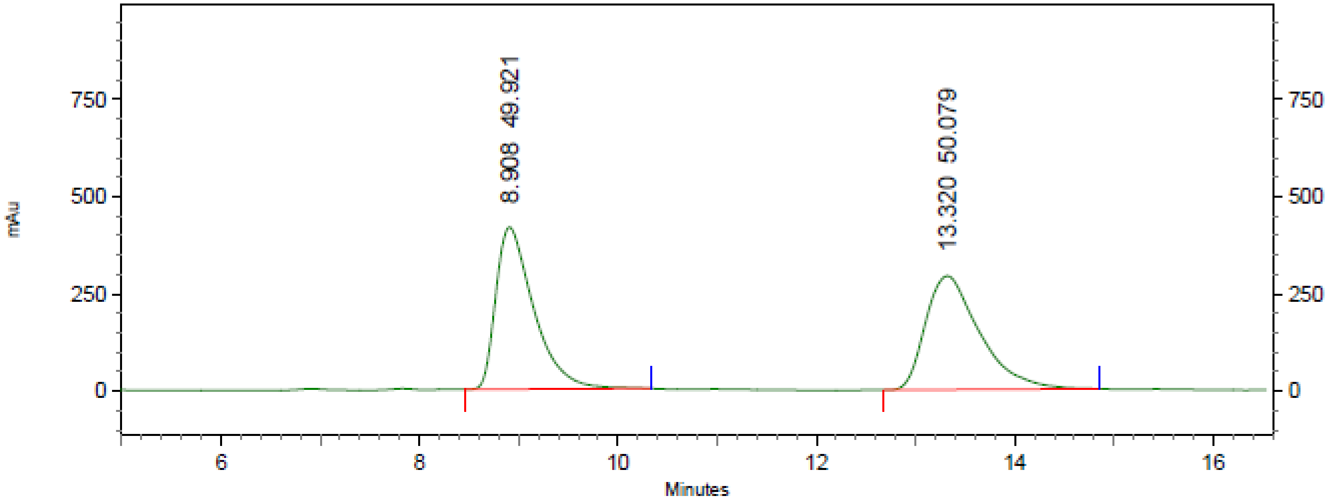

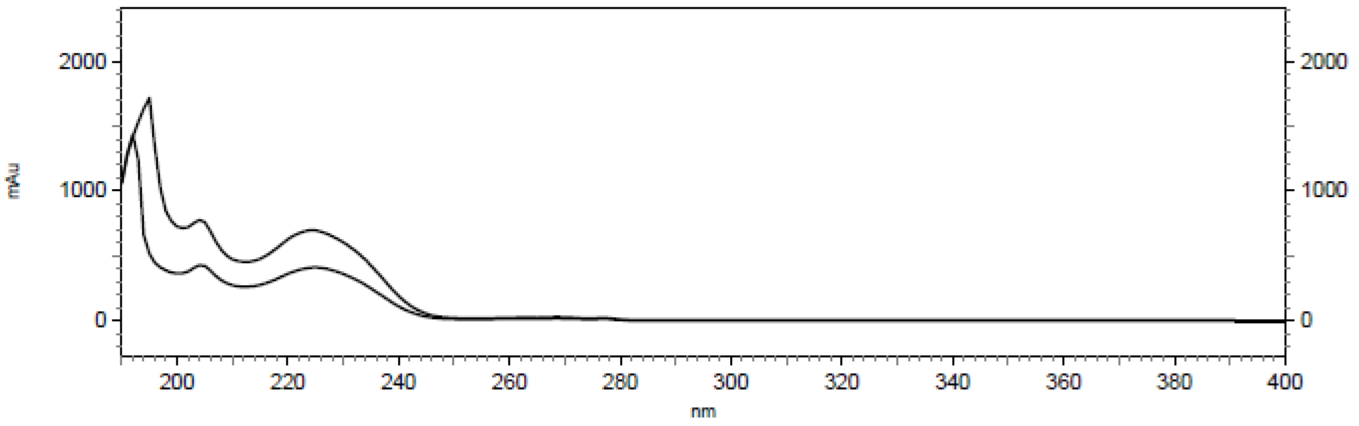

13: 214 nm, 4 nm Results

Pk #	Retention Time	Area Percent
1	8.908	49.921
2	13.320	50.079
Totals		100.000

2-(4-(tert-butyl)phenyl)cyclopropanecarboxamide (L1)

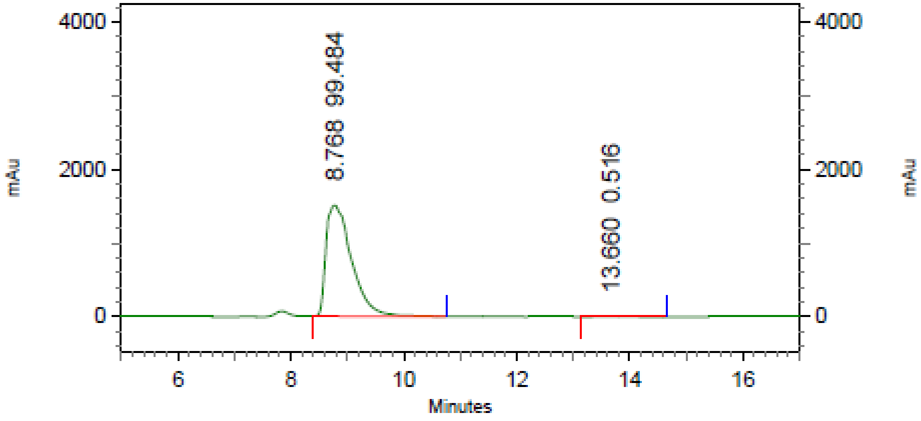

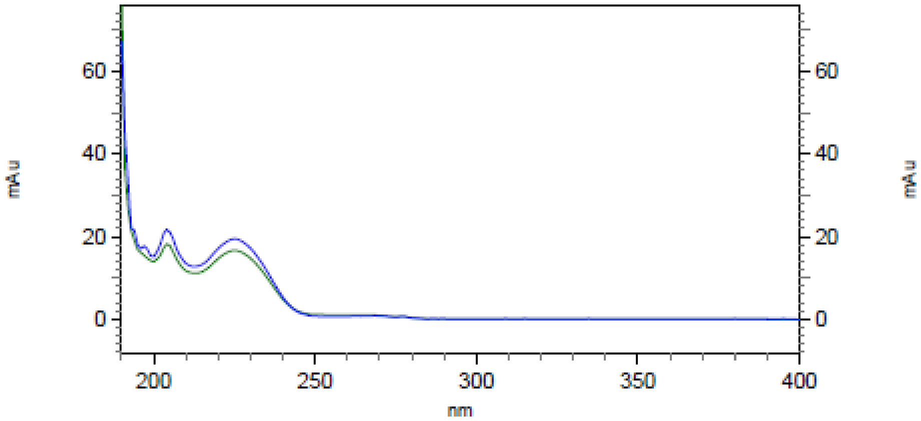

4: 223 nm, 4 nm

Results

Name	Retention Time	Area Percent	Pk #
	8.768	99.484	1
	13.660	0.516	2
Totals		100.000	

[H_2(**P2**)] 3,5-Di^{t}Bu-(4'-^{t}Bu)XuPhyrin

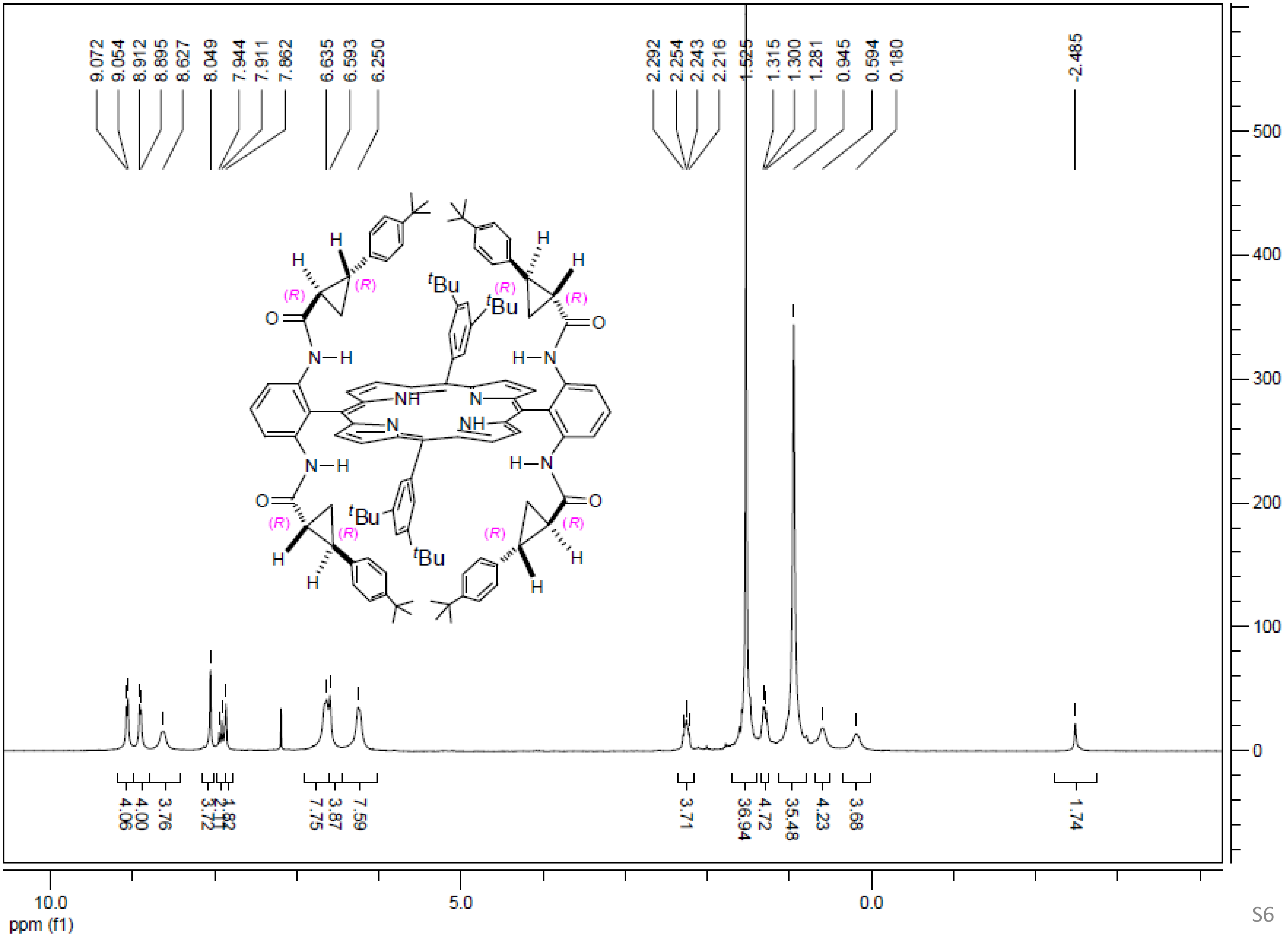

[H_2(**P2**)] 3,5-Di^{t}Bu-(4'-^{t}Bu)XuPhyrin

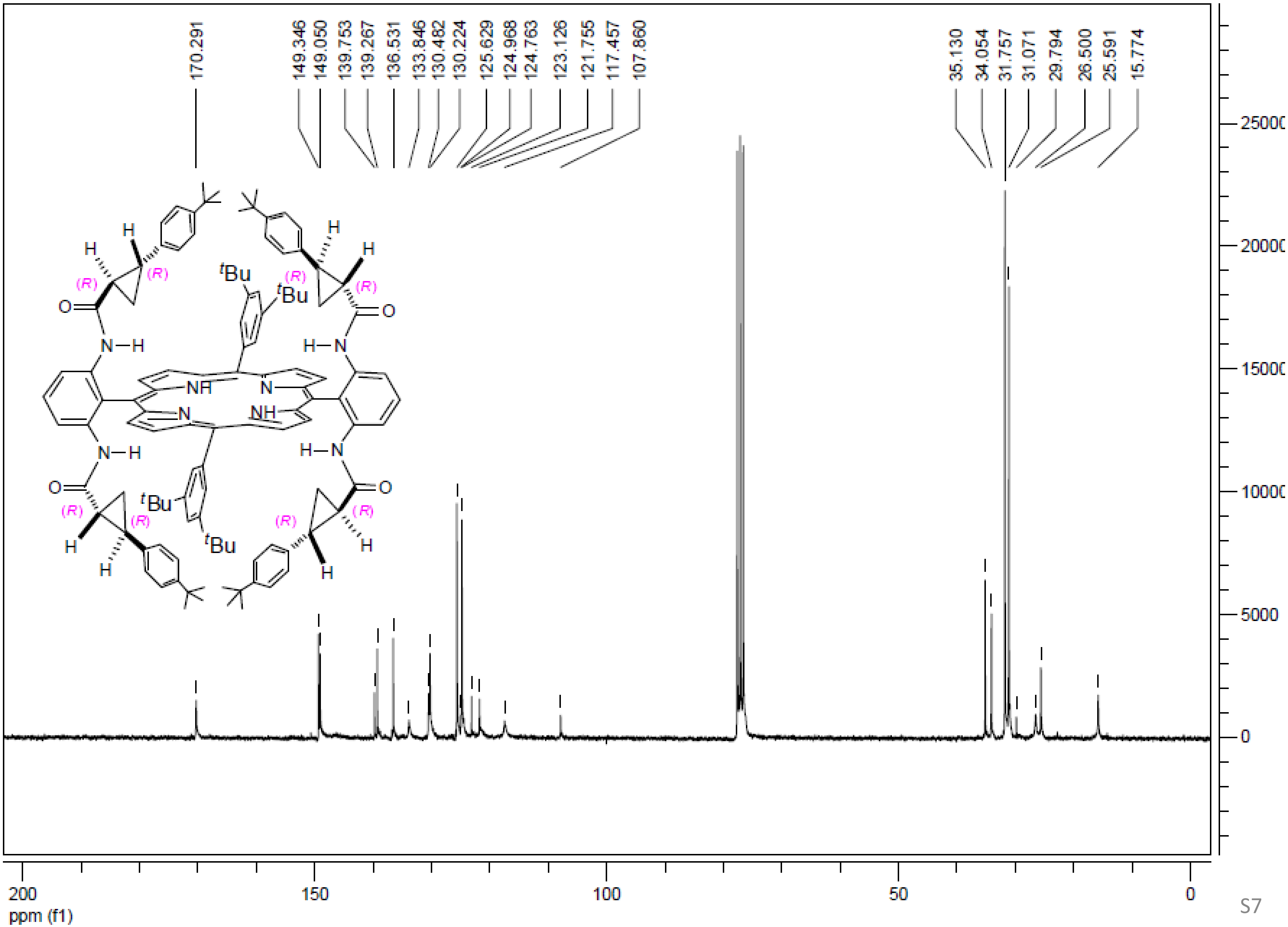

5,15-Bis(2,6-dibromophenyl)-10,20-bis(3,5-diisopropylphenyl)porphyrin

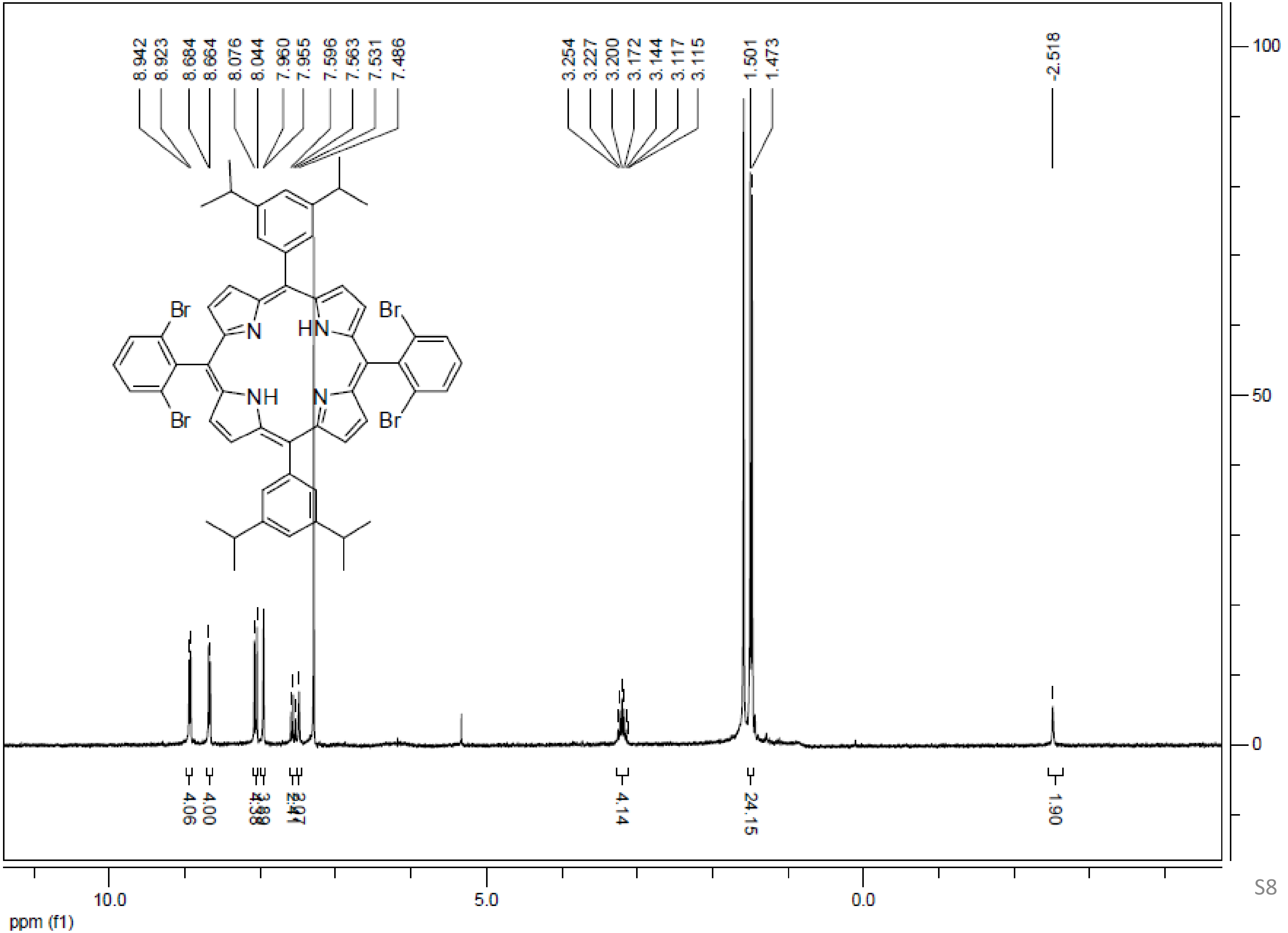

5,15-Bis(2,6-dibromophenyl)-10,20-bis(3,5-diisopropylphenyl)porphyrin

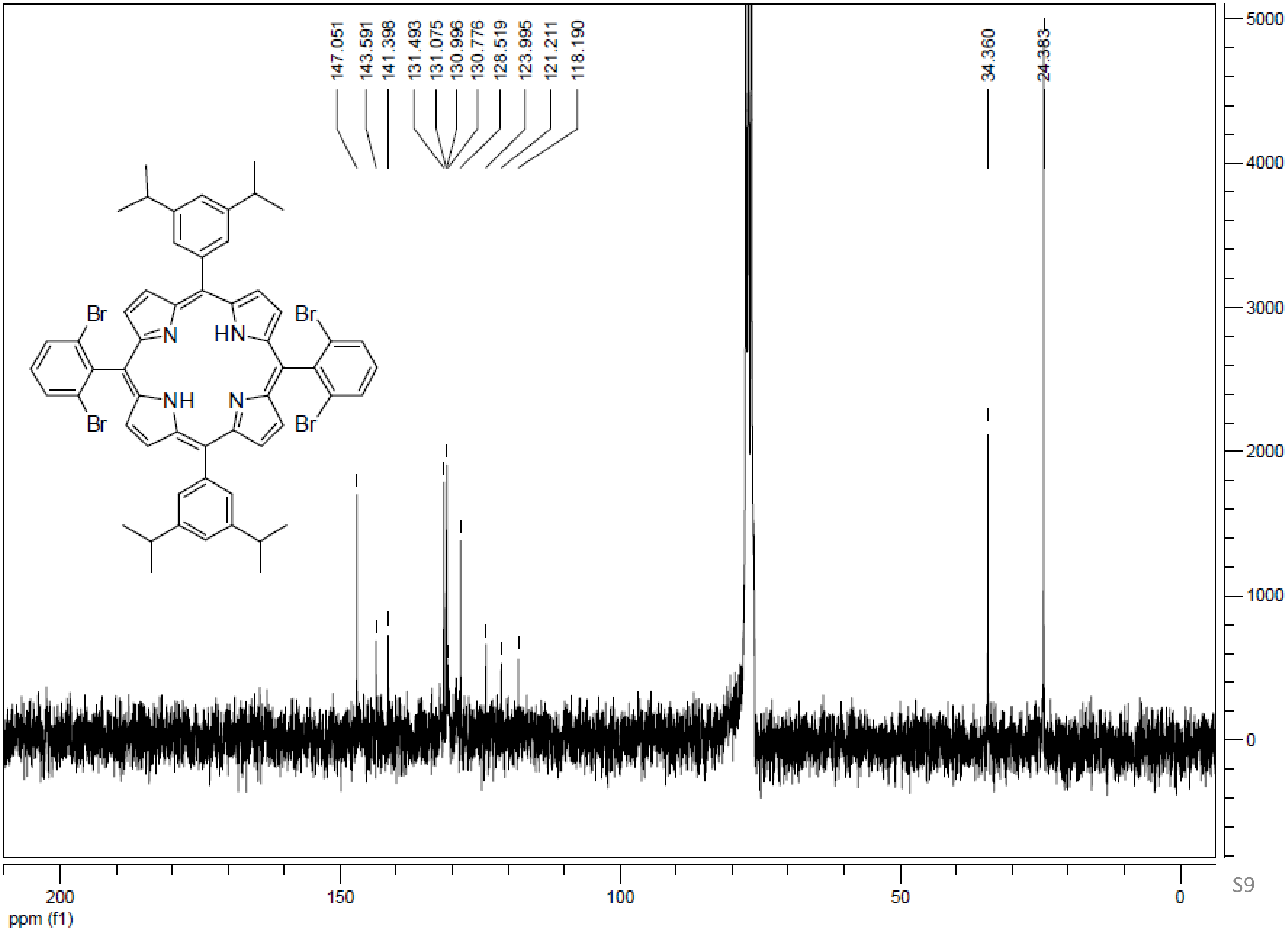

[H_2(**P3**)] *3,5*-Di*i*Pr-(4'-^{t}Bu)XuPhyrin

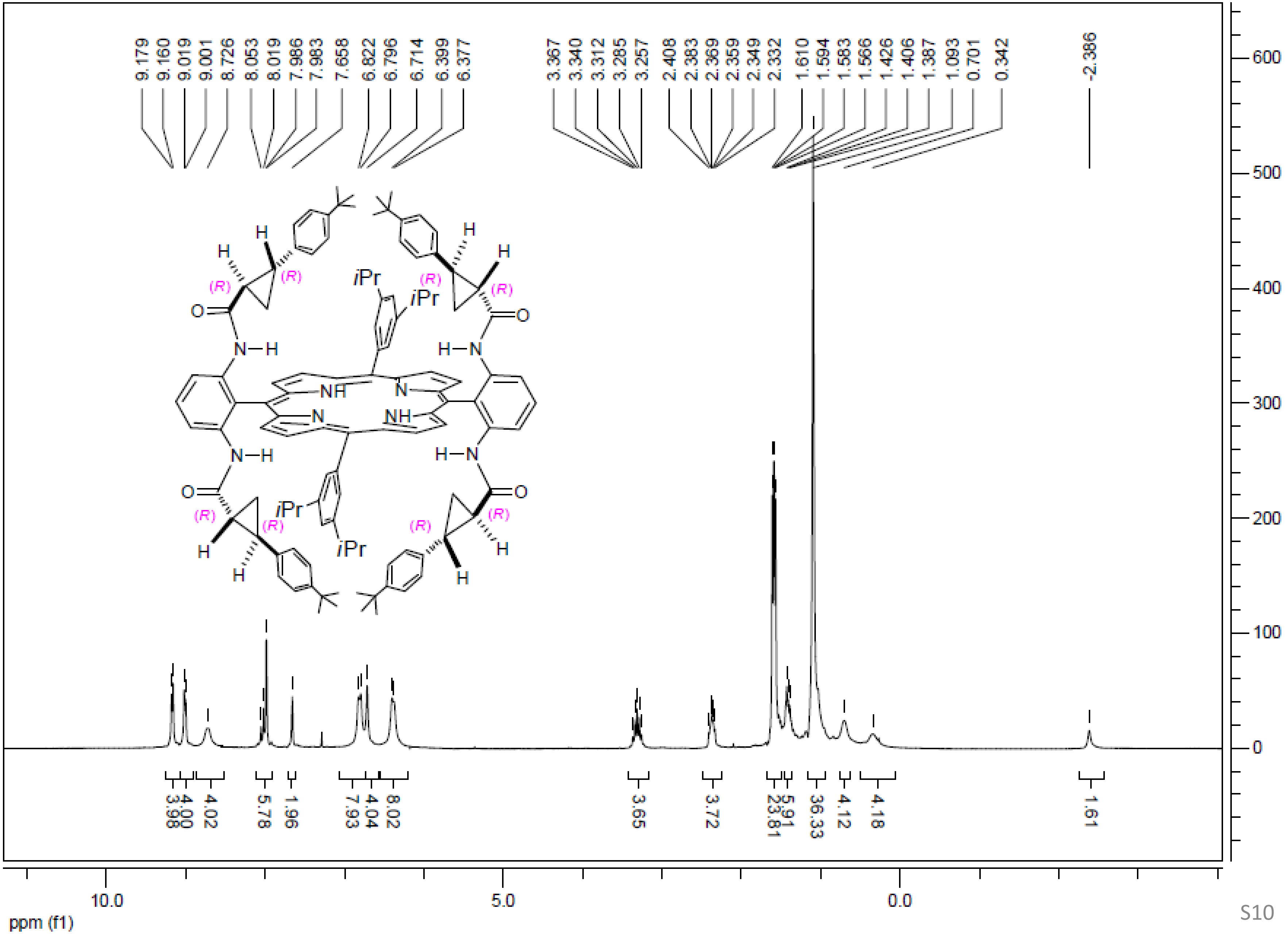

[H_2(**P3**)] *3,5*-Di*i*Pr-(4'-^{t}Bu)XuPhyrin

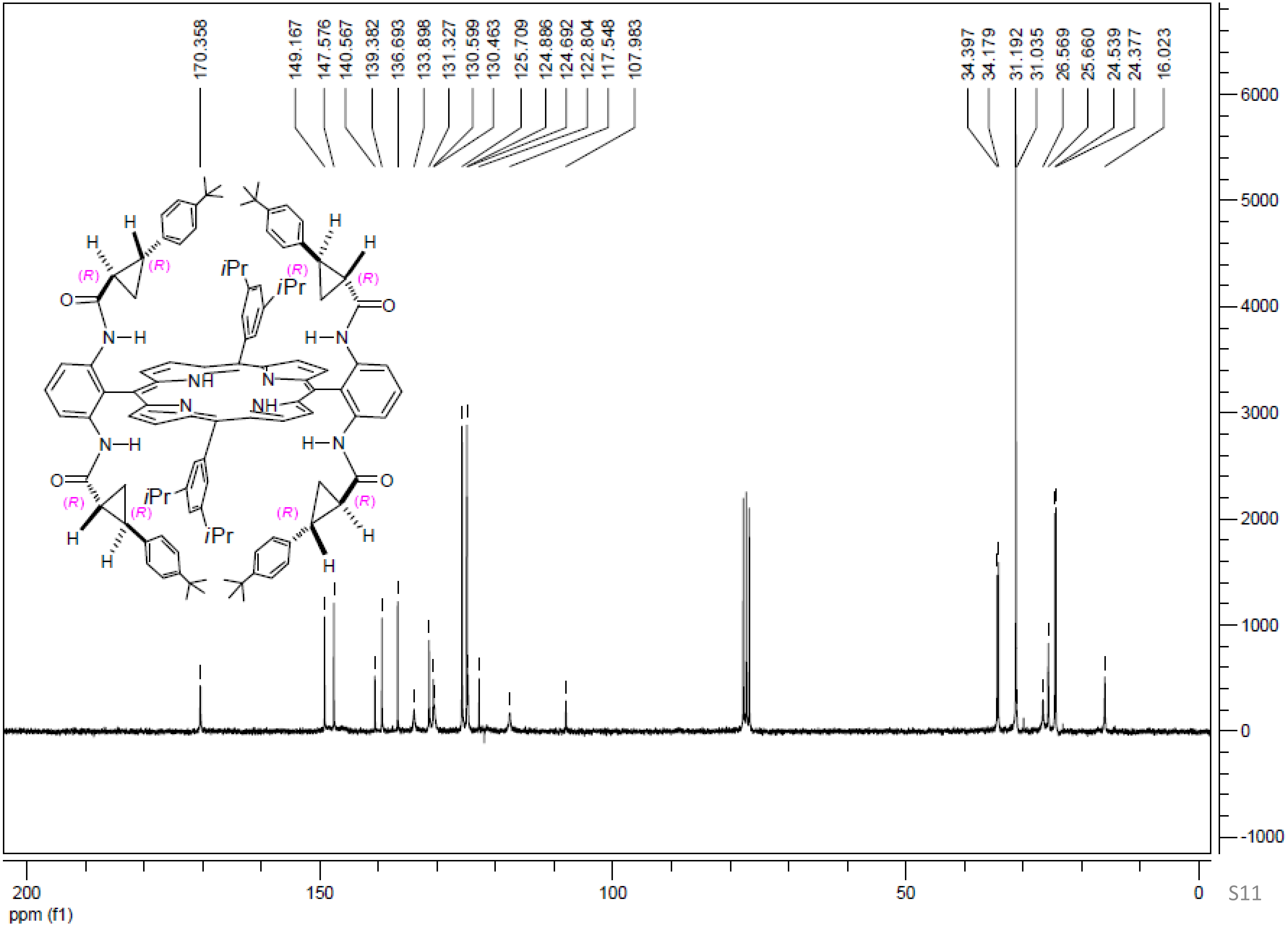

methyl 2-diazo-2-((3-(4-nitrophenyl)propyl)sulfonyl)acetate (**1a**)

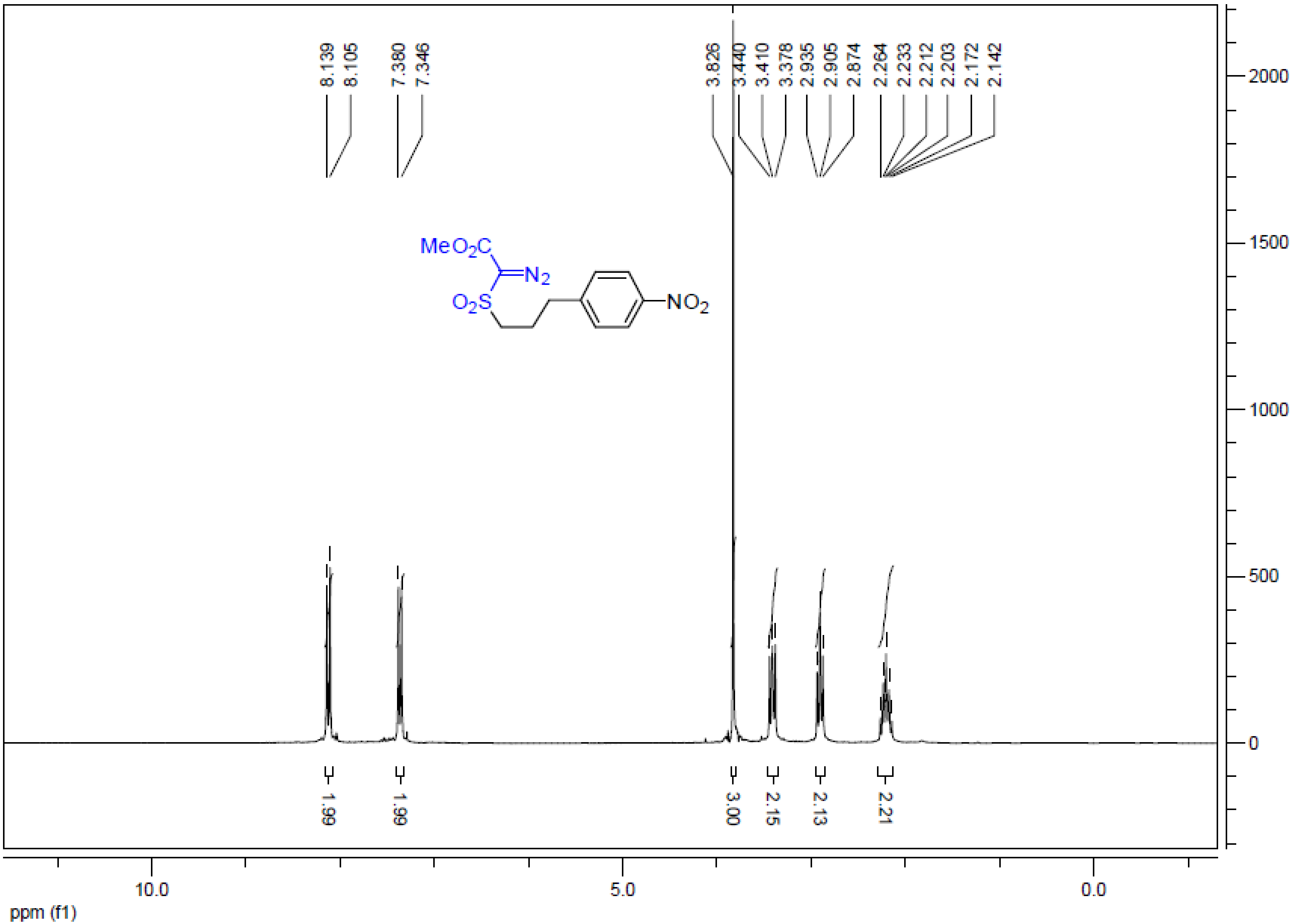

methyl 2-diazo-2-((3-(4-nitrophenyl)propyl)sulfonyl)acetate (**1a**)

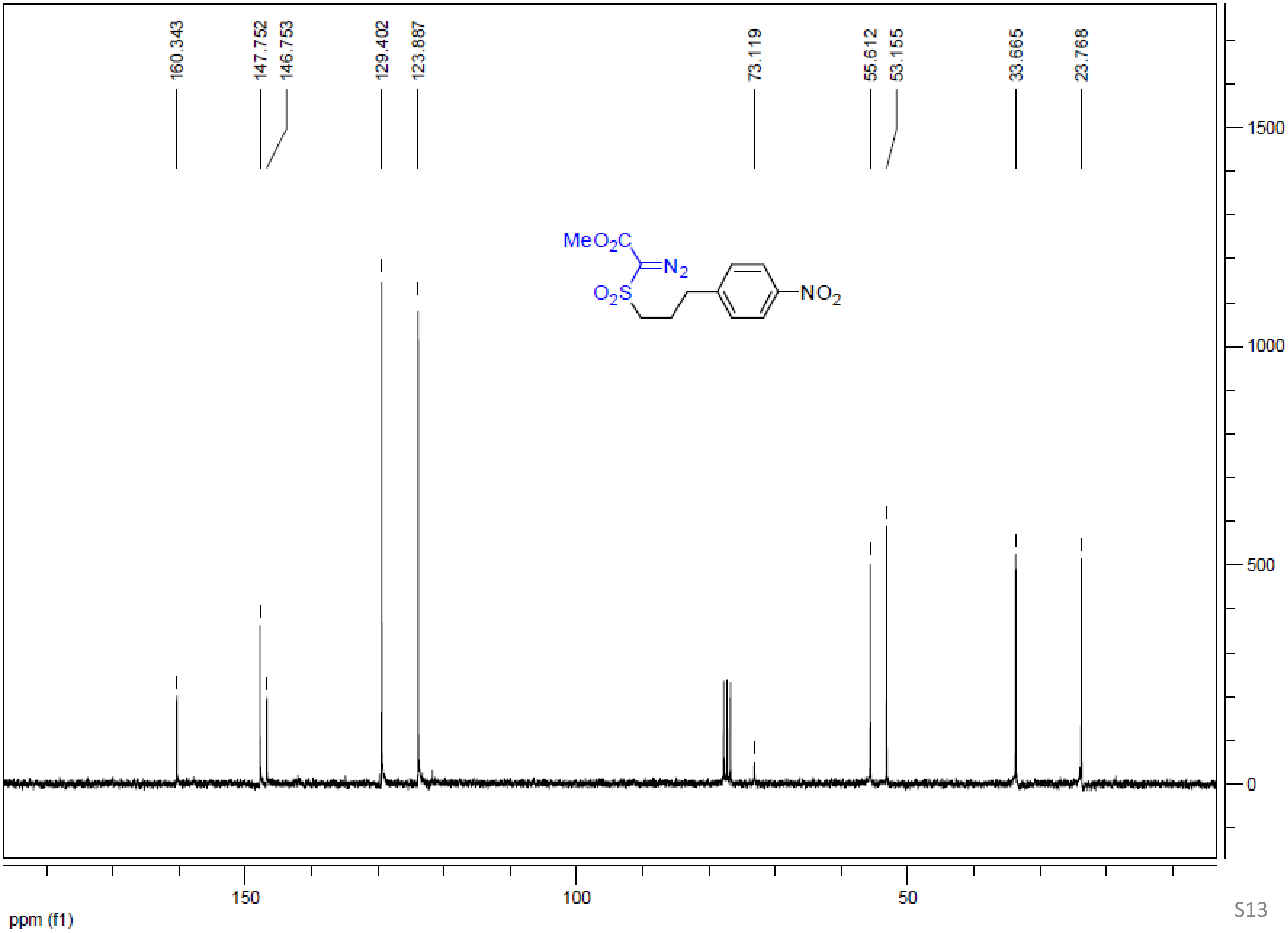

methyl 2-diazo-2-((3-(4-(trifluoromethyl)phenyl)propyl)sulfonyl)acetate (**1b**)

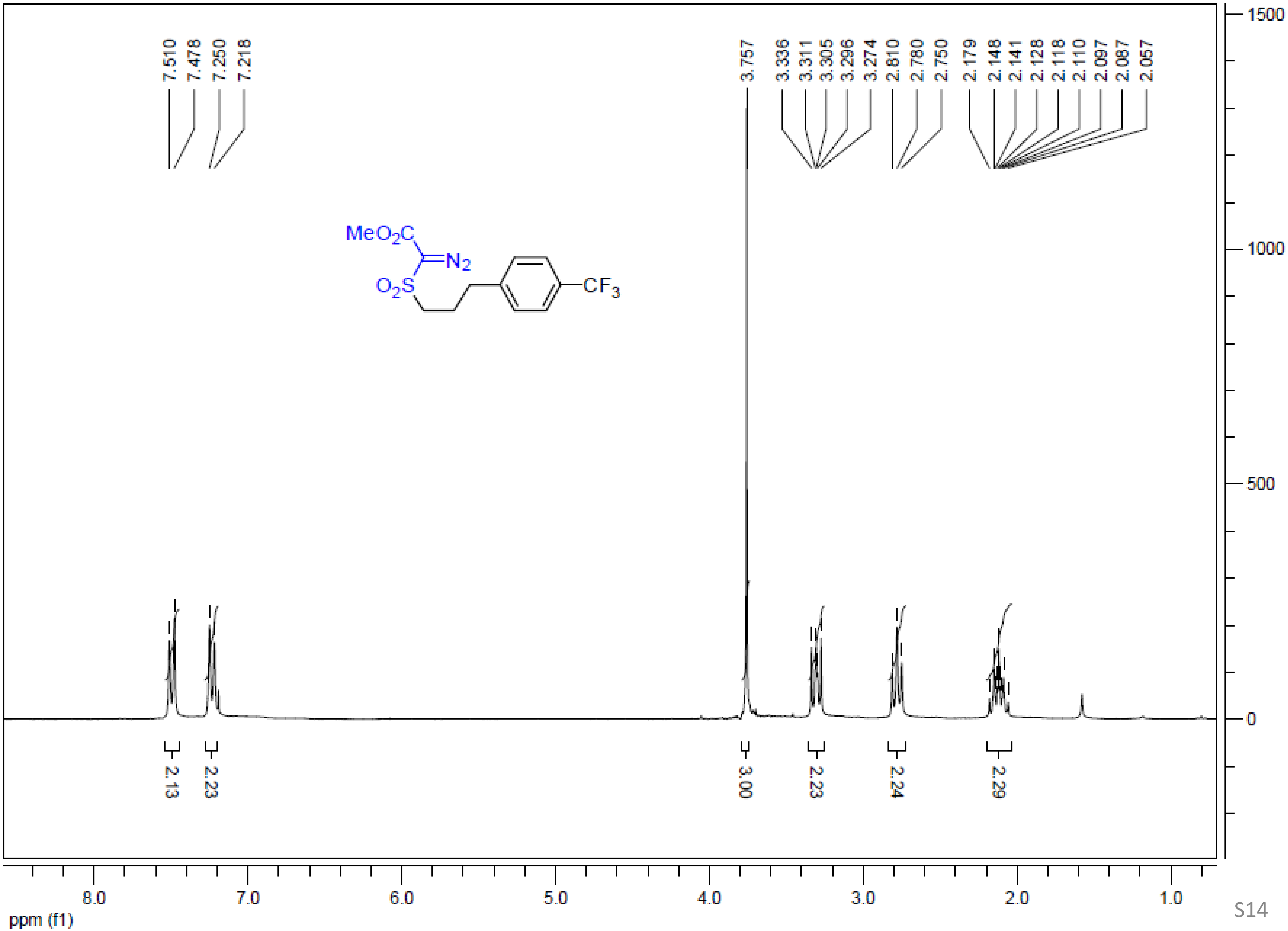

methyl 2-diazo-2-((3-(4-(trifluoromethyl)phenyl)propyl)sulfonyl)acetate (**1b**)

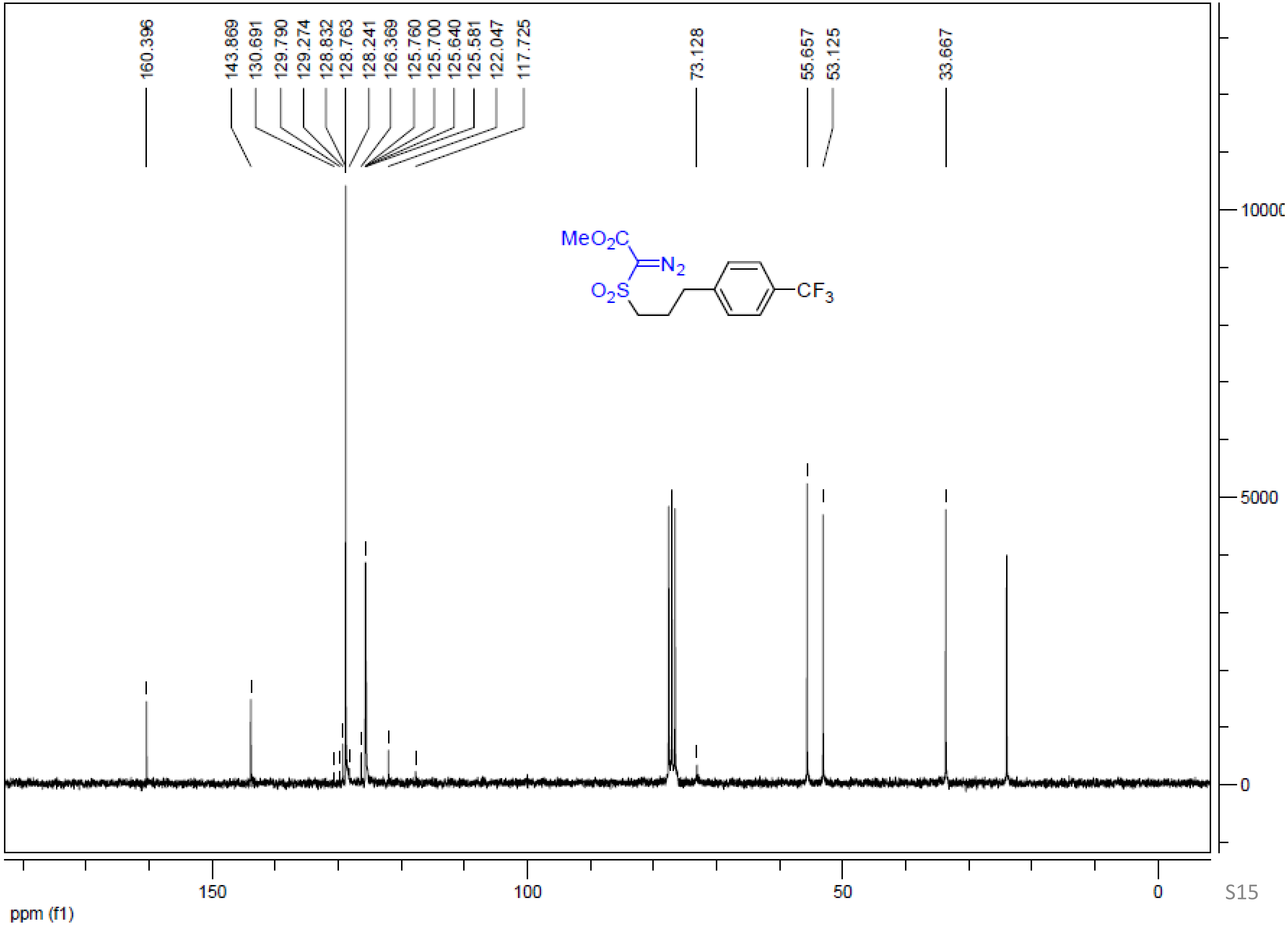

methyl 2-diazo-2-((3-(4-fluorophenyl)propyl)sulfonyl)acetate (**1c**)

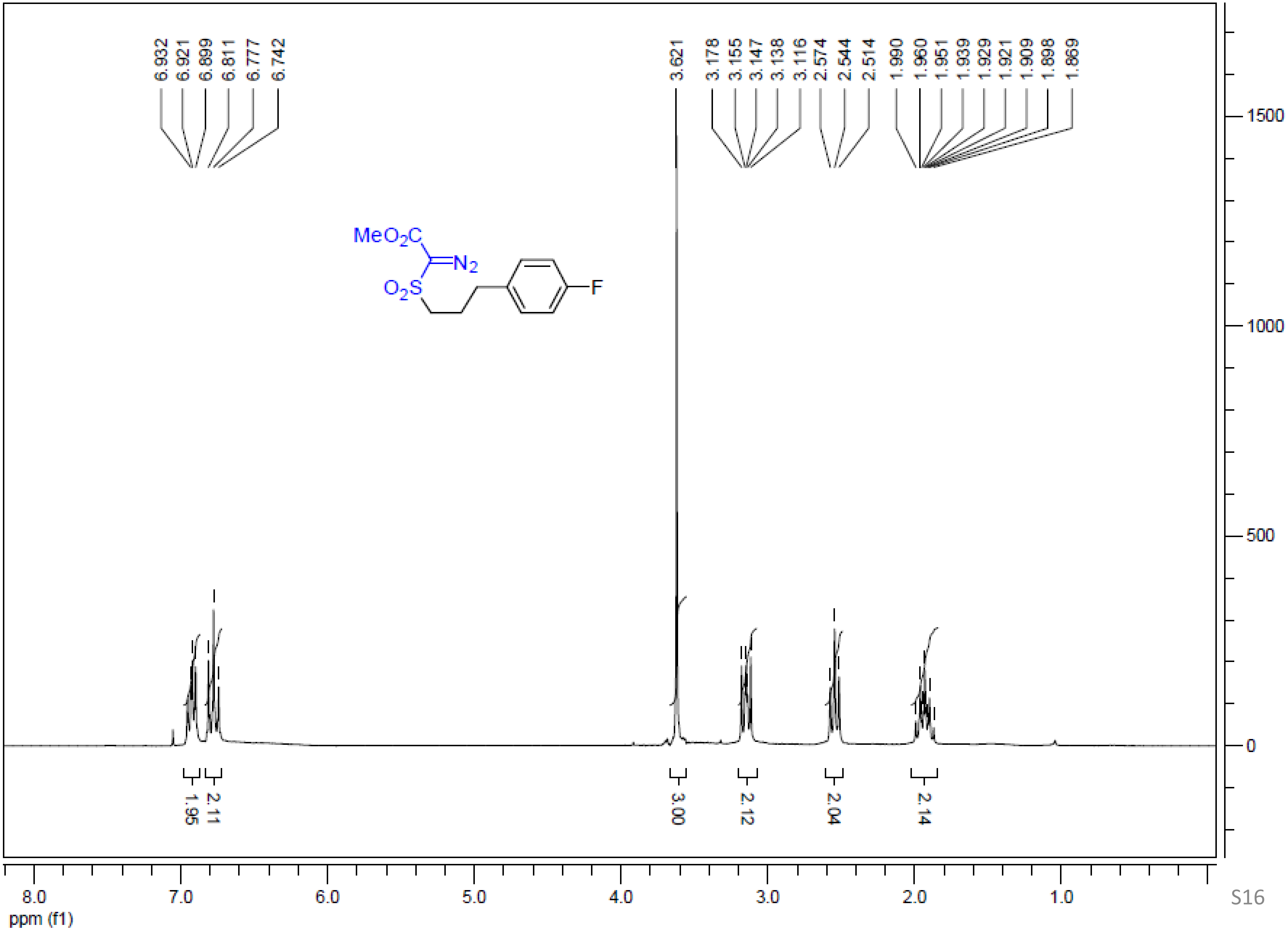

methyl 2-diazo-2-((3-(4-fluorophenyl)propyl)sulfonyl)acetate (**1c**)

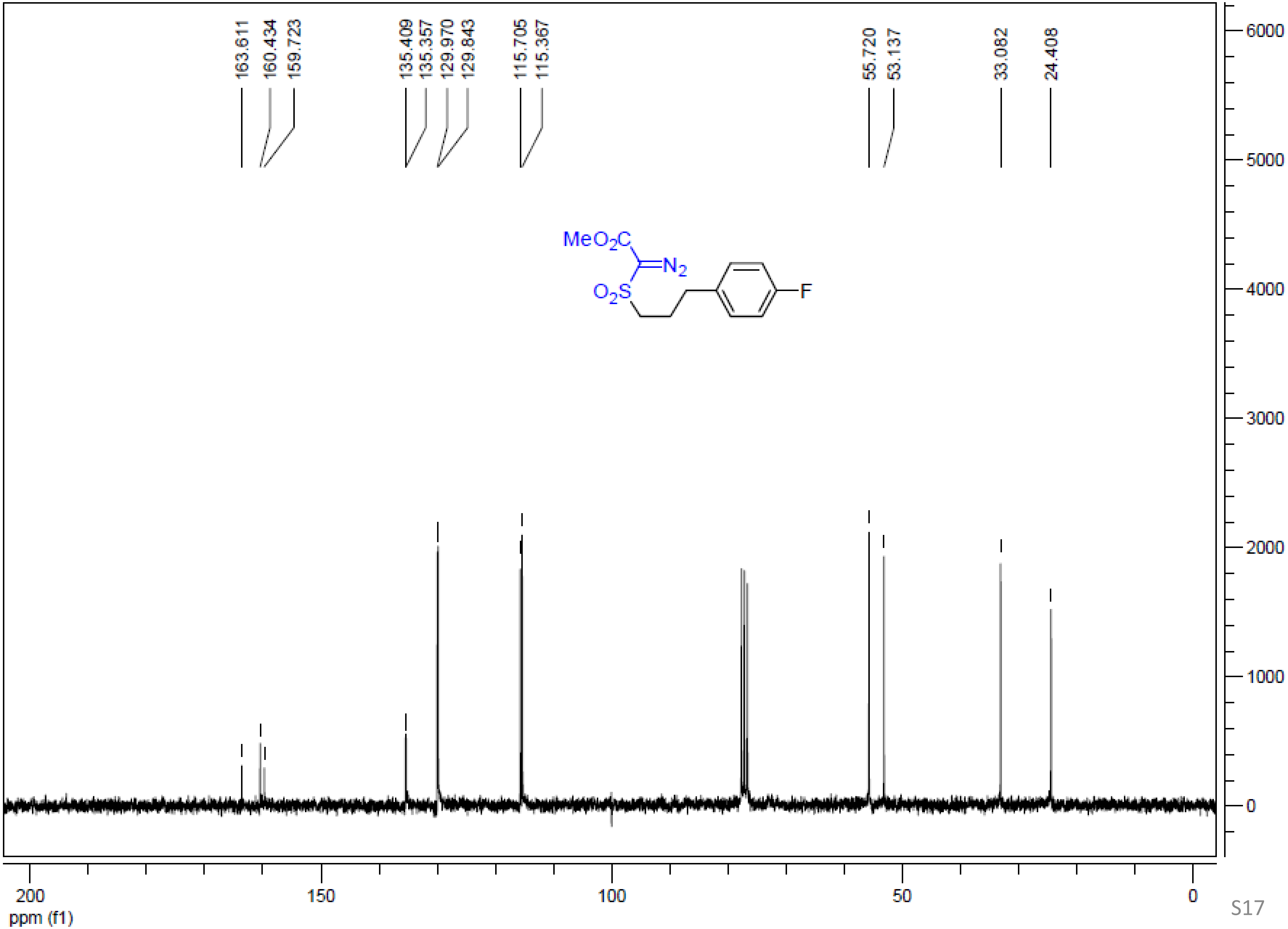

methyl 2-((3-(4-chlorophenyl)propyl)sulfonyl)-2-diazoacetate (**1d**)

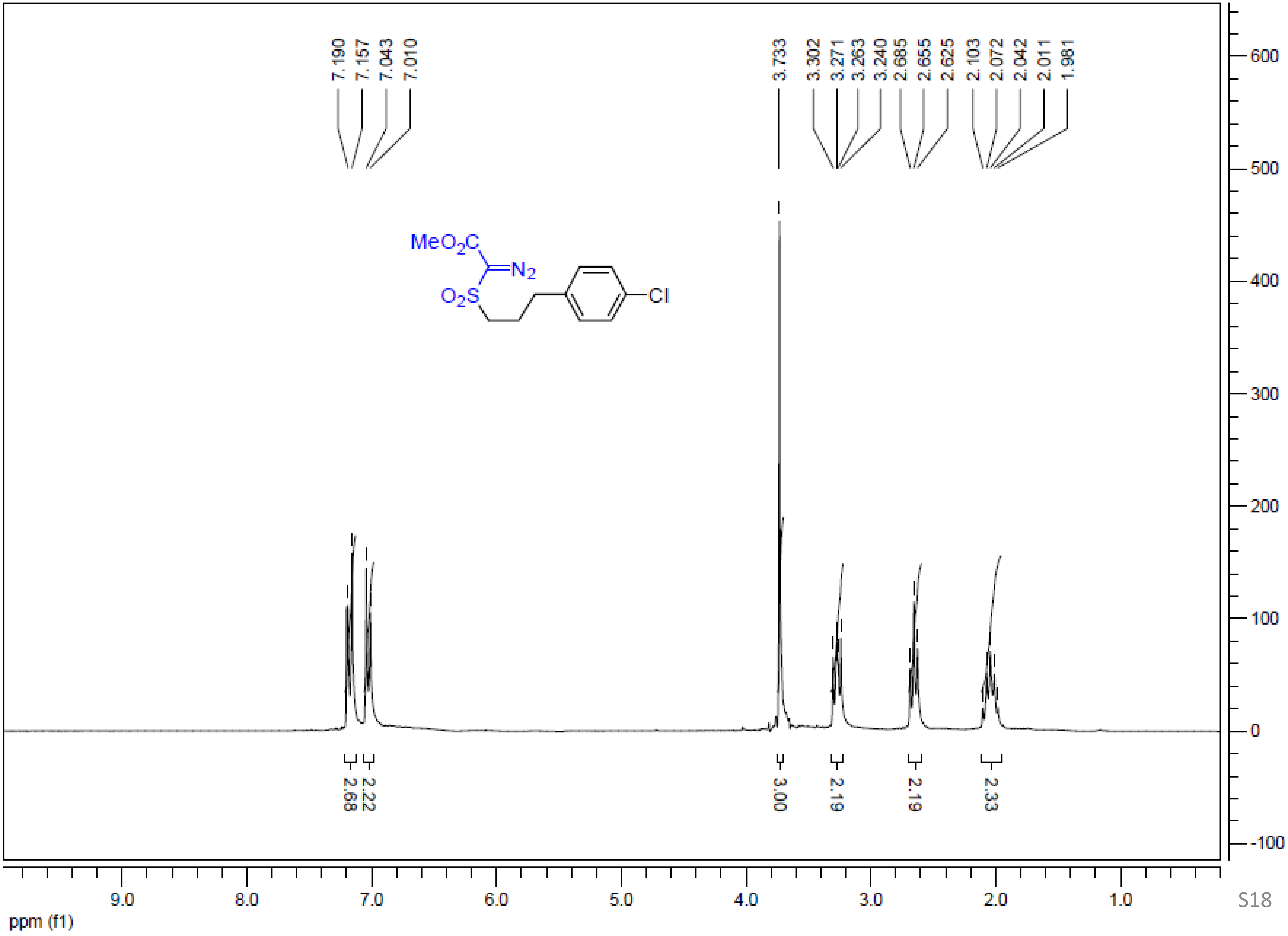

methyl 2-((3-(4-chlorophenyl)propyl)sulfonyl)-2-diazoacetate (**1d**)

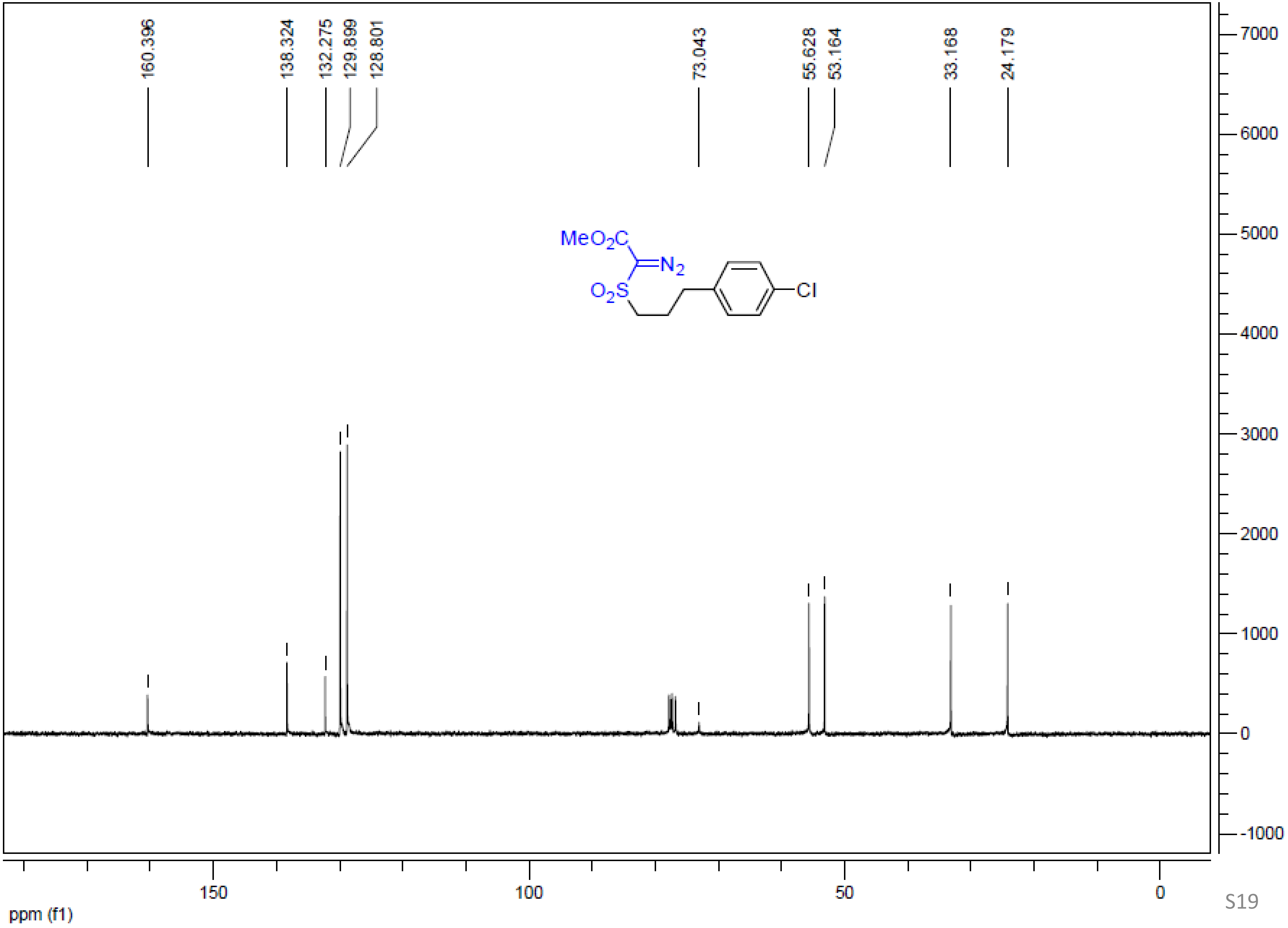

methyl 2-diazo-2-((3-(3,5-dichlorophenyl)propyl)sulfonyl)acetate (**1e**)

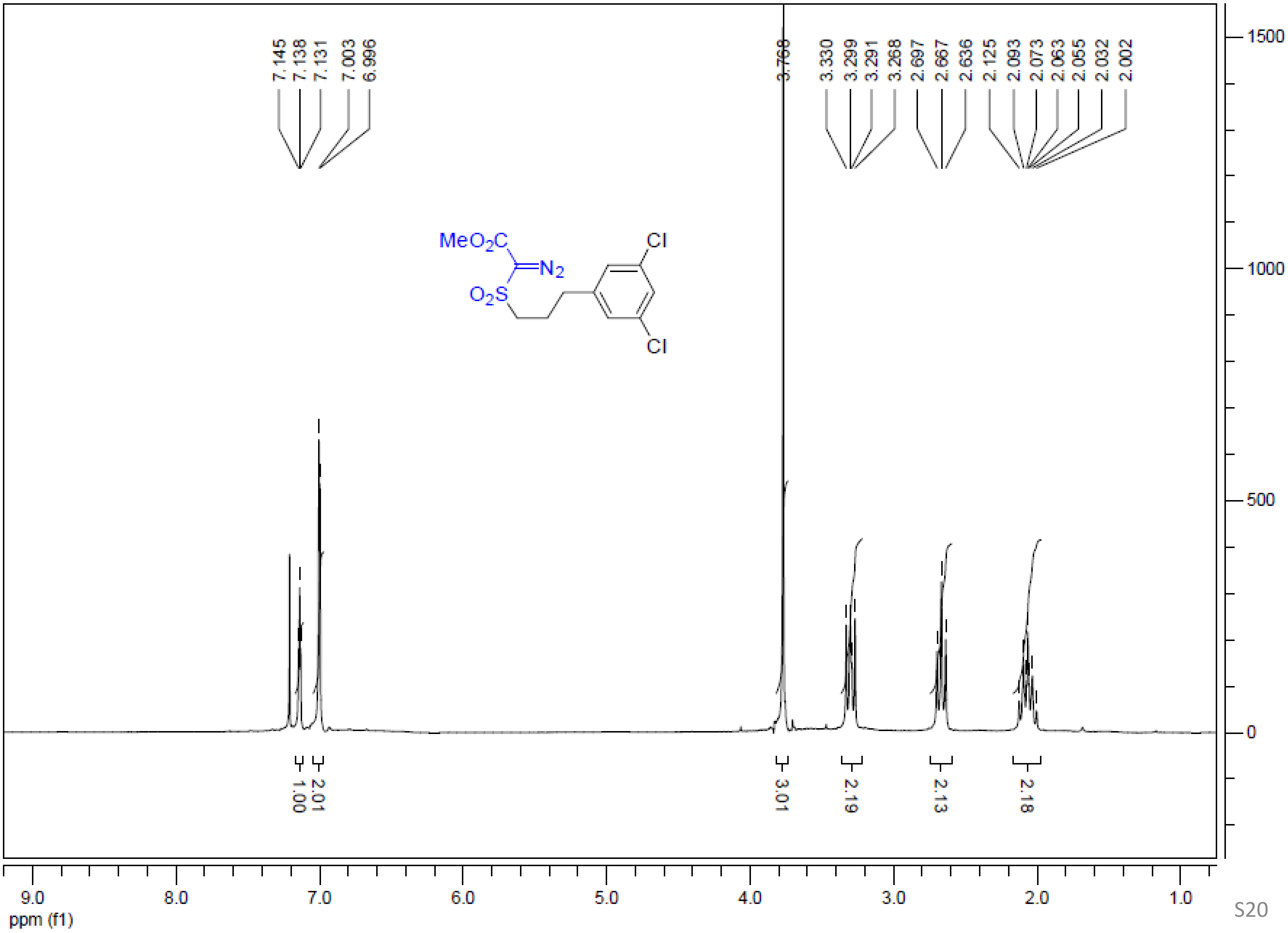

methyl 2-diazo-2-((3-(3,5-dichlorophenyl)propyl)sulfonyl)acetate (**1e**)

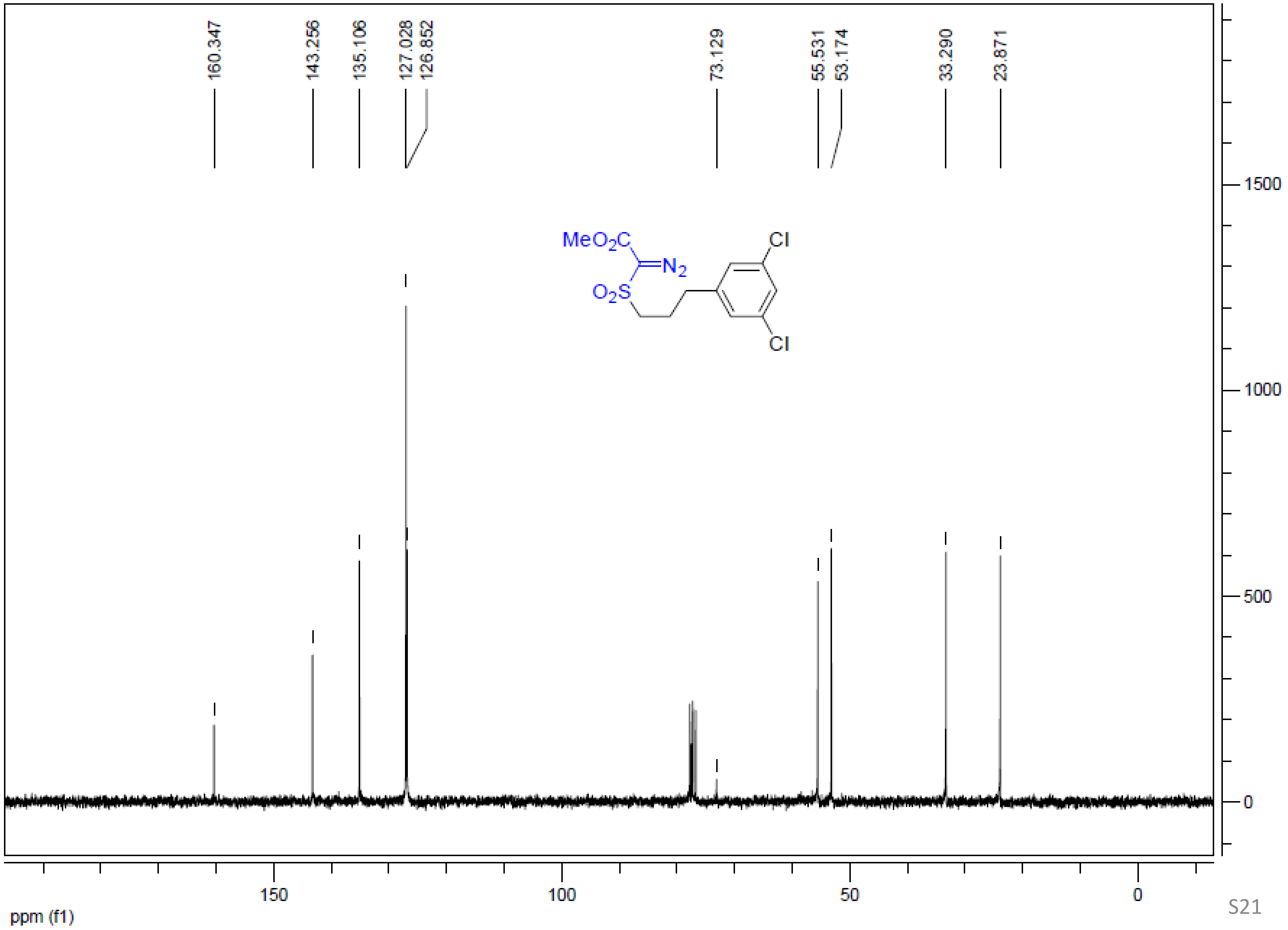

methyl 2-((3-(3-bromophenyl)propyl)sulfonyl)-2-diazoacetate (**1f**)

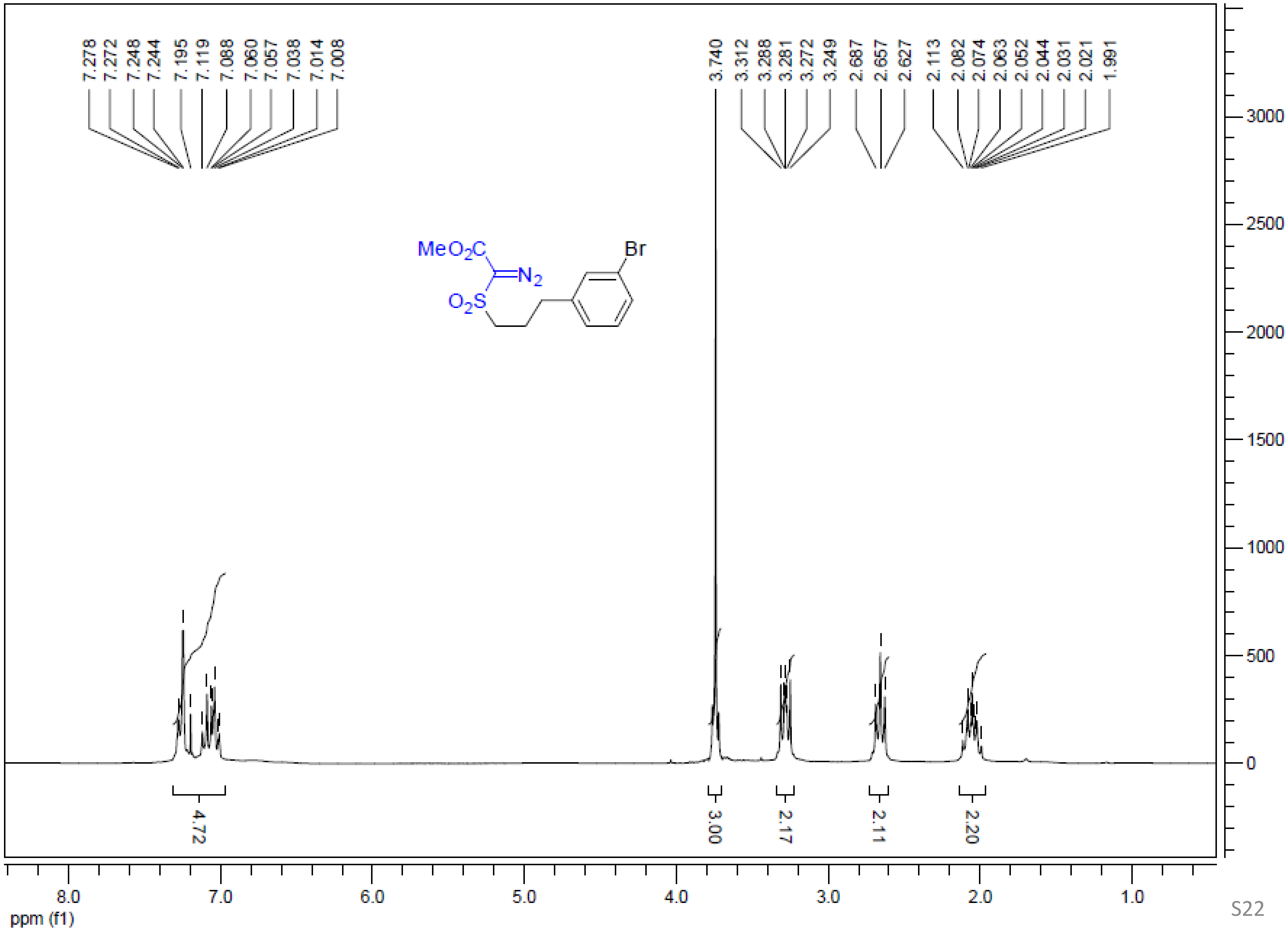

methyl 2-((3-(3-bromophenyl)propyl)sulfonyl)-2-diazoacetate (**1f**)

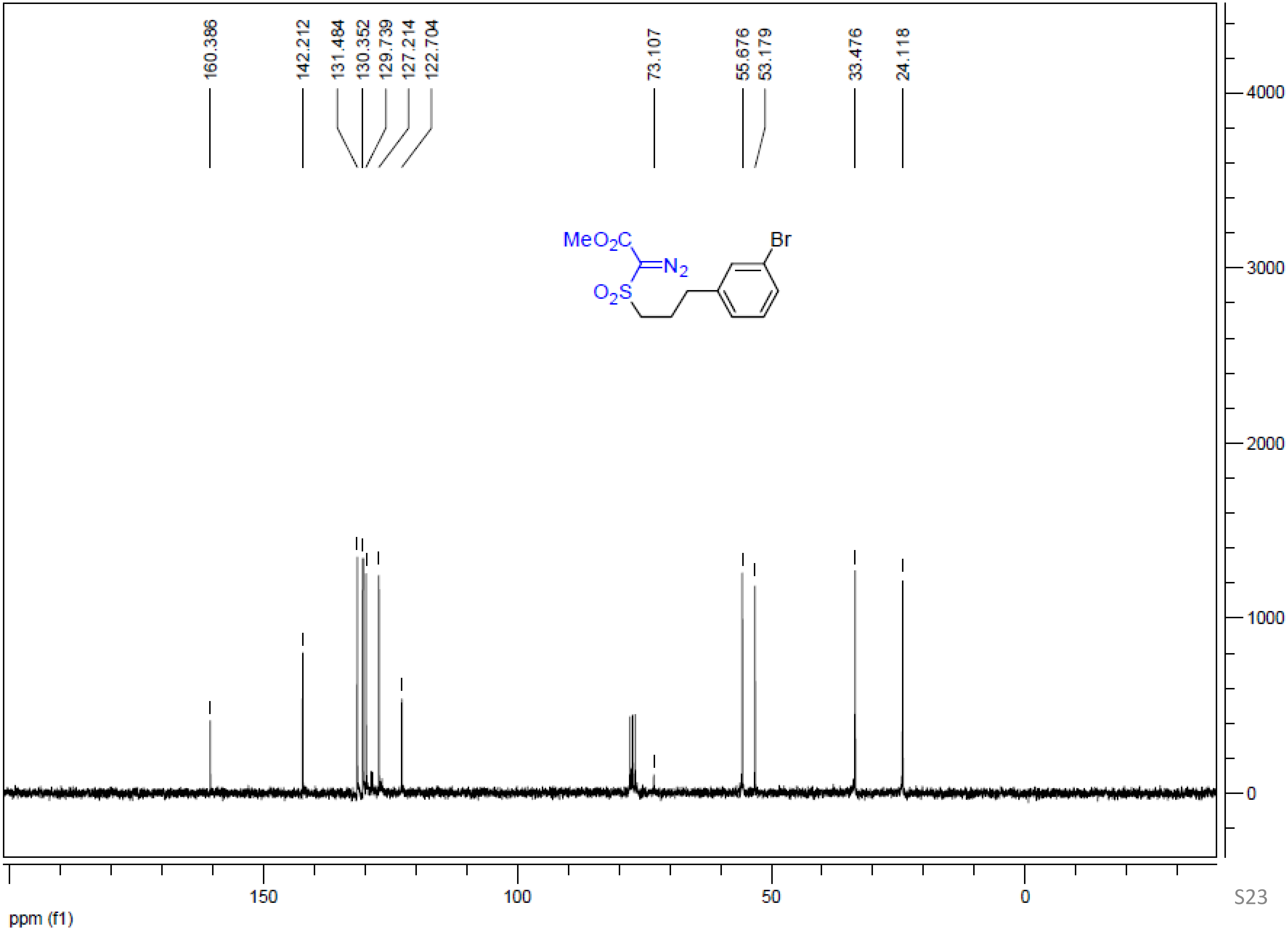

methyl 2-diazo-2-((3-phenylpropyl)sulfonyl)acetate (**1g**)

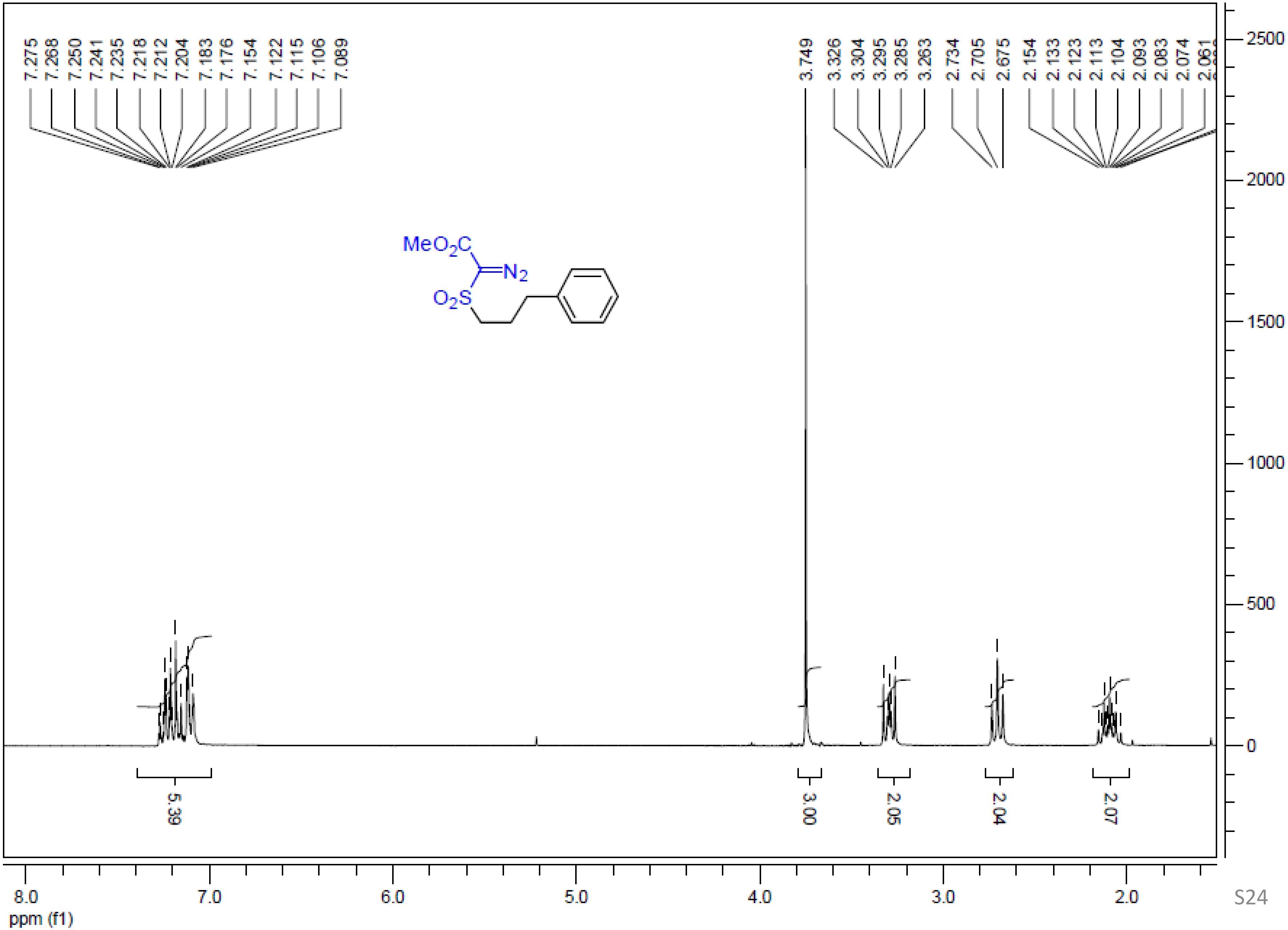

methyl 2-diazo-2-((3-phenylpropyl)sulfonyl)acetate (**1g**)

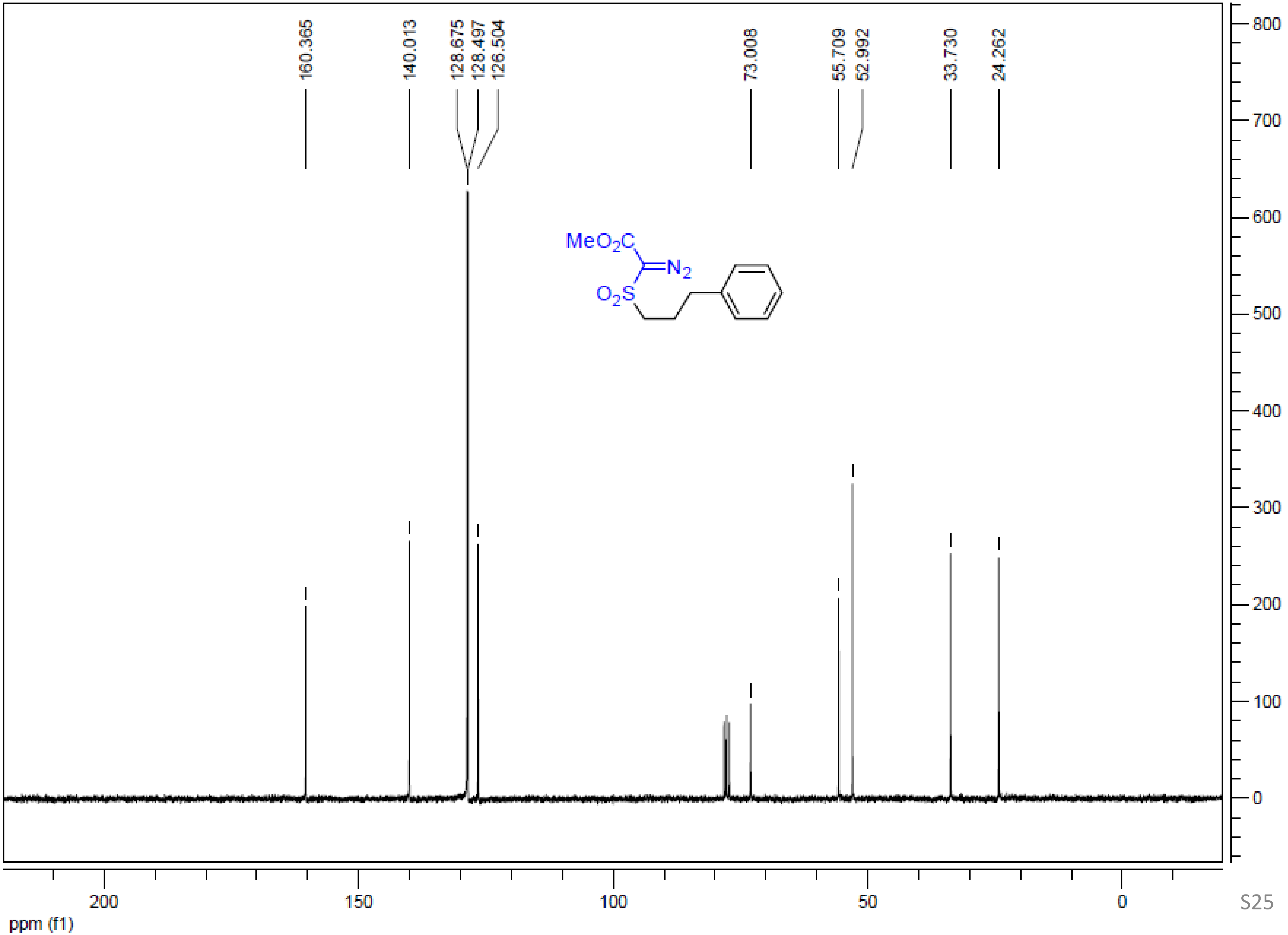

methyl 2-diazo-2-((3-(p-tolyl)propyl)sulfonyl)acetate (**1h**)

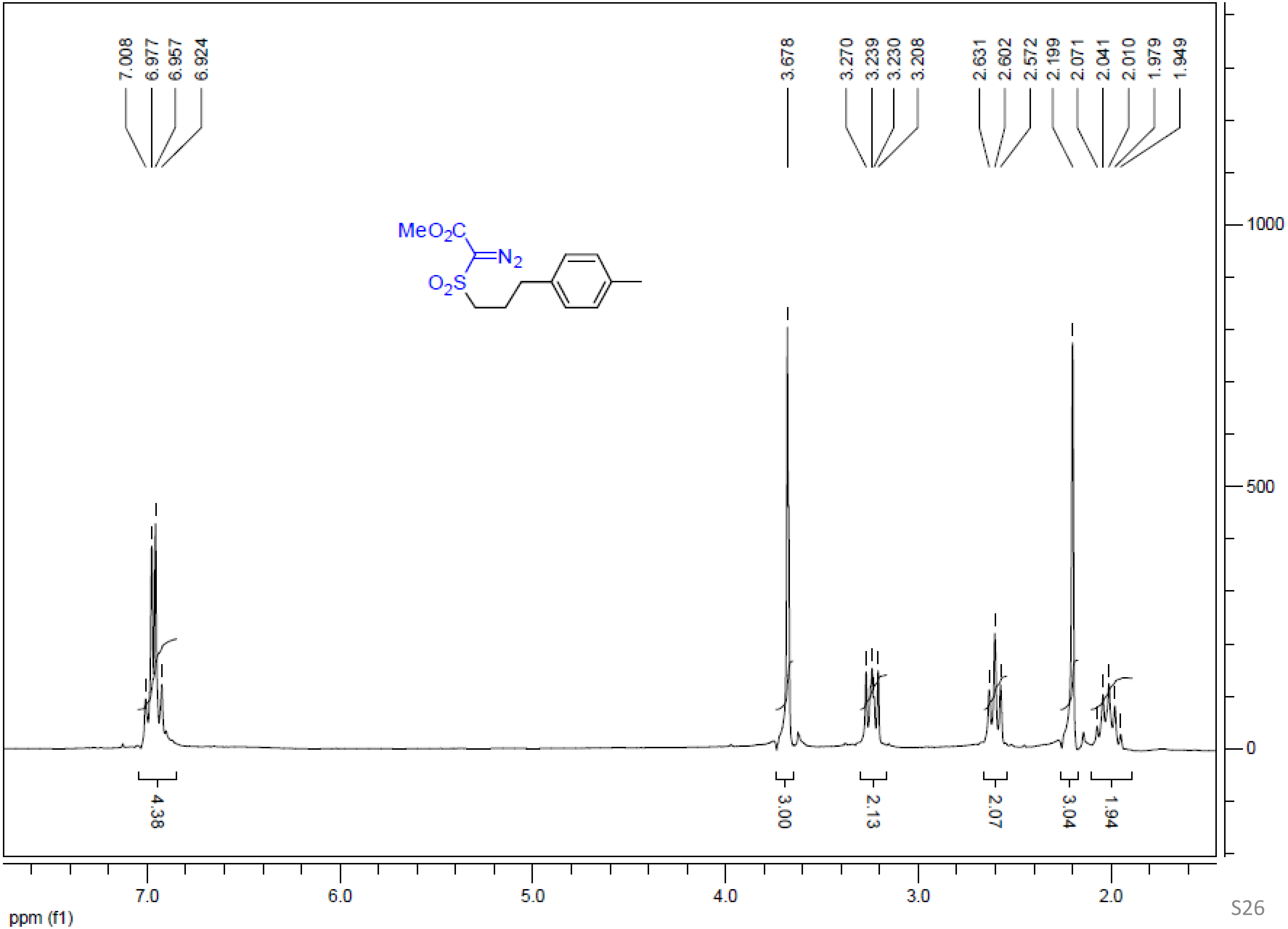

methyl 2-diazo-2-((3-(p-tolyl)propyl)sulfonyl)acetate (**1h**)

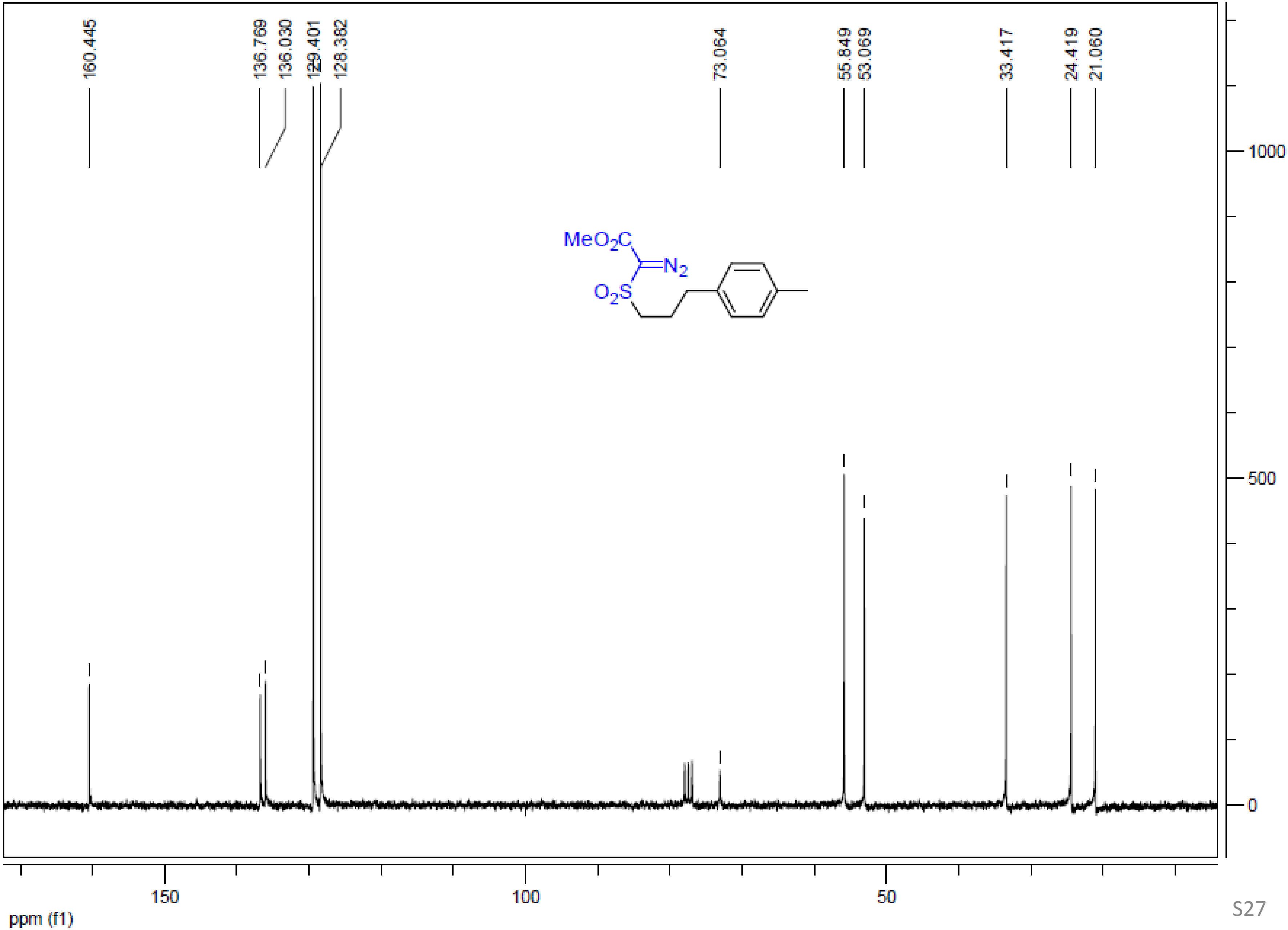

methyl 2-diazo-2-((3-(4-methoxyphenyl)propyl)sulfonyl)acetate (**1i**)

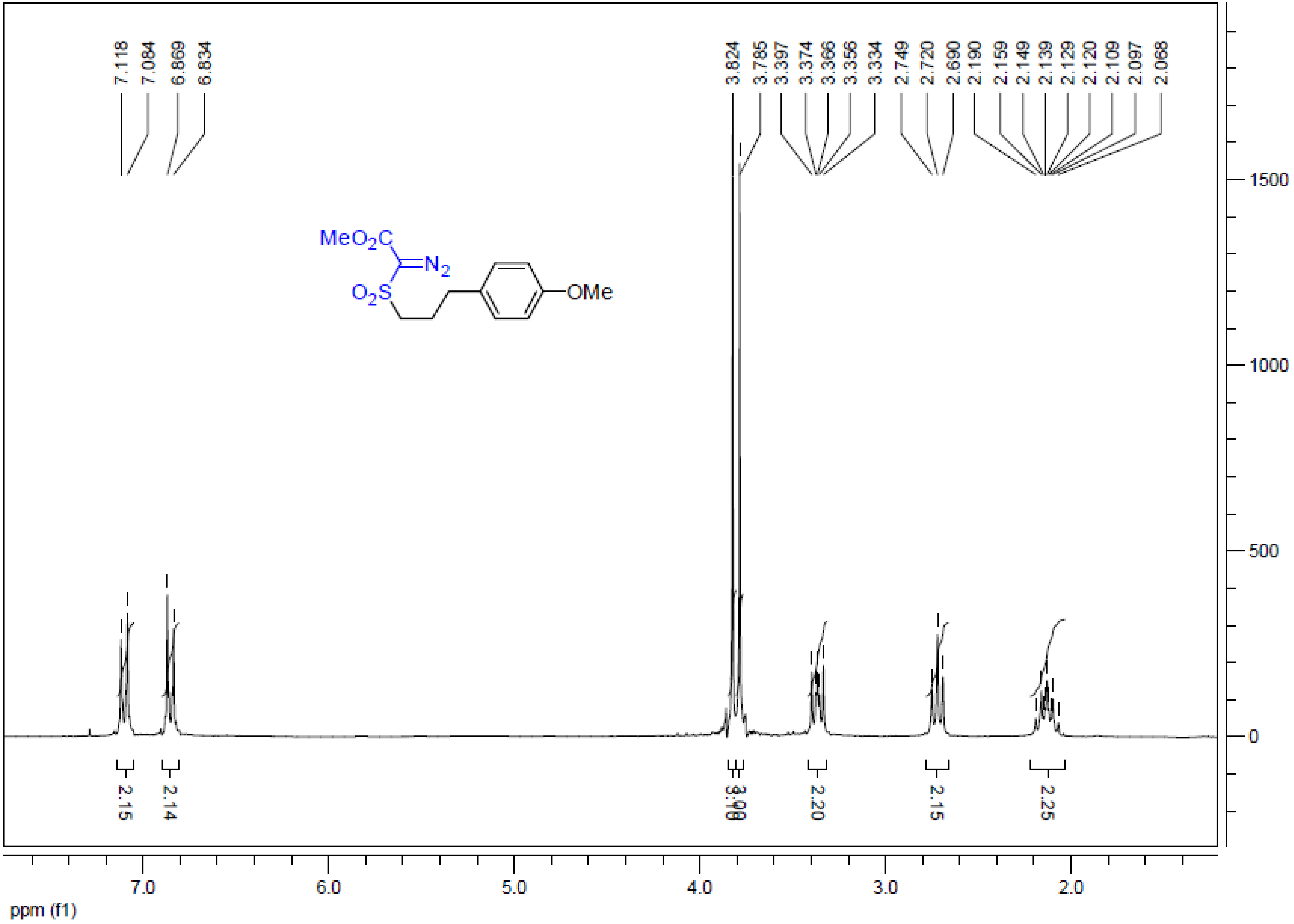

methyl 2-diazo-2-((3-(4-methoxyphenyl)propyl)sulfonyl)acetate (**1i**)

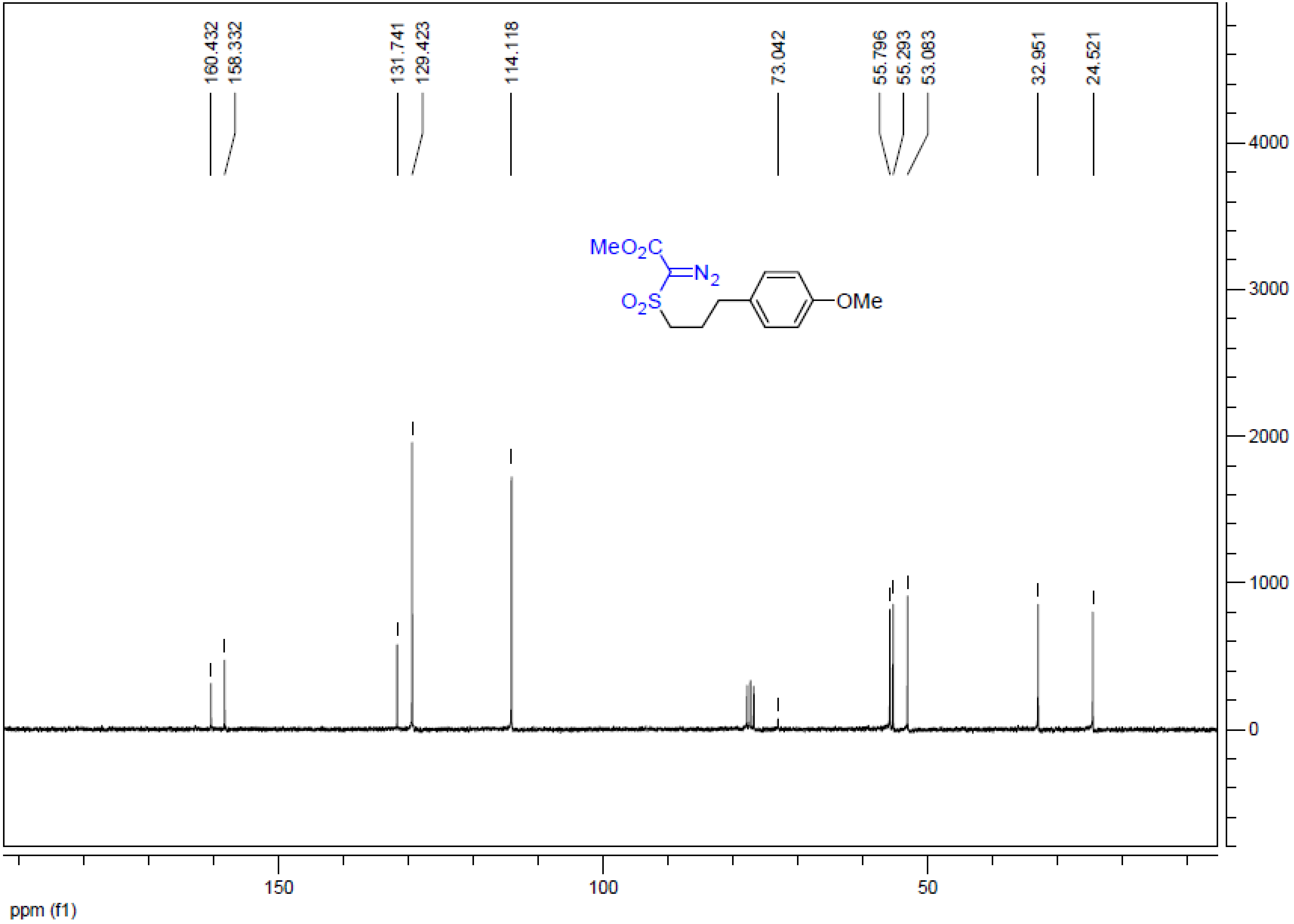

methyl 2-diazo-2-((3-(4-(methoxymethoxy)phenyl)propyl)sulfonyl)acetate (**1j**)

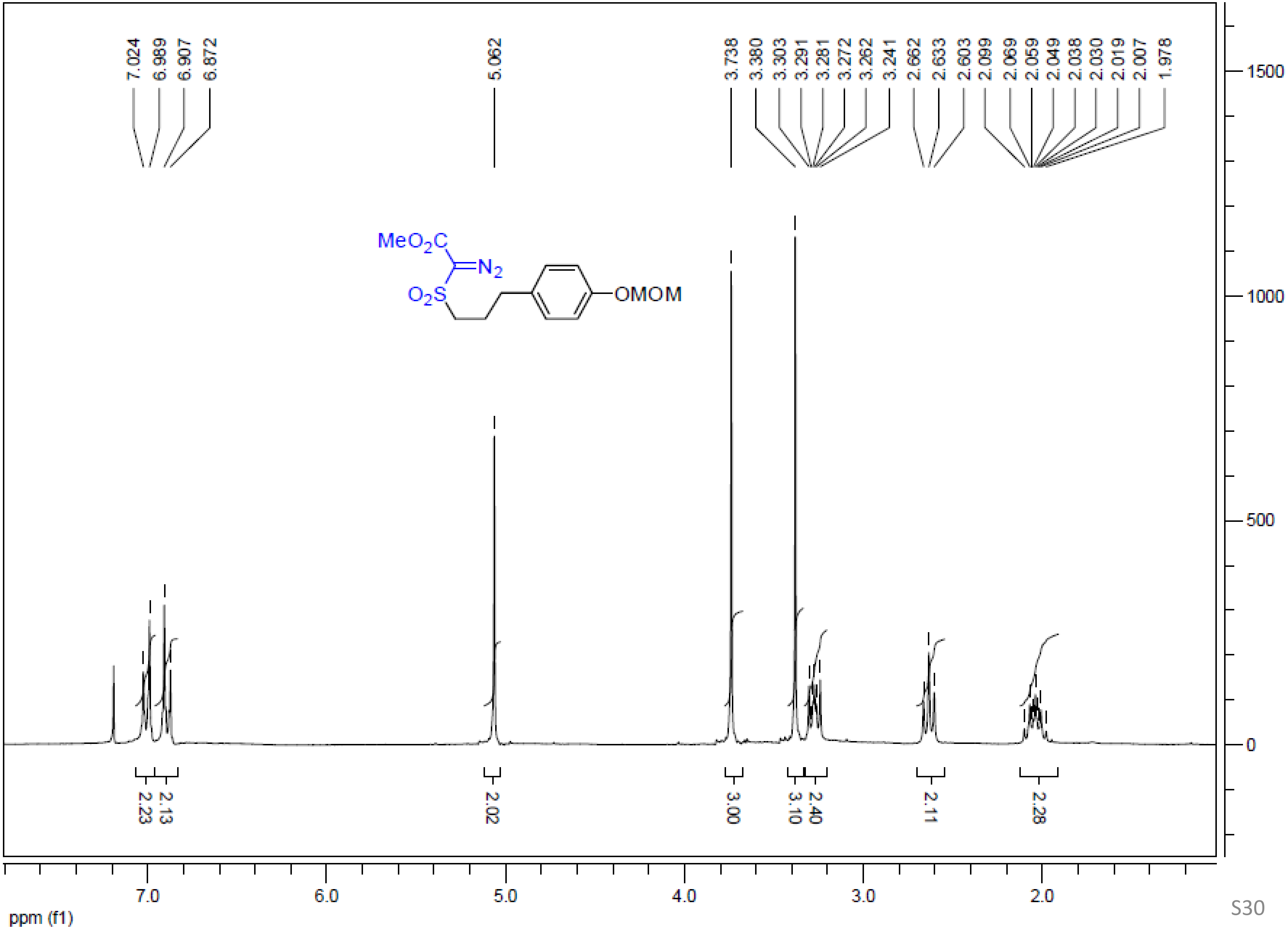

methyl 2-diazo-2-((3-(4-(methoxymethoxy)phenyl)propyl)sulfonyl)acetate (**1j**)

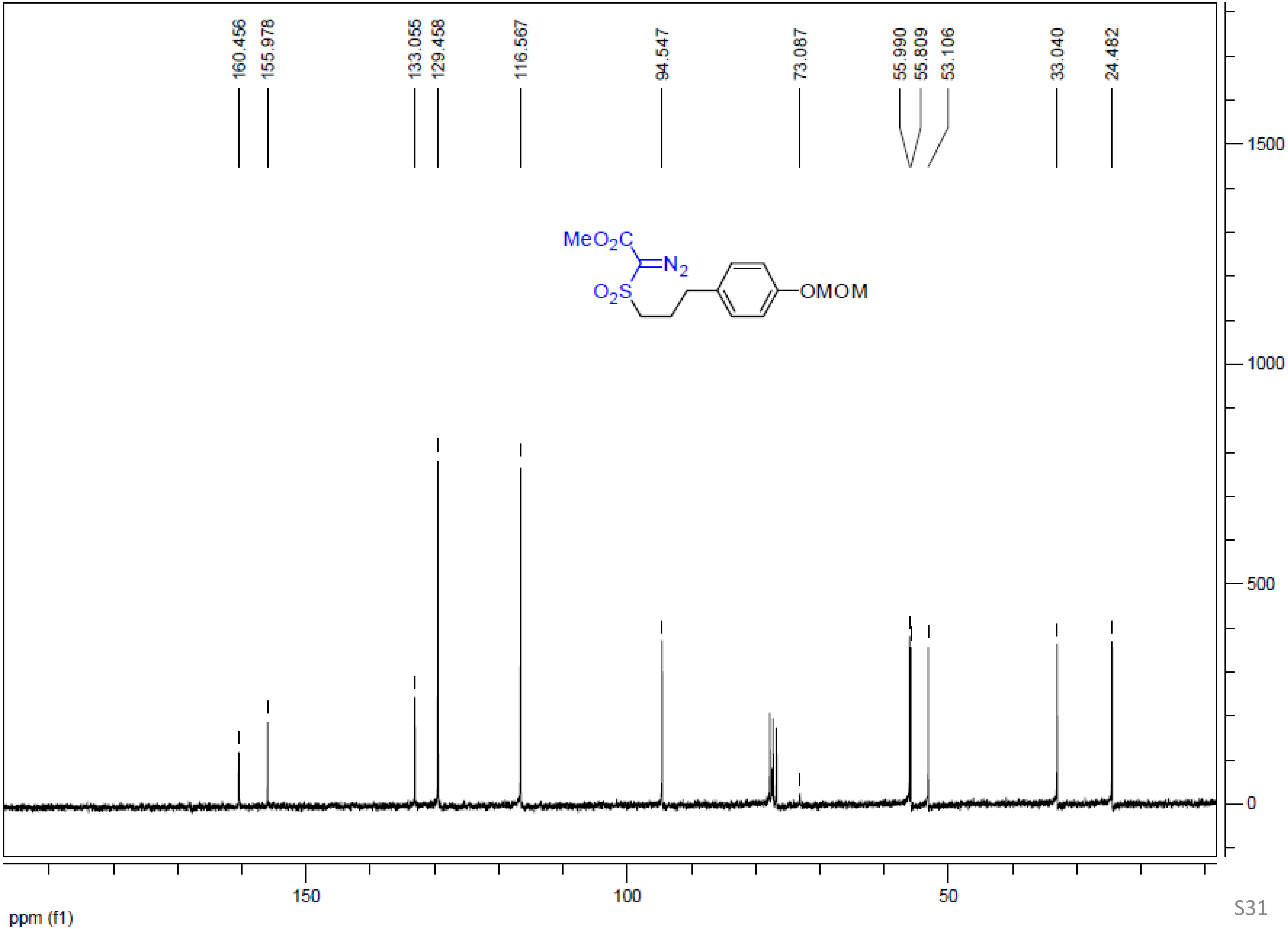

methyl 2-diazo-2-((3-(4-hydroxyphenyl)propyl)sulfonyl)acetate (**1k**)

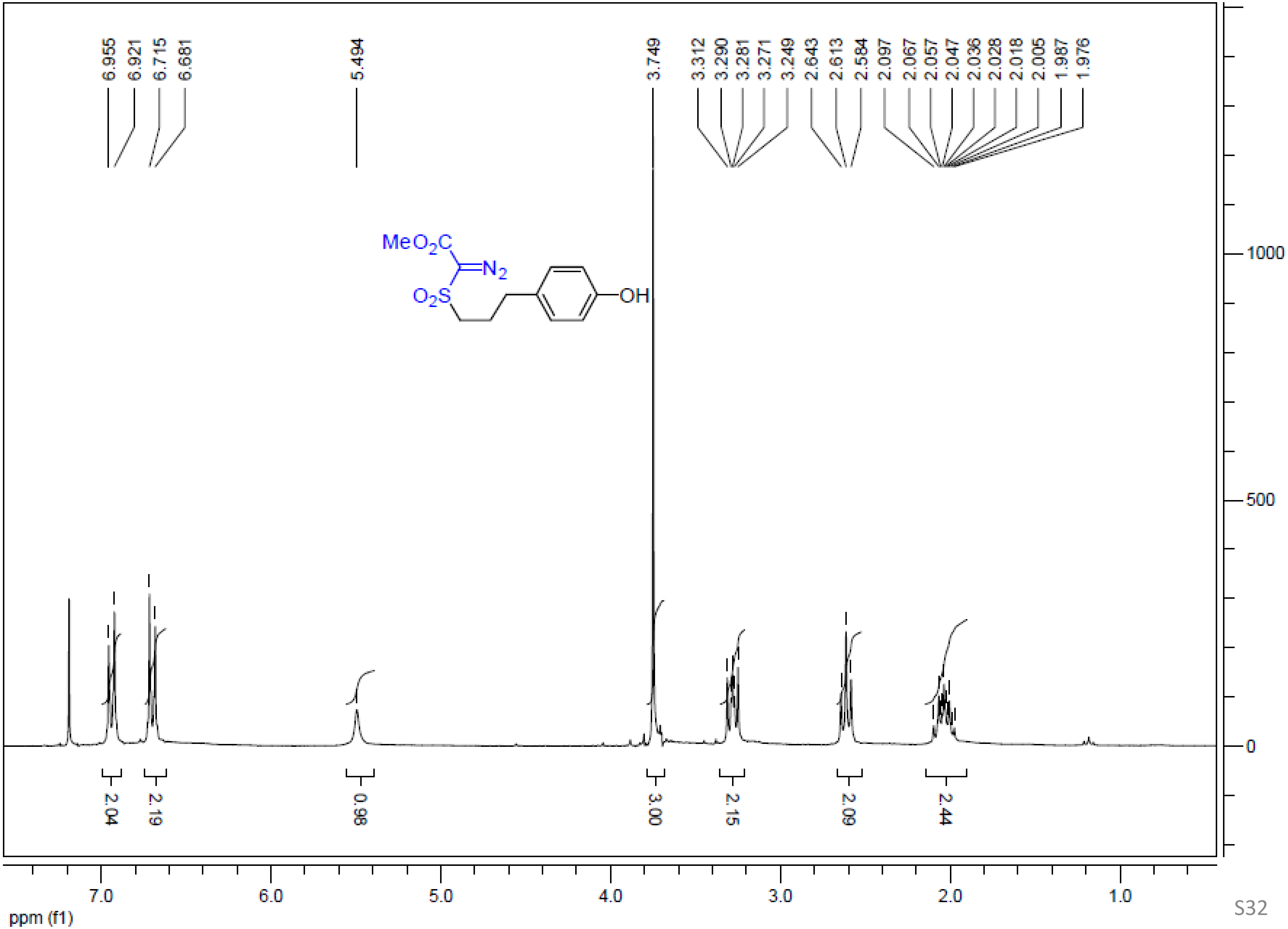

methyl 2-diazo-2-((3-(4-hydroxyphenyl)propyl)sulfonyl)acetate (**1k**)

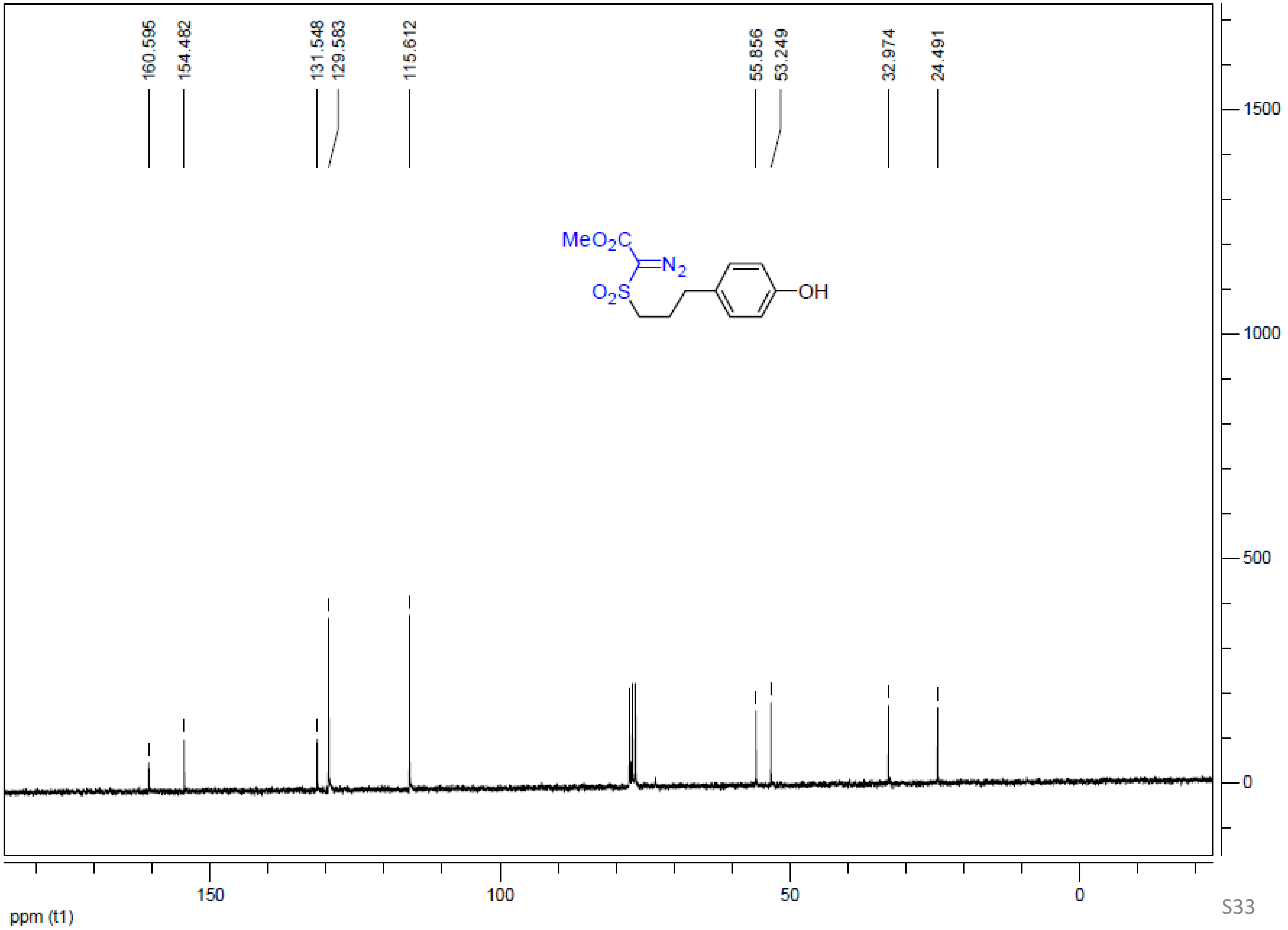

methyl 2-((3-(4-aminophenyl)propyl)sulfonyl)-2-diazoacetate (**1l**)

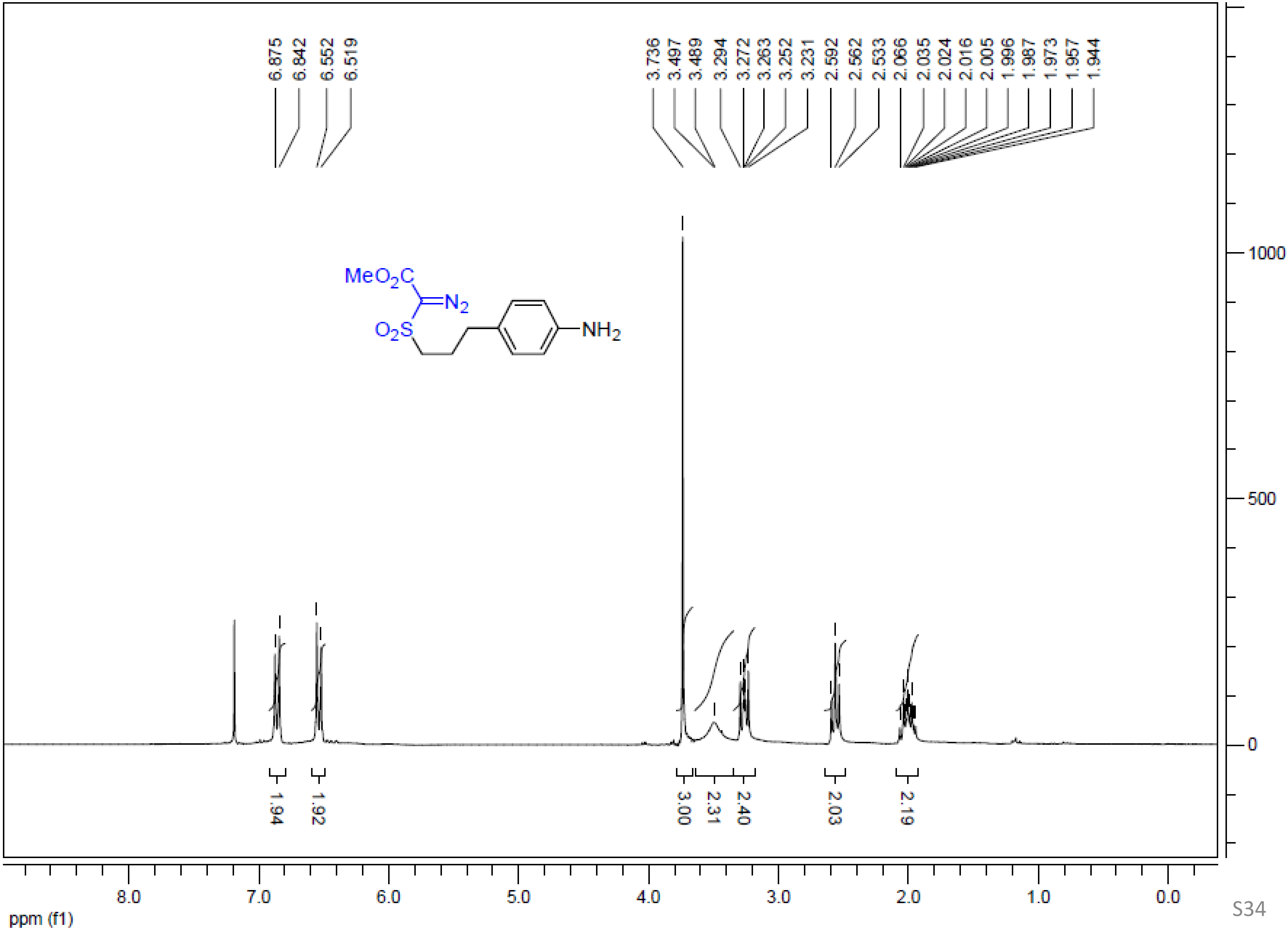

methyl 2-((3-(4-aminophenyl)propyl)sulfonyl)-2-diazoacetate (**1l**)

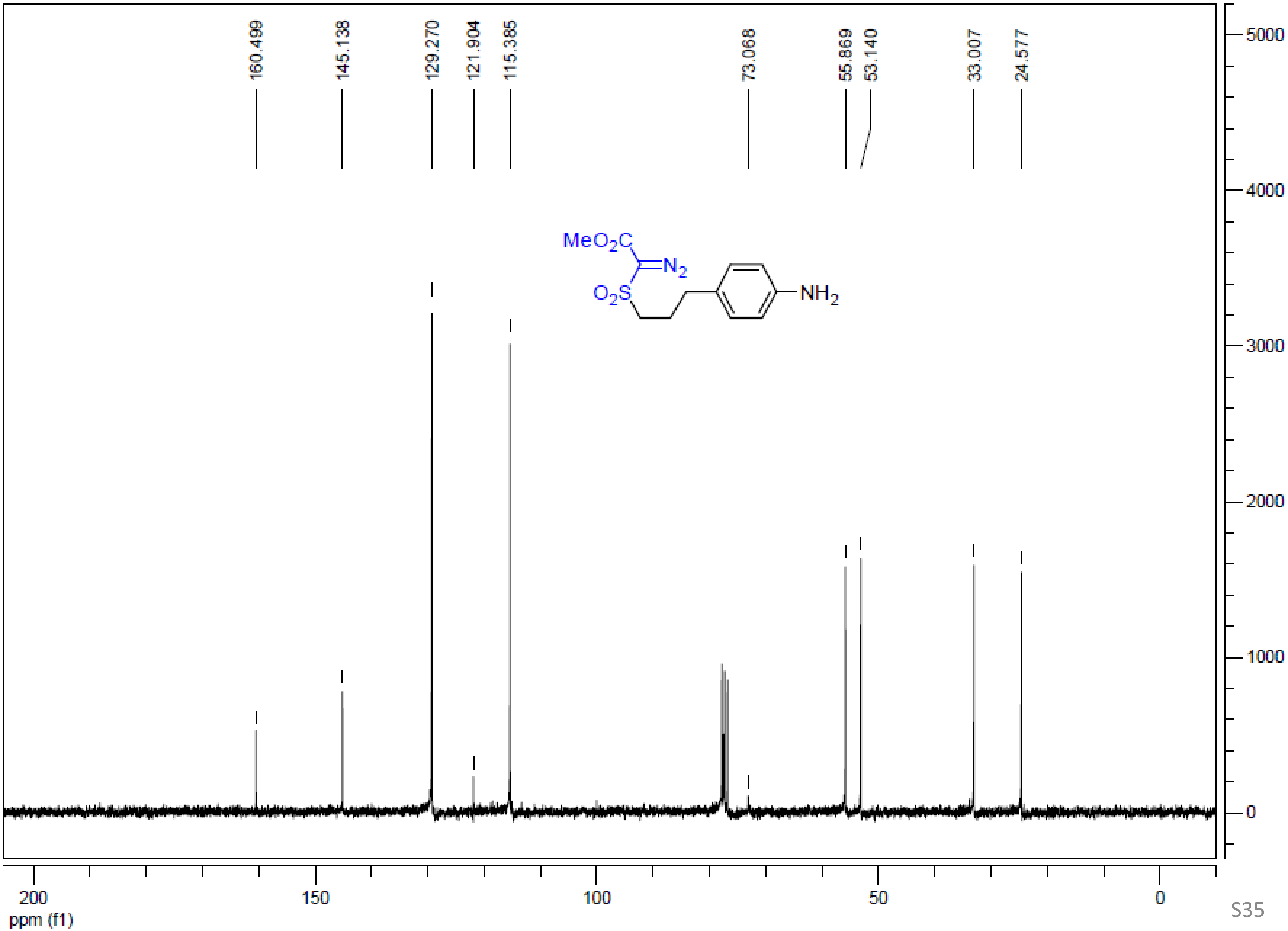

methyl 2-((3-(4-acetamidophenyl)propyl)sulfonyl)-2-diazoacetate (**1m**)

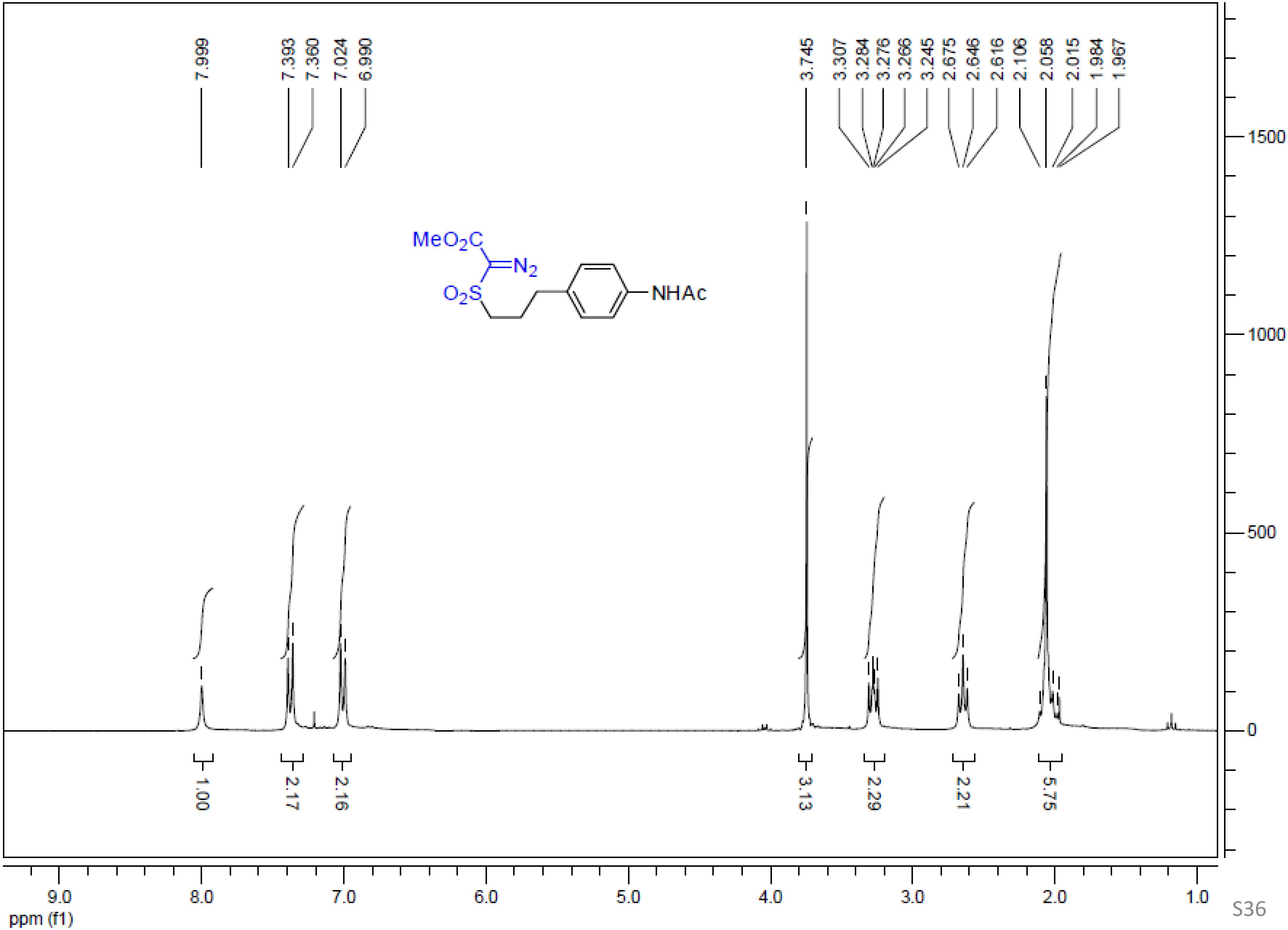

methyl 2-((3-(4-acetamidophenyl)propyl)sulfonyl)-2-diazoacetate (**1m**)

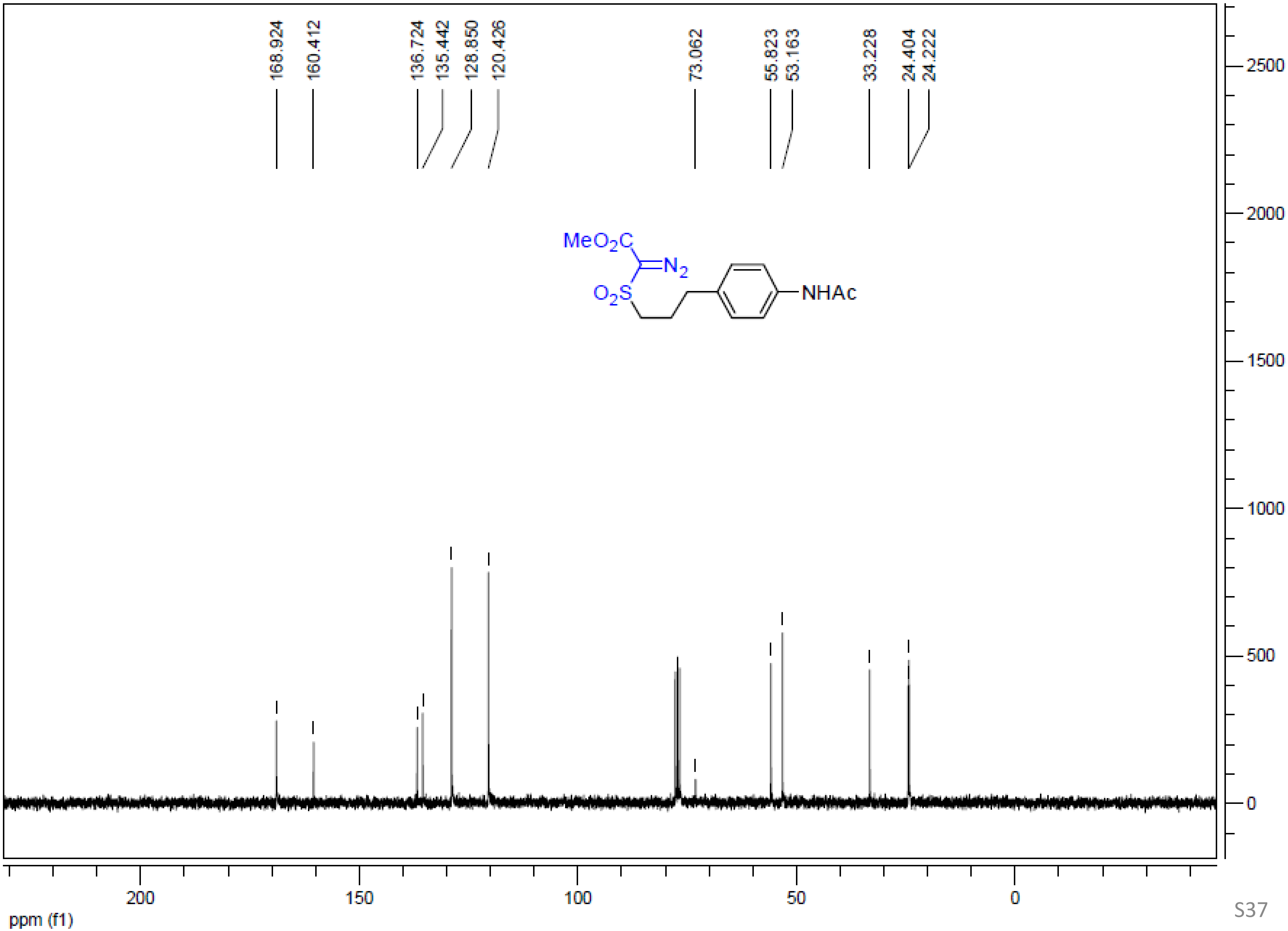

methyl 2-diazo-2-((3-(1-phenyl-1H-1,2,3-triazol-4-yl)propyl)sulfonyl)acetate (**1n**)

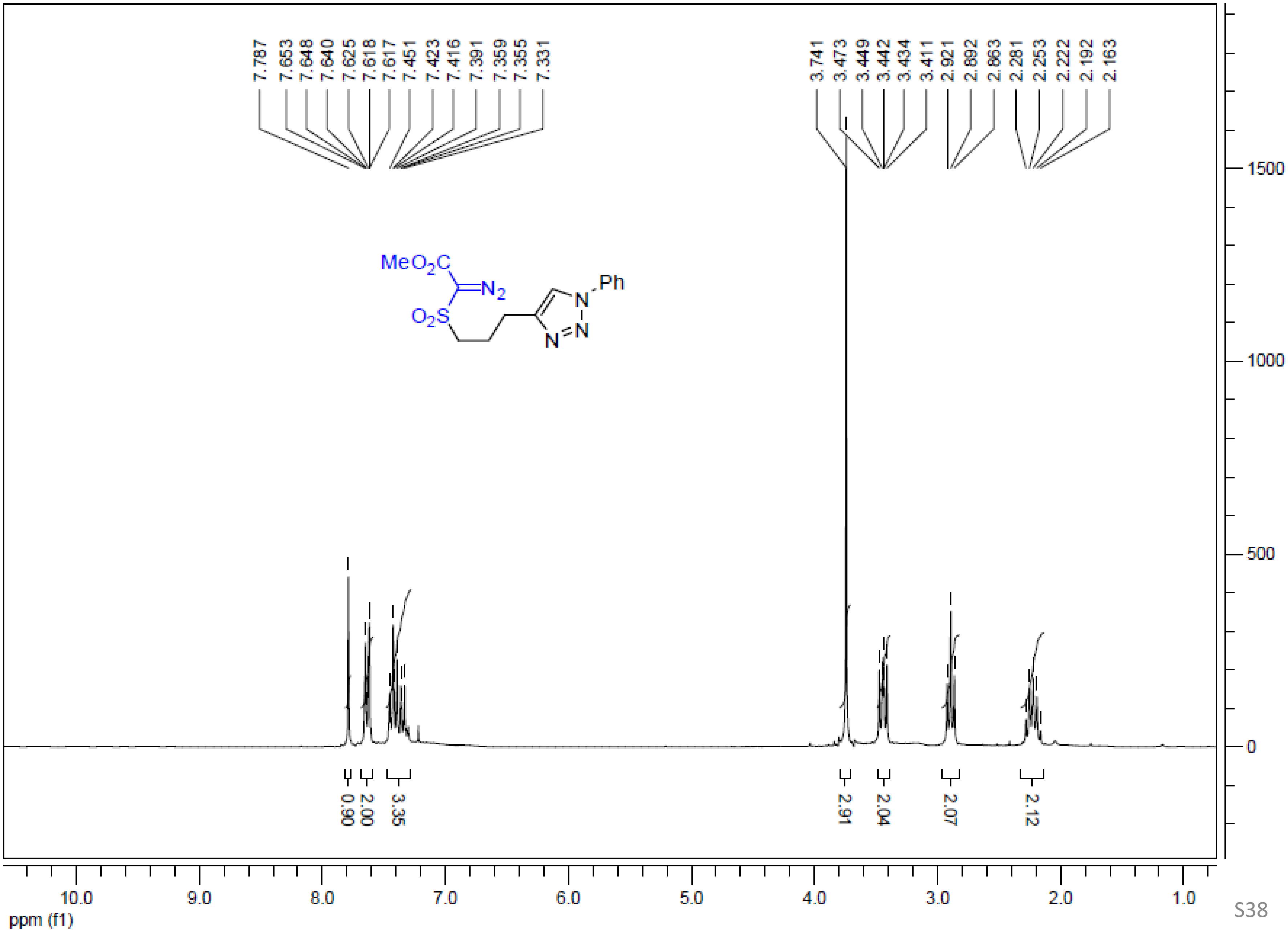

methyl 2-diazo-2-((3-(1-phenyl-1H-1,2,3-triazol-4-yl)propyl)sulfonyl)acetate (**1n**)

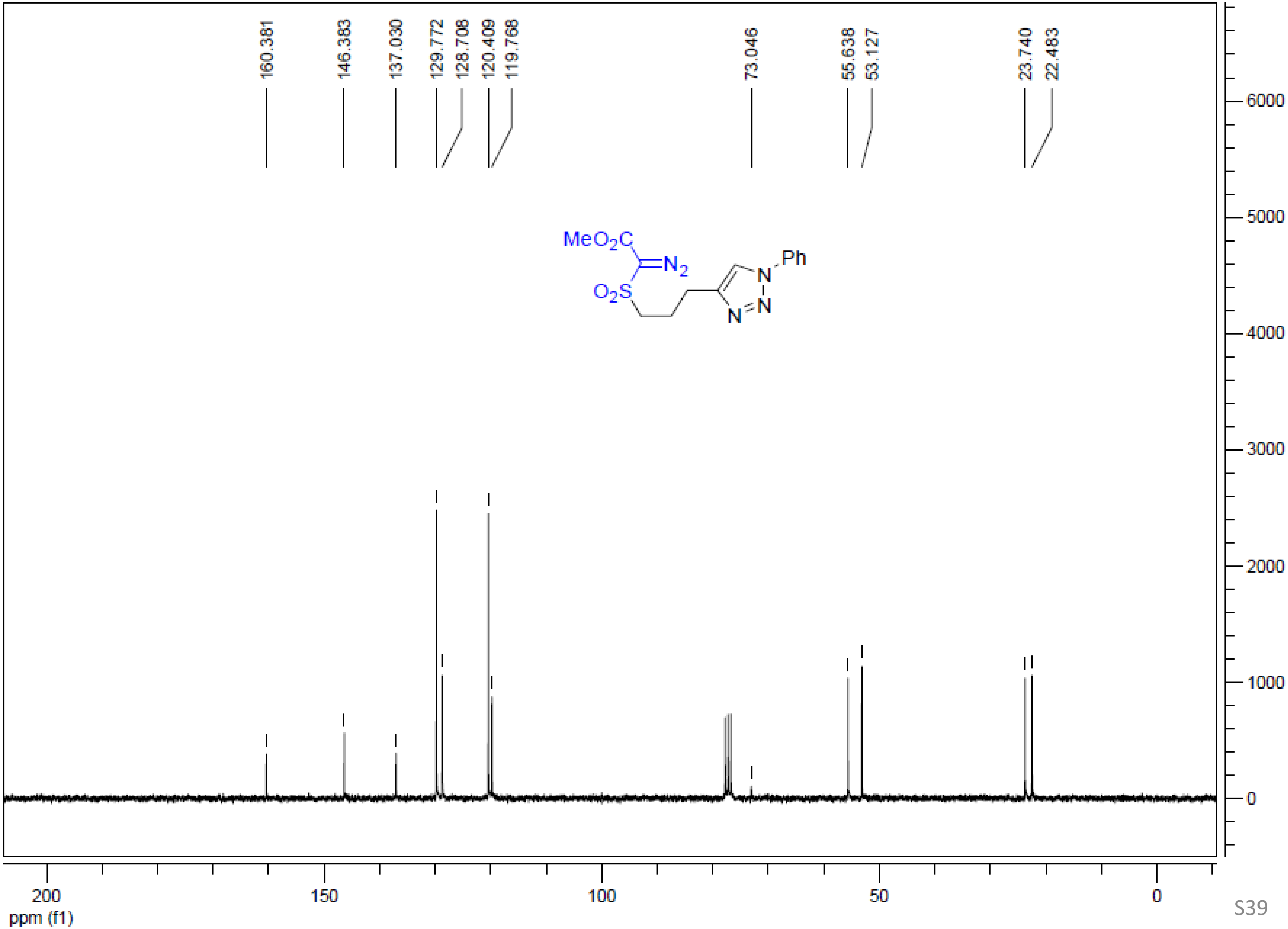

methyl 2-diazo-2-(pent-4-en-1-ylsulfonyl)acetate (**1o**)

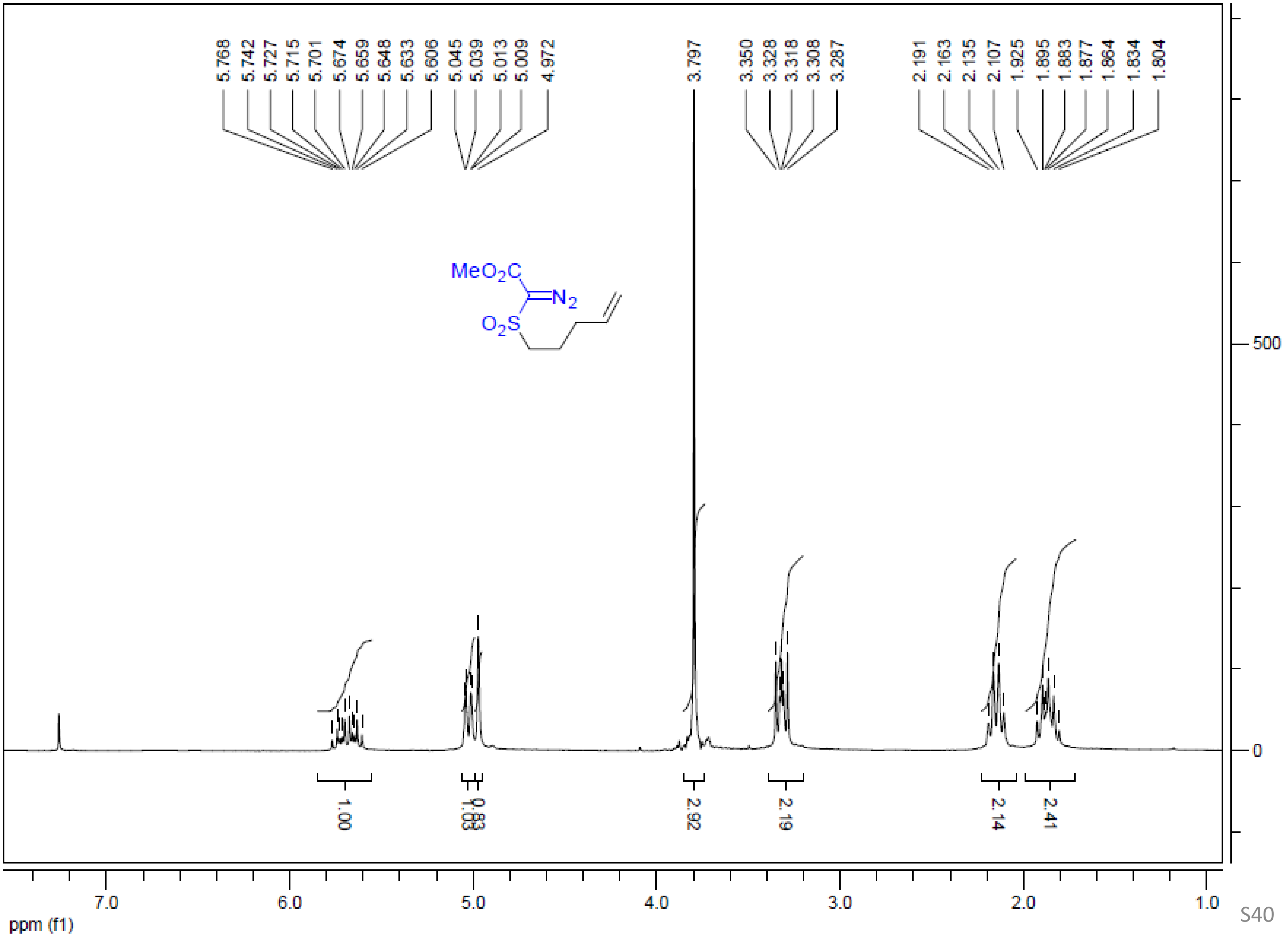

methyl 2-diazo-2-(pent-4-en-1-ylsulfonyl)acetate (**1o**)

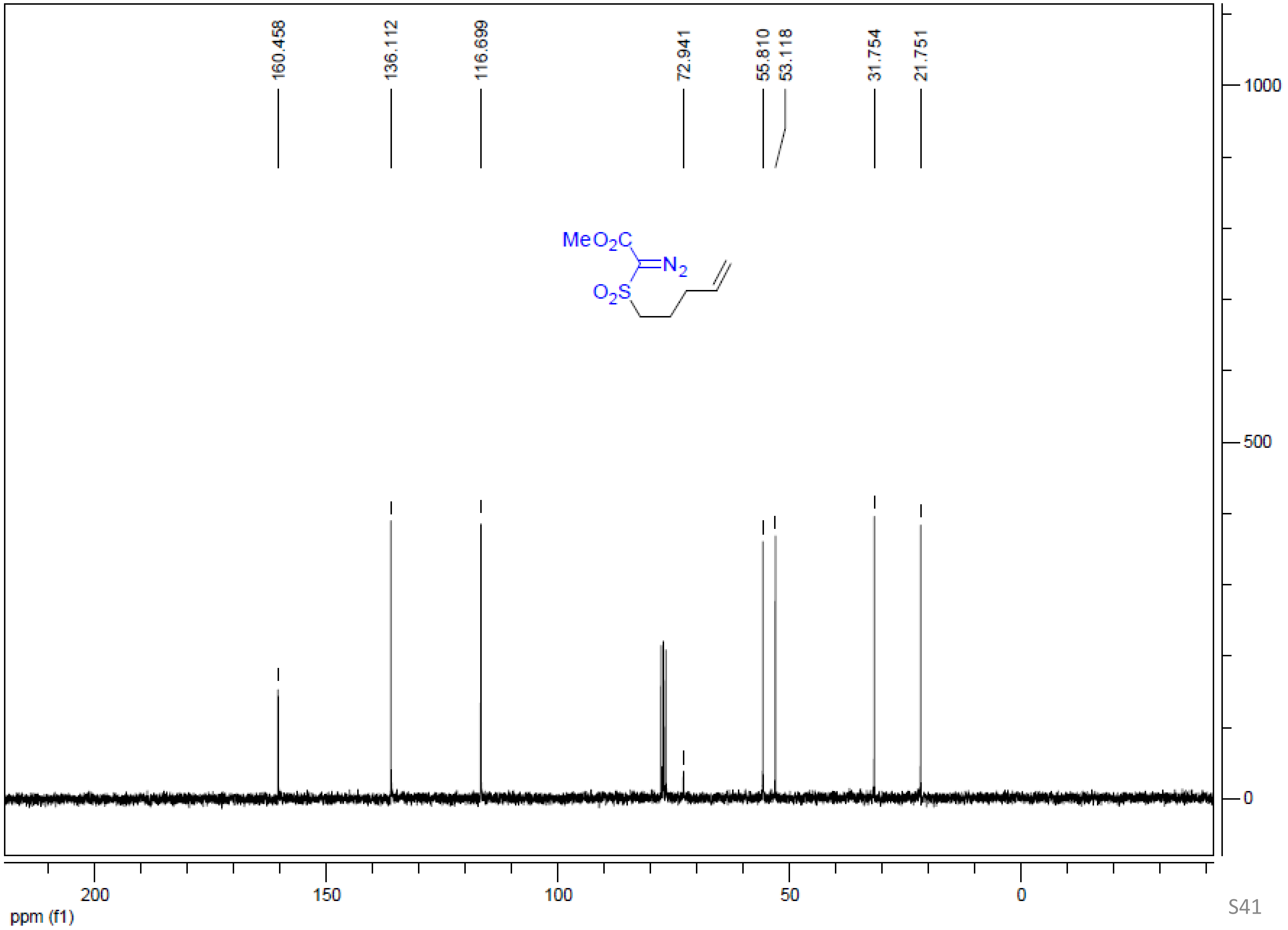

methyl 2-diazo-2-((4-methylpent-4-en-1-yl)sulfonyl)acetate (**1p**)

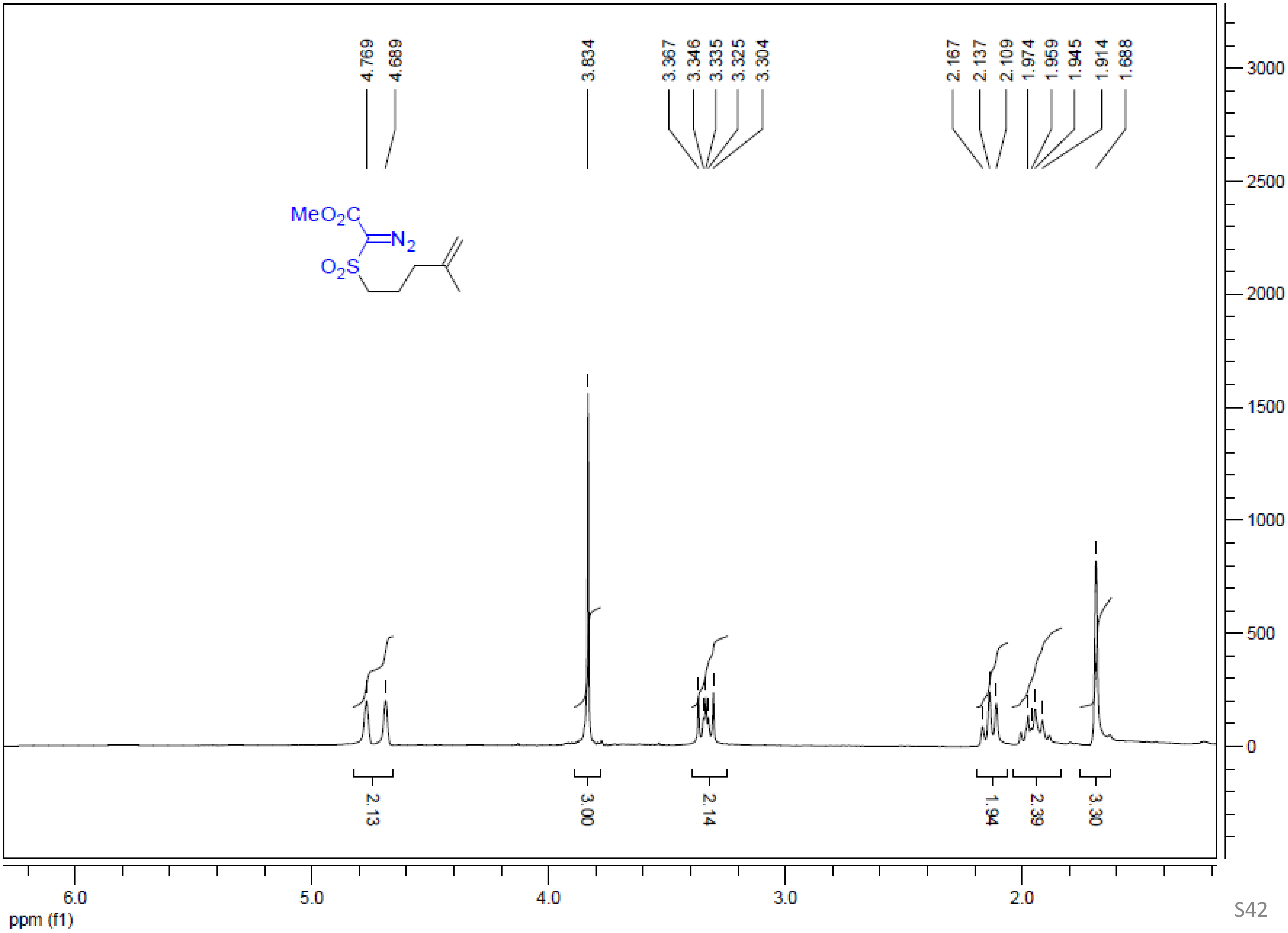

methyl 2-diazo-2-((4-methylpent-4-en-1-yl)sulfonyl)acetate (**1p**)

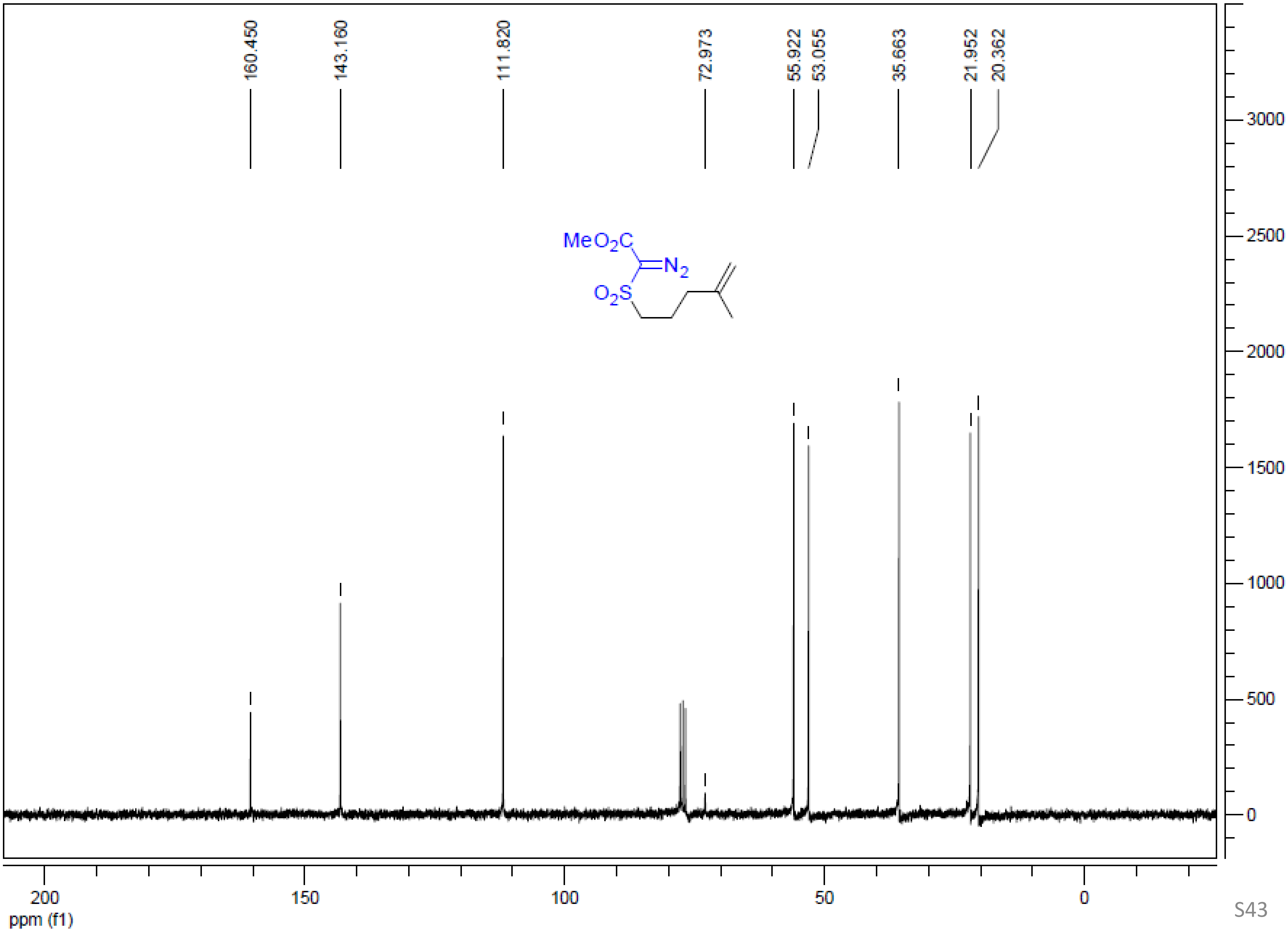

methyl 2-diazo-2-(hexa-4,5-dien-1-ylsulfonyl)acetate (**1q**)

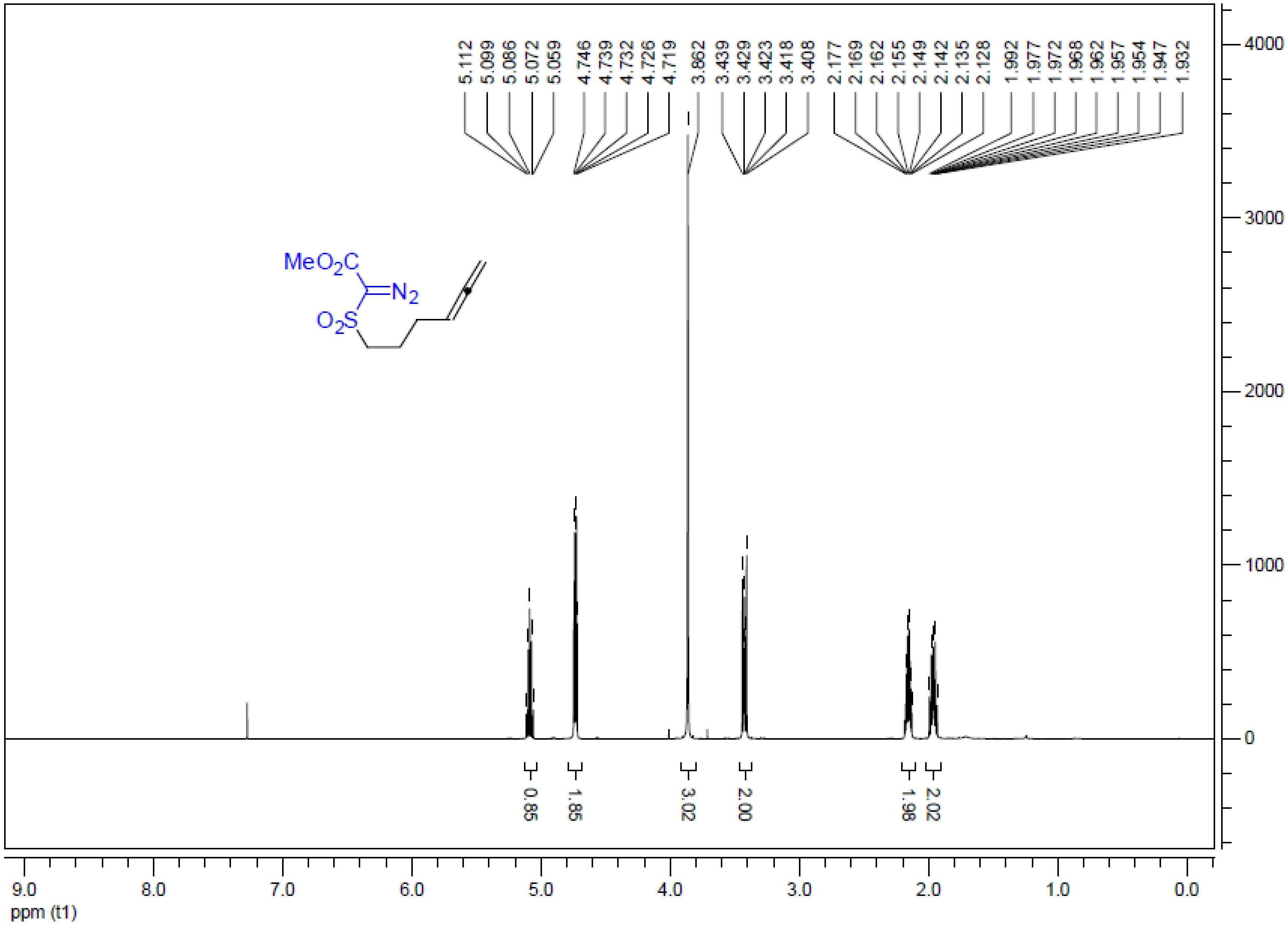

methyl 2-diazo-2-(hexa-4,5-dien-1-ylsulfonyl)acetate (**1q**)

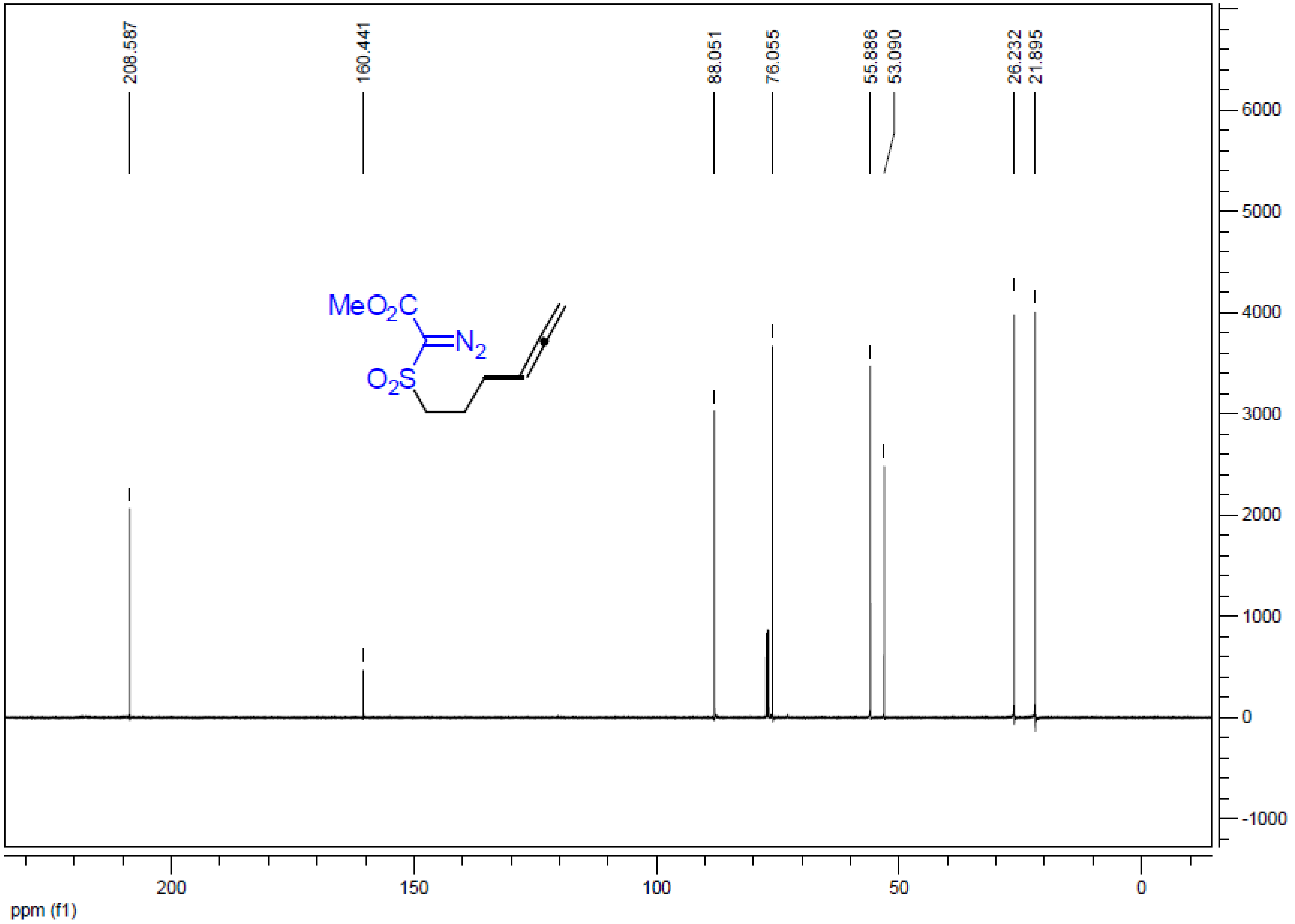

(E)-methyl 2-diazo-2-(hex-4-en-1-ylsulfonyl)acetate (**1r**)

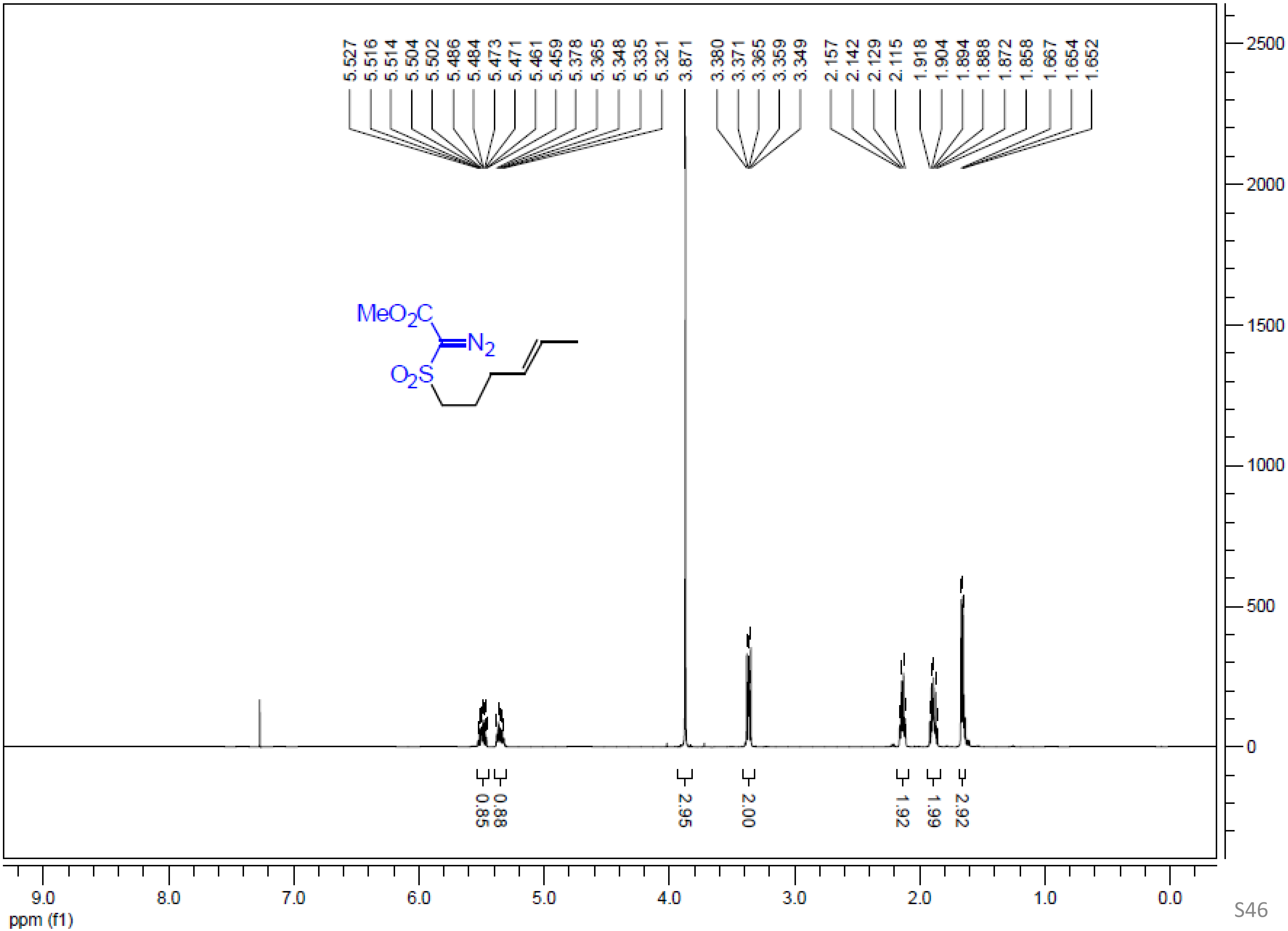

(E)-methyl 2-diazo-2-(hex-4-en-1-ylsulfonyl)acetate (**1r**)

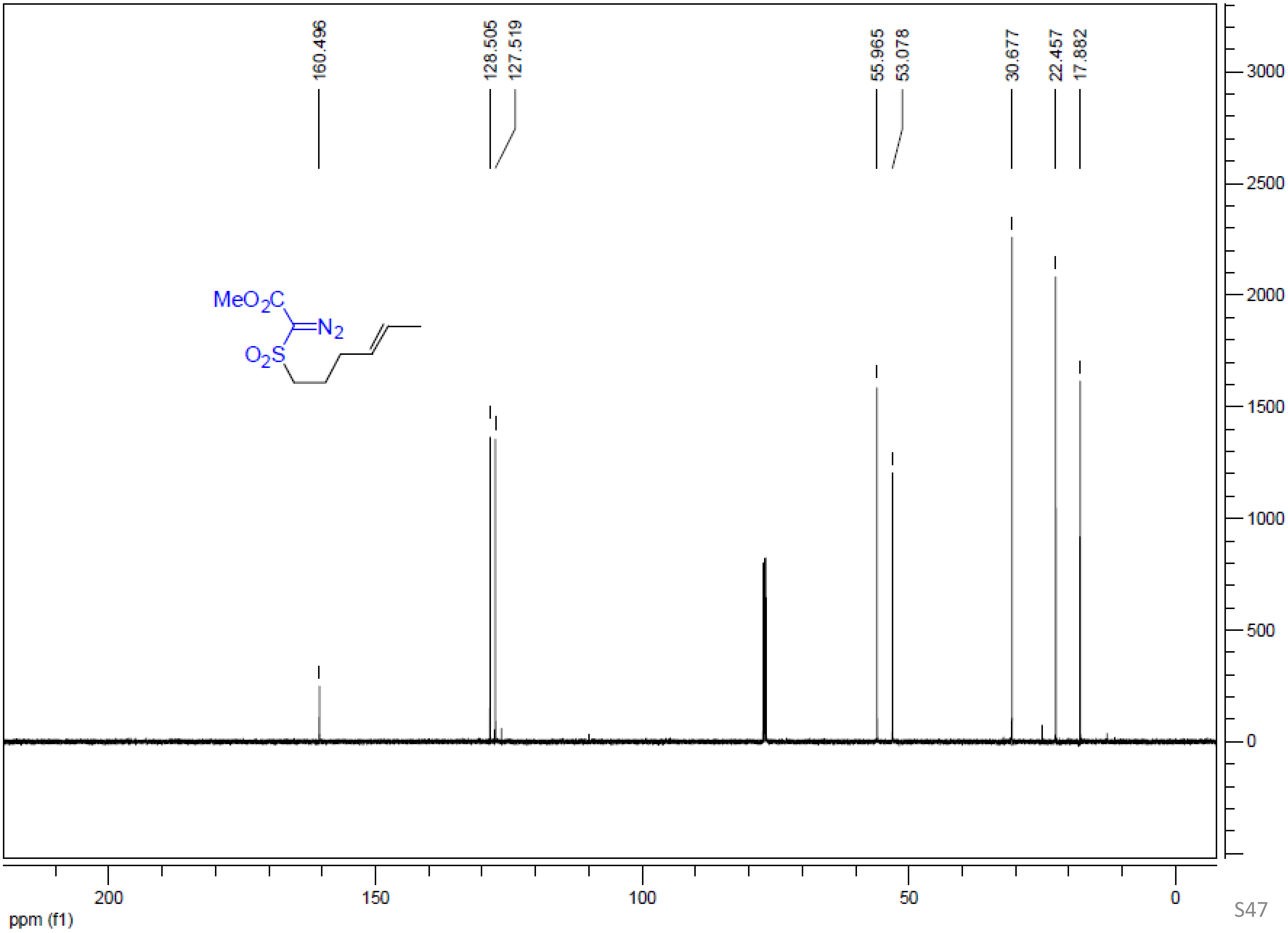

(Z)-methyl 2-diazo-2-(hex-4-en-1-ylsulfonyl)acetate (**1s**)

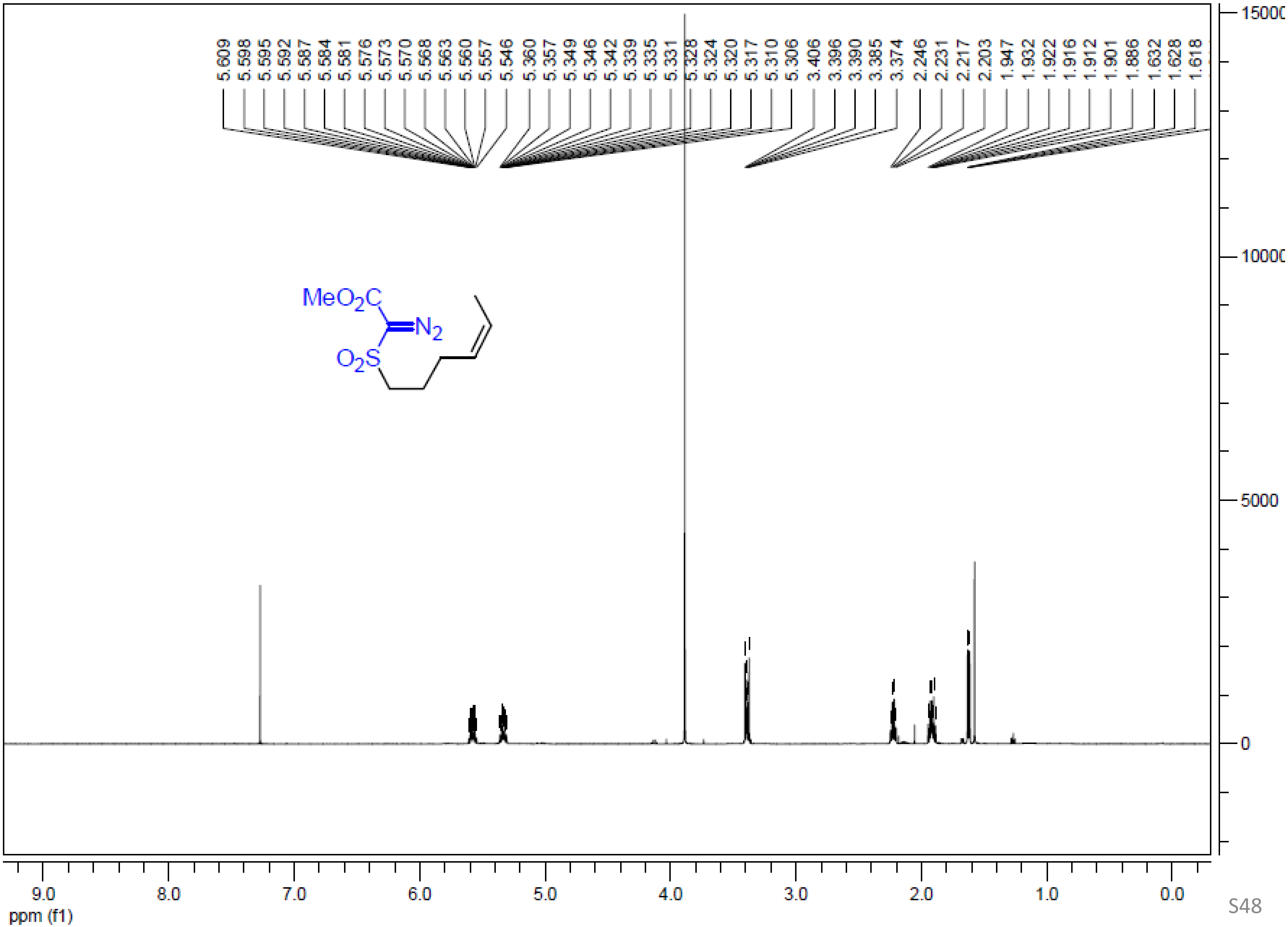

(Z)-methyl 2-diazo-2-(hex-4-en-1-ylsulfonyl)acetate (**1s**)

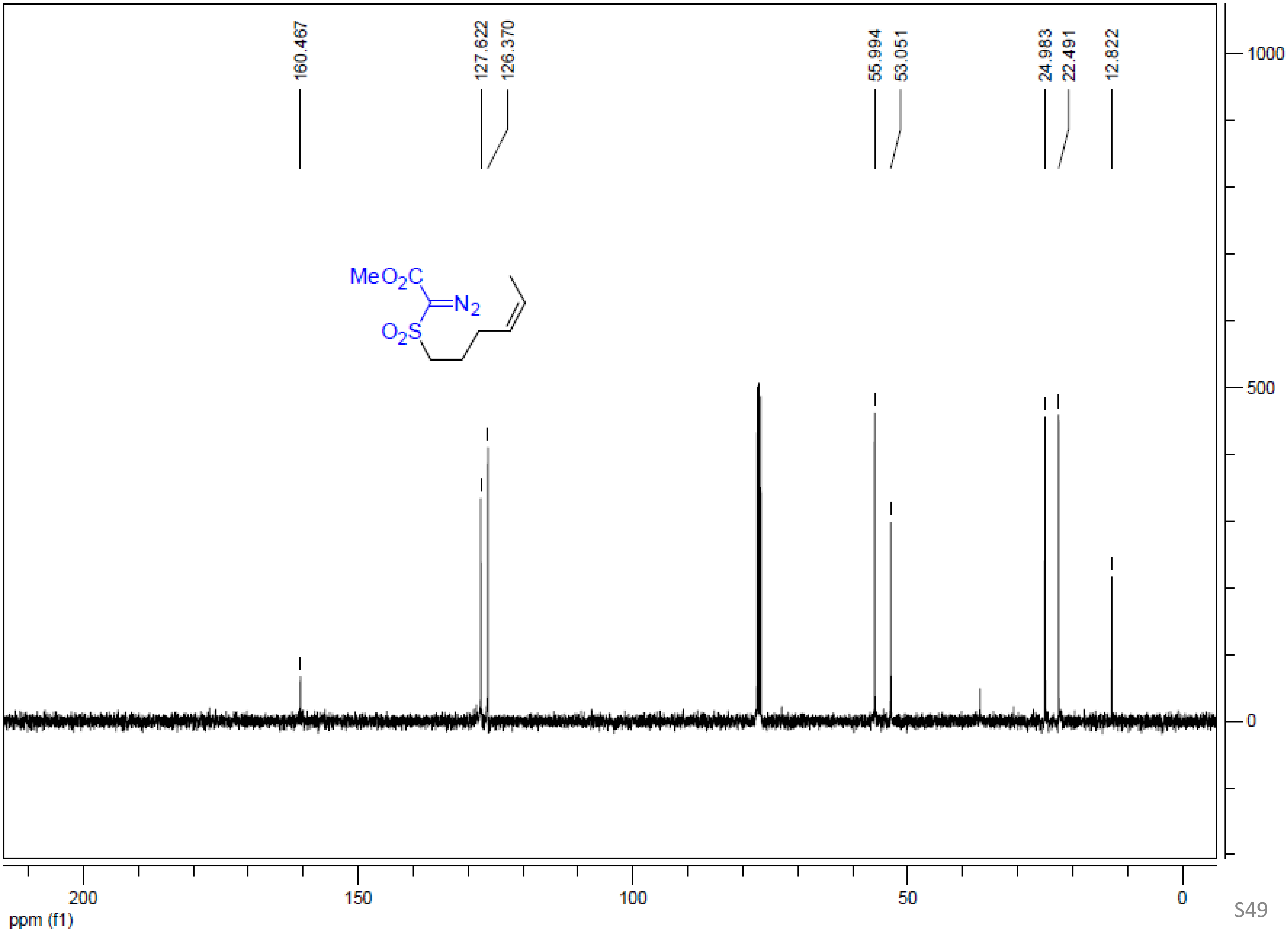

methyl 3-(4-nitrophenyl)tetrahydrothiophene-2-carboxylate 1,1-dioxide (**2a**)

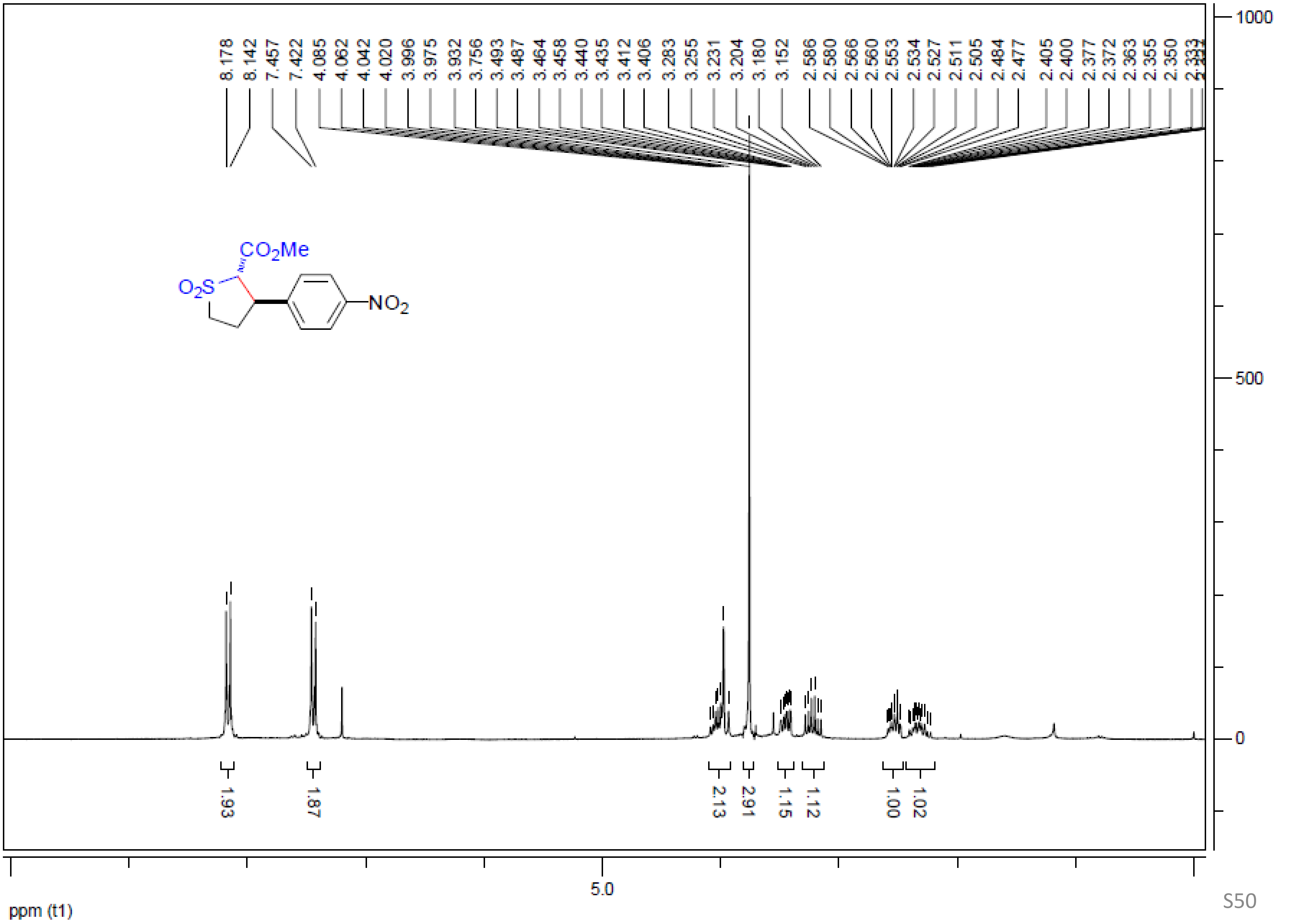

methyl 3-(4-nitrophenyl)tetrahydrothiophene-2-carboxylate 1,1-dioxide (**2a**)

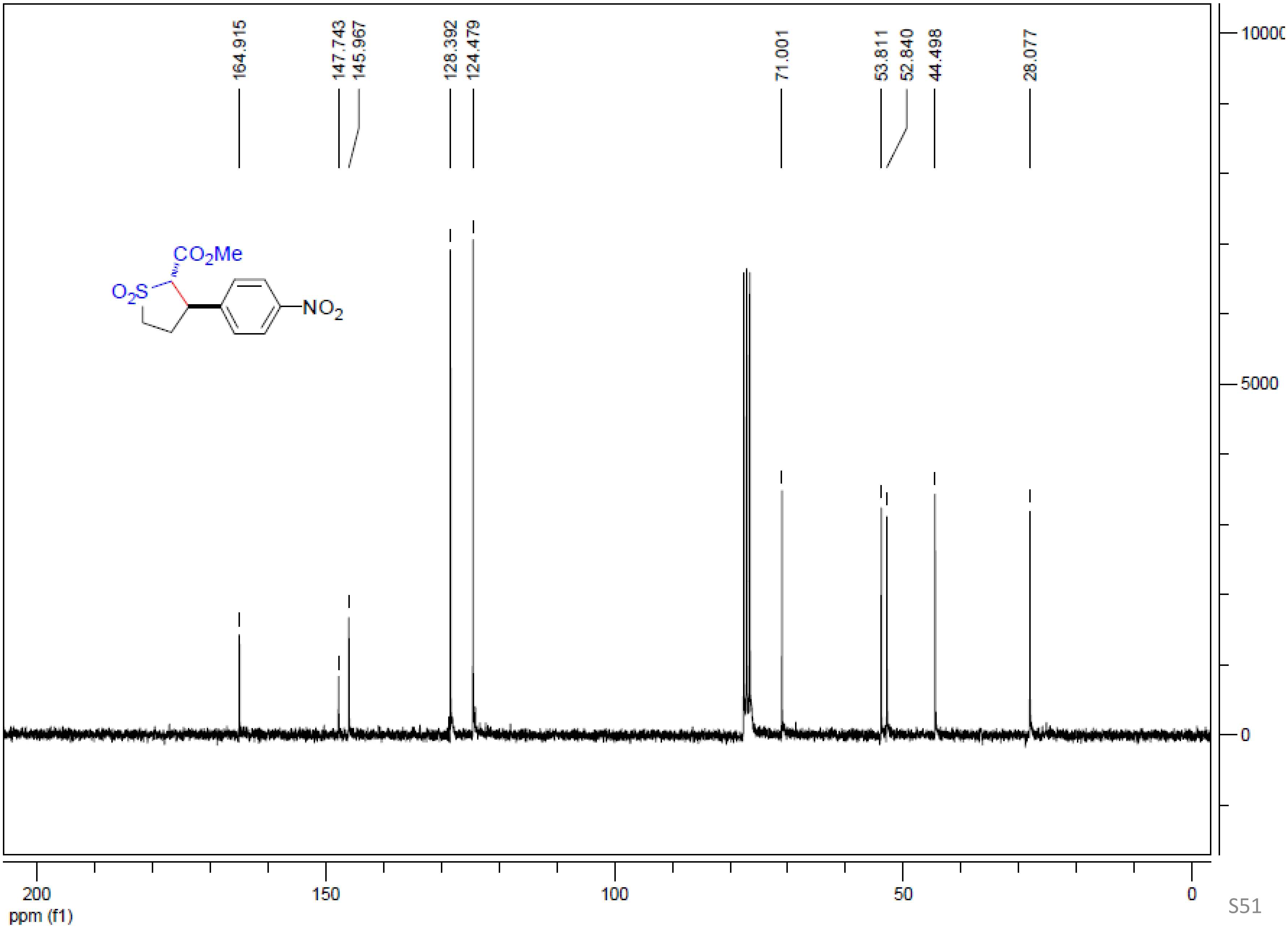

methyl 3-(4-nitrophenyl)tetrahydrothiophene-2-carboxylate 1,1-dioxide (**2a**)

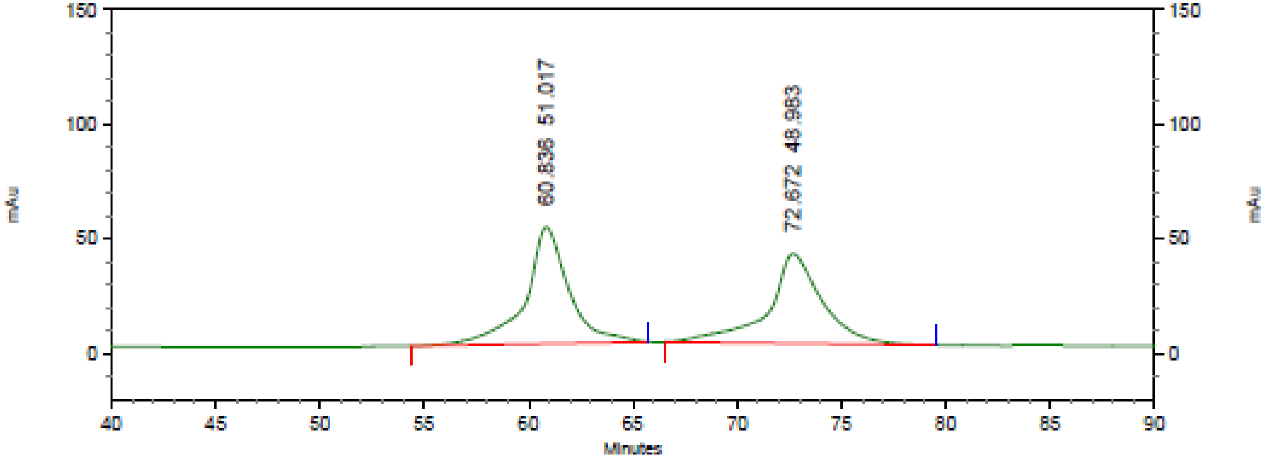

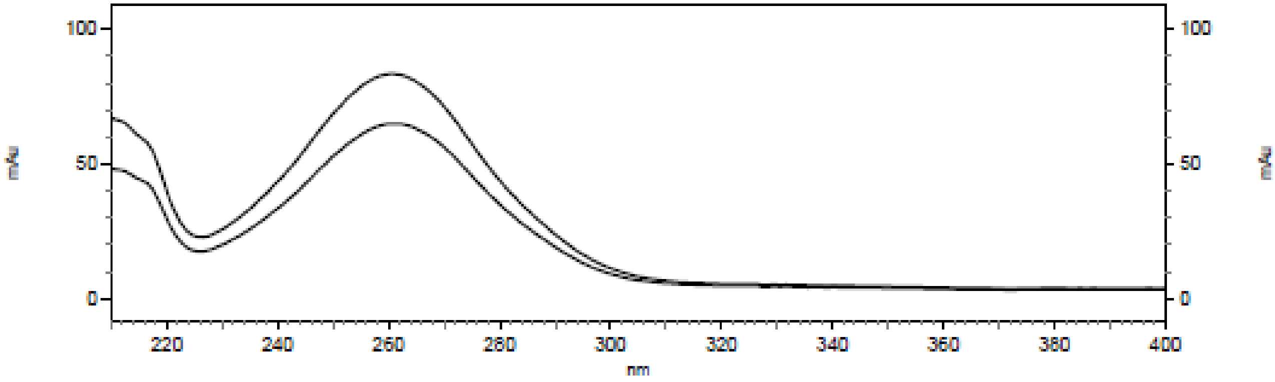

6: 268 nm, 4 nm Results

Pk #	Retention Time	Area Percent
1	0.168	0.000
2	60.836	51.017
3	72.672	48.983
Totals		100.000

methyl 3-(4-nitrophenyl)tetrahydrothiophene-2-carboxylate 1,1-dioxide (**2a**)

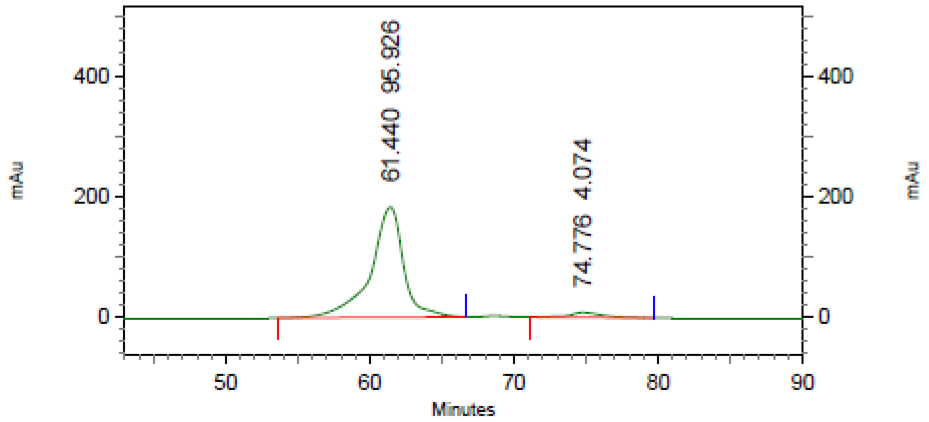

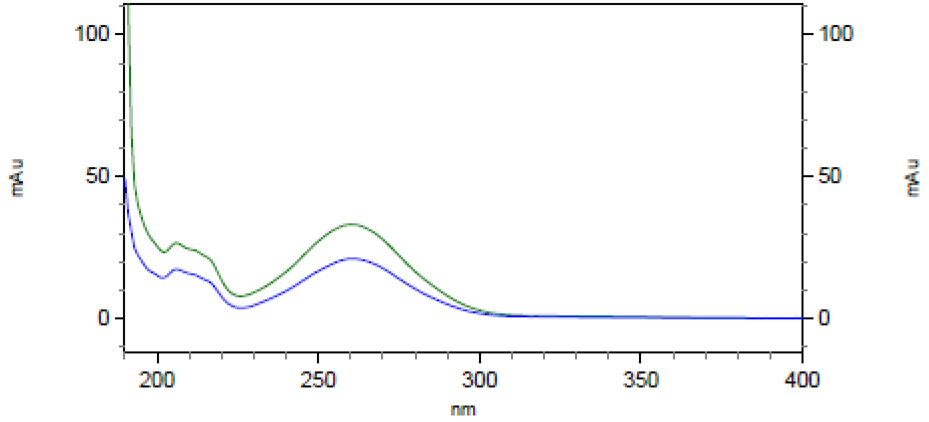

8: 280 nm, 4 nm

Results

Pk #	Name	Retention Time	Area Percent
1		61.440	95.926
2		74.776	4.074
Totals			100.000

methyl 3-(4-(trifluoromethyl)phenyl)tetrahydrothiophene-2-carboxylate 1,1-dioxide (**2b**)

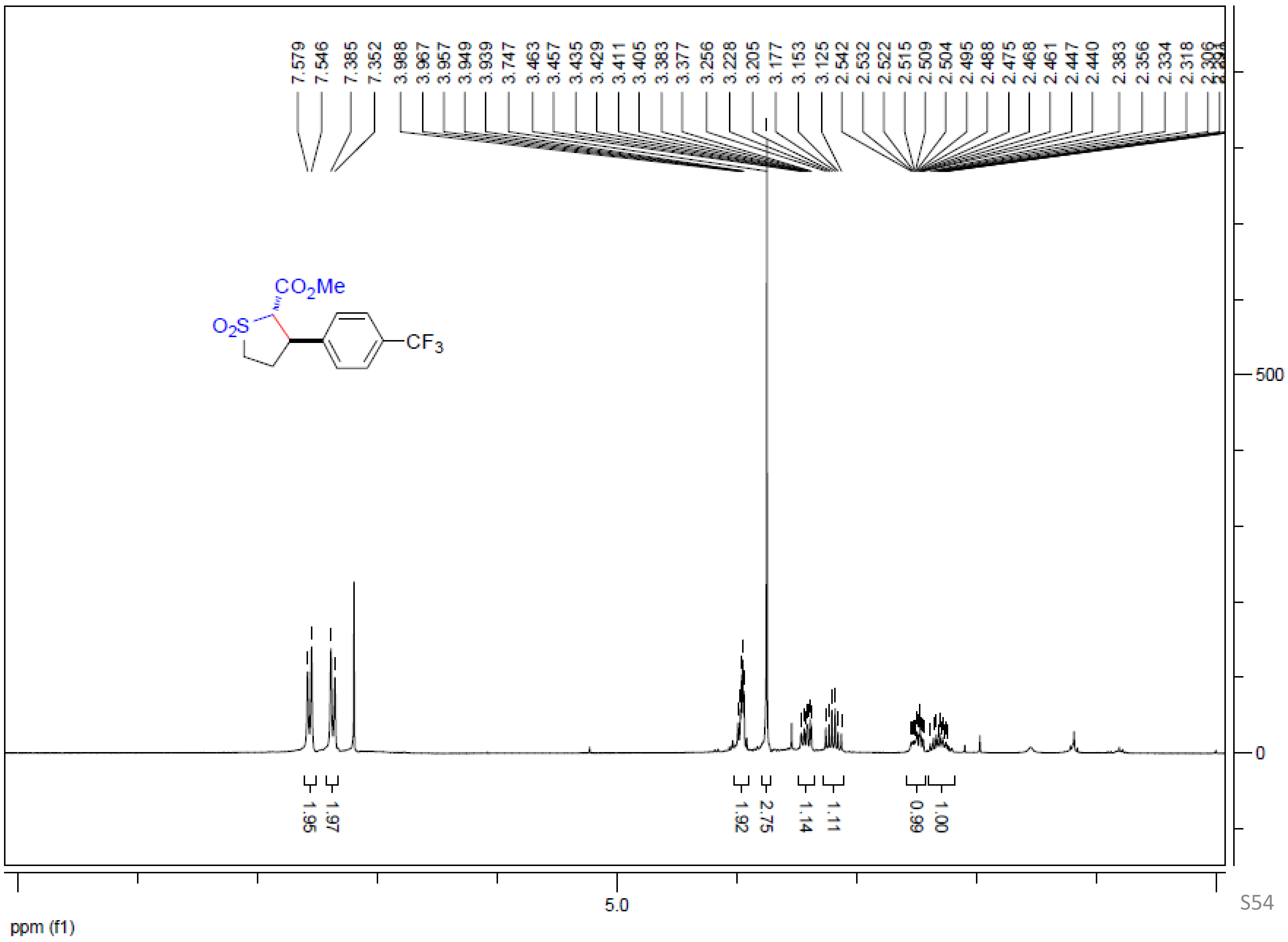

methyl 3-(4-(trifluoromethyl)phenyl)tetrahydrothiophene-2-carboxylate 1,1-dioxide (**2b**)

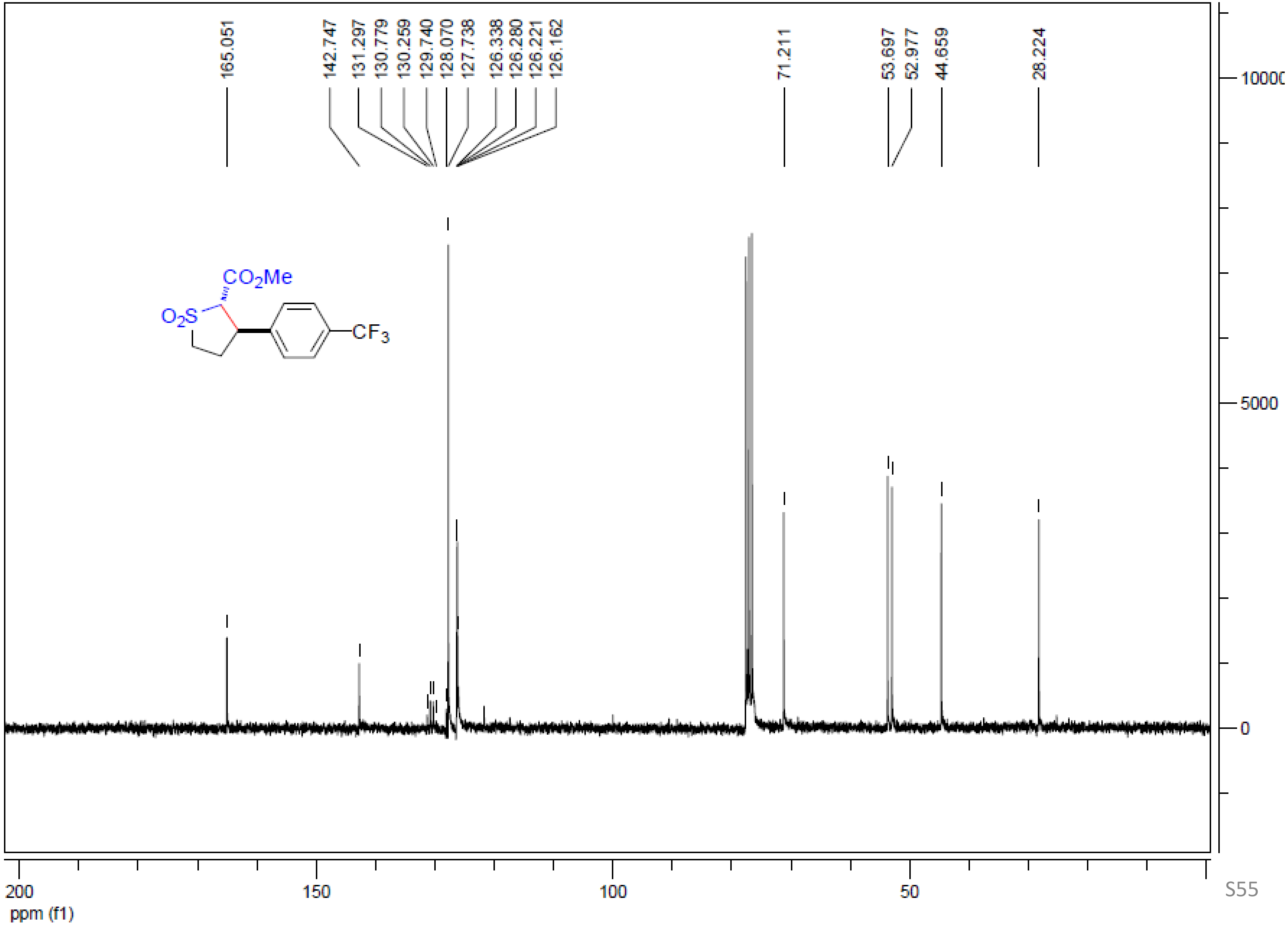

methyl 3-(4-(trifluoromethyl)phenyl)tetrahydrothiophene-2-carboxylate 1,1-dioxide (**2b**)

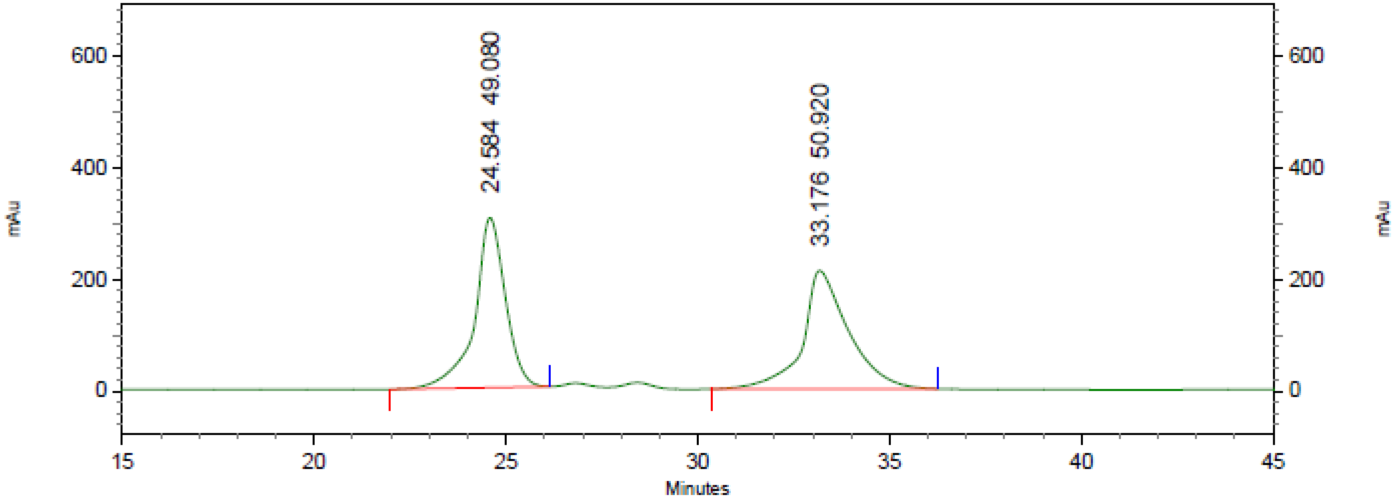

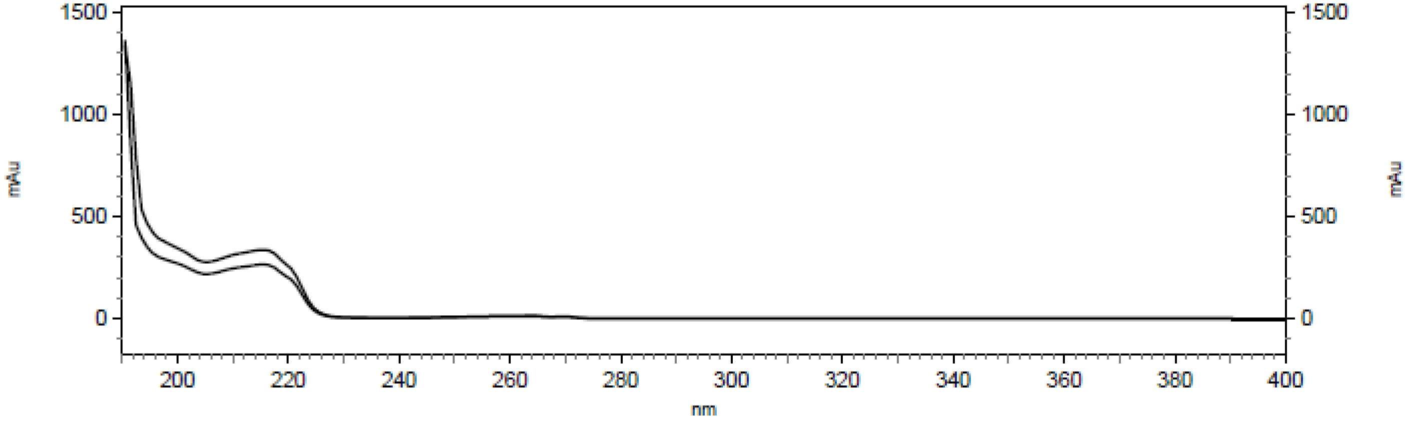

7: 221 nm, 4 nm Results

Pk #	Retention Time	Area Percent
1	24.584	49.080
2	33.176	50.920
Totals		100.000

methyl 3-(4-(trifluoromethyl)phenyl)tetrahydrothiophene-2-carboxylate 1,1-dioxide (**2b**)

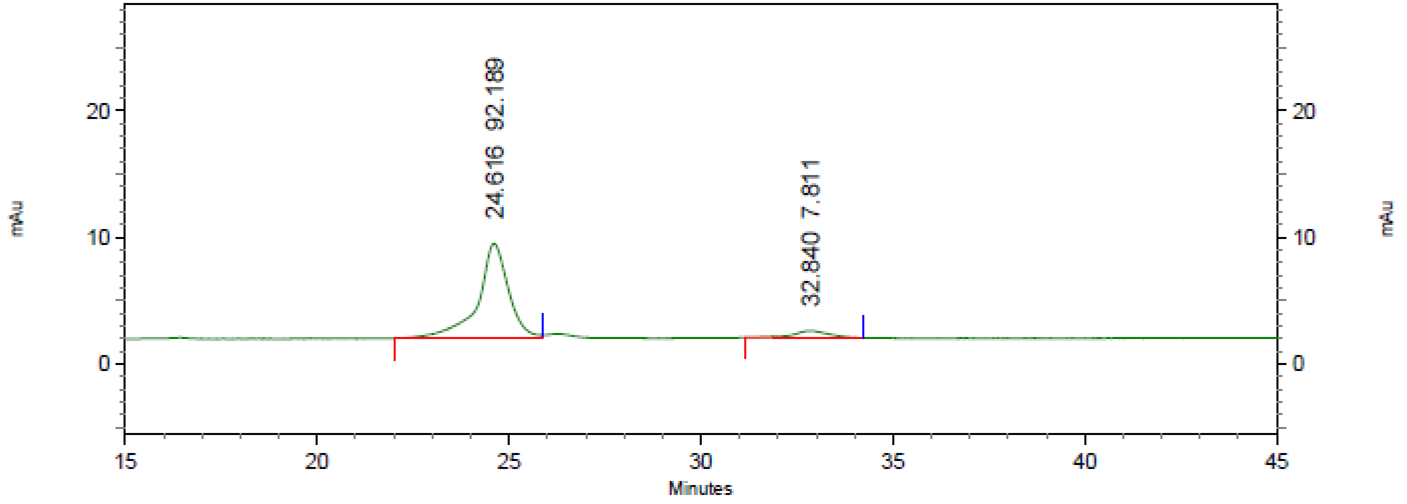

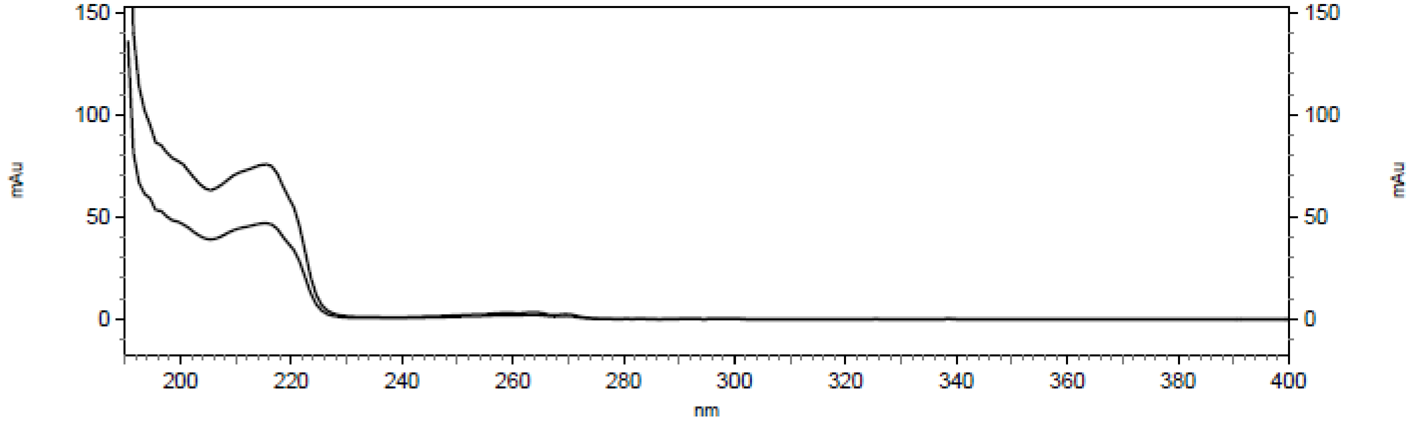

4: 260 nm, 4 nm Results

Pk #	Retention Time	Area Percent
1	24.616	92.189
2	32.840	7.811
Totals		100.000

methyl 3-(4-fluorophenyl)tetrahydrothiophene-2-carboxylate 1,1-dioxide (**2c**)

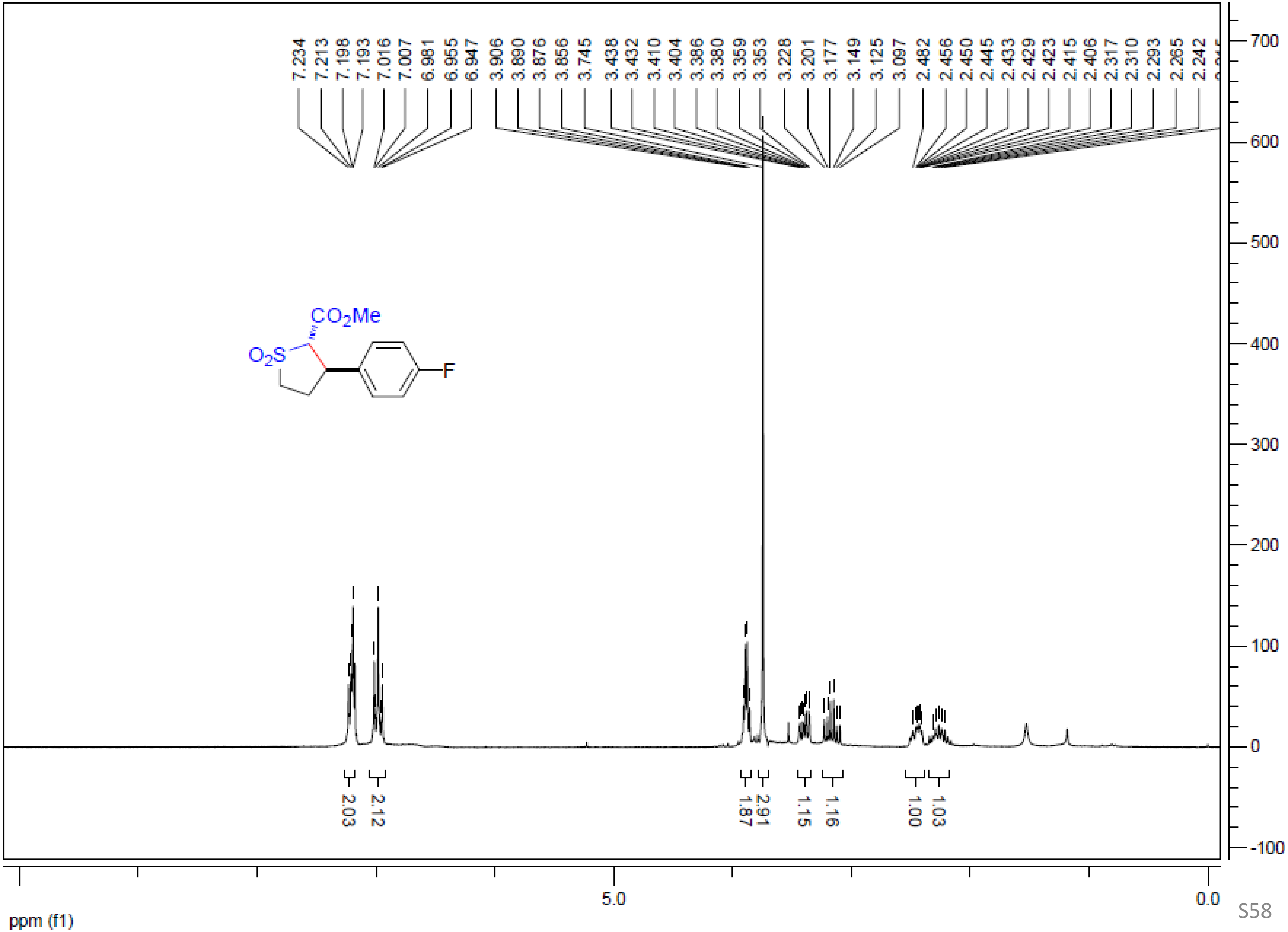

methyl 3-(4-fluorophenyl)tetrahydrothiophene-2-carboxylate 1,1-dioxide (**2c**)

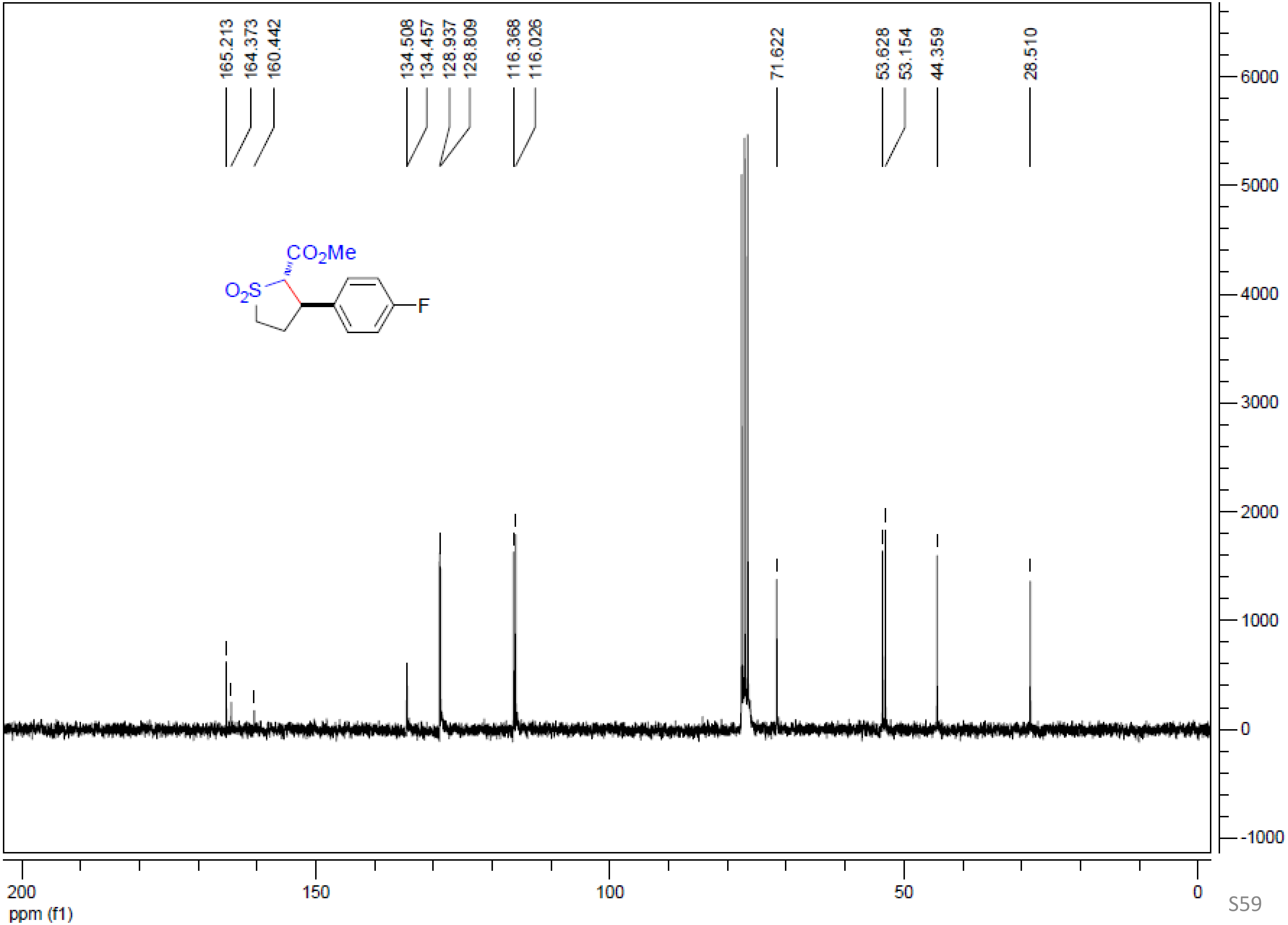

methyl 3-(4-fluorophenyl)tetrahydrothiophene-2-carboxylate 1,1-dioxide (**2c**)

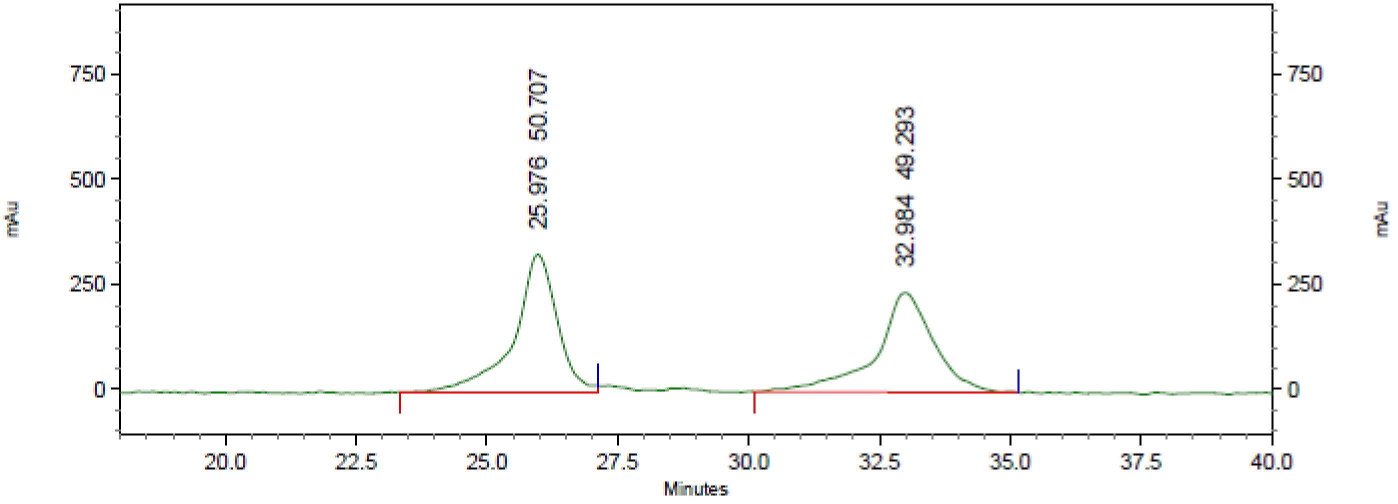

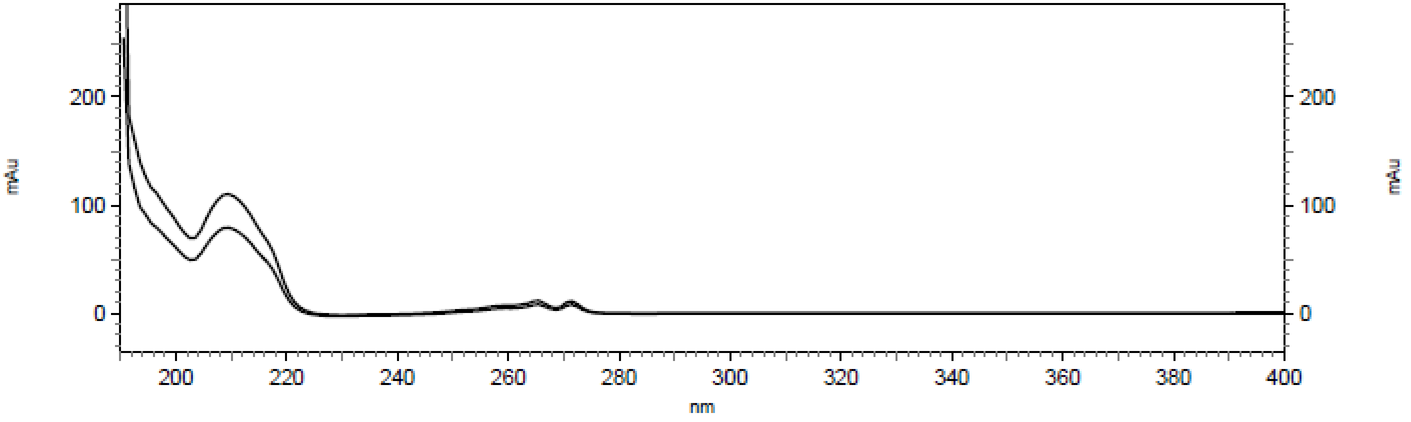

11: 210 nm, 4 nm Results

Pk #	Retention Time	Area Percent
1	25.976	50.707
2	32.984	49.293
Totals		100.000

methyl 3-(4-fluorophenyl)tetrahydrothiophene-2-carboxylate 1,1-dioxide (**2c**)

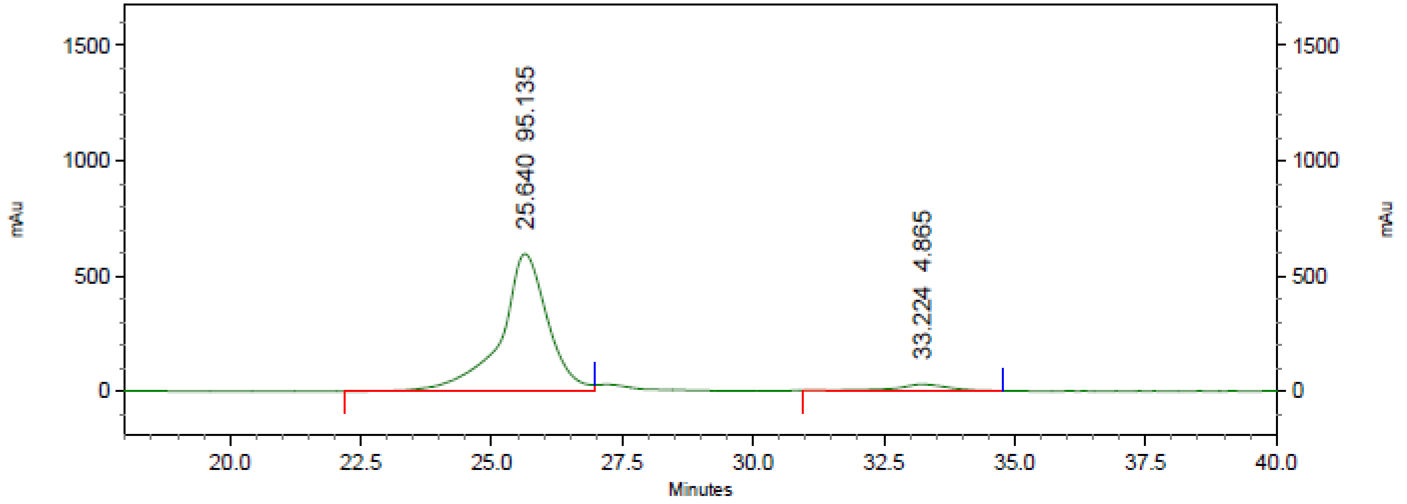

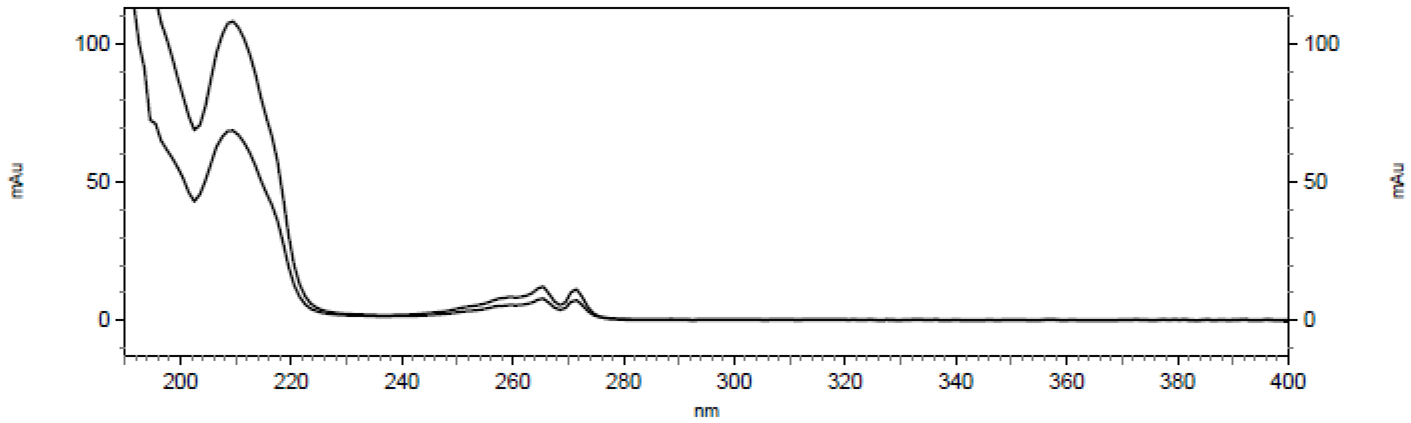

4: 212 nm, 4 nm Results

Pk #	Retention Time	Area Percent
1	25.640	95.135
2	33.224	4.865
Totals		100.000

methyl 3-(4-chlorophenyl)tetrahydrothiophene-2-carboxylate 1,1-dioxide (**2d**)

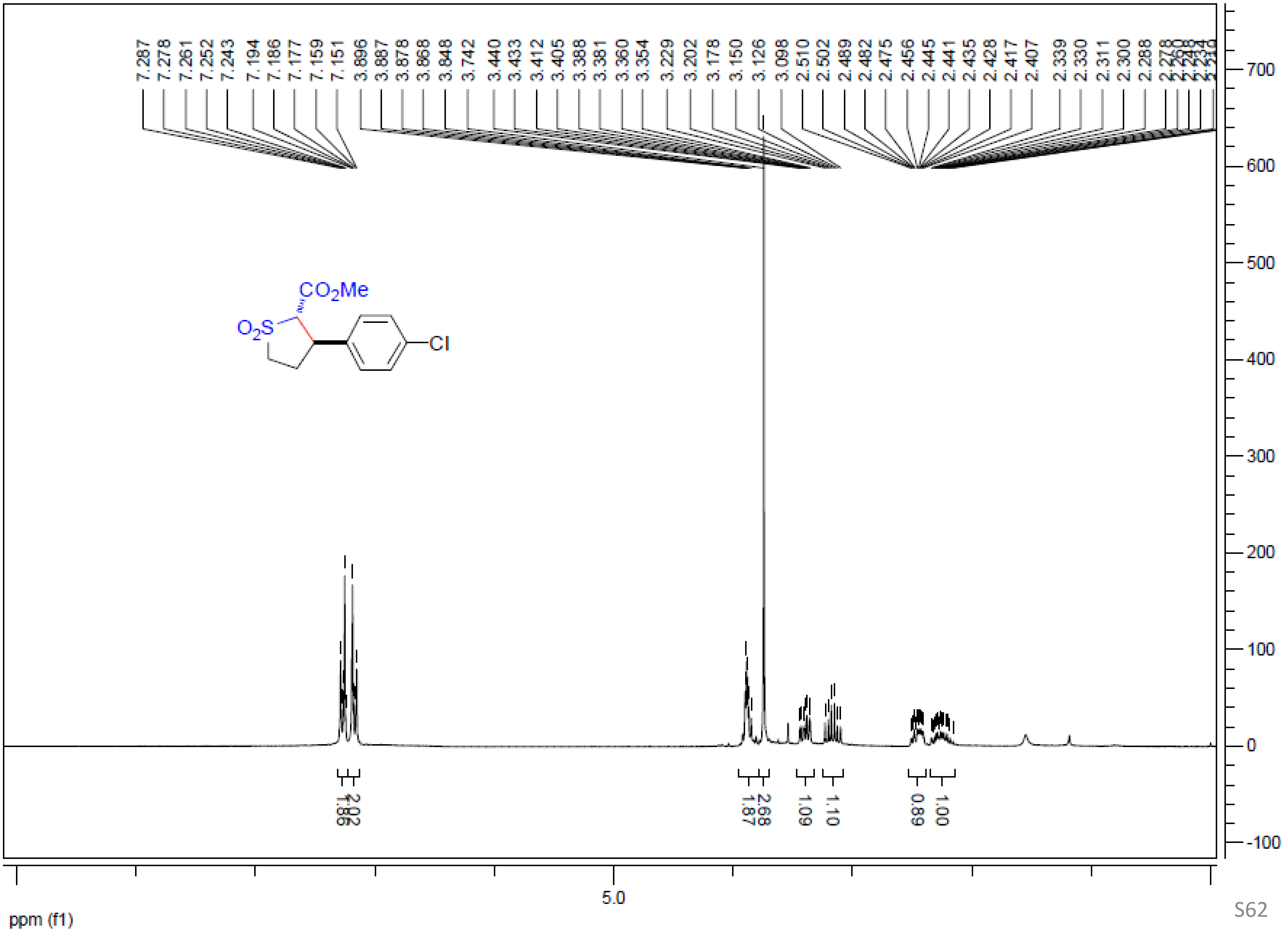

methyl 3-(4-chlorophenyl)tetrahydrothiophene-2-carboxylate 1,1-dioxide (**2d**)

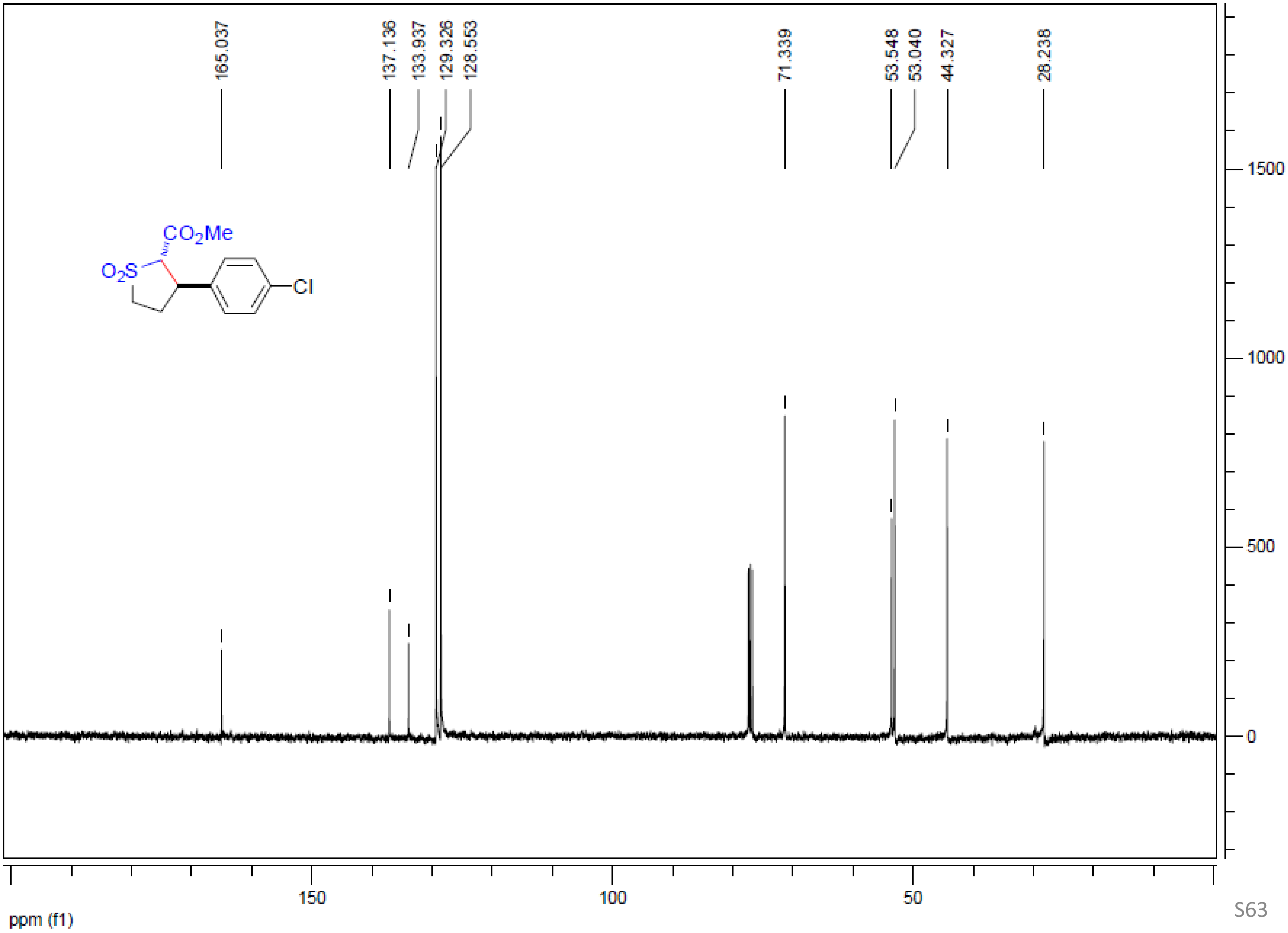

methyl 3-(4-chlorophenyl)tetrahydrothiophene-2-carboxylate 1,1-dioxide (**2d**)

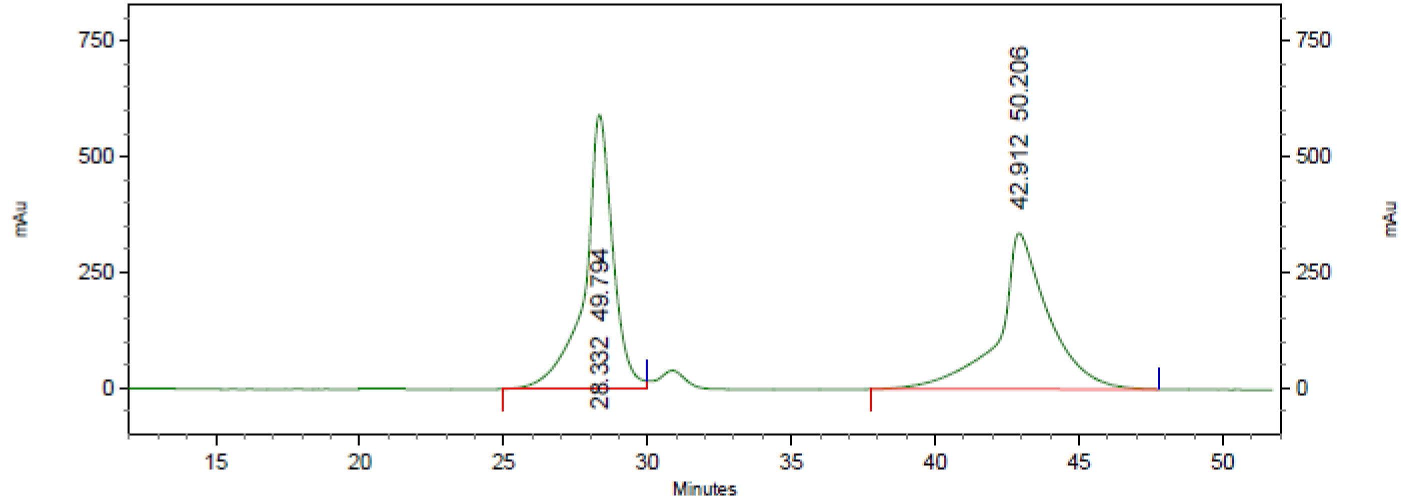

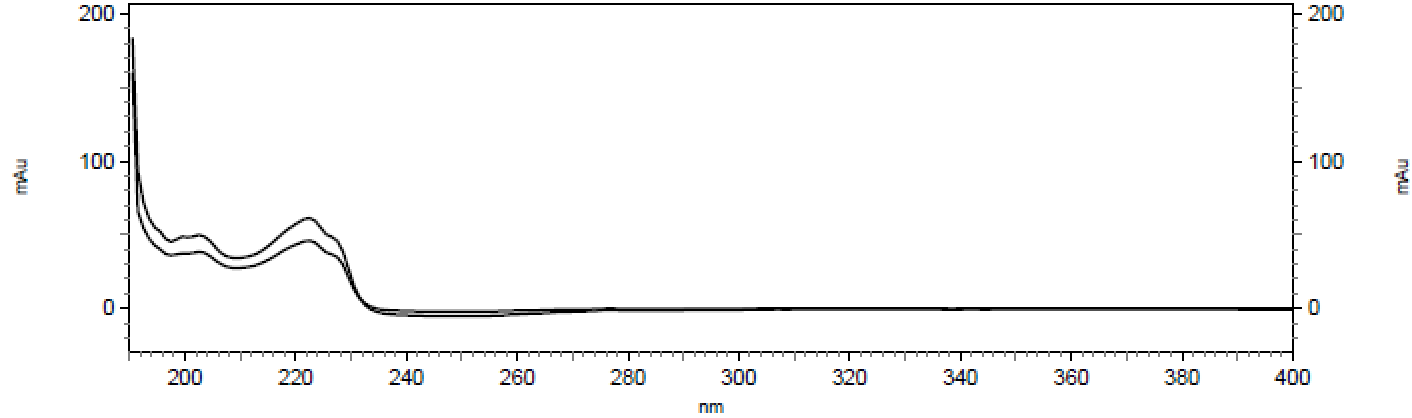

11: 223 nm, 4 nm Results

Pk #	Retention Time	Area Percent
1	28.332	49.794
2	42.912	50.206
Totals		100.000

methyl 3-(4-chlorophenyl)tetrahydrothiophene-2-carboxylate 1,1-dioxide (**2d**)

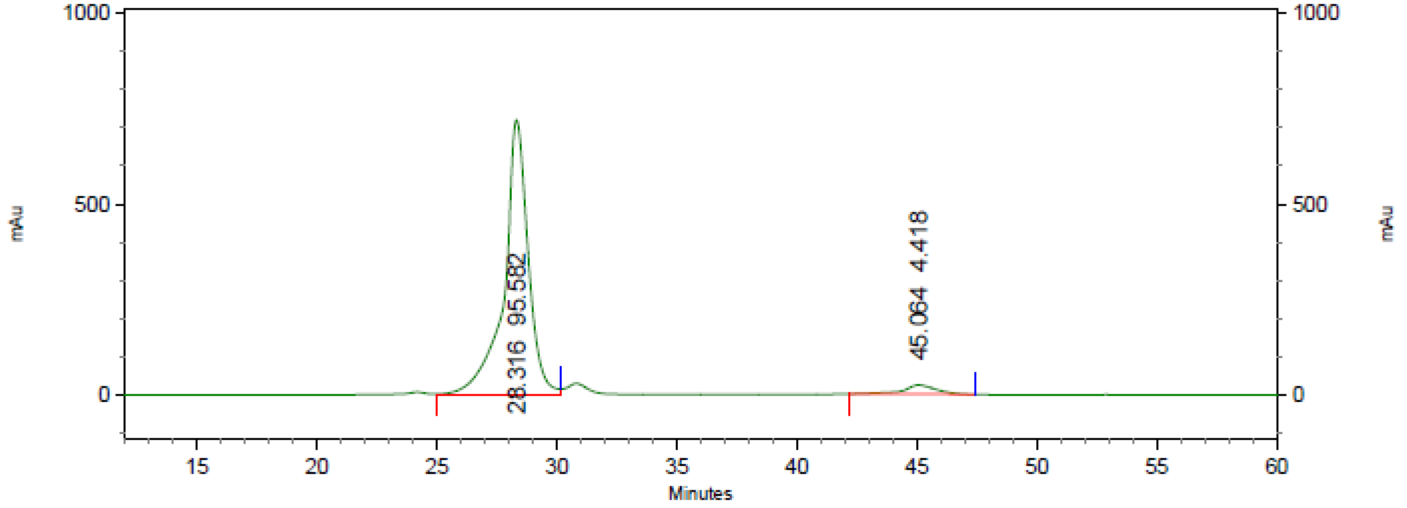

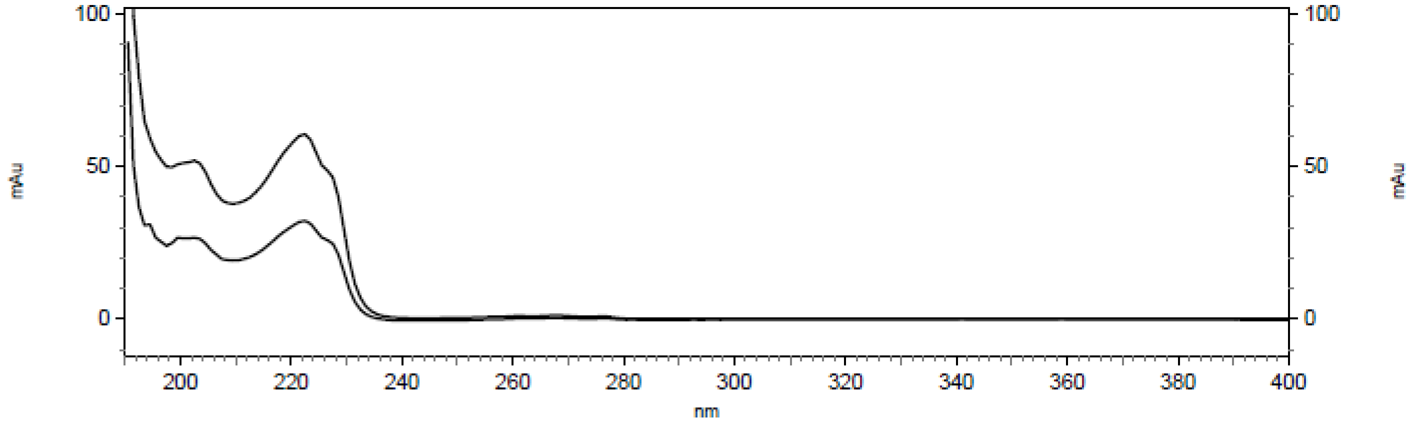

11: 223 nm, 4 nm Results

Pk #	Retention Time	Area Percent
1	28.316	95.582
2	45.064	4.418
Totals		100.000

methyl 3-(3,5-dichlorophenyl)tetrahydrothiophene-2-carboxylate 1,1-dioxide (**2e**)

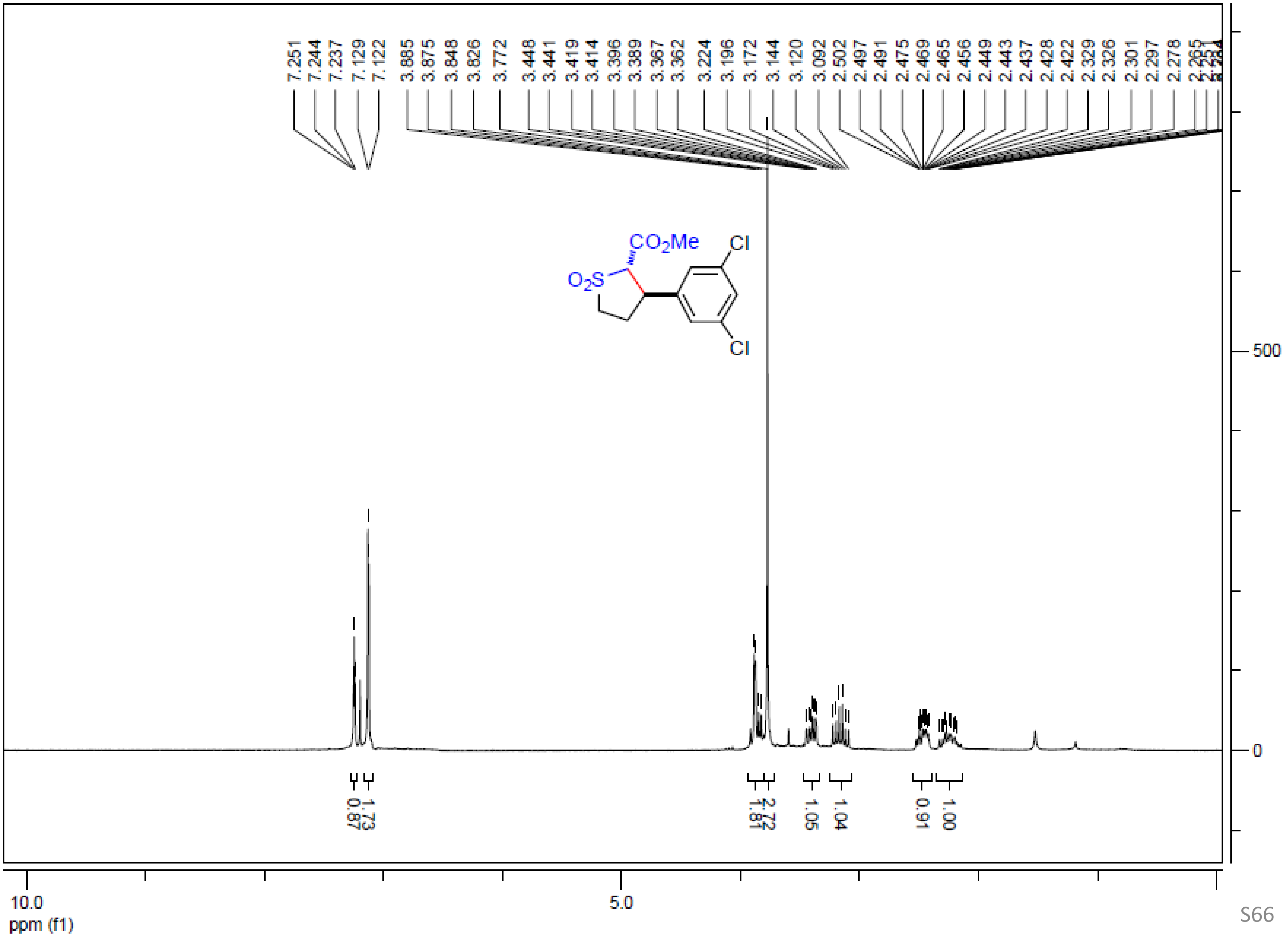

methyl 3-(3,5-dichlorophenyl)tetrahydrothiophene-2-carboxylate 1,1-dioxide (**2e**)

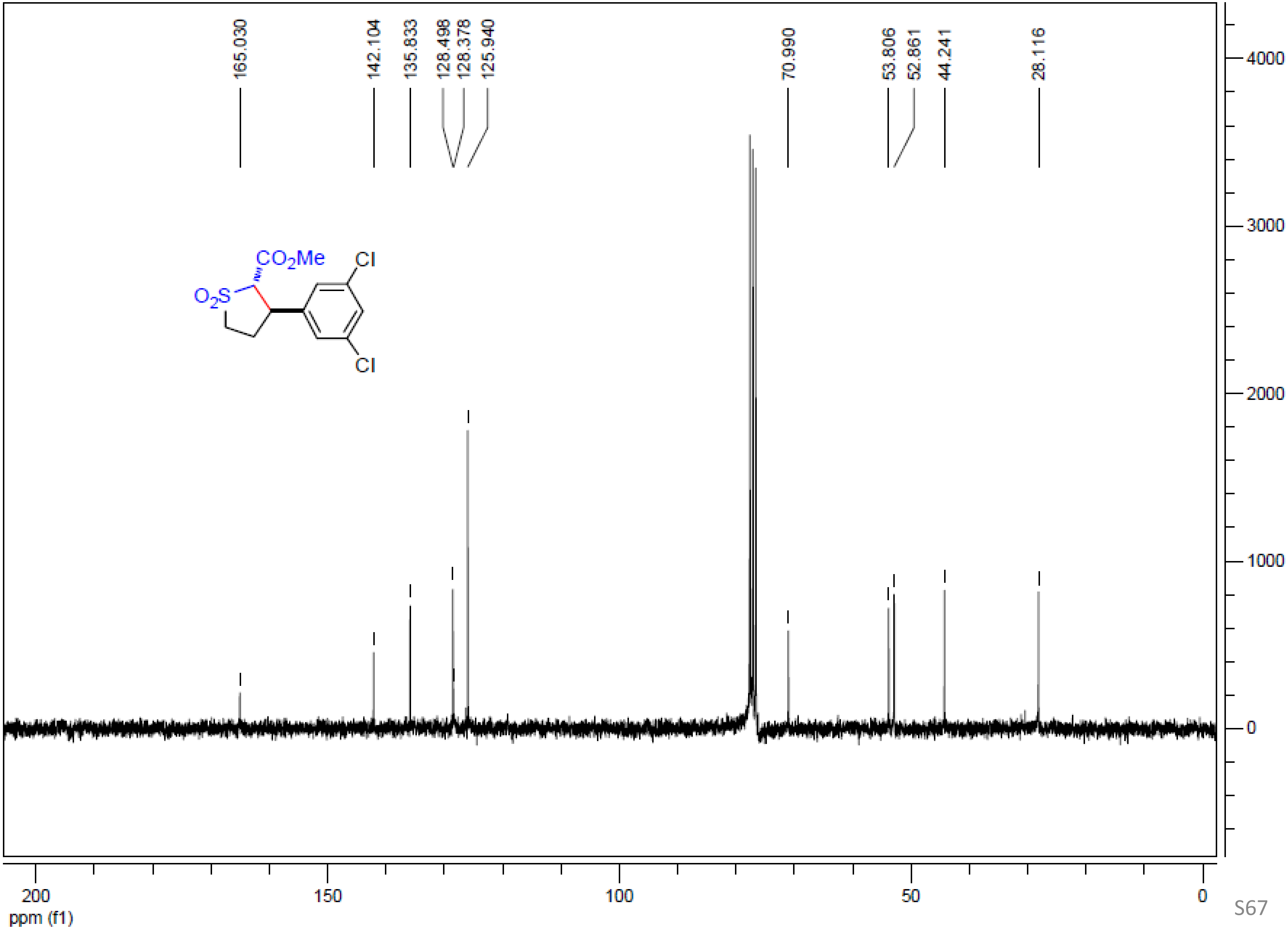

methyl 3-(3,5-dichlorophenyl)tetrahydrothiophene-2-carboxylate 1,1-dioxide (**2e**)

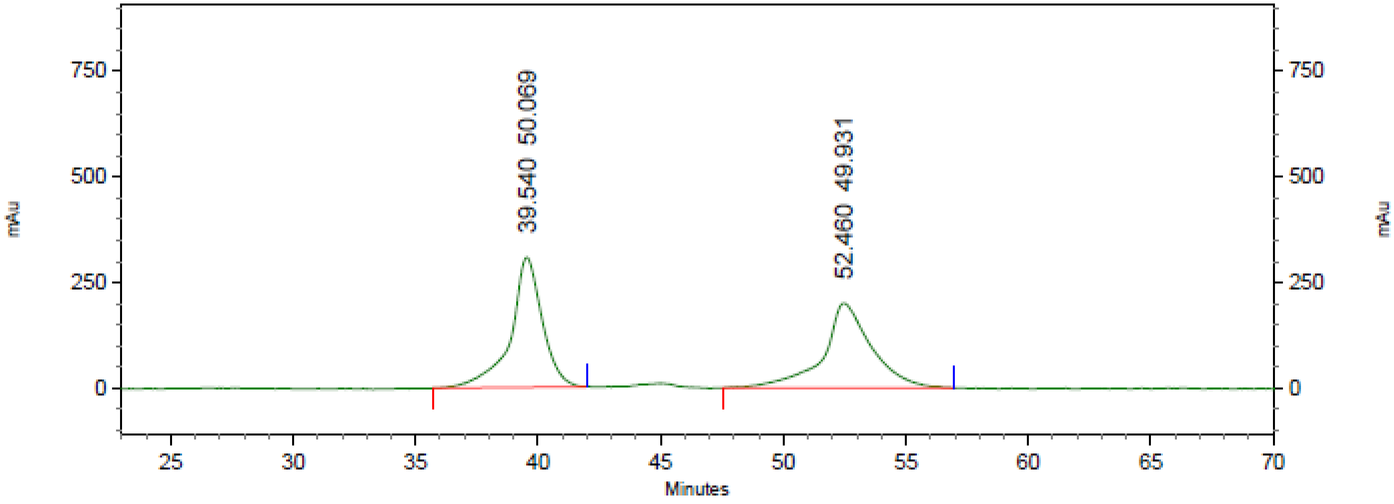

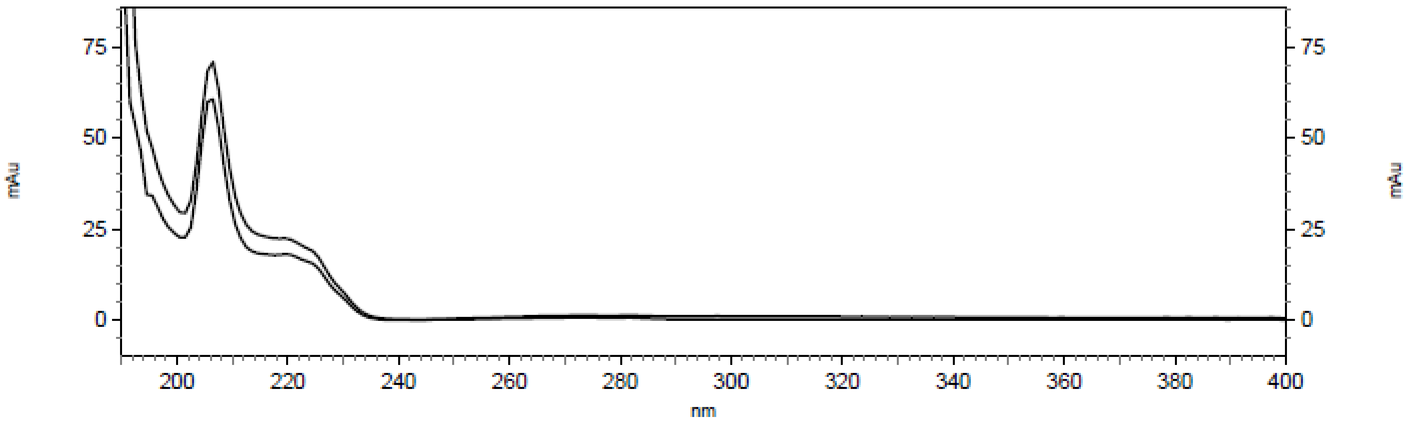

9: 210 nm, 4 nm Results

Pk #	Retention Time	Area Percent
1	39.540	50.069
2	52.460	49.931
Totals		100.000

methyl 3-(3,5-dichlorophenyl)tetrahydrothiophene-2-carboxylate 1,1-dioxide (**2e**)

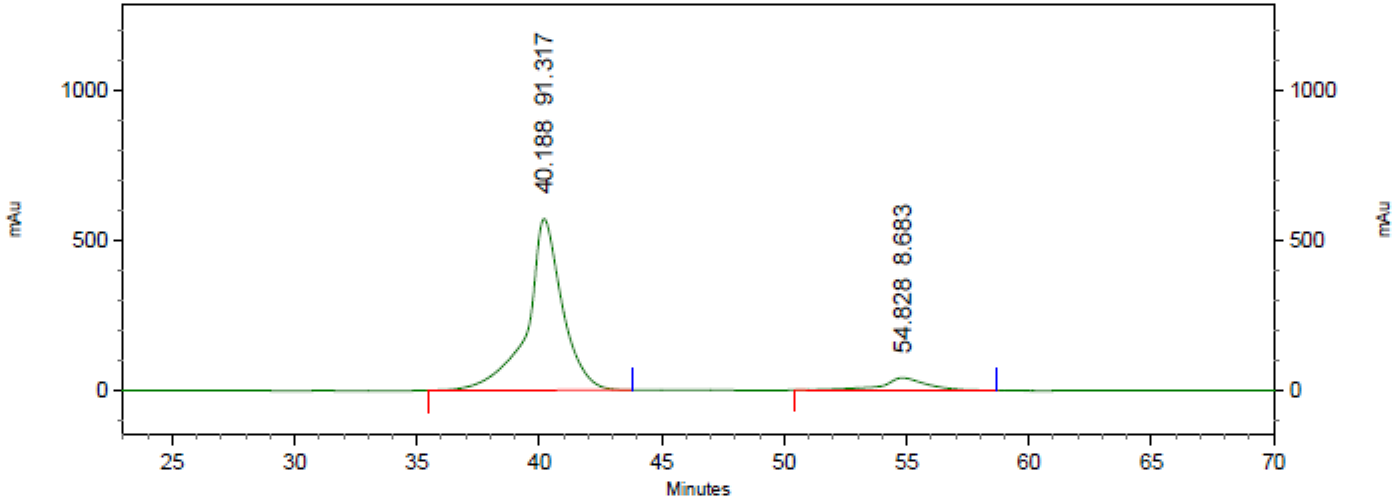

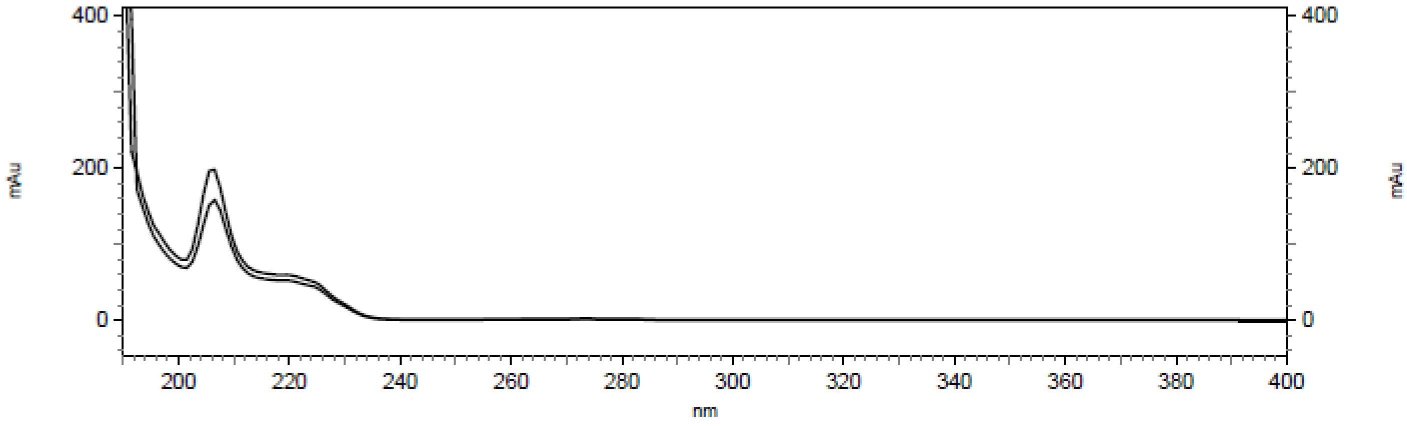

9: 210 nm, 4 nm Results

Pk #	Retention Time	Area Percent
1	40.188	91.317
2	54.828	8.683
Totals		100.000

methyl 3-(3-bromophenyl)tetrahydrothiophene-2-carboxylate 1,1-dioxide (**2f**)

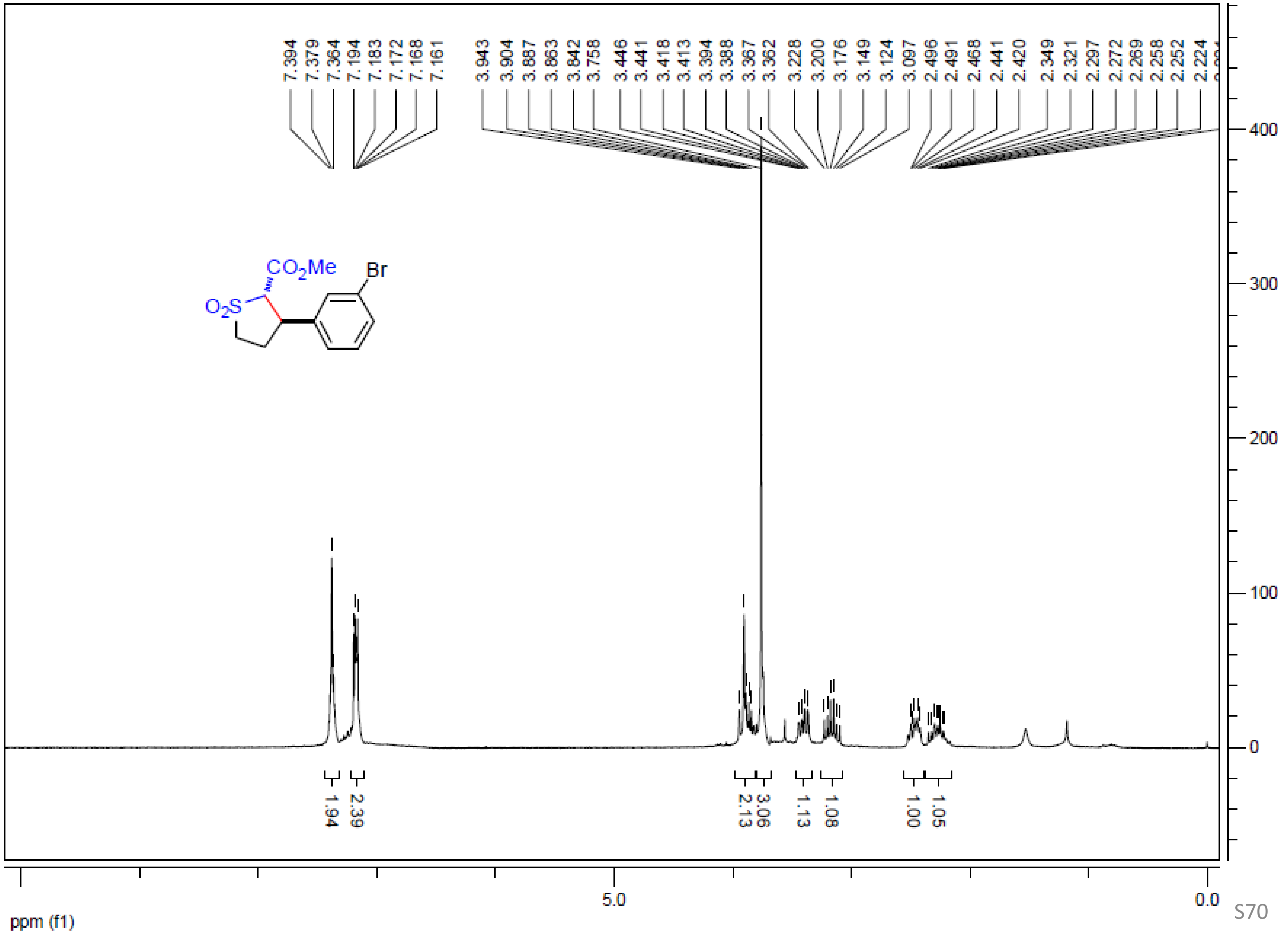

methyl 3-(3-bromophenyl)tetrahydrothiophene-2-carboxylate 1,1-dioxide (**2f**)

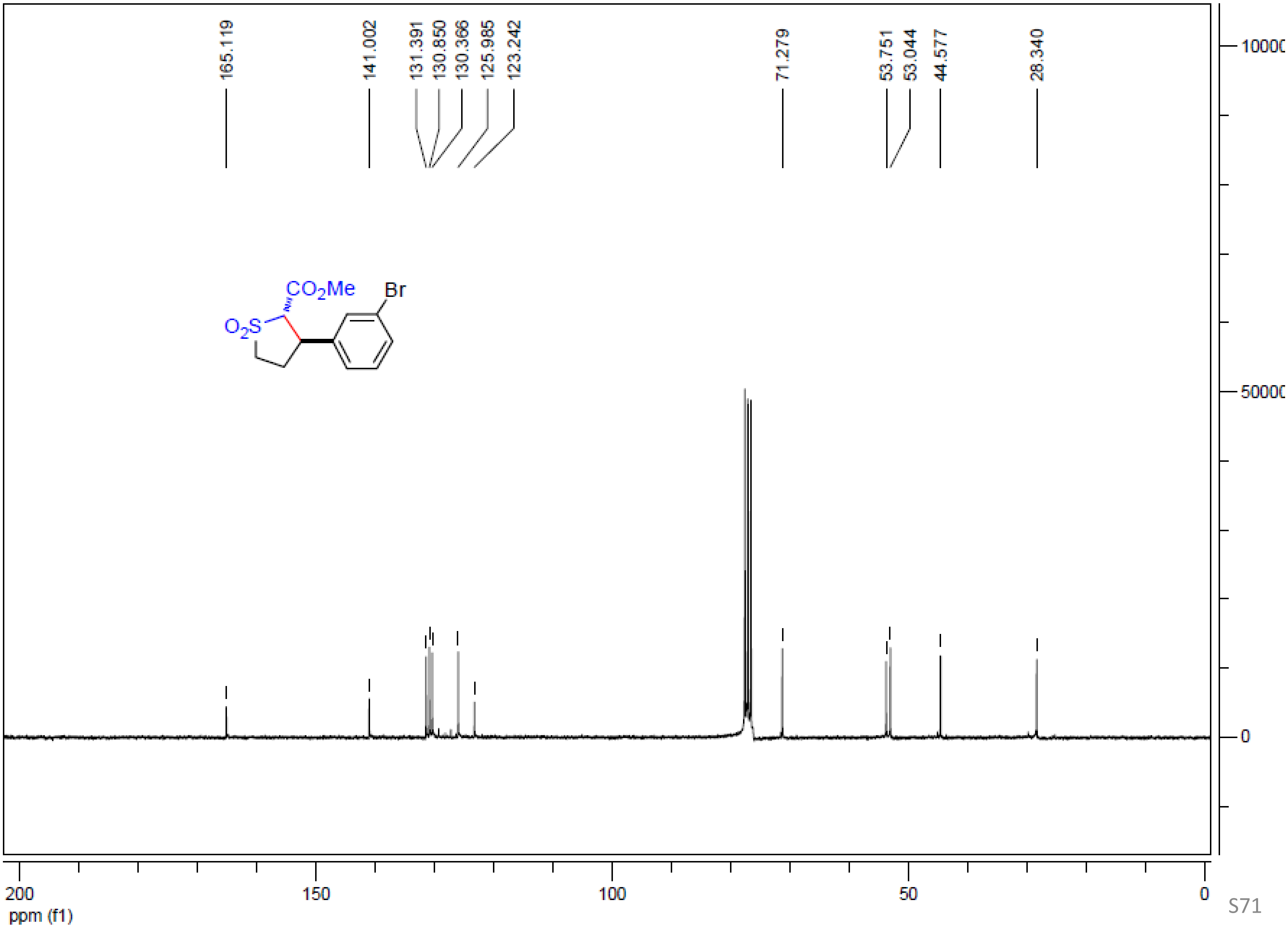

methyl 3-(3-bromophenyl)tetrahydrothiophene-2-carboxylate 1,1-dioxide (**2f**)

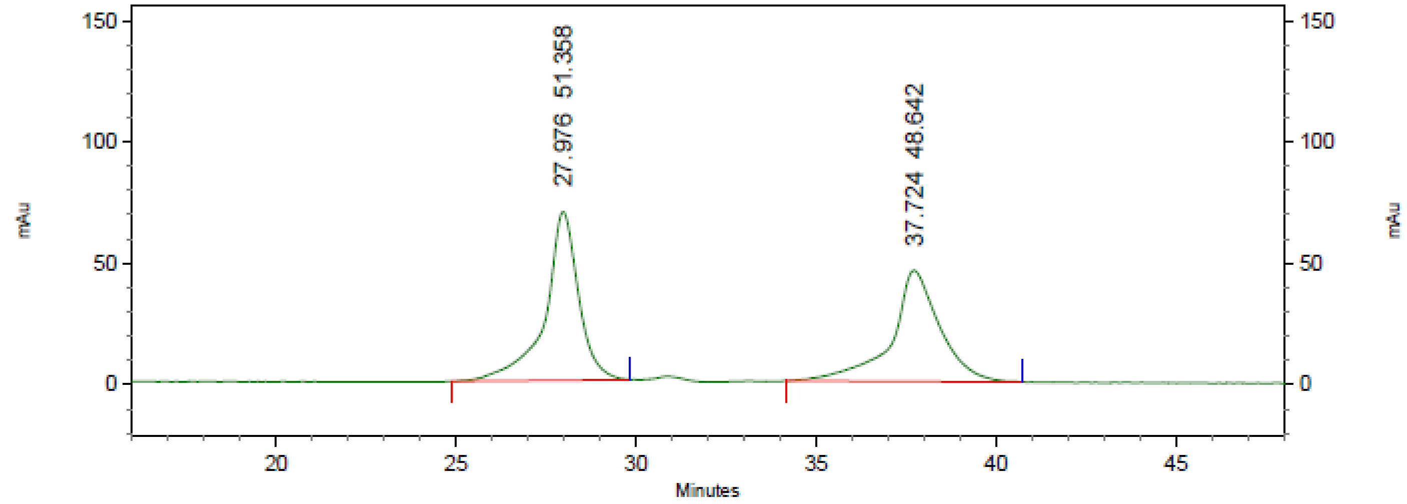

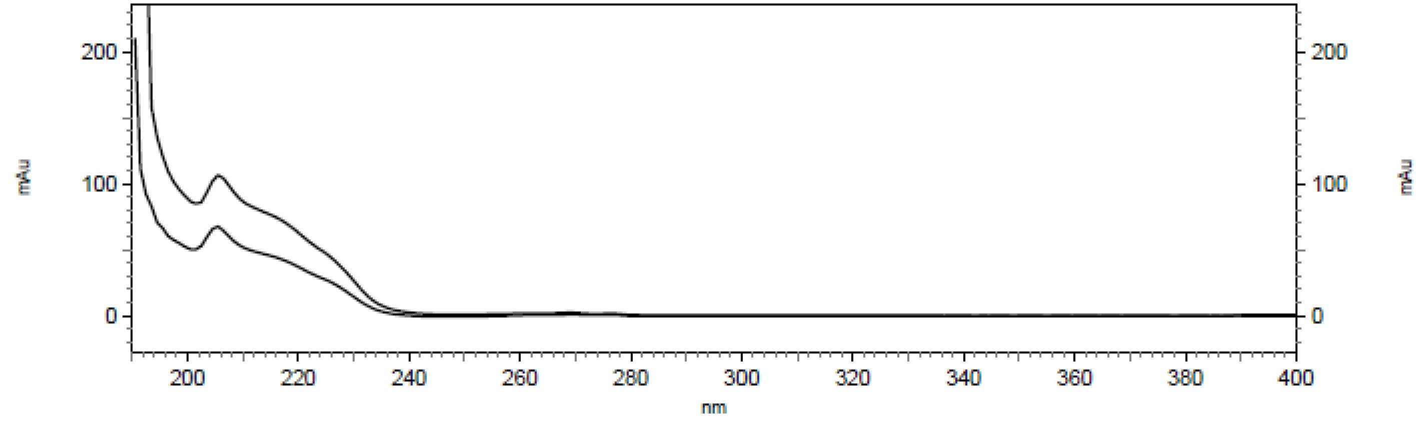

11: 226 nm, 4 nm Results

Pk #	Retention Time	Area Percent
1	27.976	51.358
2	37.724	48.642
Totals		100.000

methyl 3-(3-bromophenyl)tetrahydrothiophene-2-carboxylate 1,1-dioxide (**2f**)

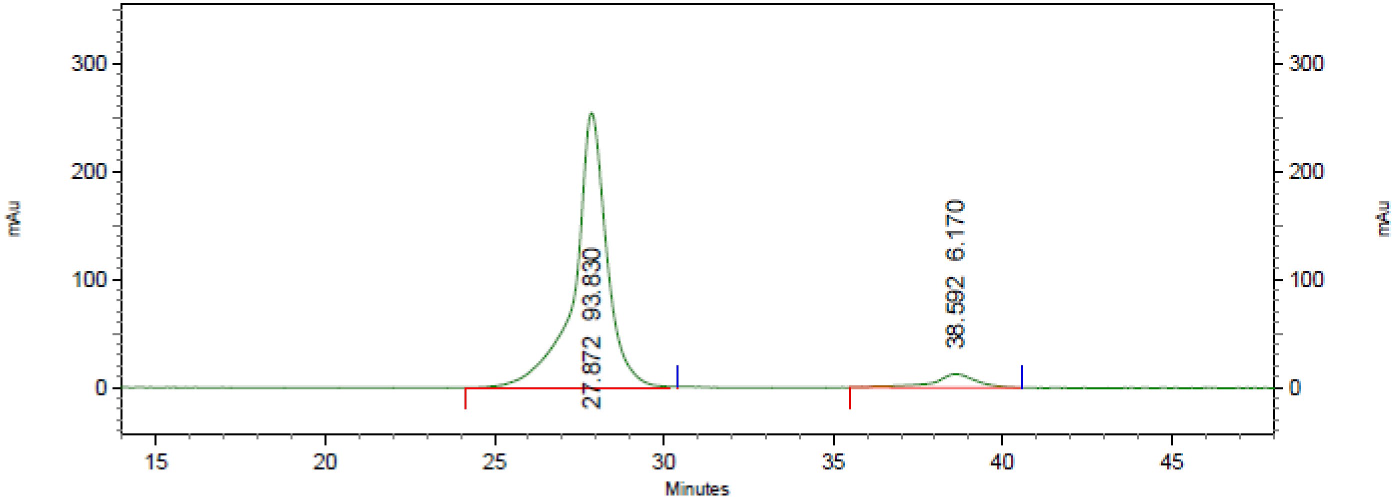

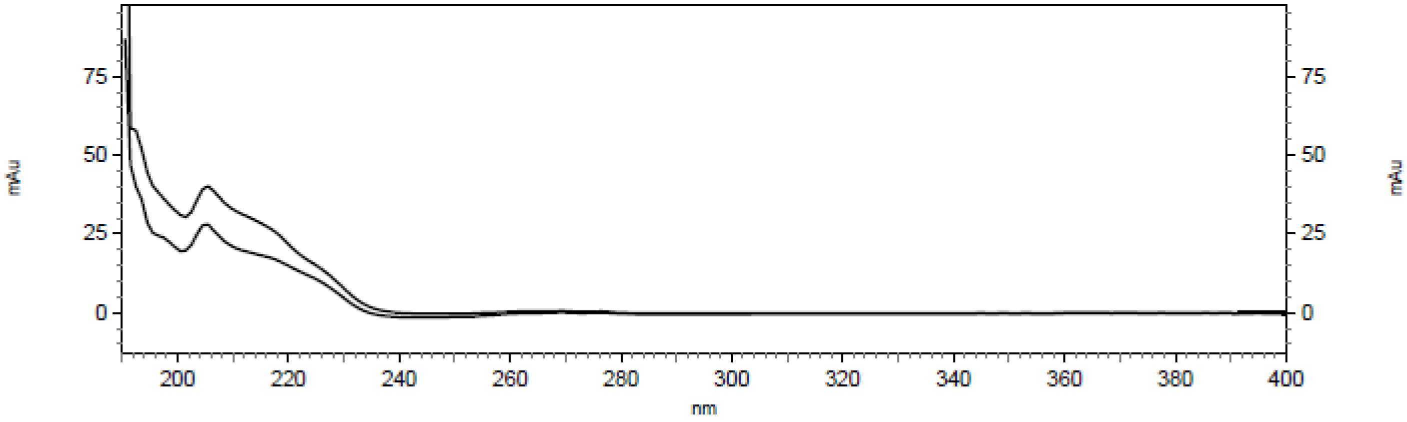

11: 223 nm, 4 nm Results

Pk #	Retention Time	Area Percent
1	27.872	93.830
2	38.592	6.170
Totals		100.000

(2S,3R)-methyl 3-phenyltetrahydrothiophene-2-carboxylate 1,1-dioxide (**2g**)

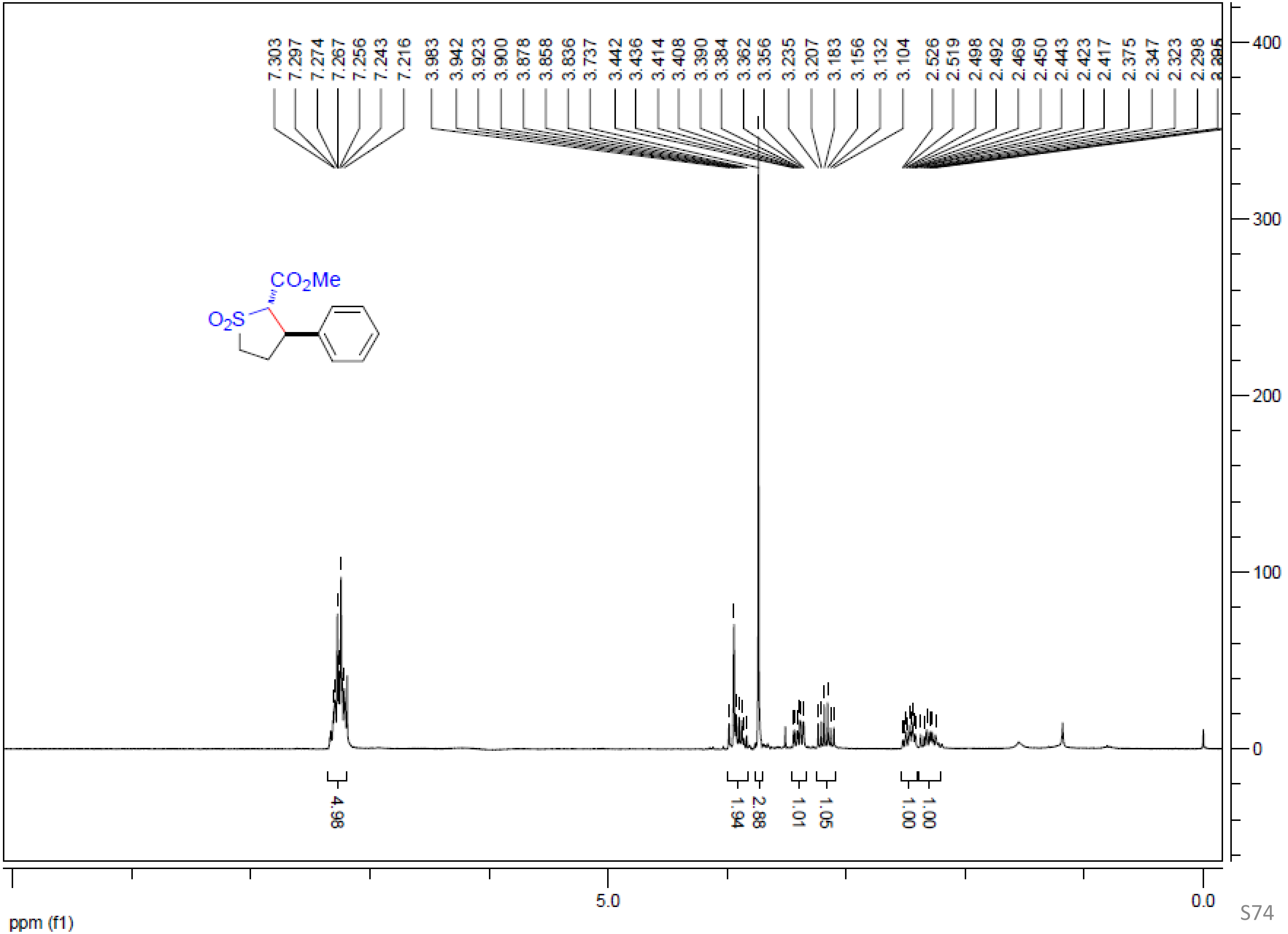

(2S,3R)-methyl 3-phenyltetrahydrothiophene-2-carboxylate 1,1-dioxide (**2g**)

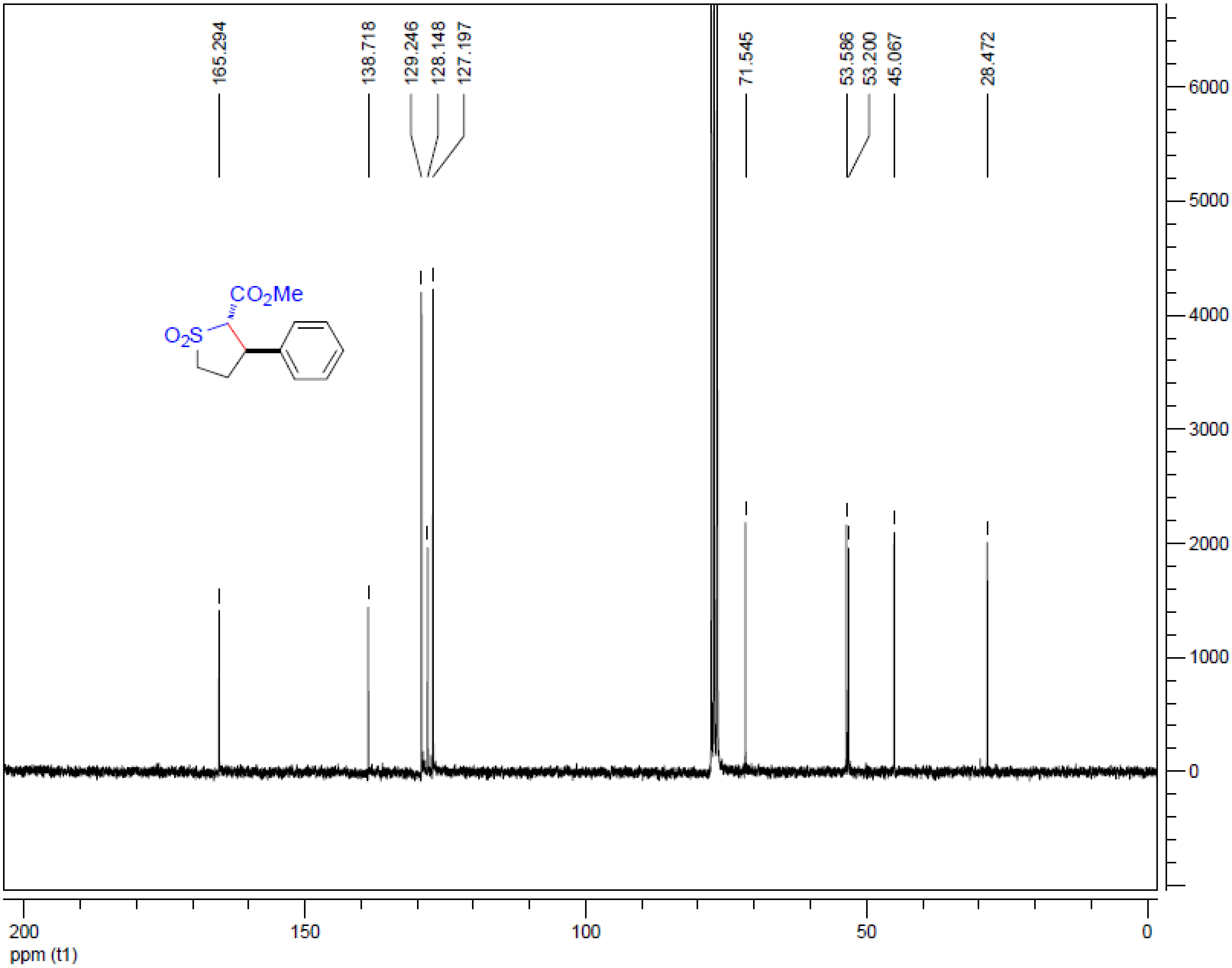

(2S,3R)-methyl 3-phenyltetrahydrothiophene-2-carboxylate 1,1-dioxide (**2g**)

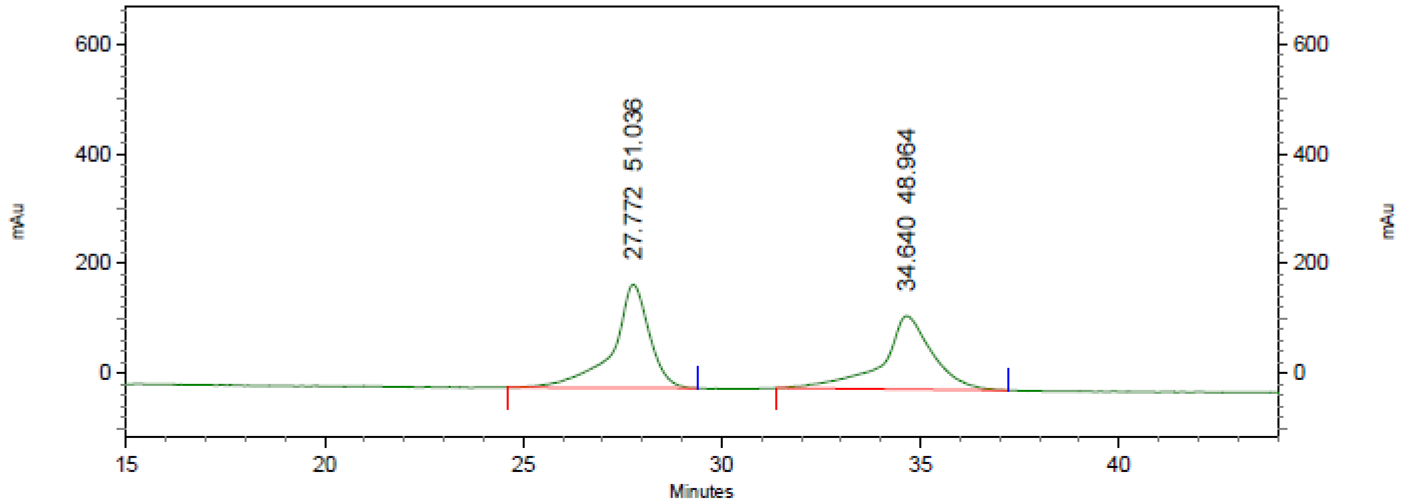

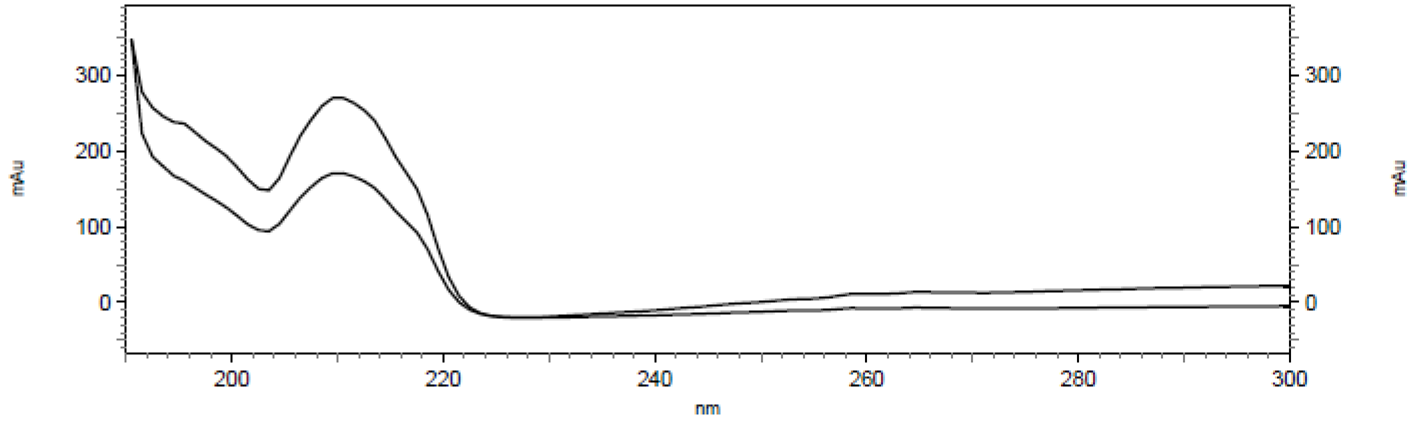

4: 215 nm, 4 nm Results

Pk #	Retention Time	Area Percent
1	27.772	51.036
2	34.640	48.964
Totals		100.000

(2S,3R)-methyl 3-phenyltetrahydrothiophene-2-carboxylate 1,1-dioxide (**2g**)

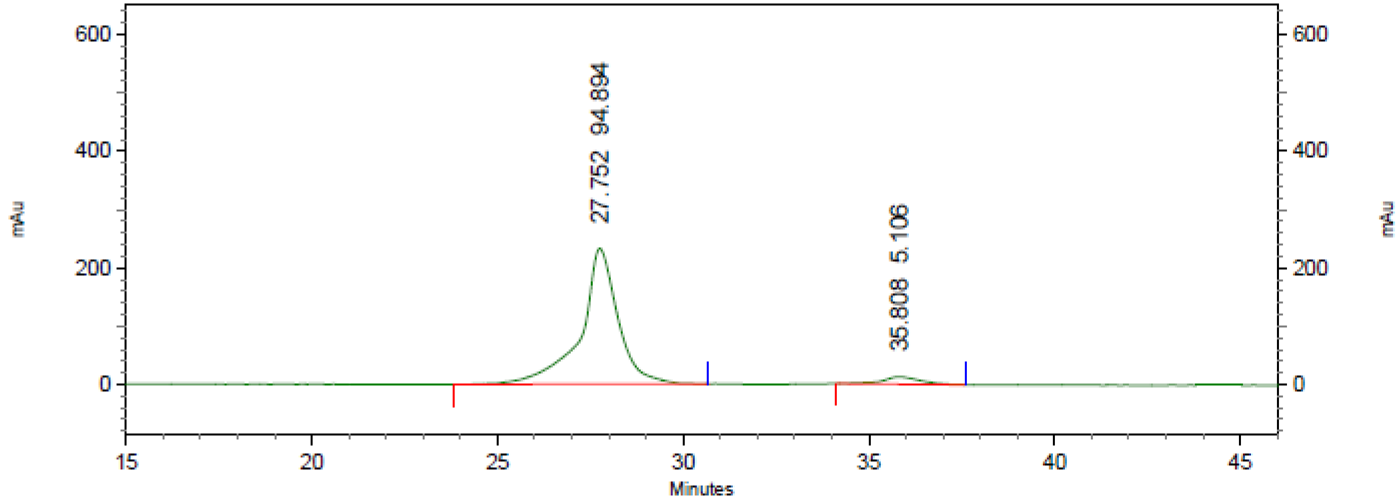

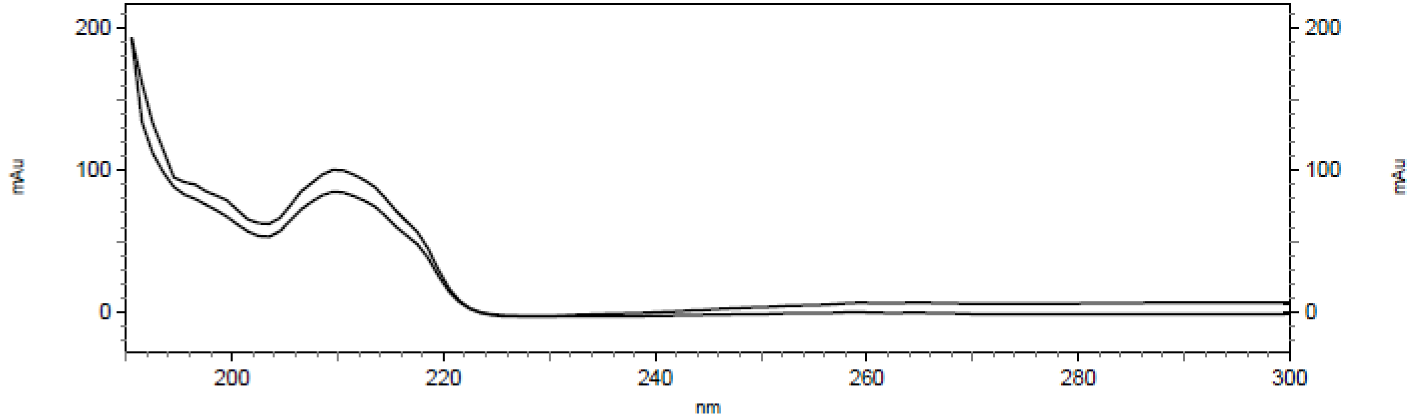

4: 215 nm, 4 nm Results

Pk #	Retention Time	Area Percent
1	27.752	94.894
2	35.808	5.106
Totals		100.000

methyl 3-(p-tolyl)tetrahydrothiophene-2-carboxylate 1,1-dioxide (**2h**)

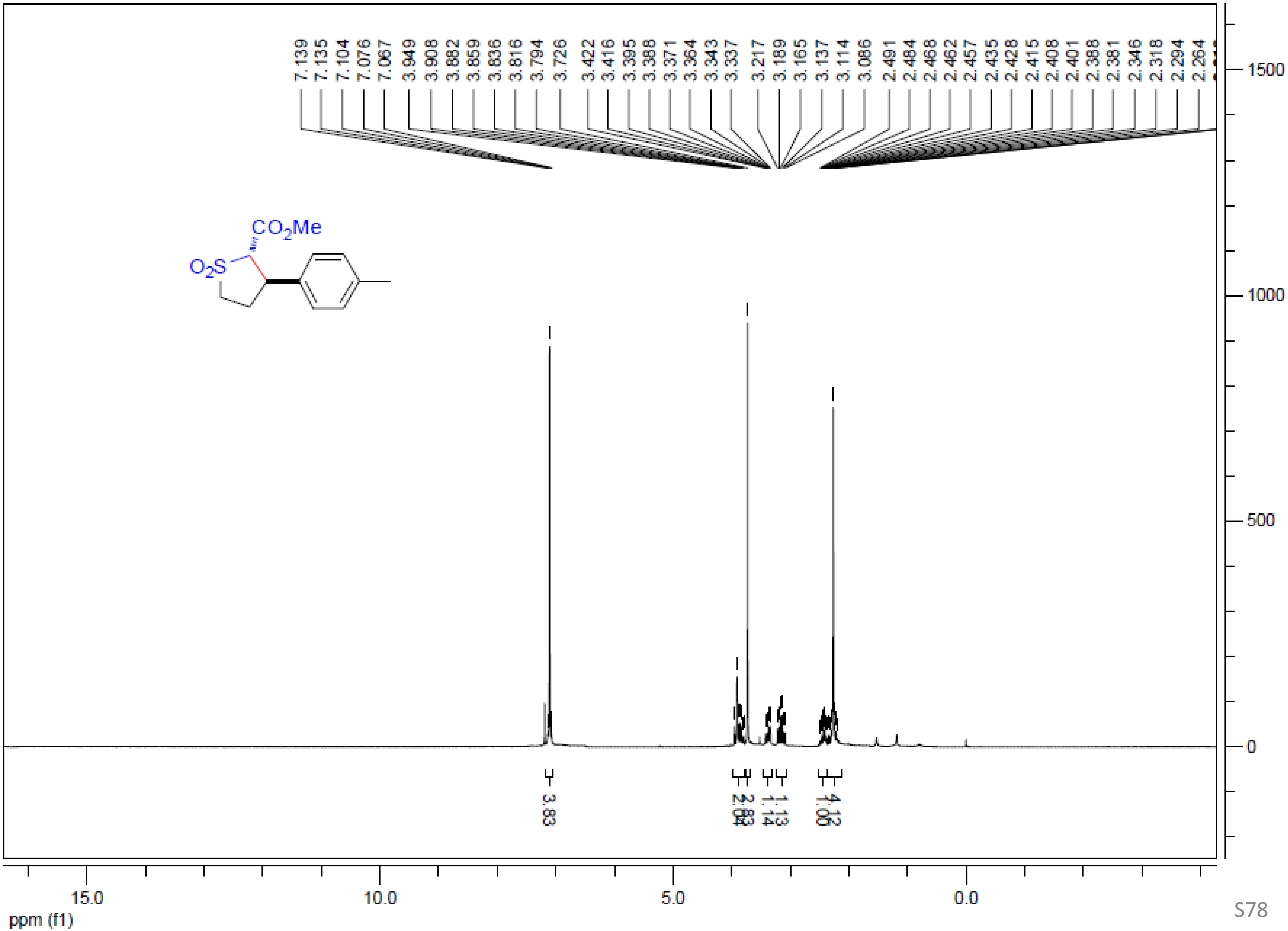

methyl 3-(p-tolyl)tetrahydrothiophene-2-carboxylate 1,1-dioxide (**2h**)

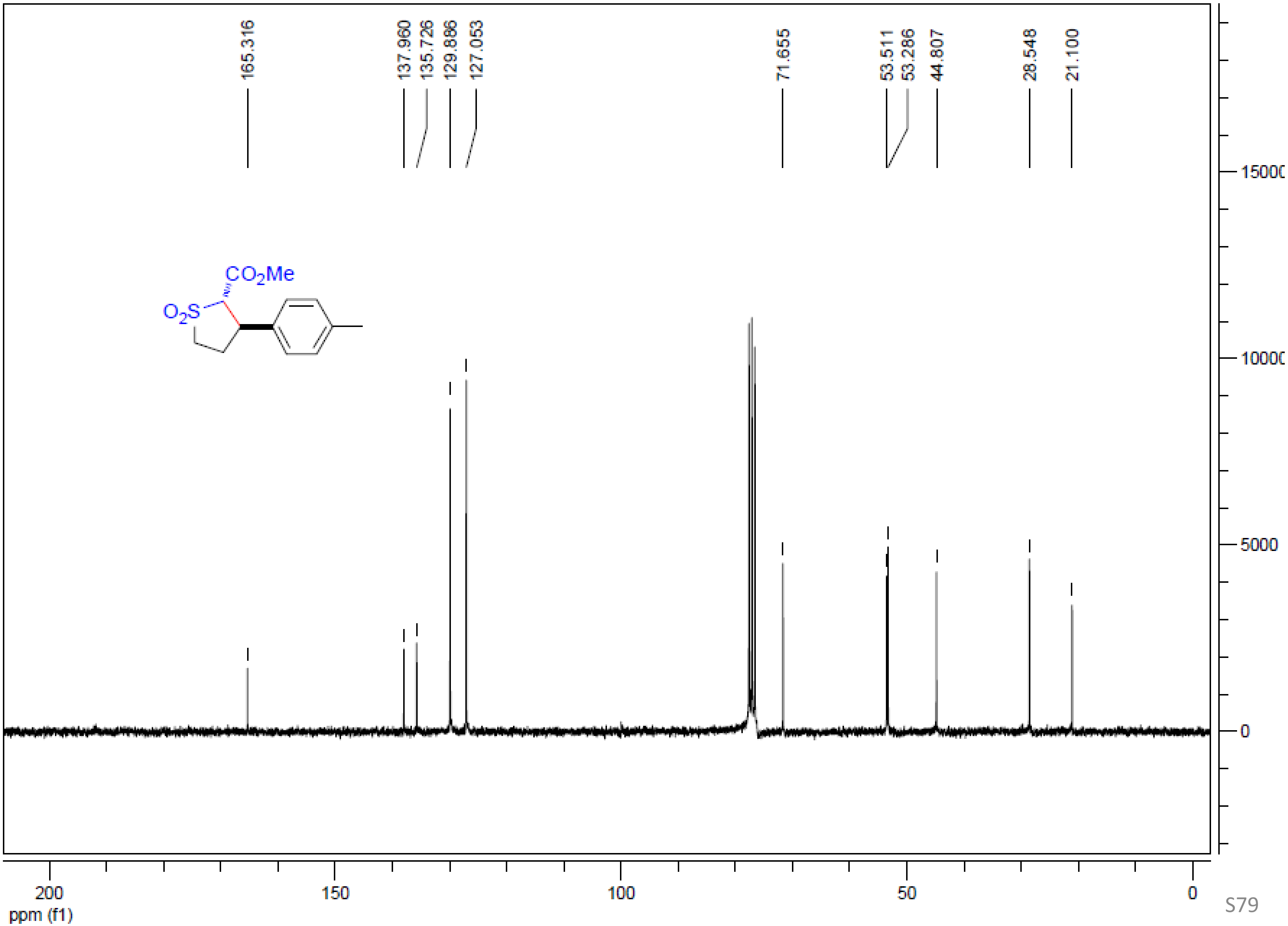

methyl 3-(p-tolyl)tetrahydrothiophene-2-carboxylate 1,1-dioxide (**2h**)

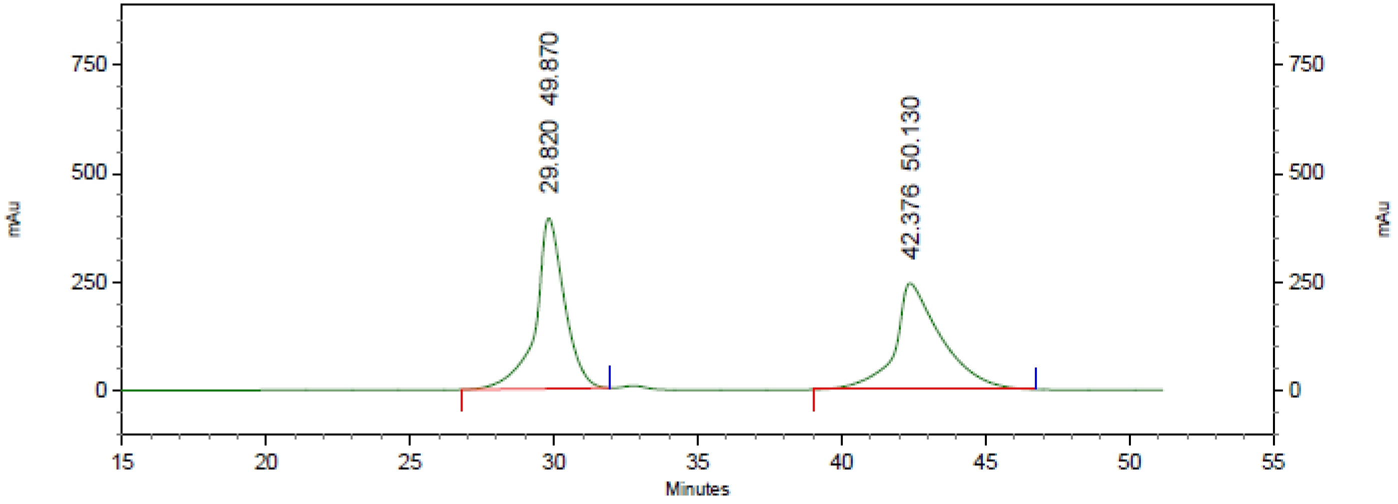

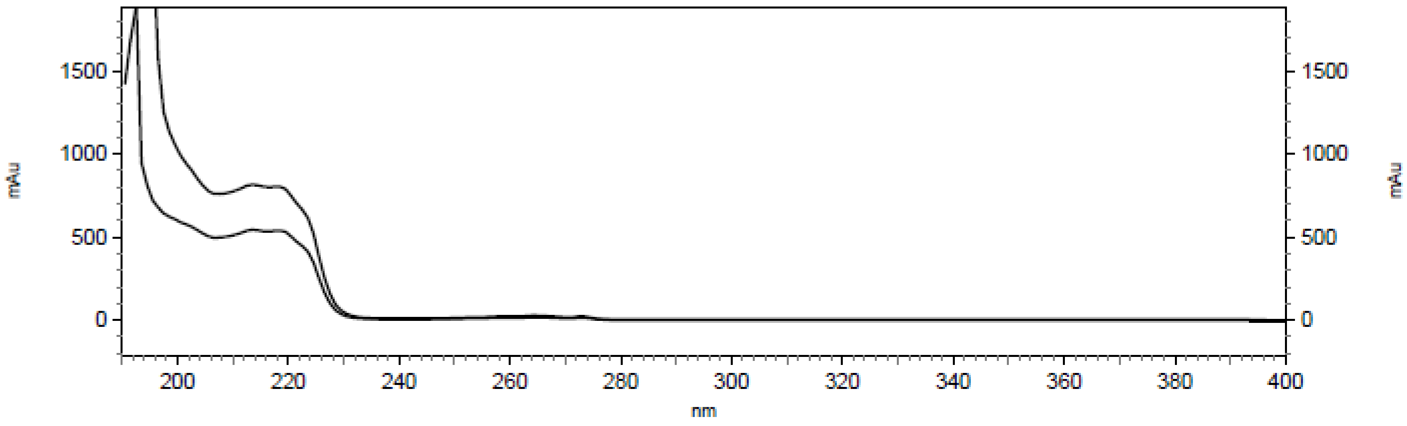

9: 218 nm, 4 nm Results

Pk #	Retention Time	Area Percent
1	29.820	49.870
2	42.376	50.130
Totals		100.000

methyl 3-(p-tolyl)tetrahydrothiophene-2-carboxylate 1,1-dioxide (**2h**)

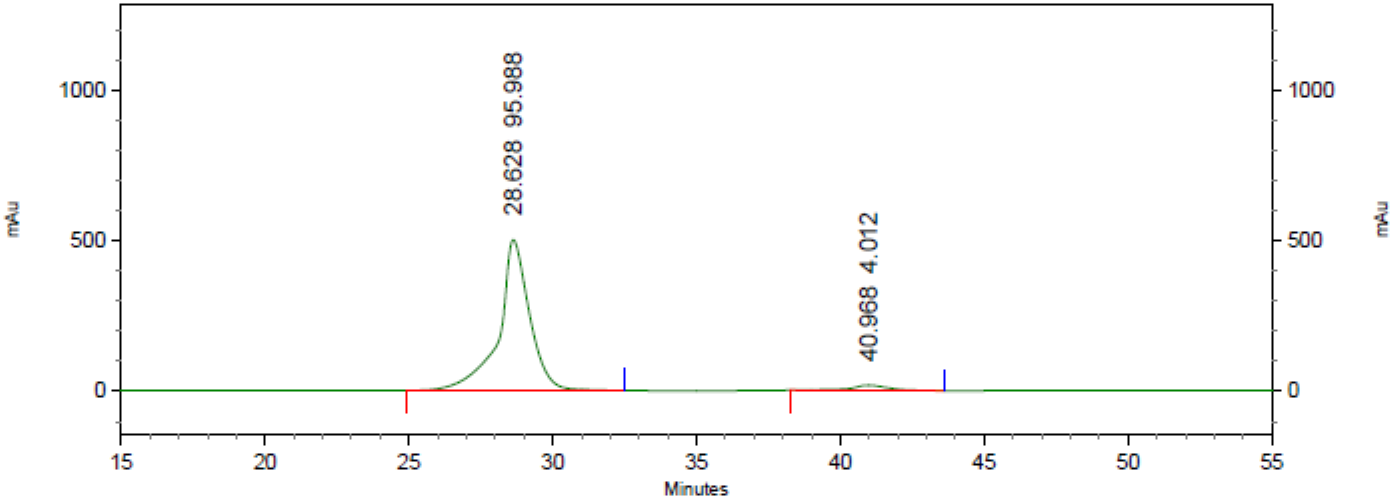

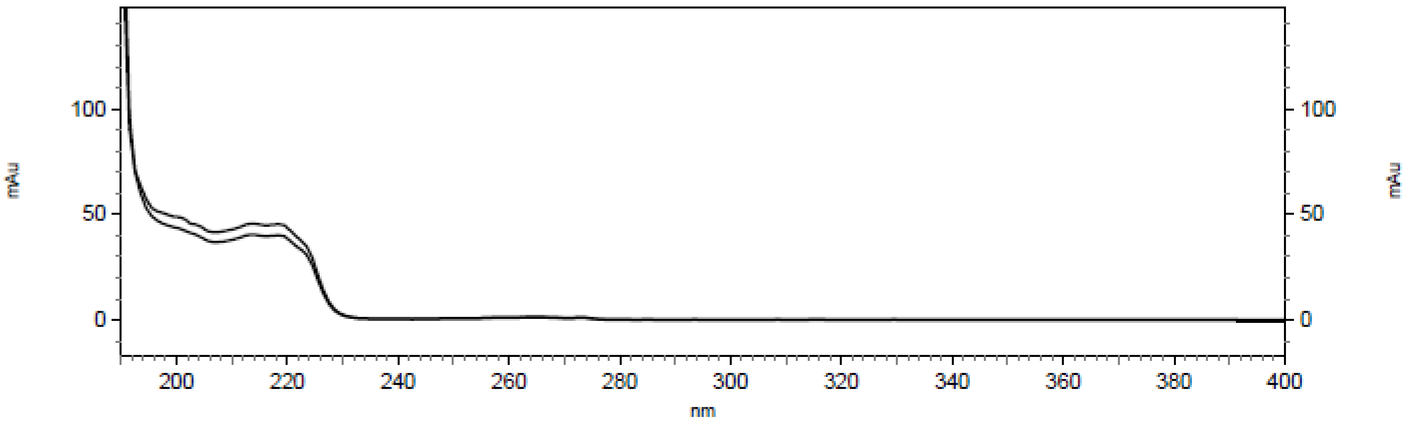

9: 218 nm, 4 nm Results

Pk #	Retention Time	Area Percent
1	28.628	95.988
2	40.968	4.012
Totals		100.000

methyl 3-(4-methoxyphenyl)tetrahydrothiophene-2-carboxylate 1,1-dioxide (**2i**)

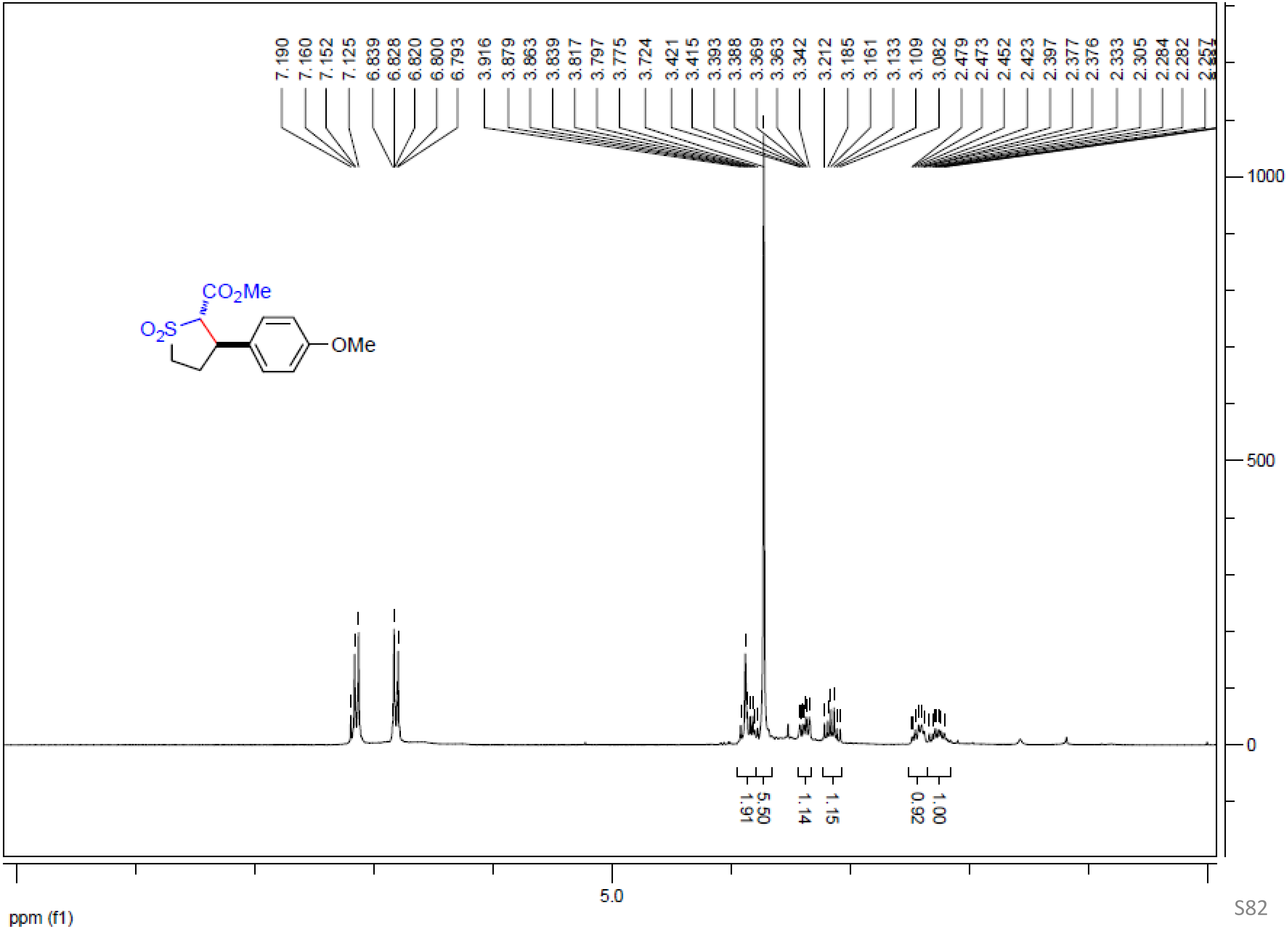

methyl 3-(4-methoxyphenyl)tetrahydrothiophene-2-carboxylate 1,1-dioxide (**2i**)

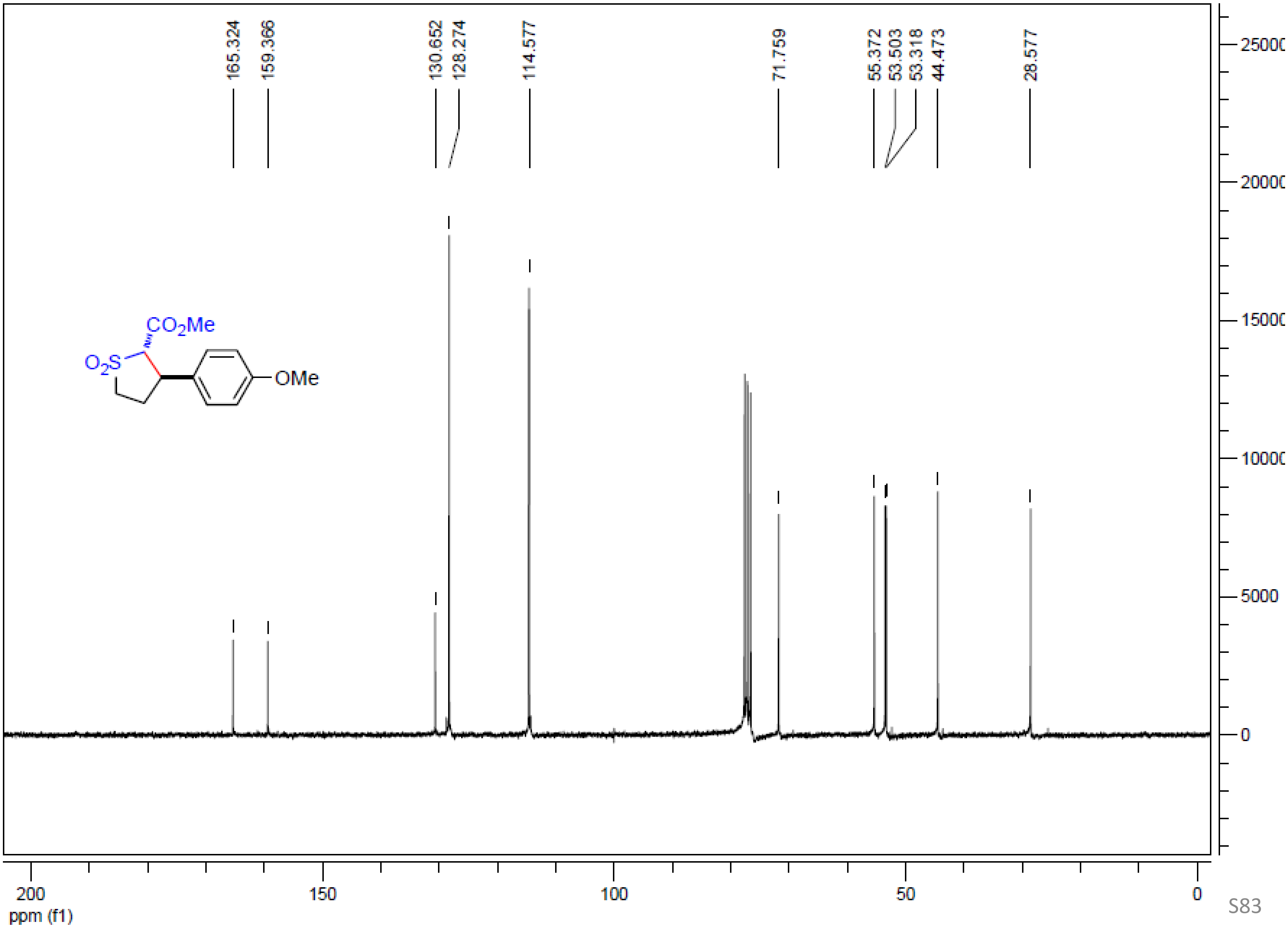

methyl 3-(4-methoxyphenyl)tetrahydrothiophene-2-carboxylate 1,1-dioxide (**2i**)

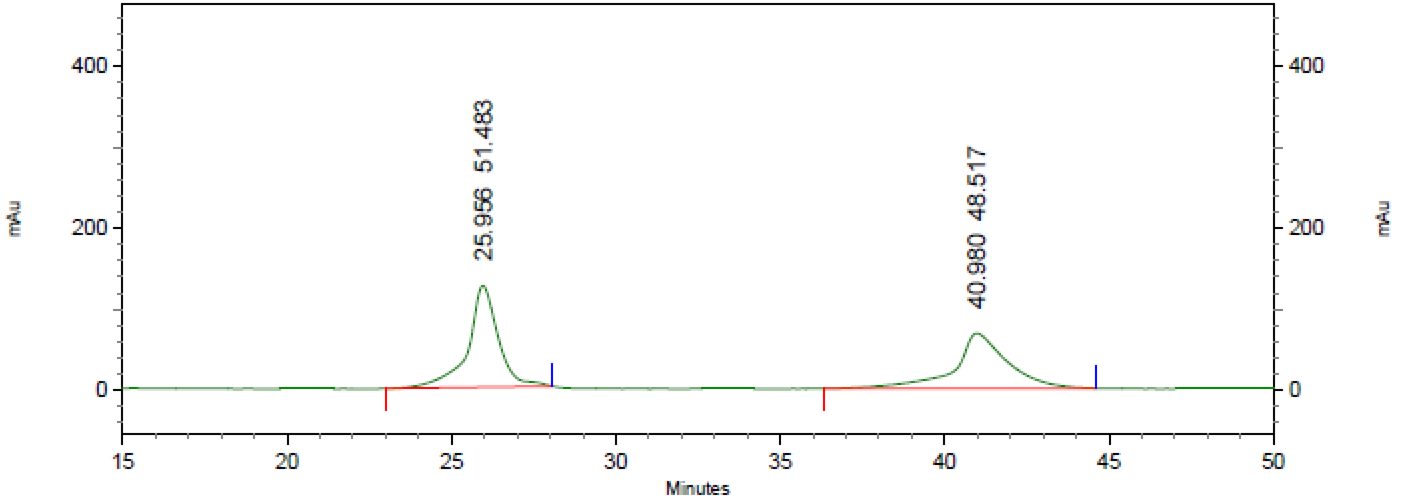

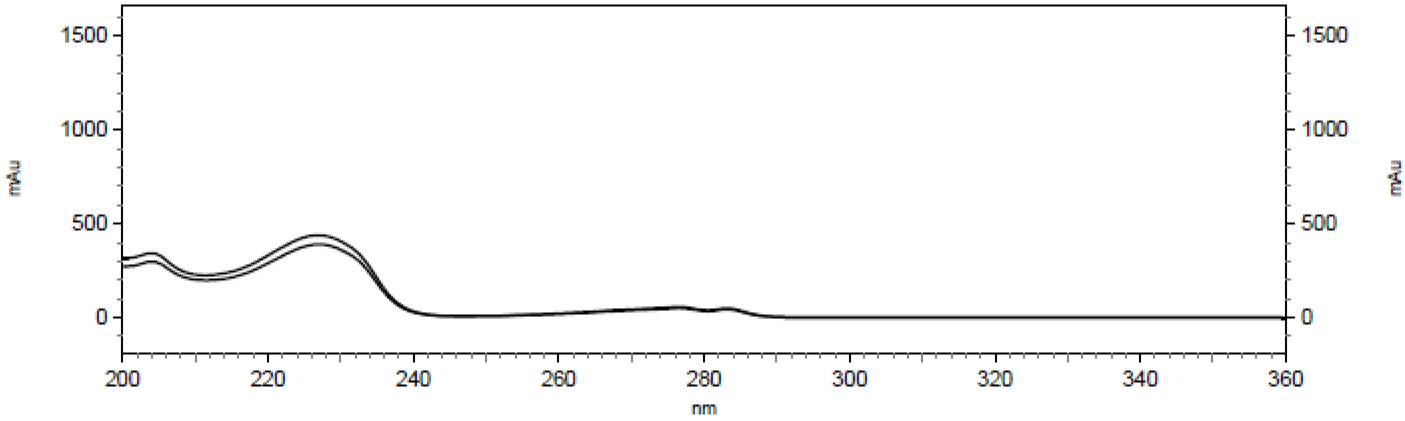

11: 224 nm, 4 nm Results

Pk #	Retention Time	Area Percent
1	25.956	51.483
2	40.980	48.517
Totals		100.000

methyl 3-(4-methoxyphenyl)tetrahydrothiophene-2-carboxylate 1,1-dioxide (**2i**)

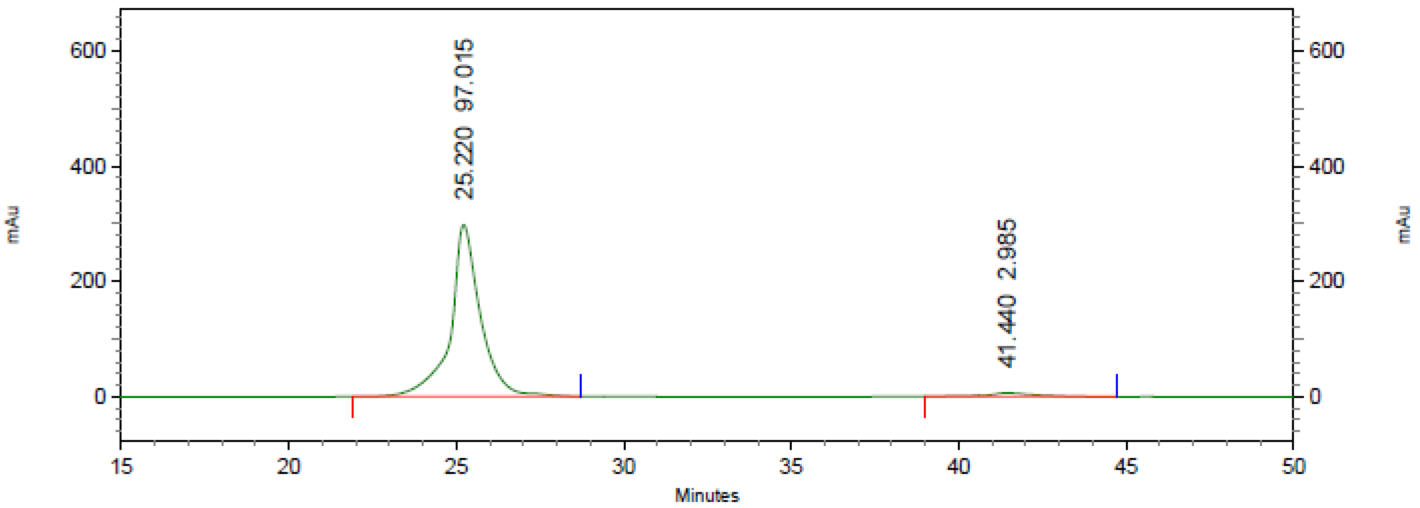

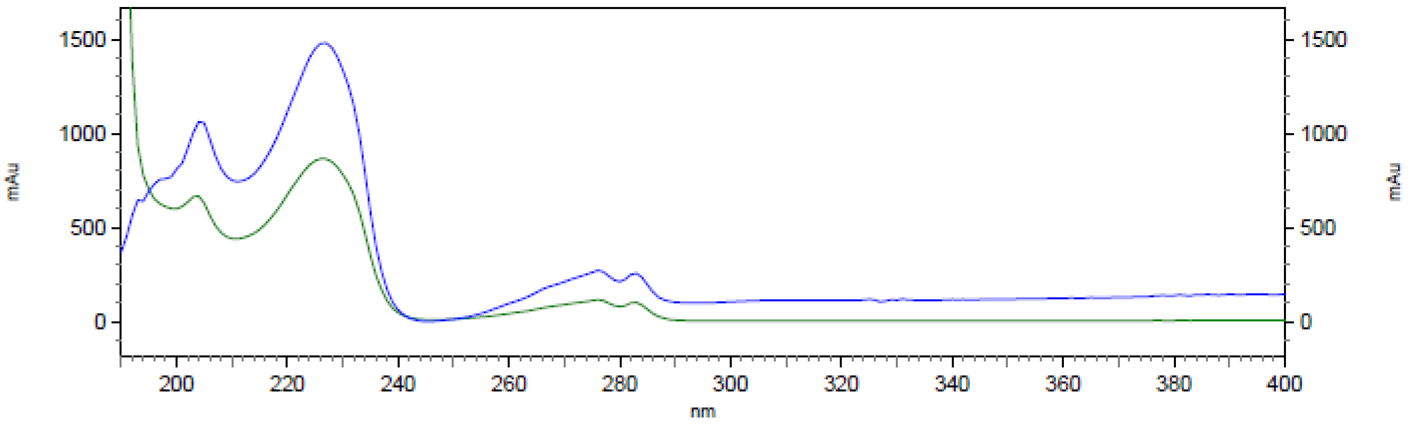

1: 272 nm, 4 nm Results

Pk #	Retention Time	Area Percent
1	25.220	97.015
2	41.440	2.985
Totals		100.000

methyl 3-(4-(methoxymethoxy)phenyl)tetrahydrothiophene-2-carboxylate 1,1-dioxide (**2j**)

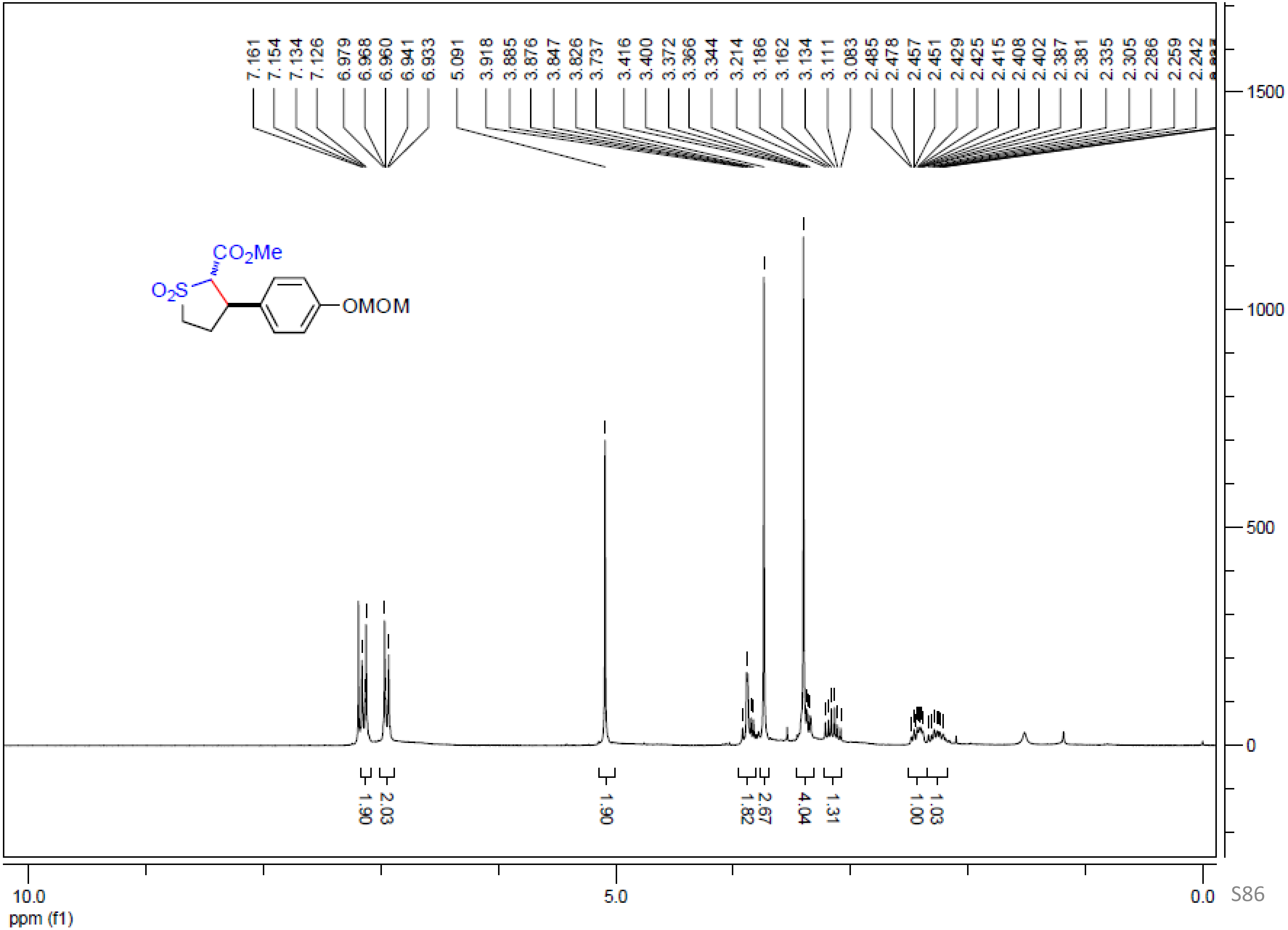

methyl 3-(4-(methoxymethoxy)phenyl)tetrahydrothiophene-2-carboxylate 1,1-dioxide (**2j**)

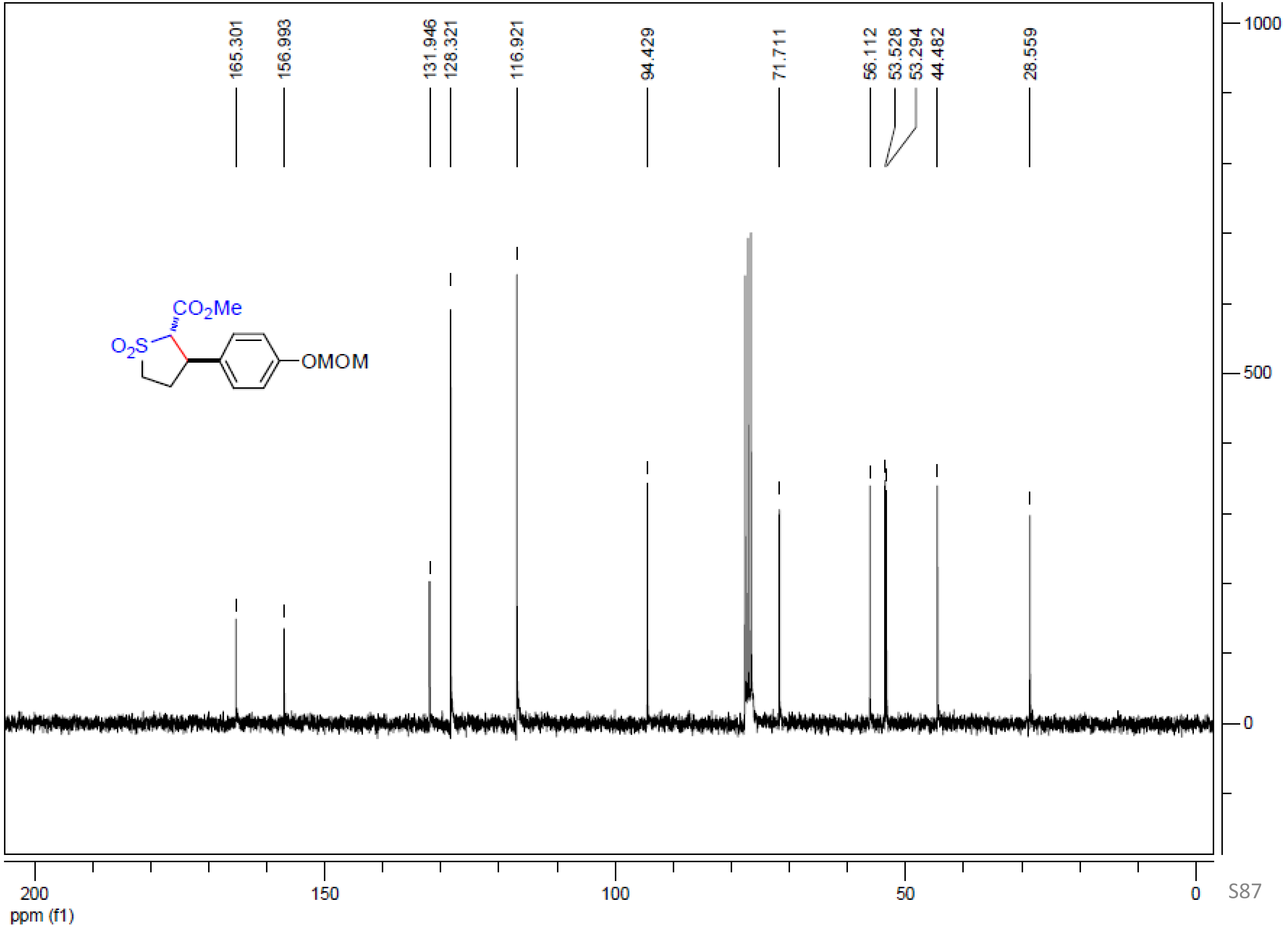

methyl 3-(4-(methoxymethoxy)phenyl)tetrahydrothiophene-2-carboxylate 1,1-dioxide (**2j**)

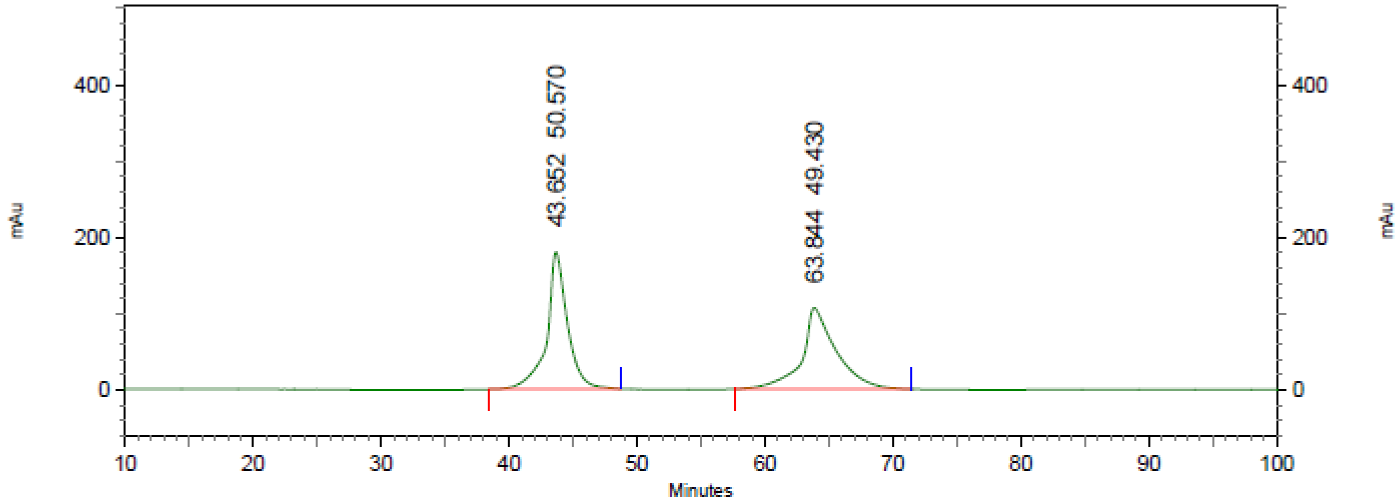

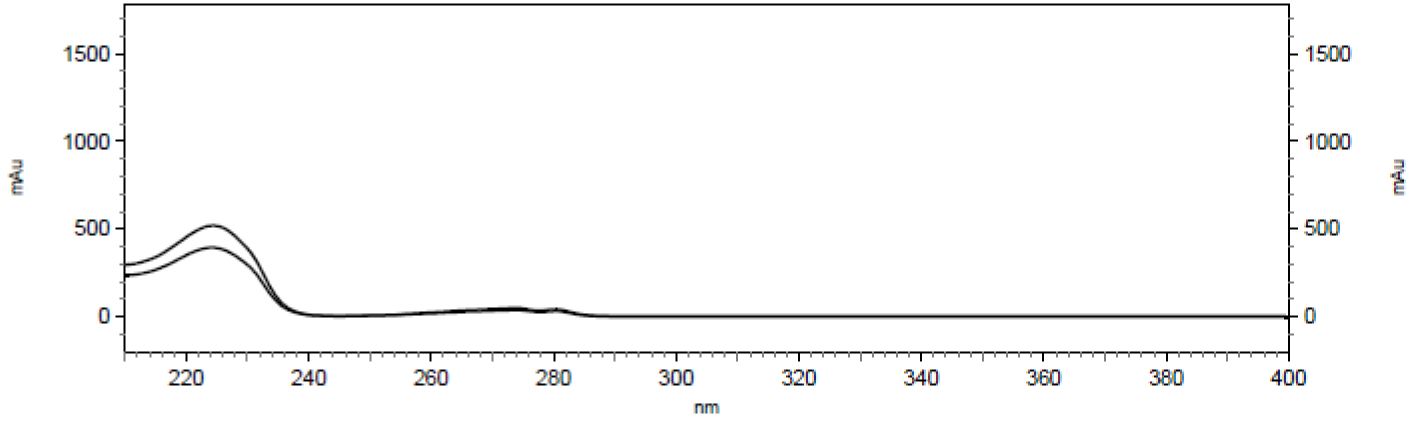

14: 225 nm, 4 nm Results

Pk #	Retention Time	Area Percent
1	43.652	50.570
2	63.844	49.430
Totals		100.000

methyl 3-(4-(methoxymethoxy)phenyl)tetrahydrothiophene-2-carboxylate 1,1-dioxide (**2j**)

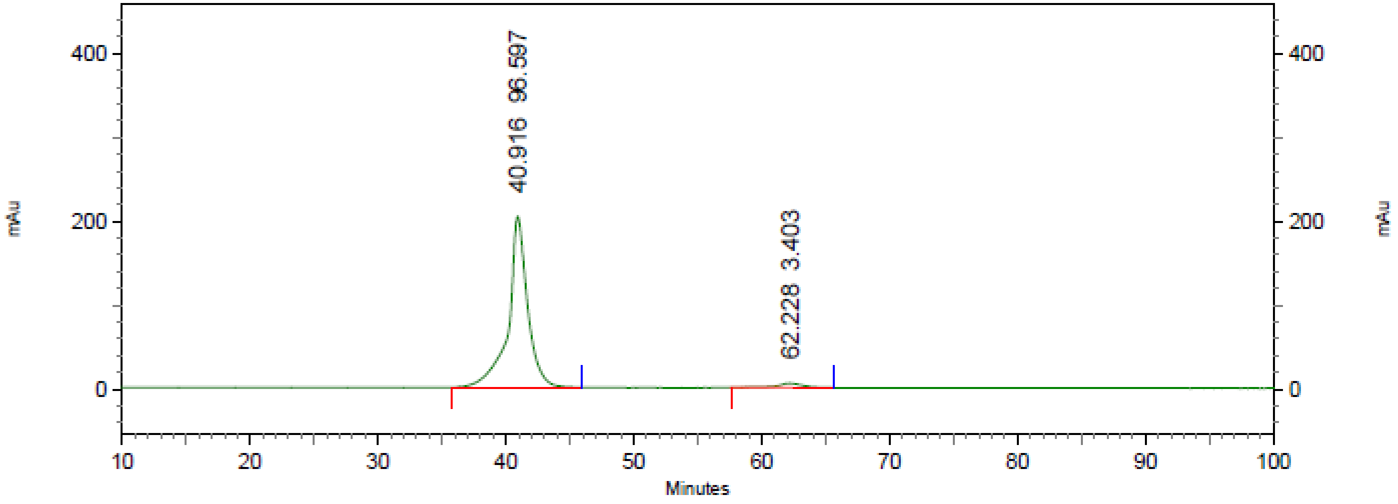

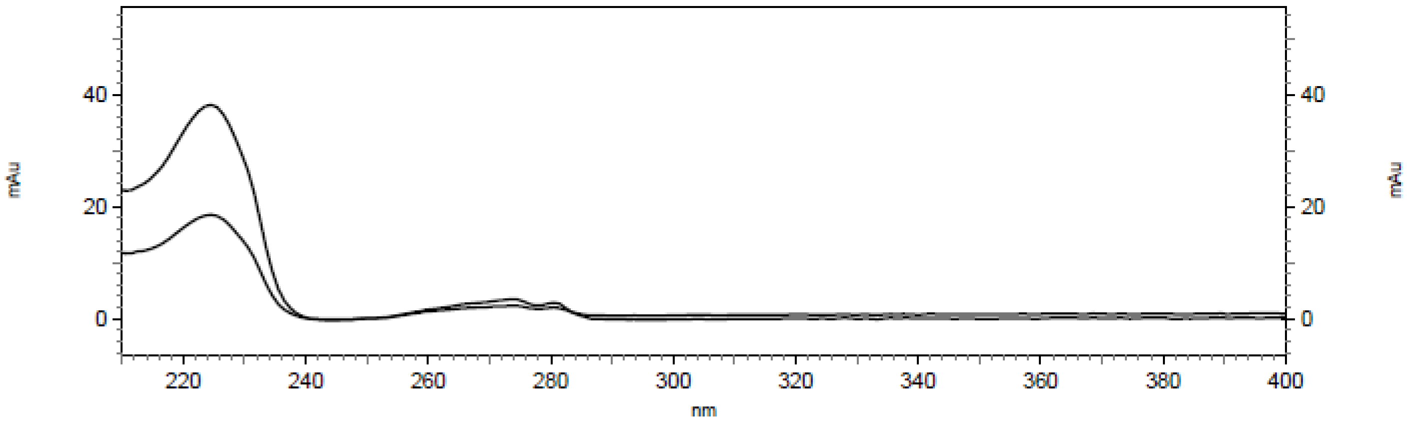

14: 225 nm, 4 nm Results

Pk #	Retention Time	Area Percent
1	40.916	96.597
2	62.228	3.403
Totals		100.000

methyl 3-(4-hydroxyphenyl)tetrahydrothiophene-2-carboxylate 1,1-dioxide (**2k**)

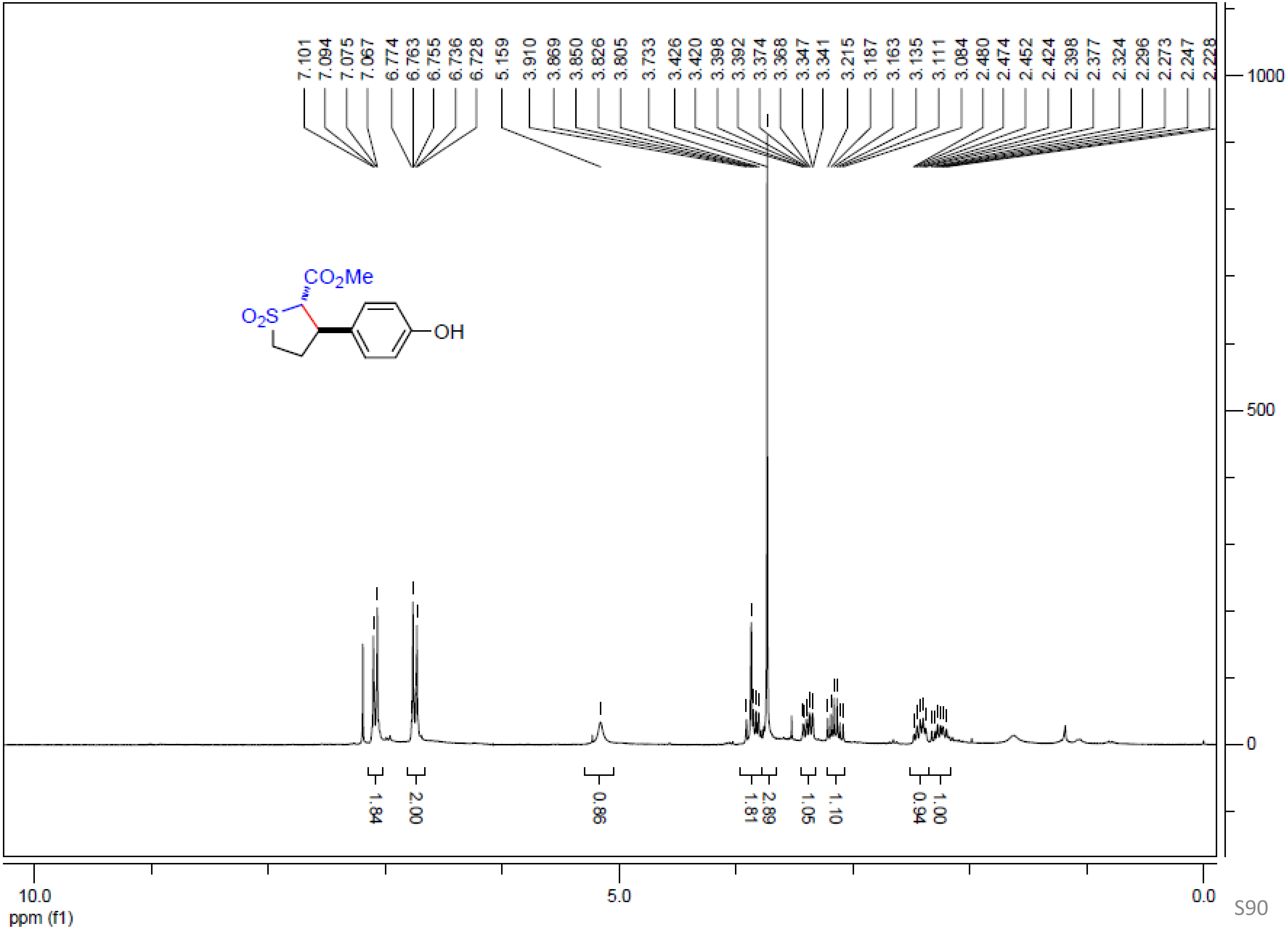

methyl 3-(4-hydroxyphenyl)tetrahydrothiophene-2-carboxylate 1,1-dioxide (**2k**)

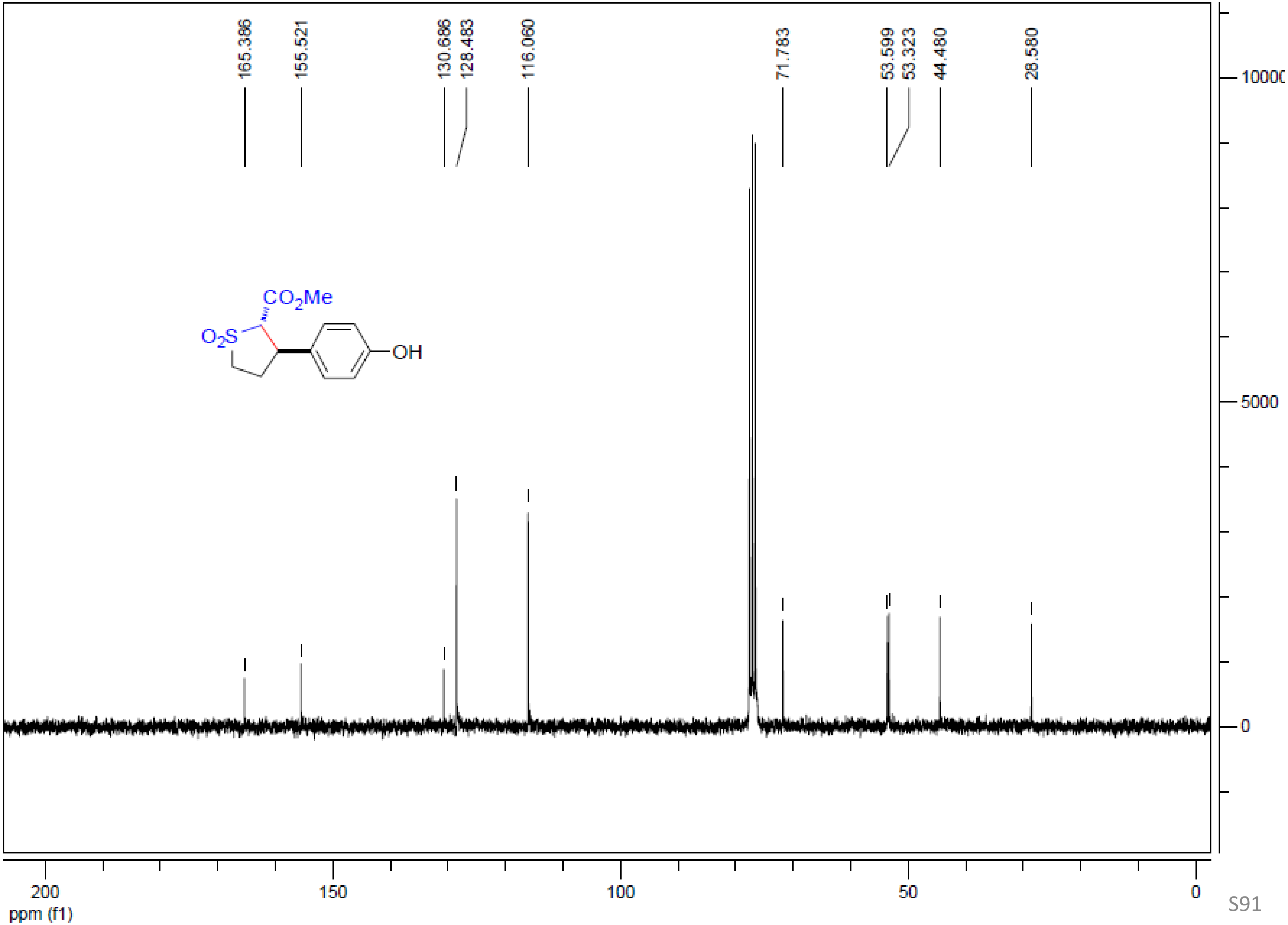

methyl 3-(4-hydroxyphenyl)tetrahydrothiophene-2-carboxylate 1,1-dioxide (**2k**)

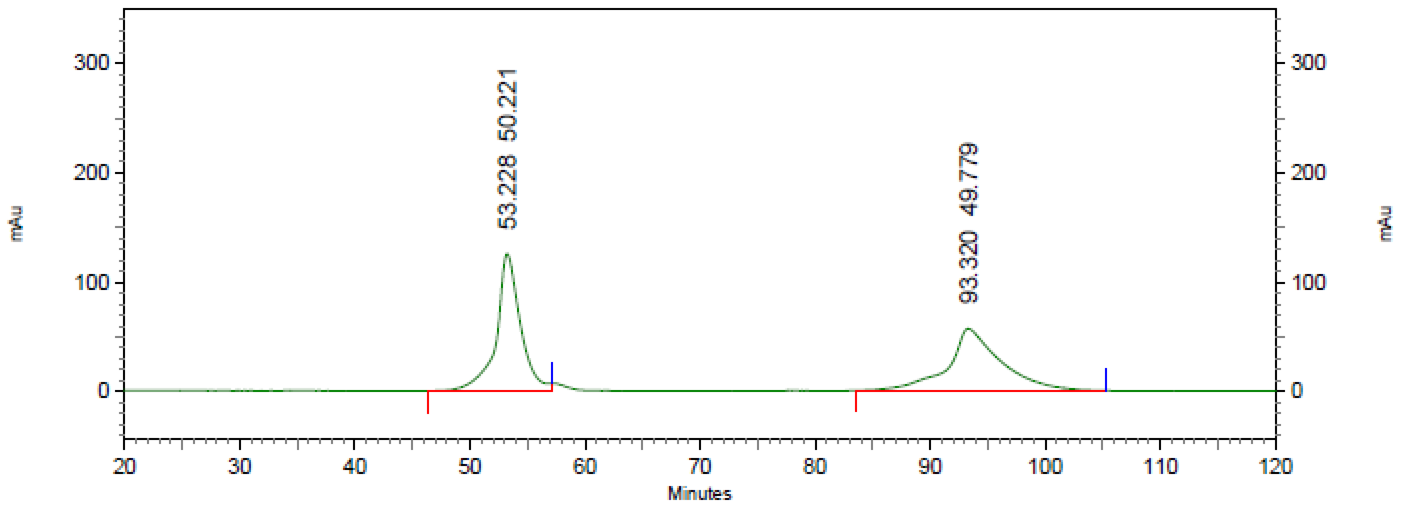

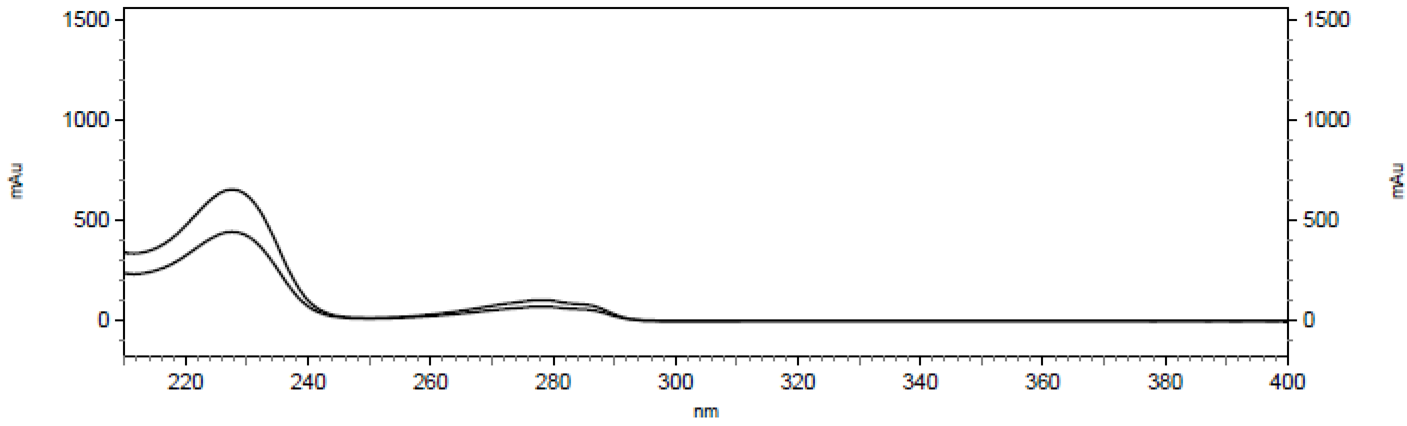

14: 225 nm, 4 nm Results

Pk #	Retention Time	Area Percent
1	53.228	50.221
2	93.320	49.779
Totals		100.000

methyl 3-(4-hydroxyphenyl)tetrahydrothiophene-2-carboxylate 1,1-dioxide (**2k**)

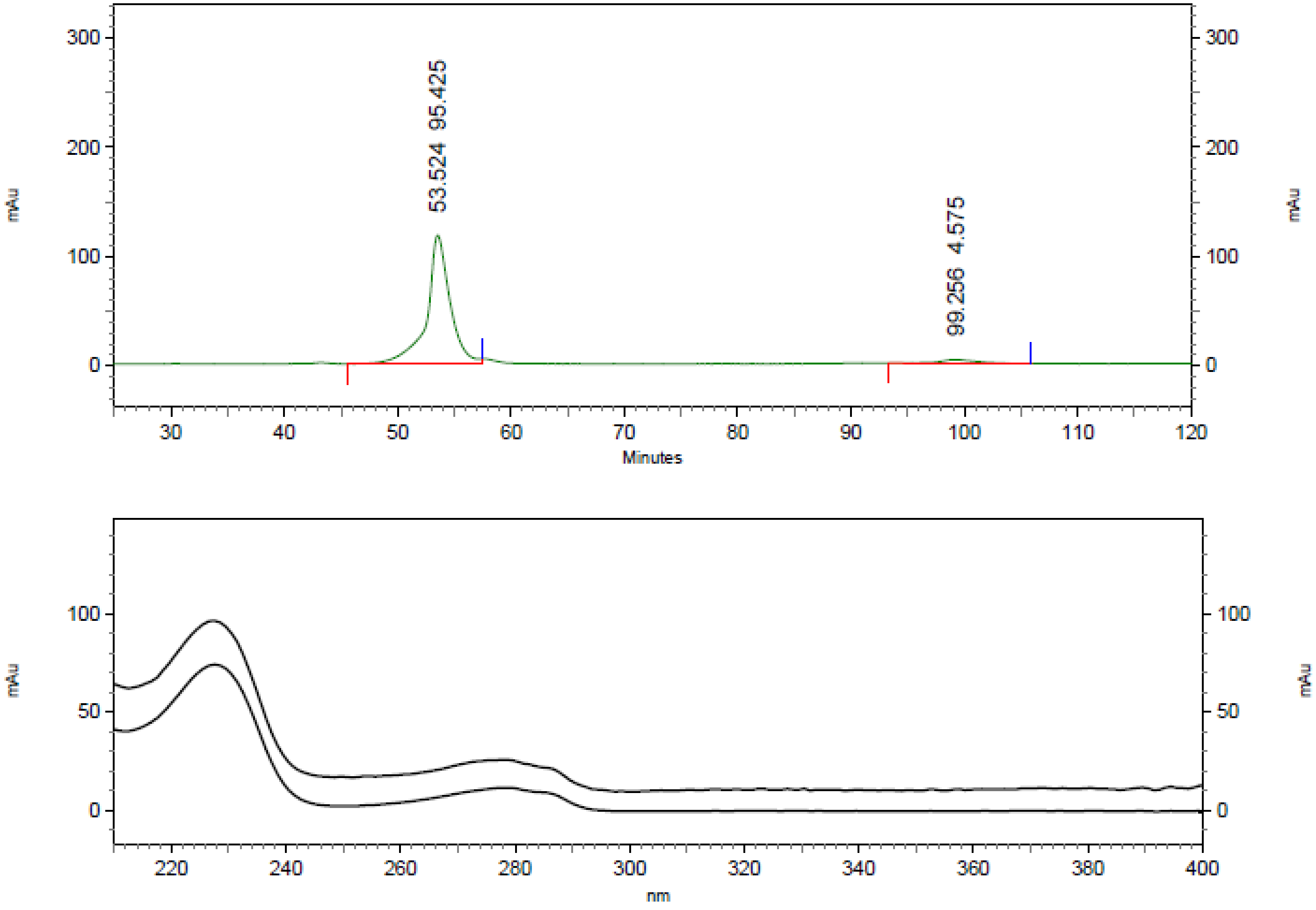

14: 225 nm, 4 nm Results

Pk #	Retention Time	Area Percent
1	53.524	95.425
2	99.256	4.575
Totals		100.000

methyl 3-(4-aminophenyl)tetrahydrothiophene-2-carboxylate 1,1-dioxide (**2l**)

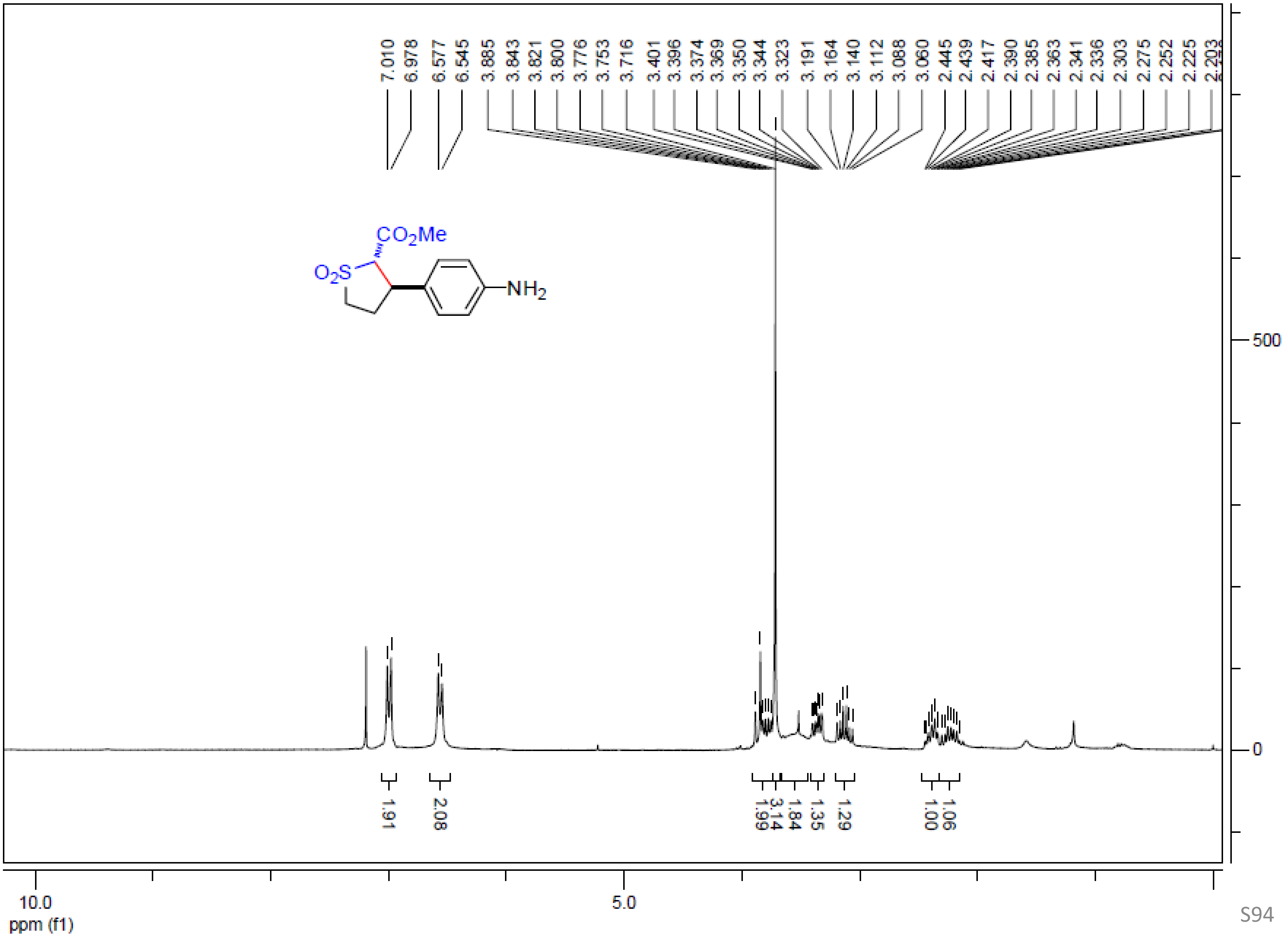

methyl 3-(4-aminophenyl)tetrahydrothiophene-2-carboxylate 1,1-dioxide (**2l**)

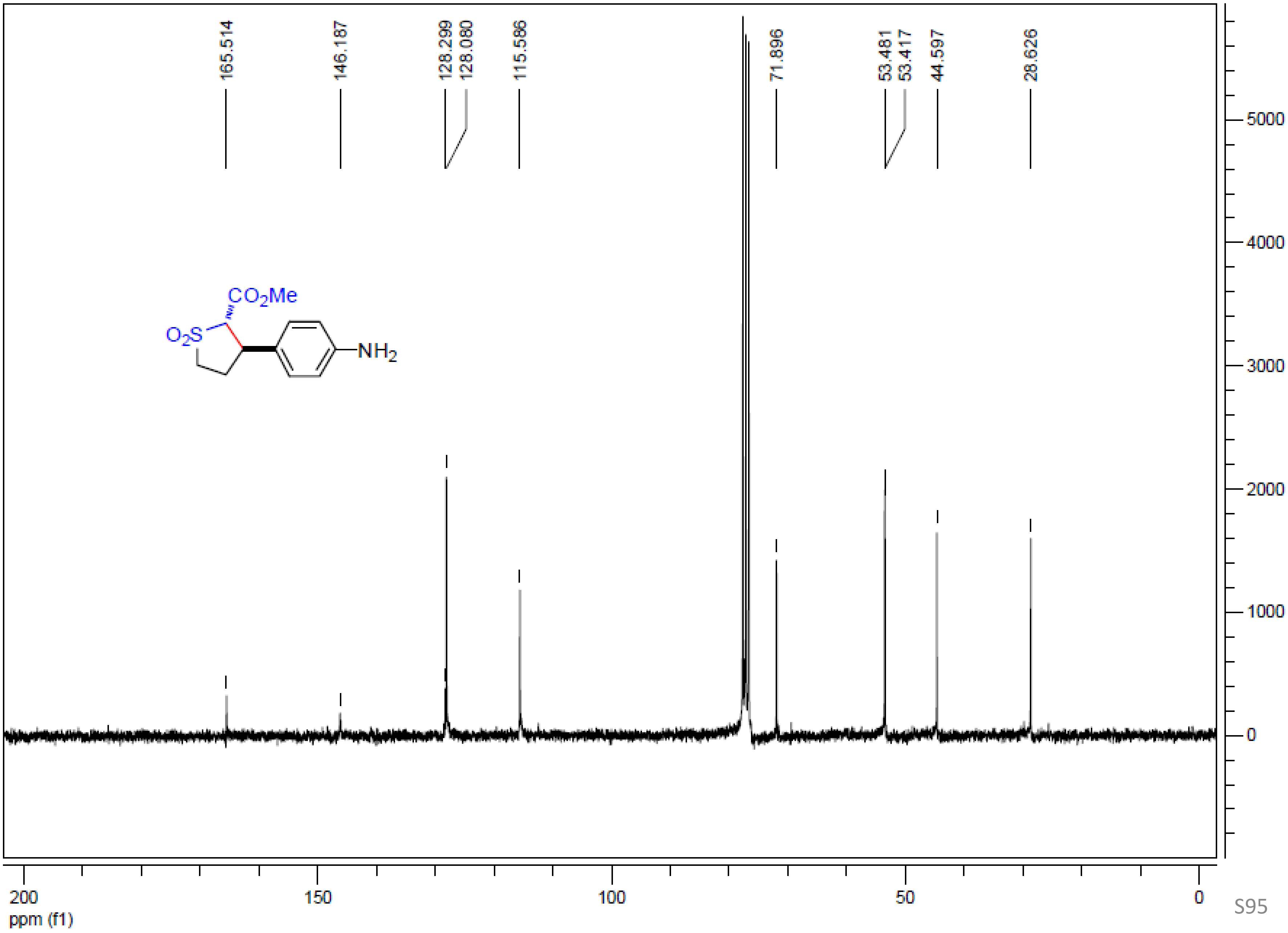

methyl 3-(4-aminophenyl)tetrahydrothiophene-2-carboxylate 1,1-dioxide (**2l**)

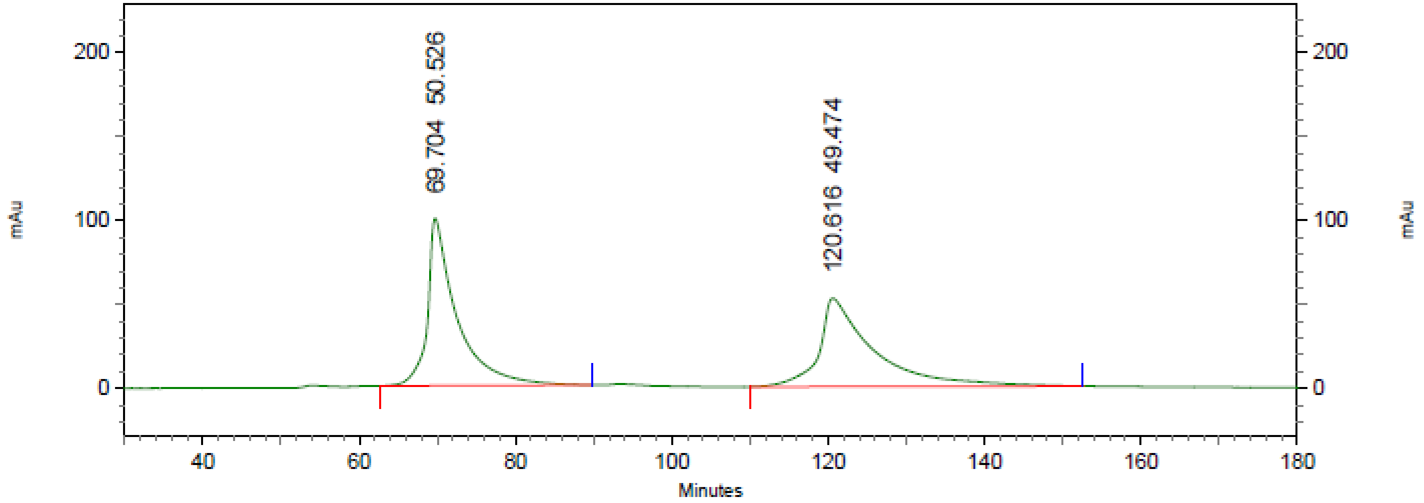

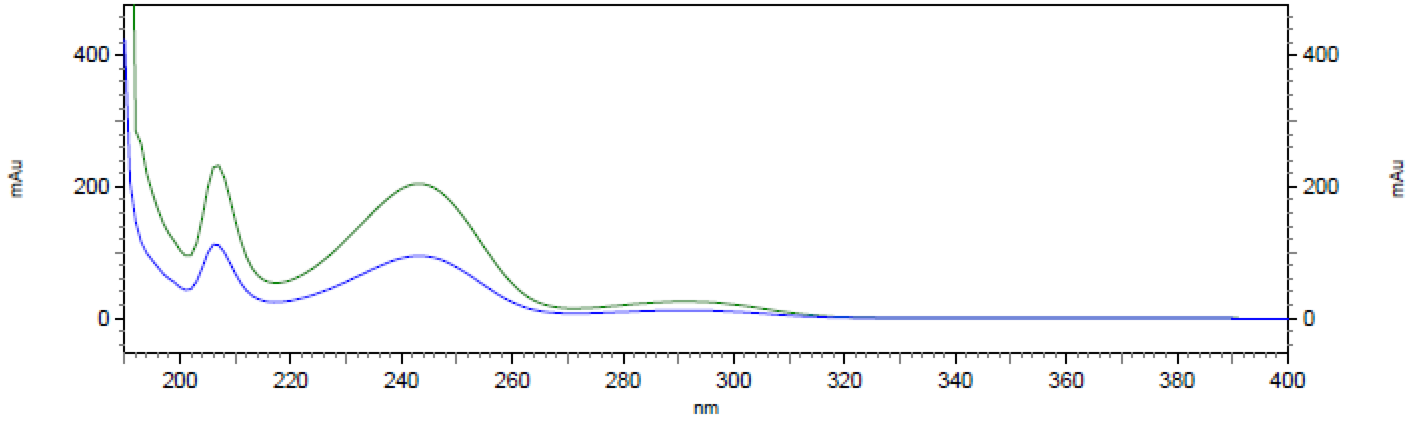

1: 246 nm, 4 nm Results

Pk #	Retention Time	Area Percent
1	69.704	50.526
2	120.616	49.474
Totals		100.000

methyl 3-(4-aminophenyl)tetrahydrothiophene-2-carboxylate 1,1-dioxide (**2l**)

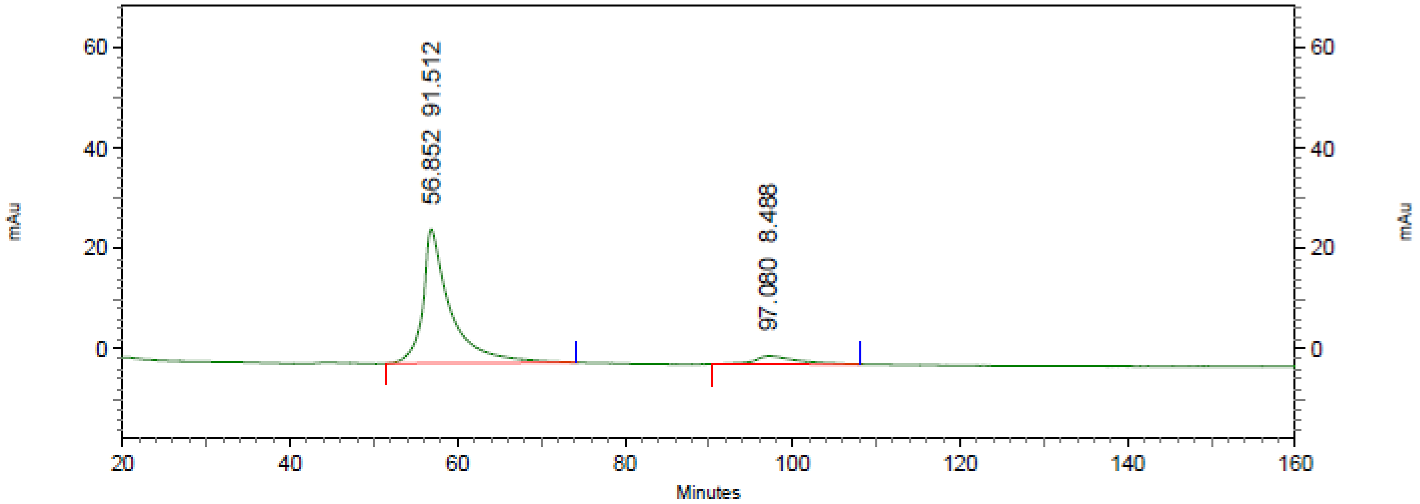

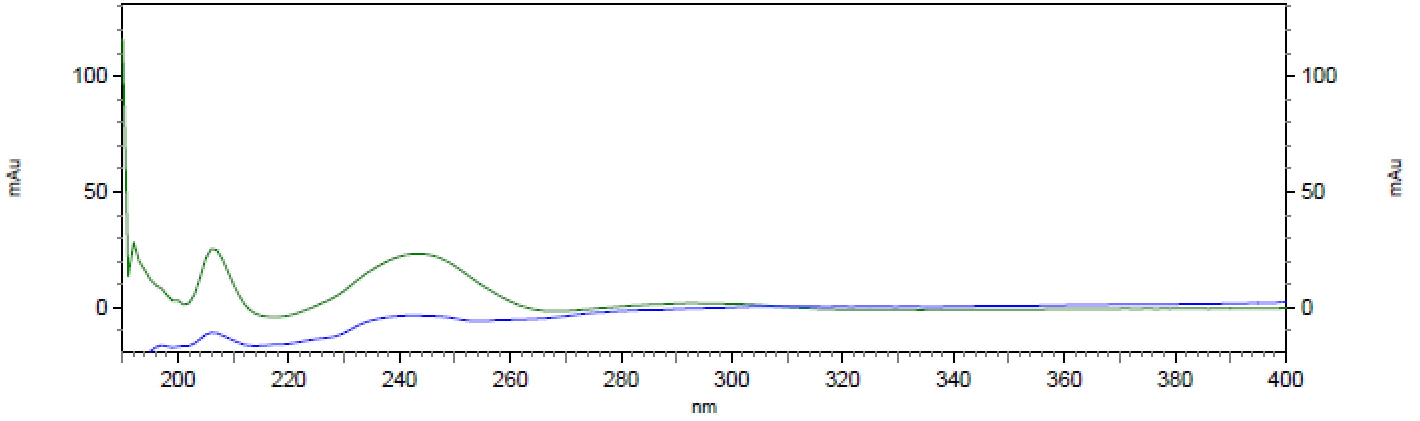

1: 247 nm, 4 nm Results

Pk #	Retention Time	Area Percent
1	56.852	91.512
2	97.080	8.488
Totals		100.000

methyl 3-(4-acetamidophenyl)tetrahydrothiophene-2-carboxylate 1,1-dioxide (**2m**)

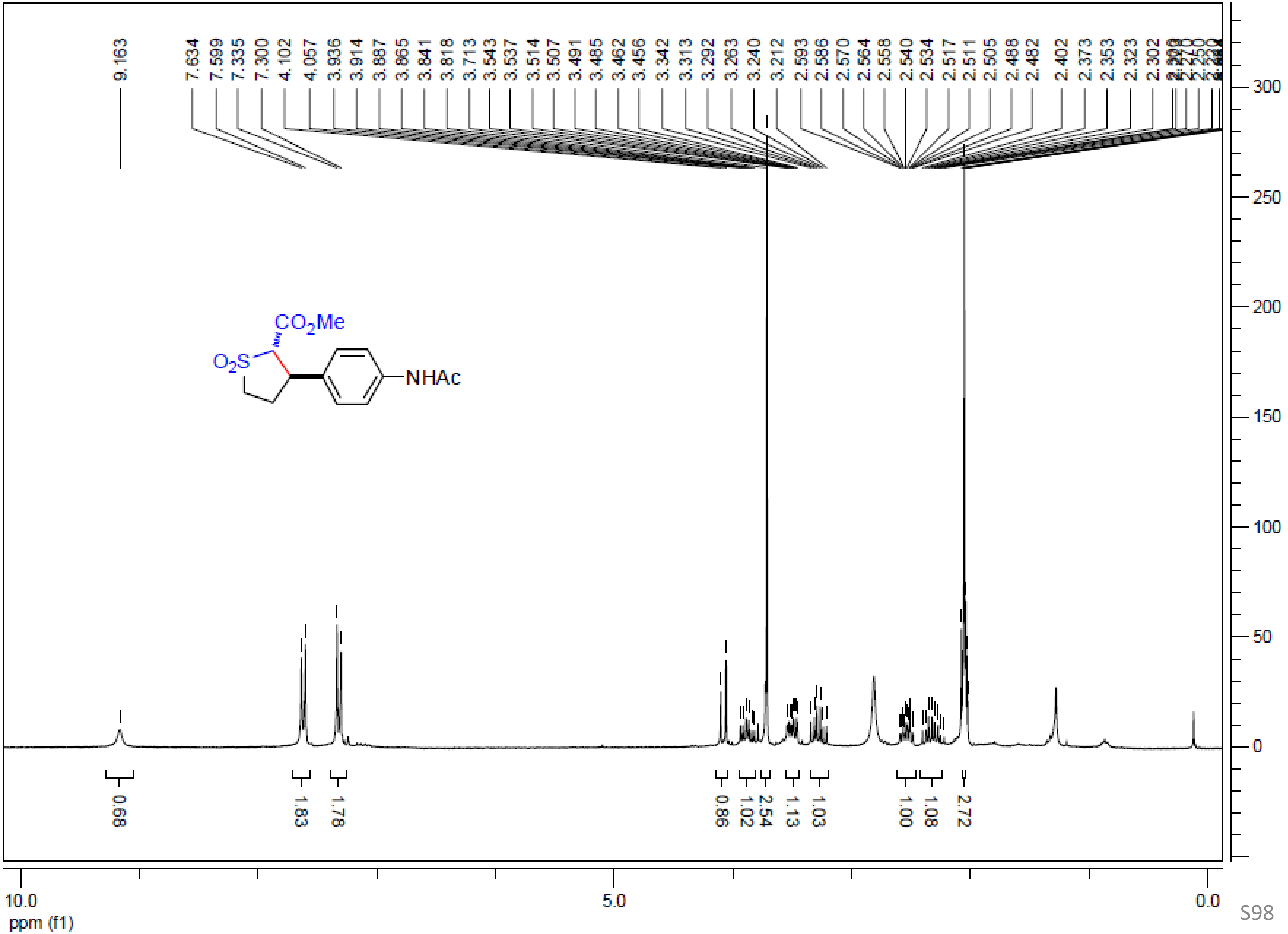

methyl 3-(4-acetamidophenyl)tetrahydrothiophene-2-carboxylate 1,1-dioxide (**2m**)

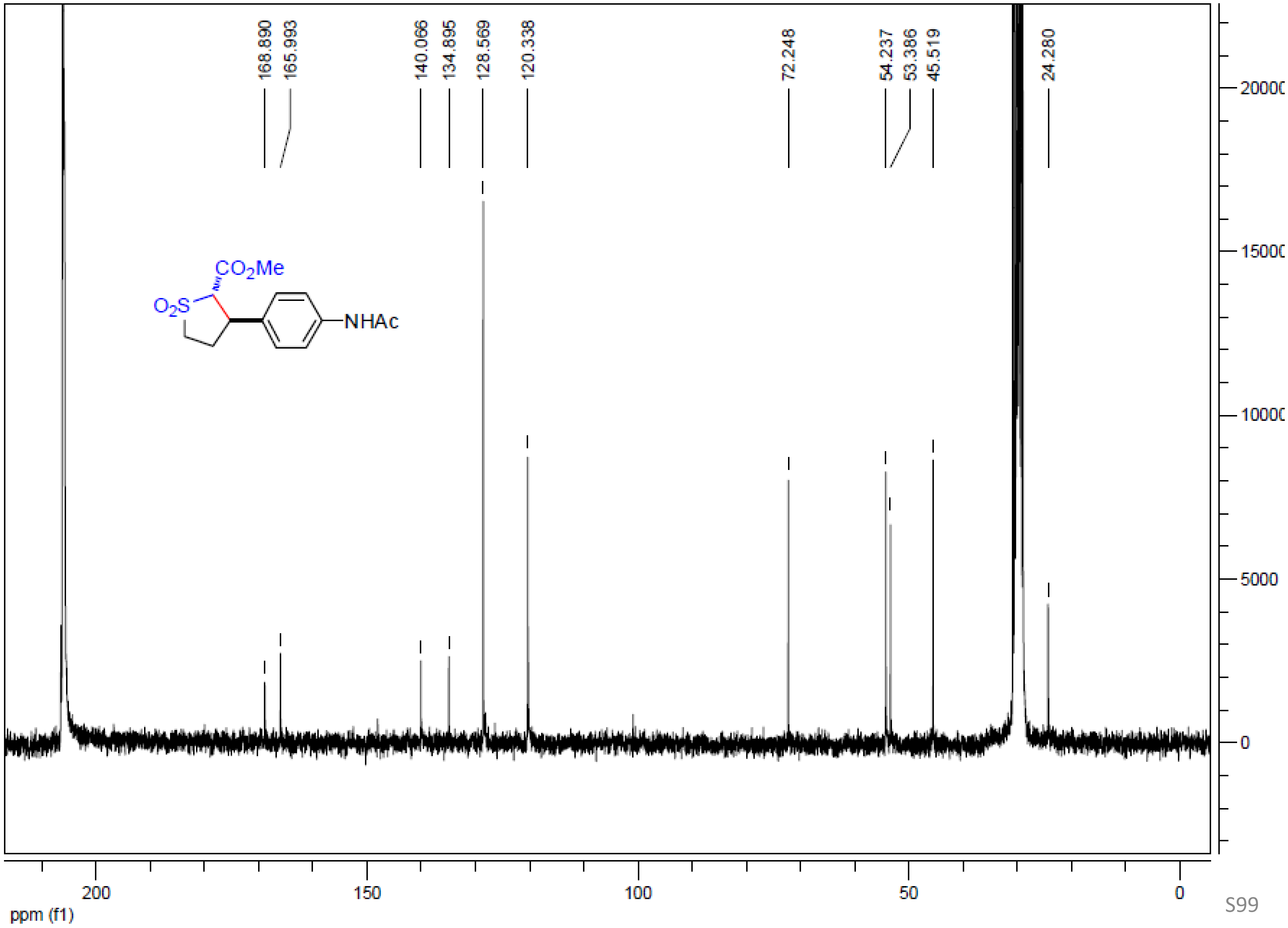

methyl 3-(4-acetamidophenyl)tetrahydrothiophene-2-carboxylate 1,1-dioxide (**2m**)

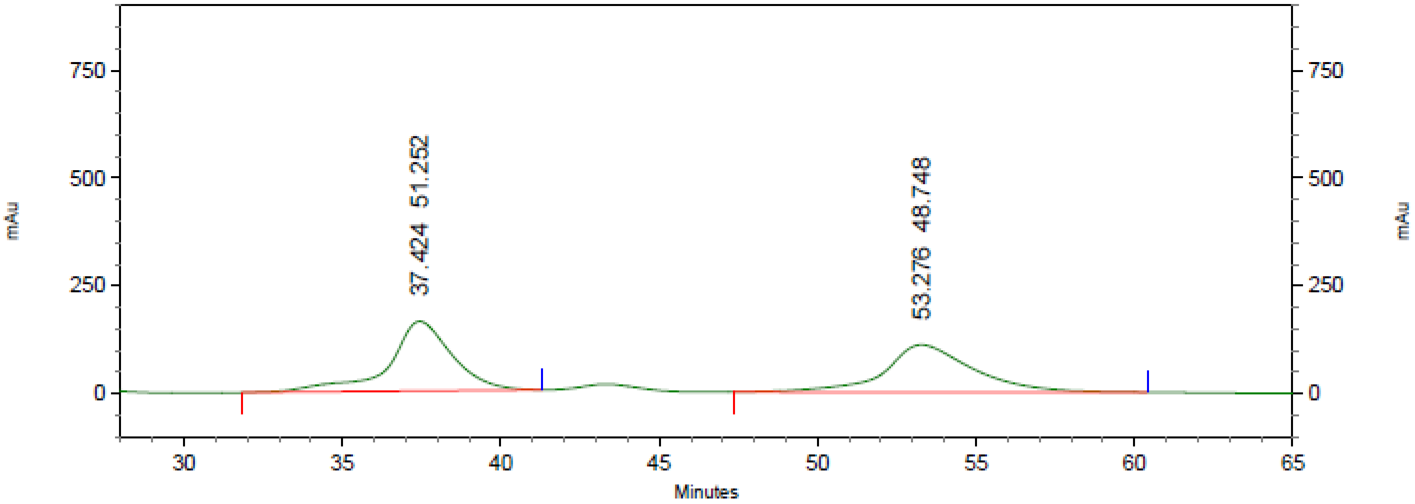

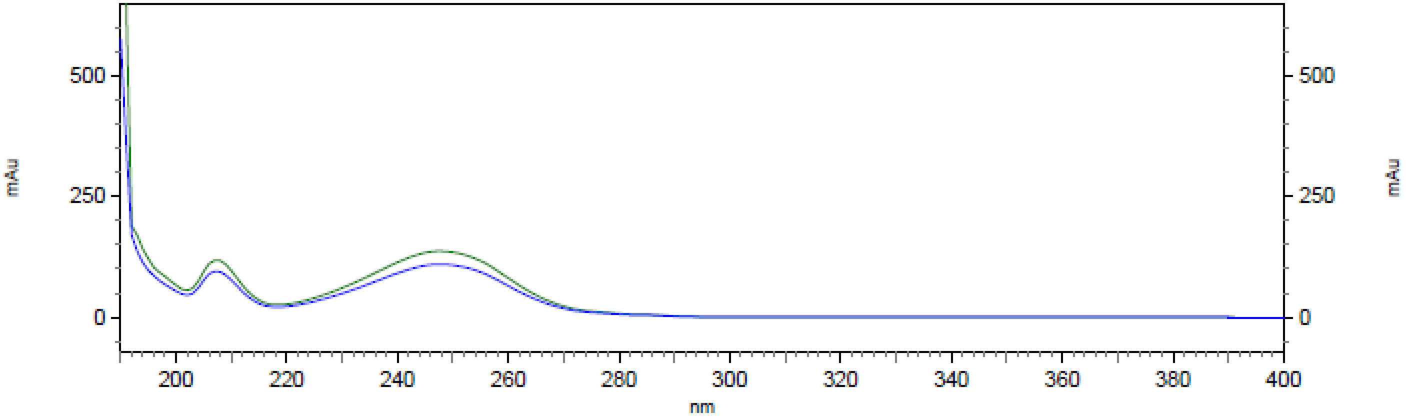

2: 247 nm, 4 nm Results

Pk #	Retention Time	Area Percent
1	37.424	51.252
2	53.276	48.748
Totals		100.000

methyl 3-(4-acetamidophenyl)tetrahydrothiophene-2-carboxylate 1,1-dioxide (**2m**)

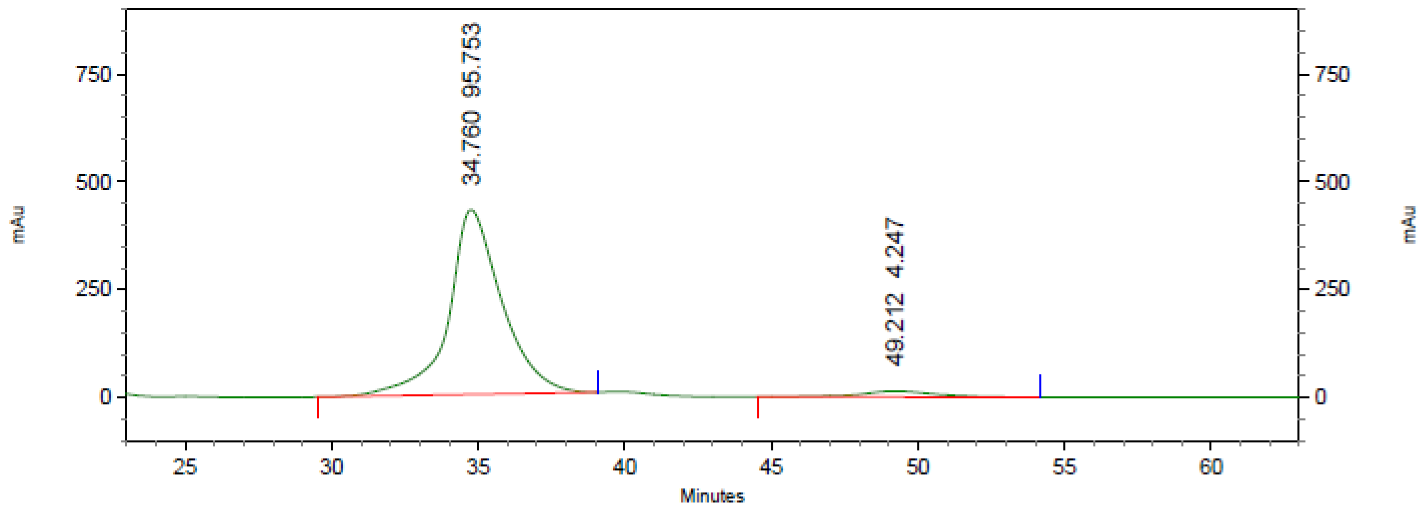

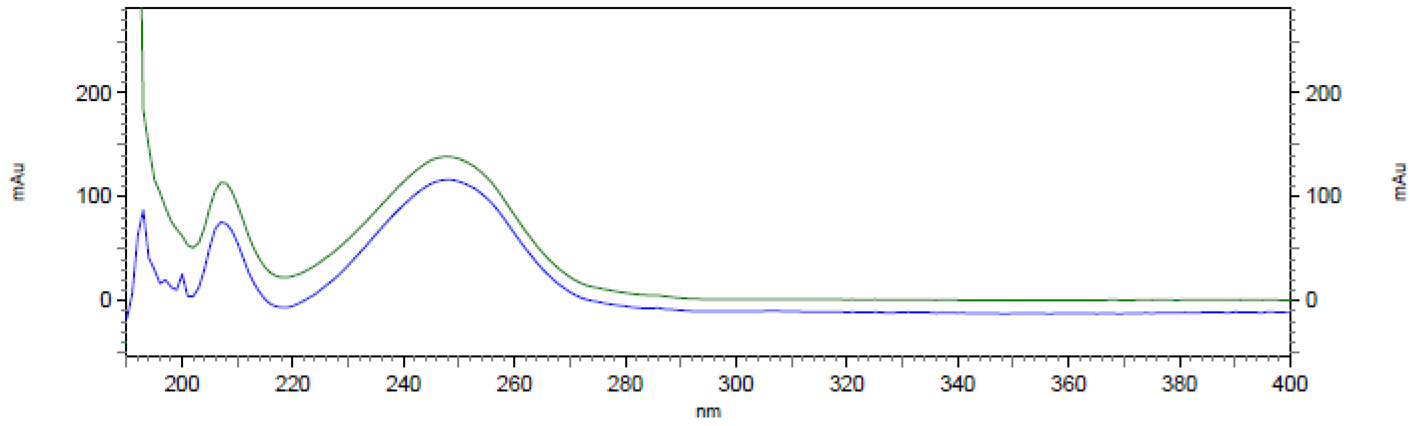

2: 250 nm, 4 nm Results

Pk #	Retention Time	Area Percent
1	34.760	95.753
2	49.212	4.247
Totals		100.000

methyl 3-(1-phenyl-1H-1,2,3-triazol-4-yl)tetrahydrothiophene-2-carboxylate 1,1-dioxide (**2n**)

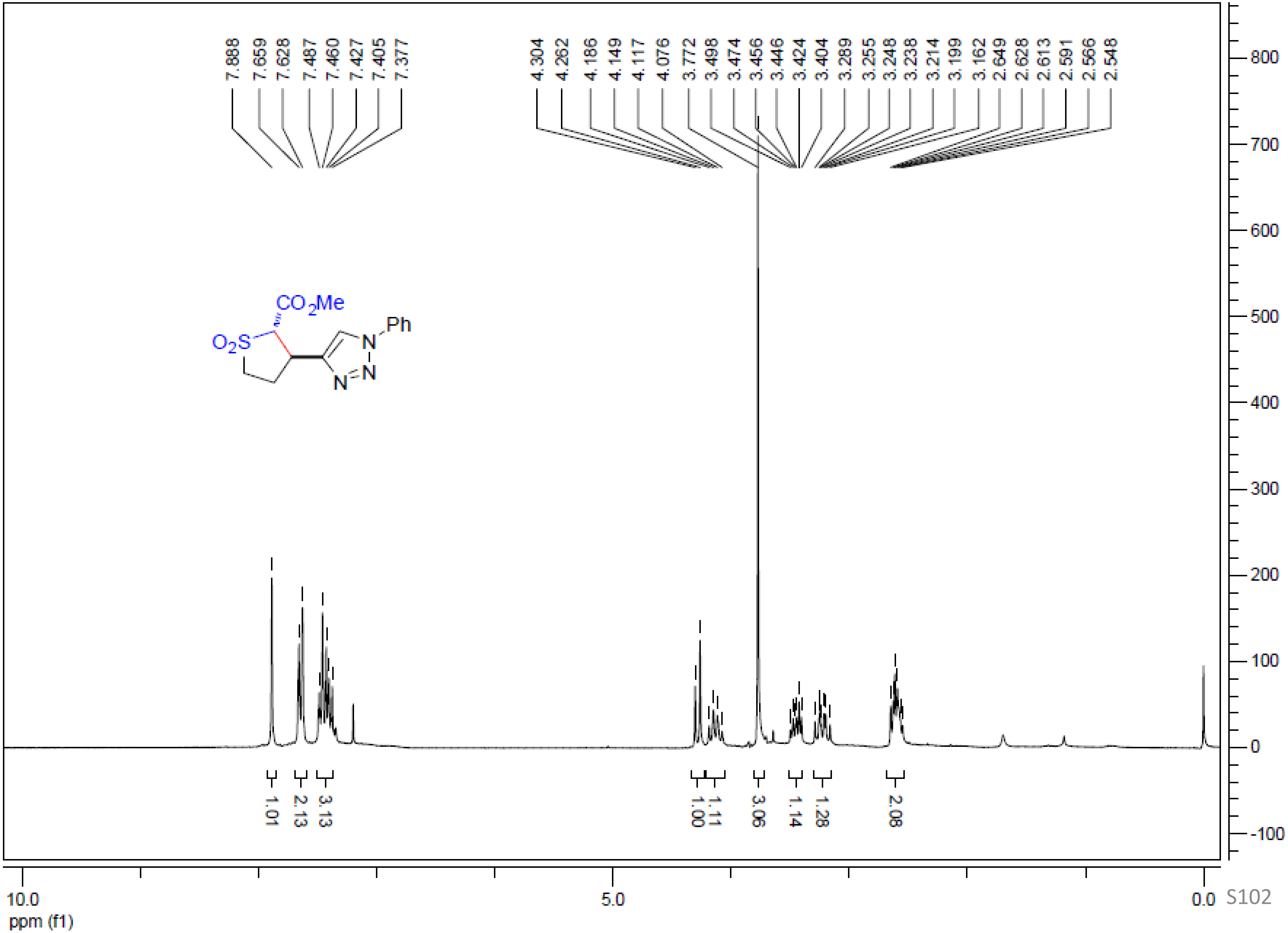

methyl 3-(1-phenyl-1H-1,2,3-triazol-4-yl)tetrahydrothiophene-2-carboxylate 1,1-dioxide (**2n**)

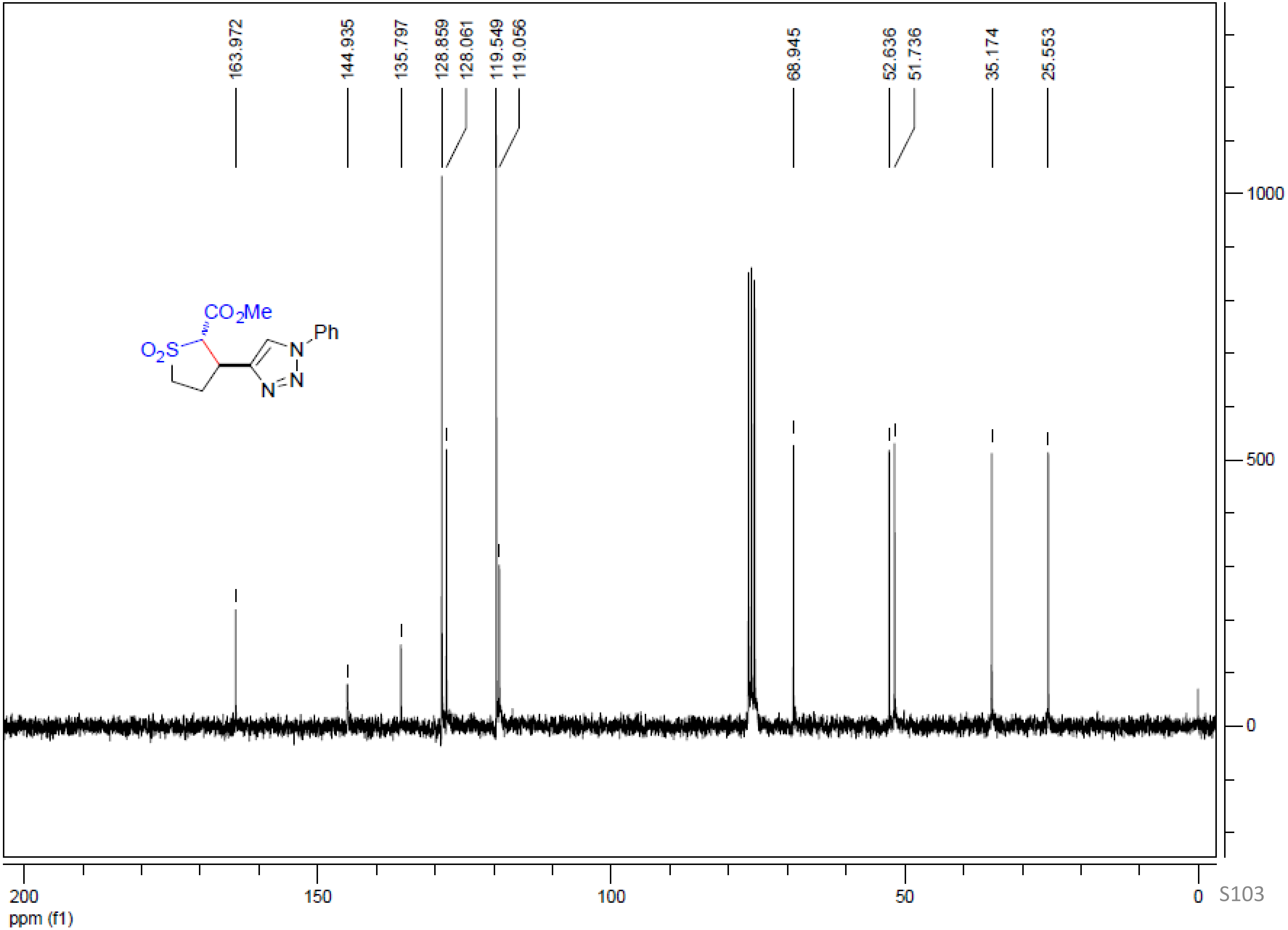

methyl 3-(1-phenyl-1H-1,2,3-triazol-4-yl)tetrahydrothiophene-2-carboxylate 1,1-dioxide (**2n**)

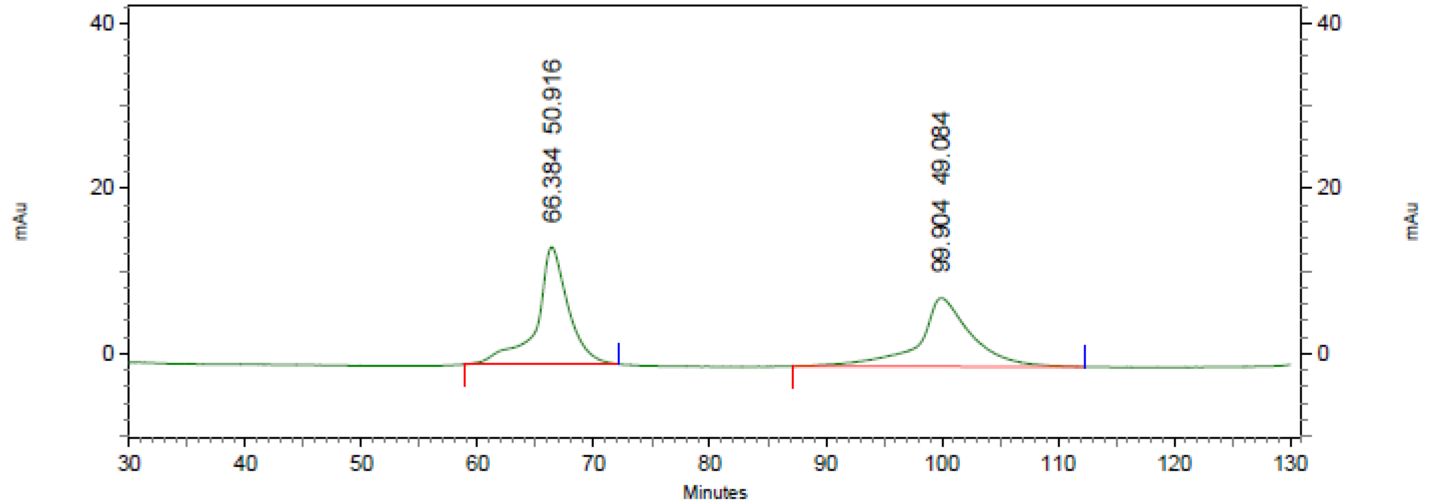

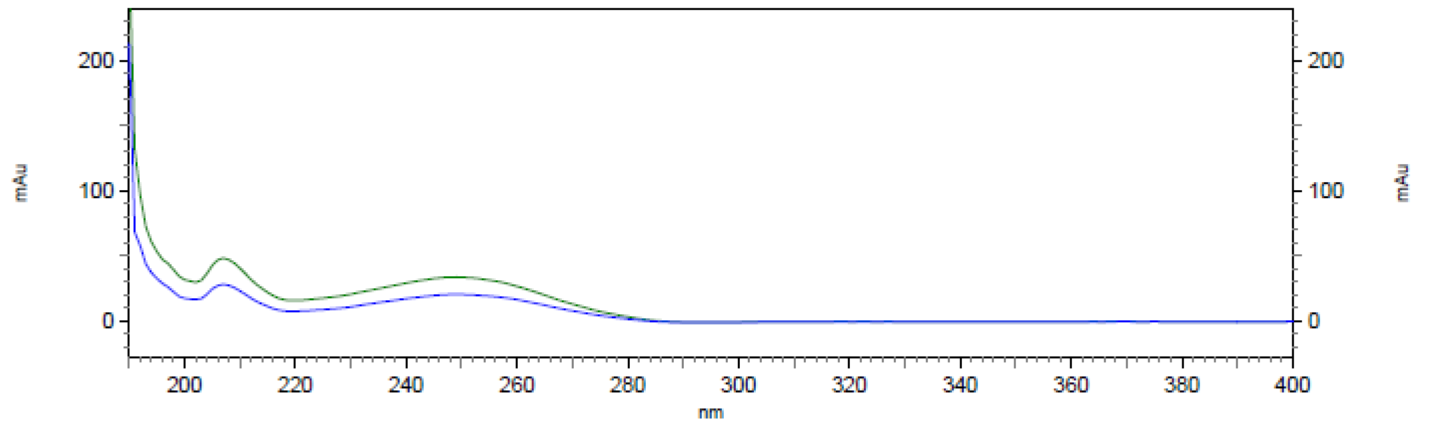

1: 245 nm, 4 nm Results

Pk #	Retention Time	Area Percent
1	66.384	50.916
2	99.904	49.084
Totals		100.000

methyl 3-(1-phenyl-1H-1,2,3-triazol-4-yl)tetrahydrothiophene-2-carboxylate 1,1-dioxide (**2n**)

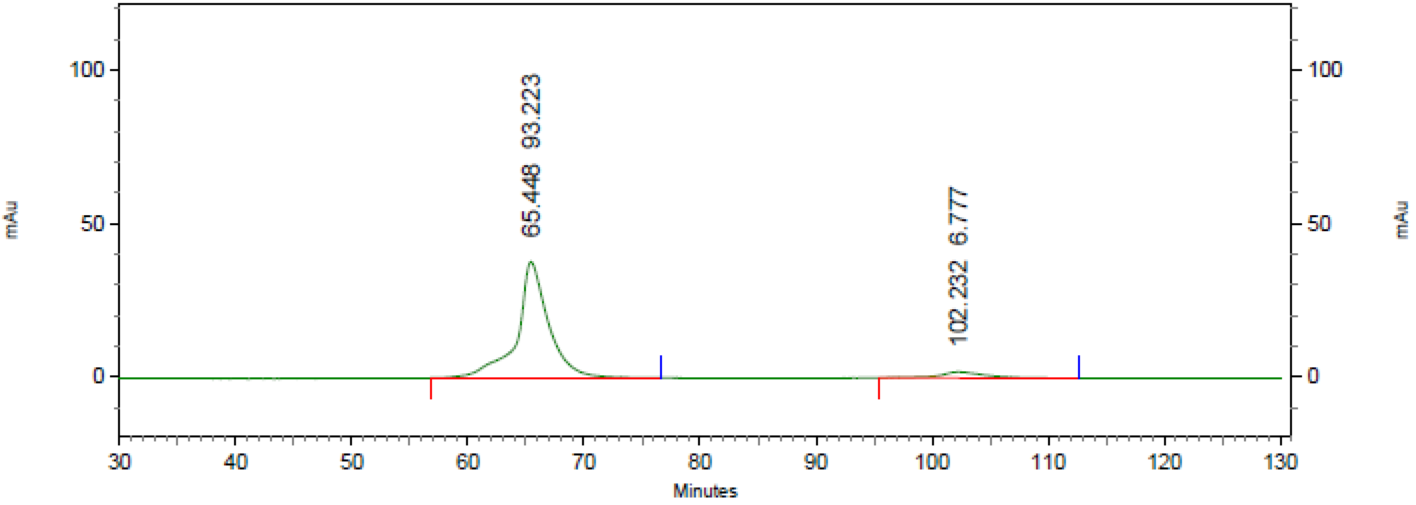

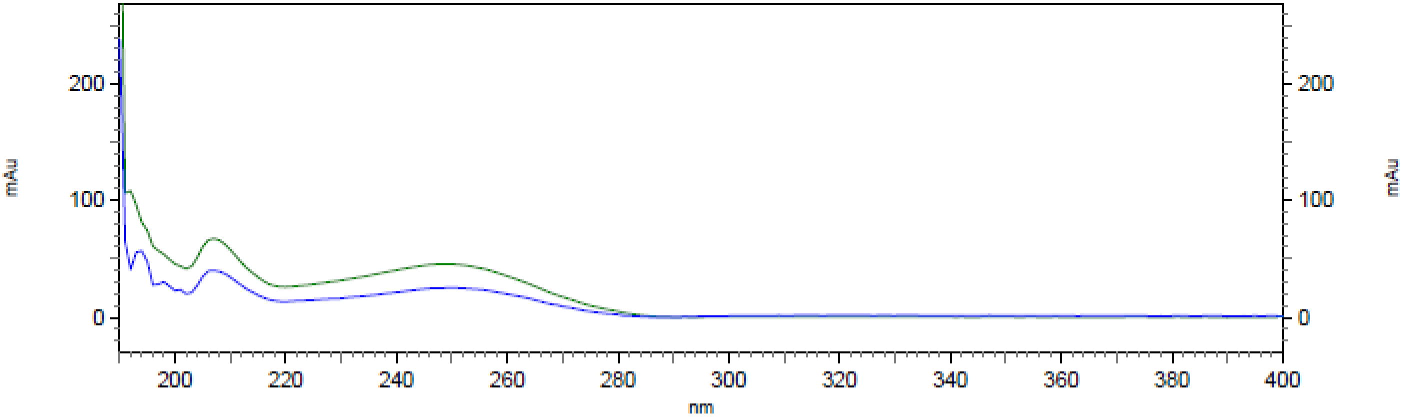

1: 244 nm, 4 nm Results

Pk #	Retention Time	Area Percent
1	65.448	93.223
2	102.232	6.777
Totals		100.000

methyl 3-vinyltetrahydrothiophene-2-carboxylate 1,1-dioxide (**2o**)

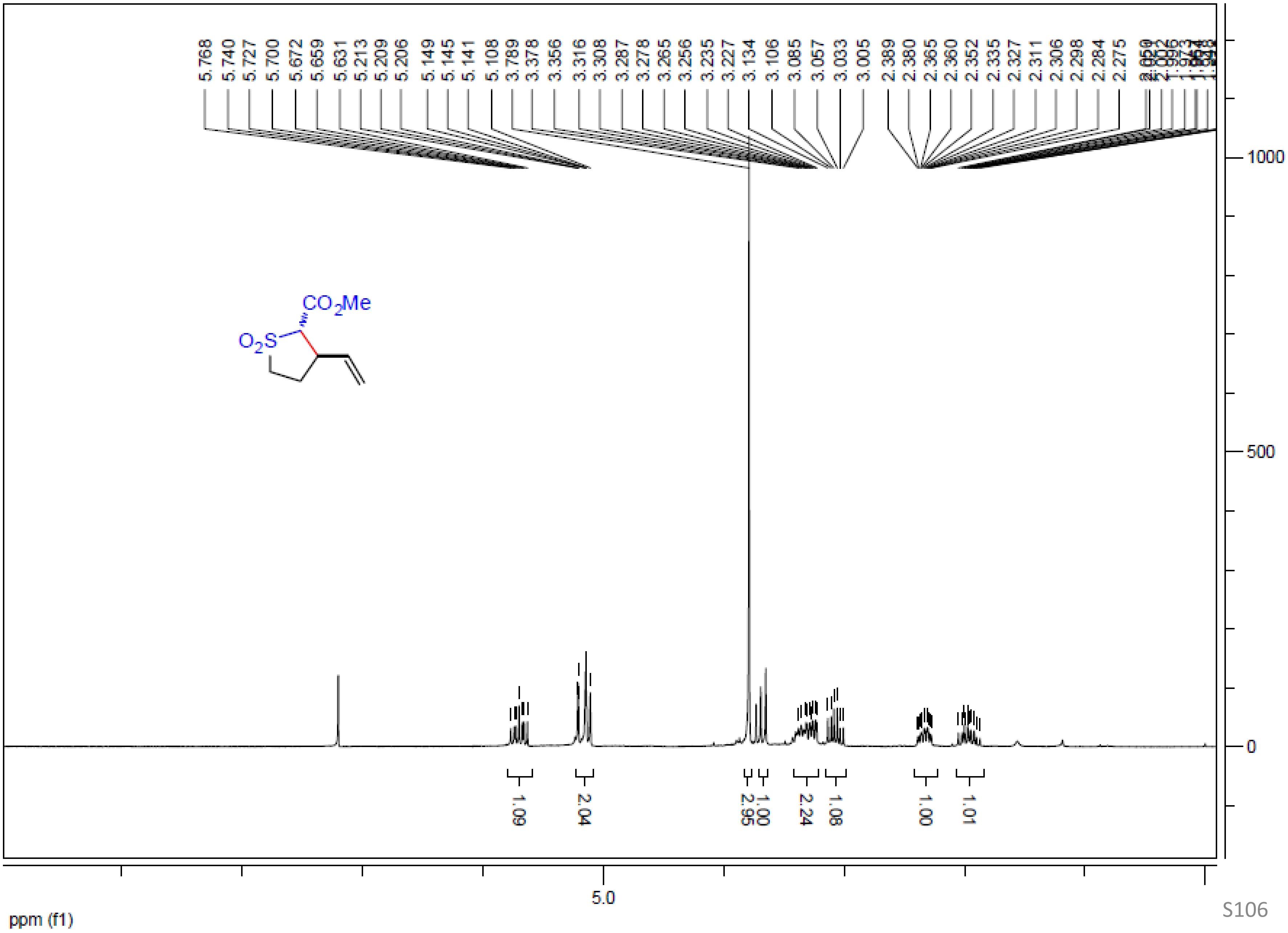

methyl 3-vinyltetrahydrothiophene-2-carboxylate 1,1-dioxide (**2o**)

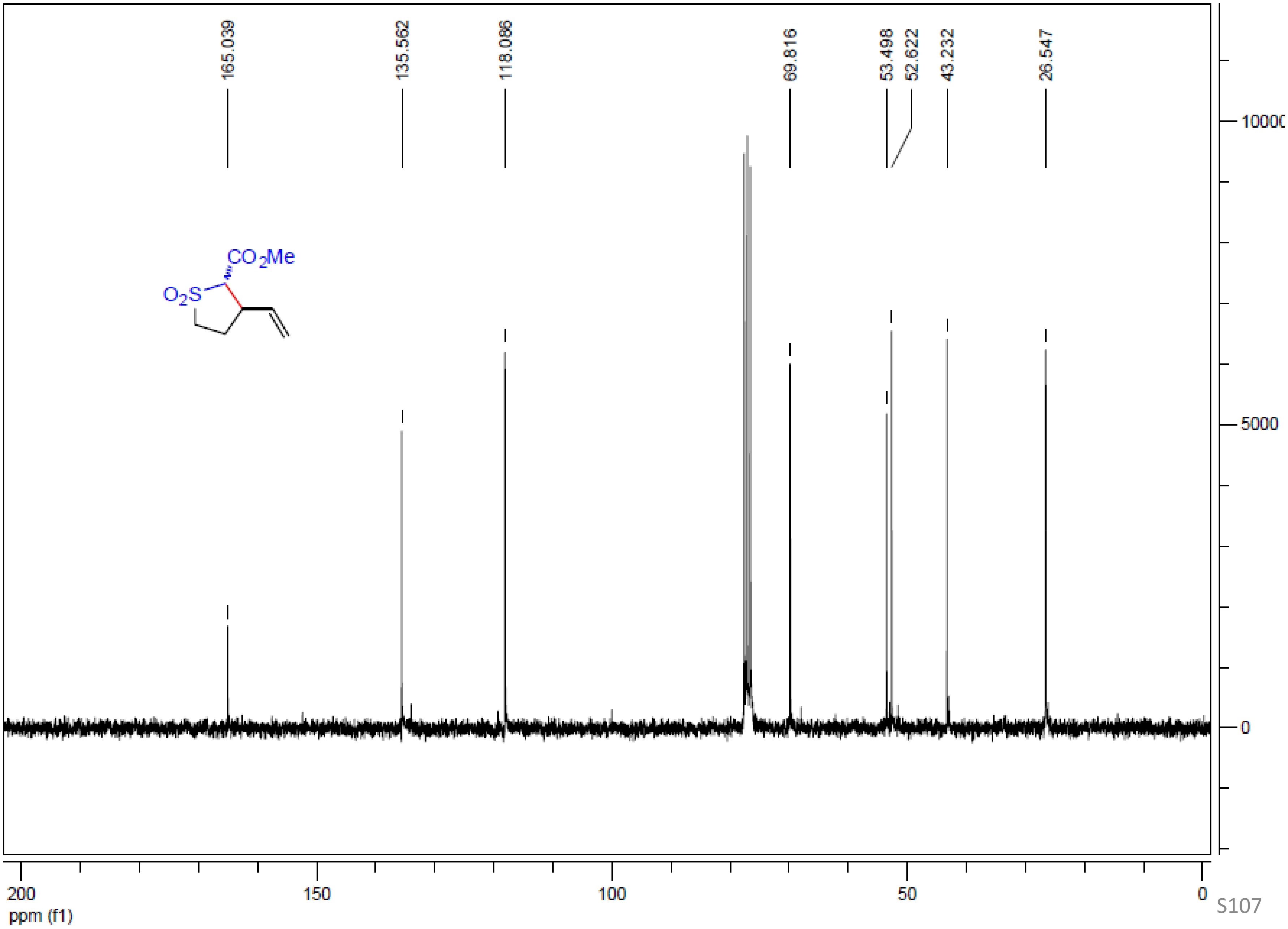

methyl 3-vinyltetrahydrothiophene-2-carboxylate 1,1-dioxide (**2o**)

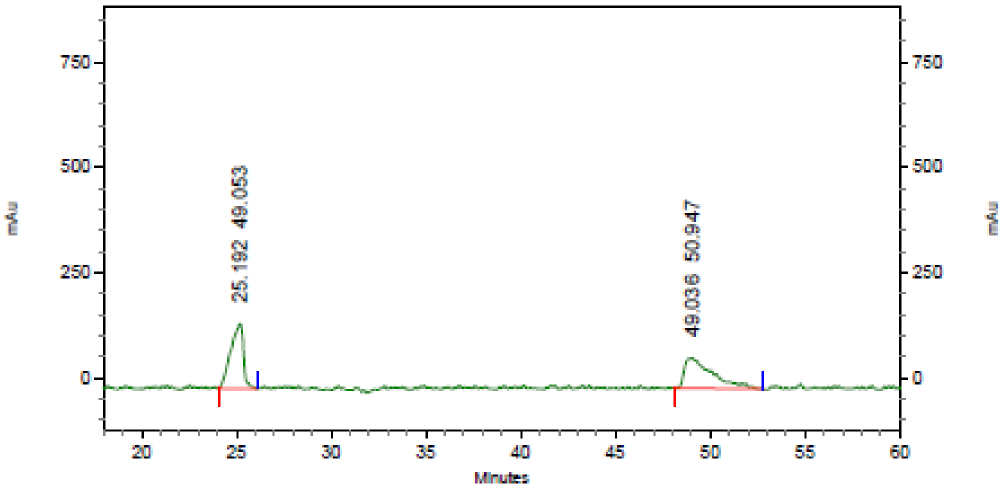

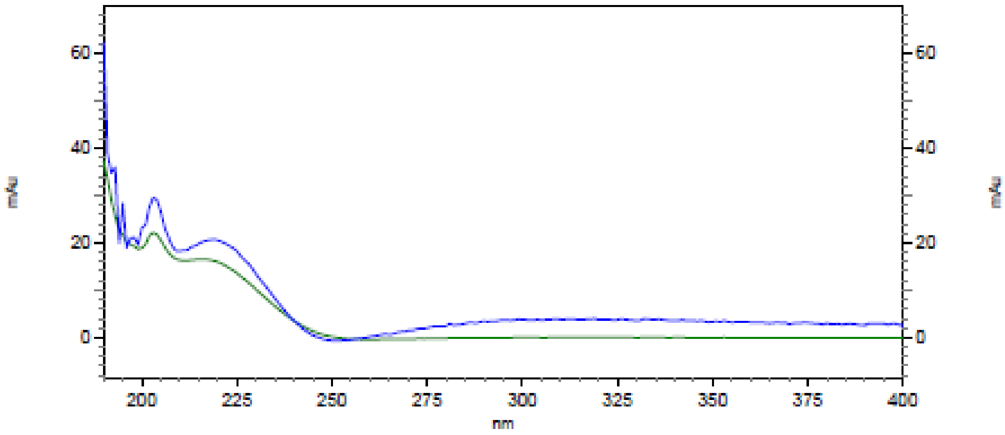

2: 213 nm, 4 nm
Results

Name	Retention Time	Area Percent	Pk #
	25.192	49.053	1
	49.036	50.947	2

Totals			
		100.000	

methyl 3-vinyltetrahydrothiophene-2-carboxylate 1,1-dioxide (**2o**)

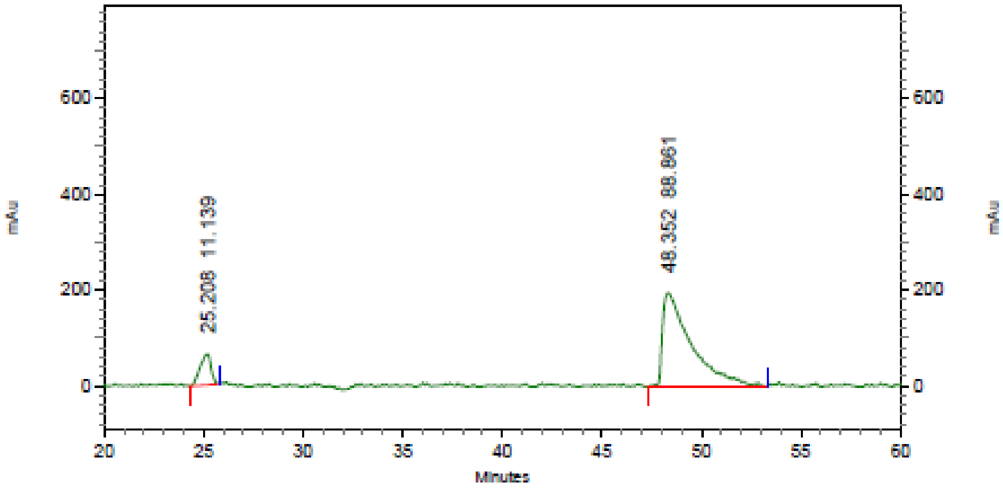

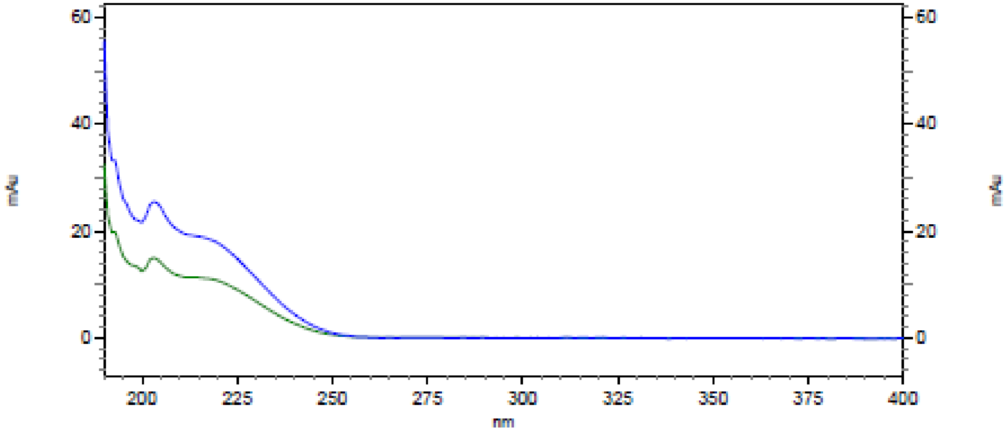

2: 213 nm, 4 nm

Results

Name	Retention Time	Area Percent	Pk #
	25.208	11.139	1
	48.352	88.861	2
Totals		100.000	

methyl 3-(prop-1-en-2-yl)tetrahydrothiophene-2-carboxylate 1,1-dioxide (**2p**)

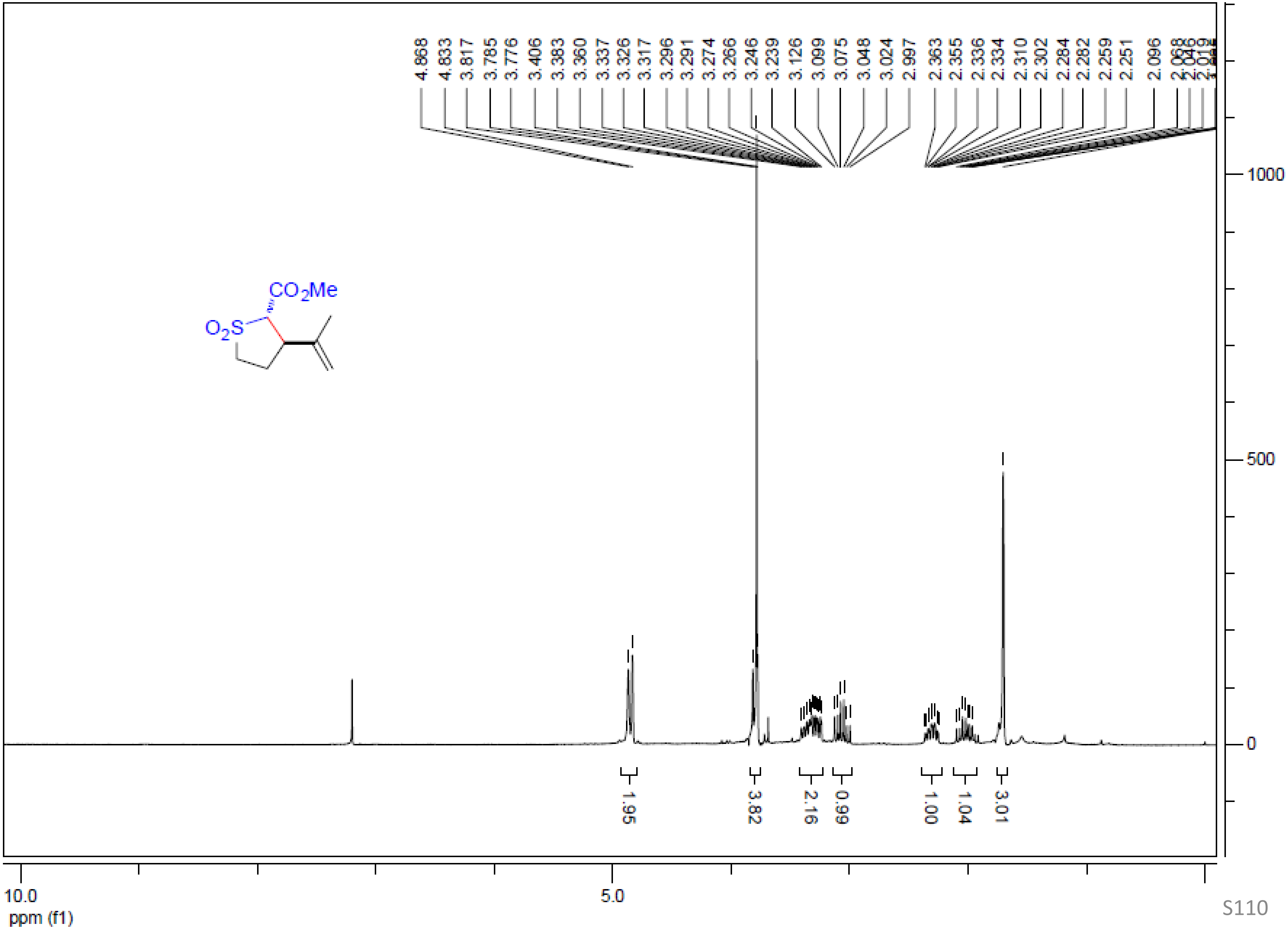

methyl 3-(prop-1-en-2-yl)tetrahydrothiophene-2-carboxylate 1,1-dioxide (**2p**)

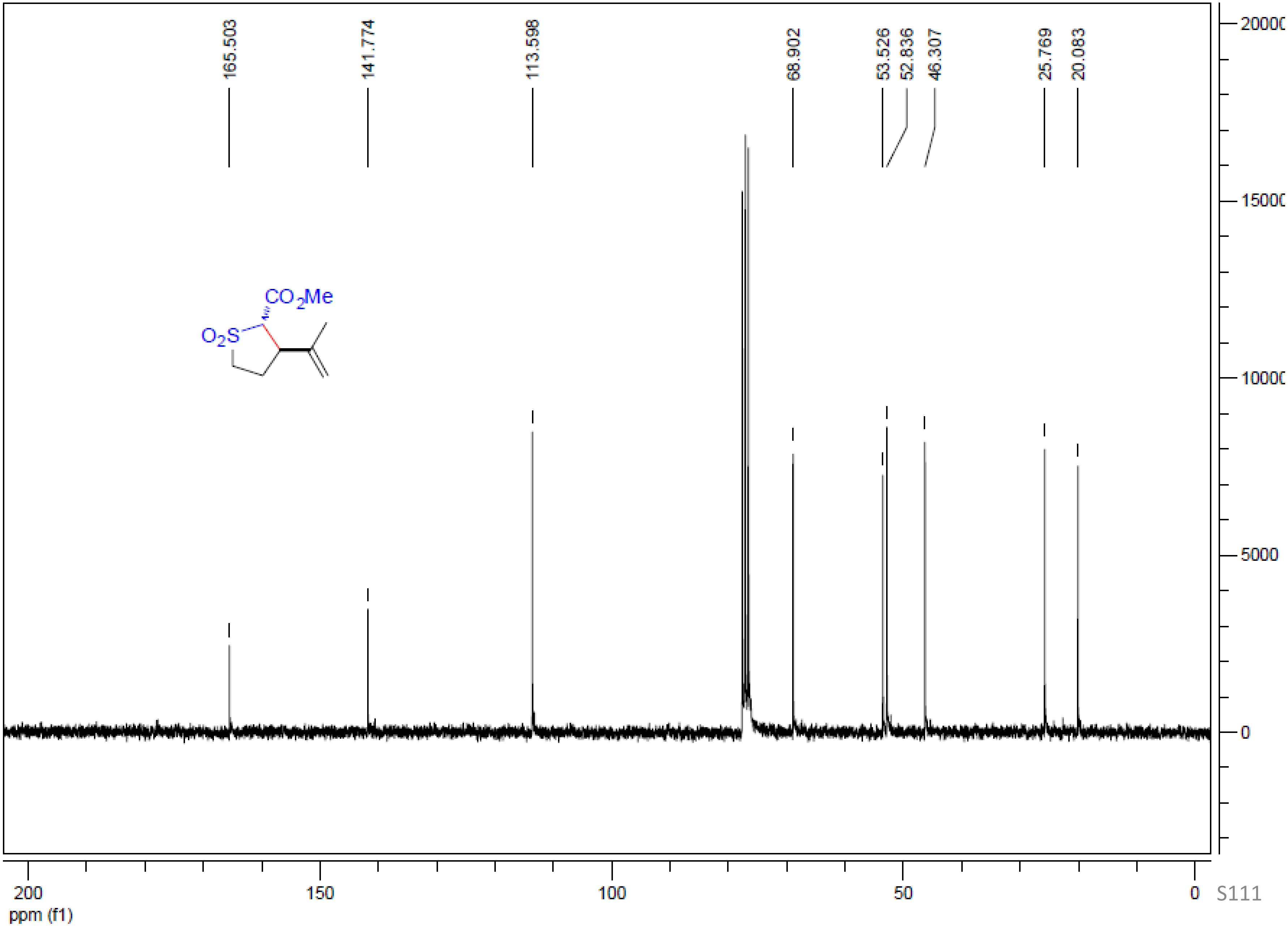

methyl 3-(prop-1-en-2-yl)tetrahydrothiophene-2-carboxylate 1,1-dioxide (**2p**)

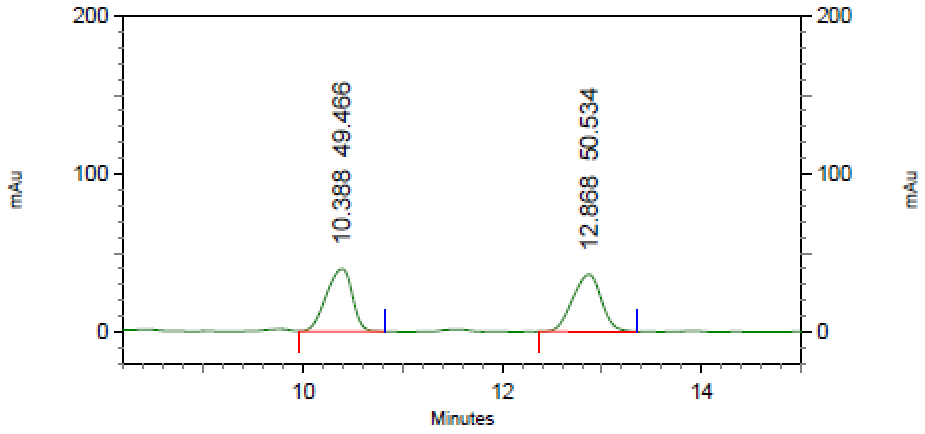

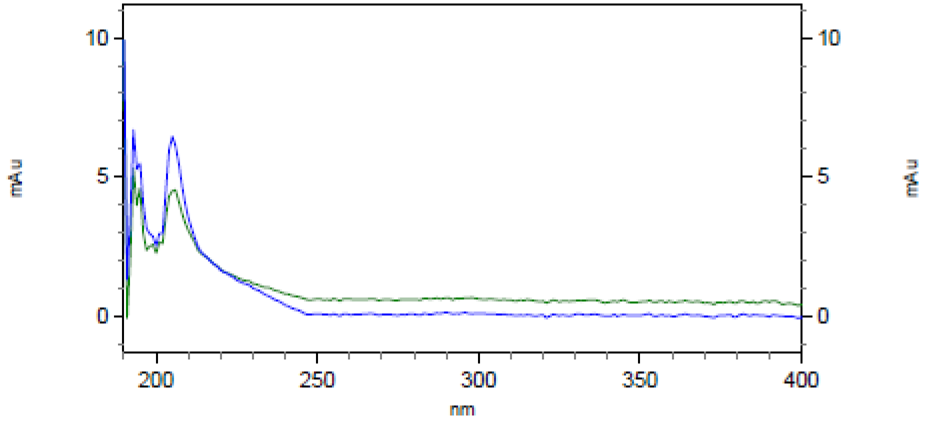

2: 227 nm, 4 nm

Results

Name	Retention Time	Area Percent	Pk #
	10.388	49.466	1
	12.868	50.534	2
Totals		100.000	

methyl 3-(prop-1-en-2-yl)tetrahydrothiophene-2-carboxylate 1,1-dioxide (**2p**)

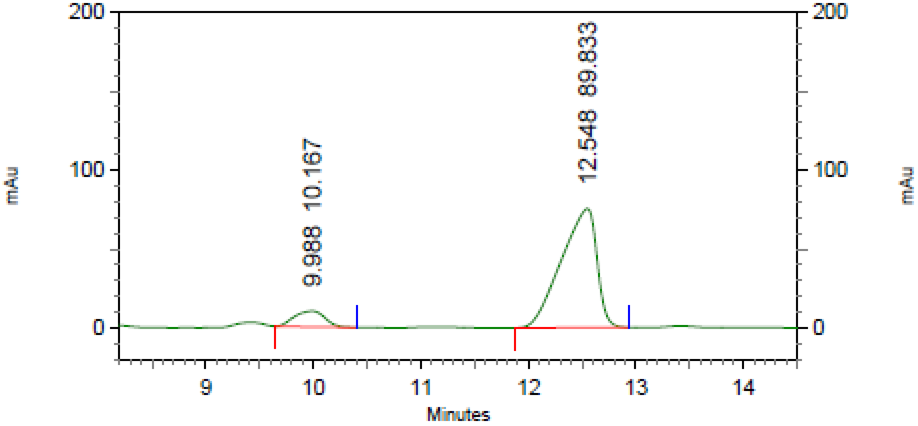

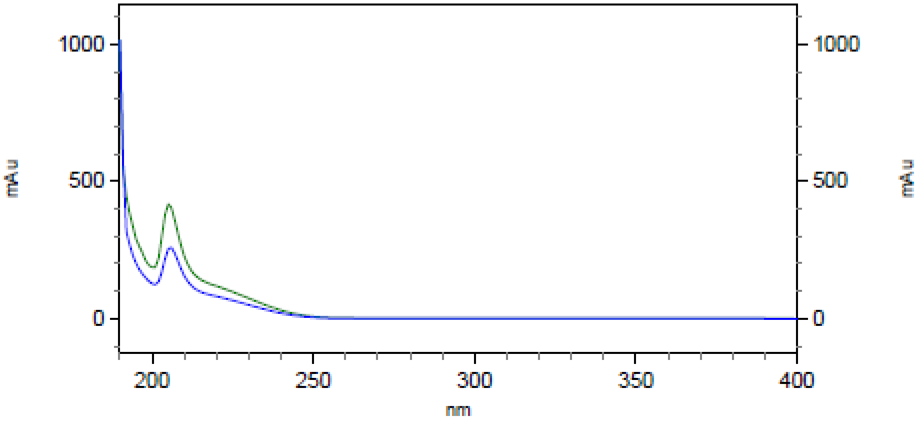

2: 227 nm, 4 nm

Results

Name	Retention Time	Area Percent	Pk #
	9.988	10.167	1
	12.548	89.833	2

Totals	
100.000	

methyl 3-(propa-1,2-dien-1-yl)tetrahydrothiophene-2-carboxylate 1,1-dioxide (**2q**)

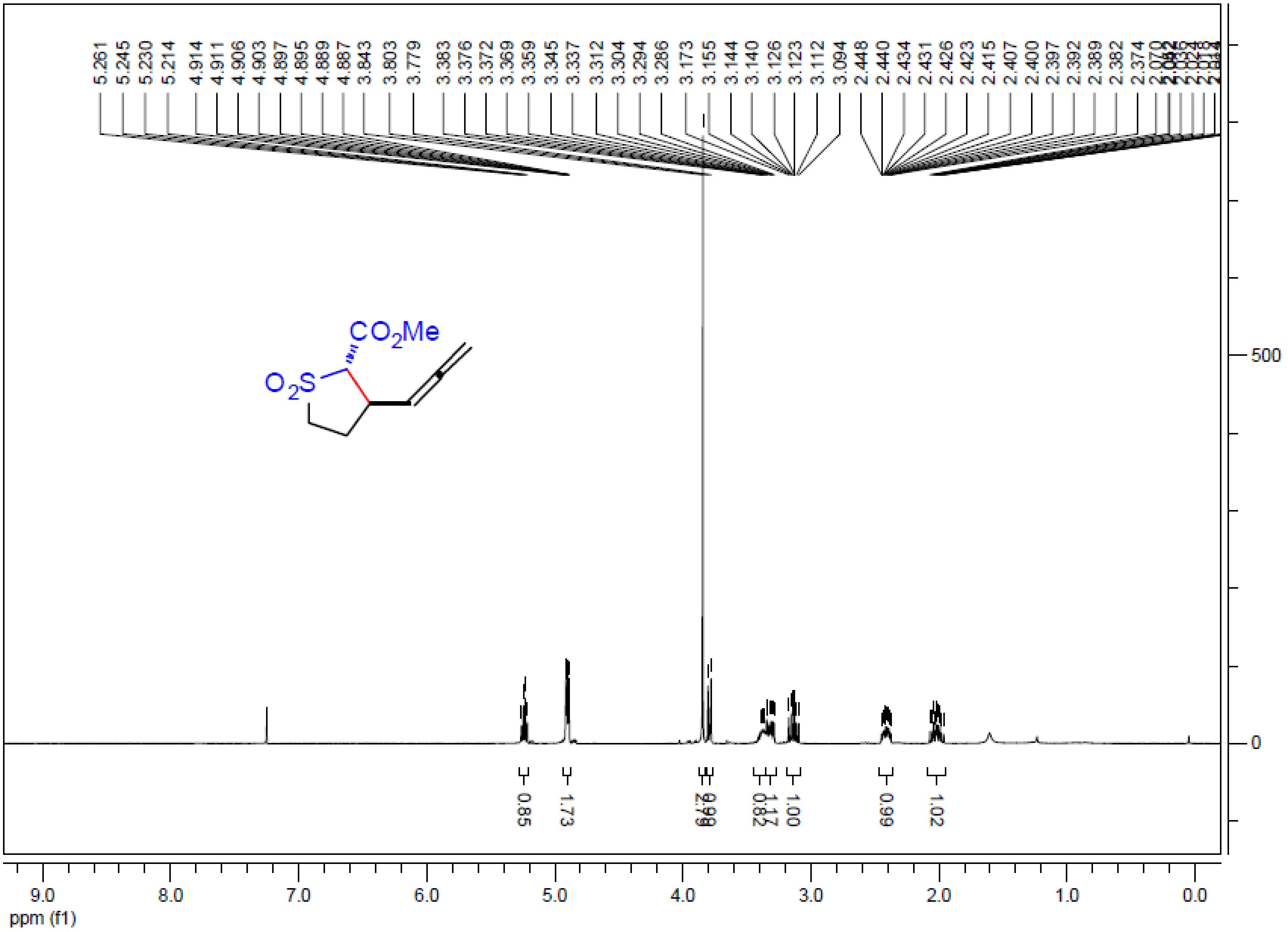

methyl 3-(propa-1,2-dien-1-yl)tetrahydrothiophene-2-carboxylate 1,1-dioxide (**2q**)

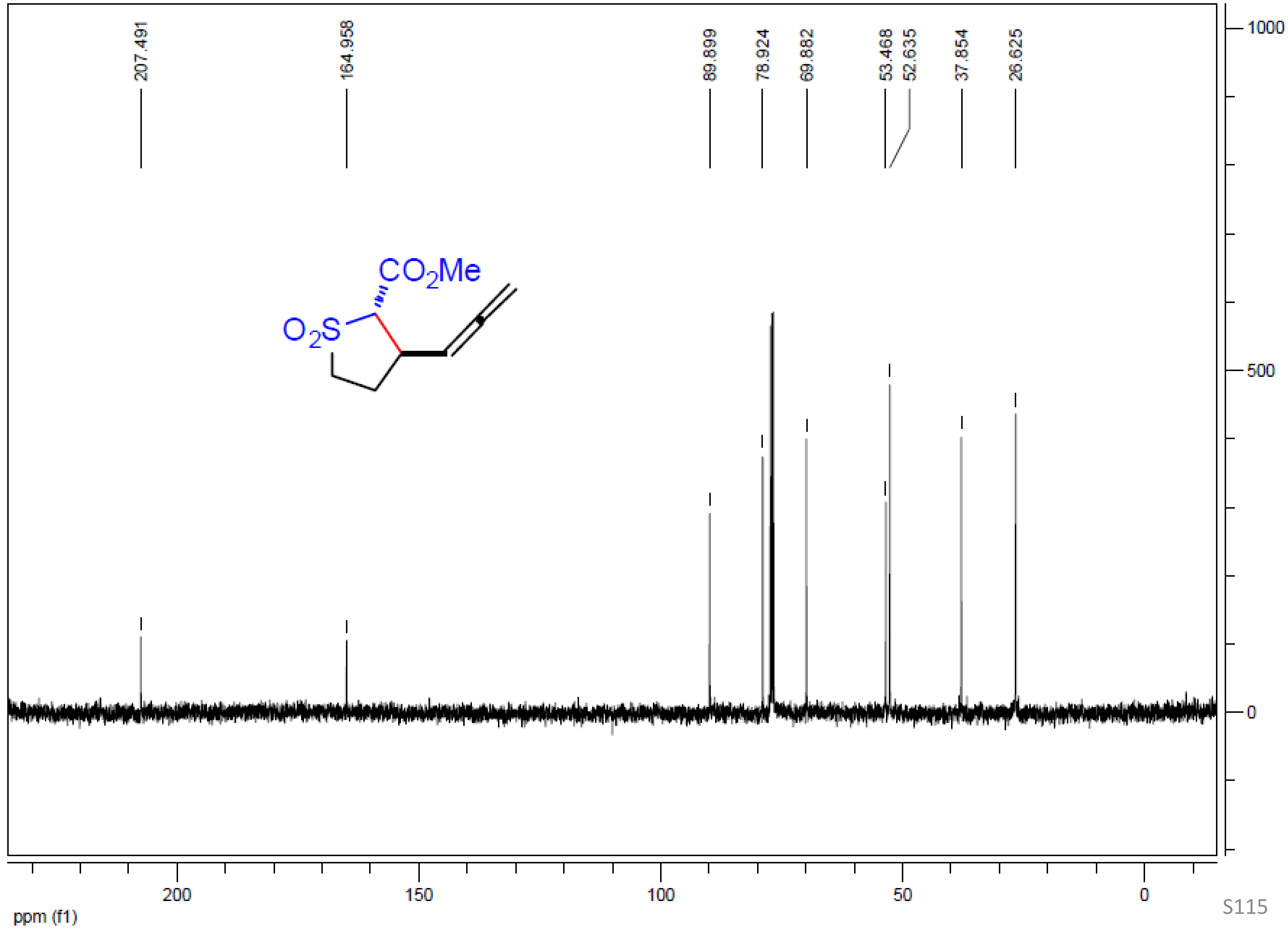

methyl 3-(propa-1,2-dien-1-yl)tetrahydrothiophene-2-carboxylate 1,1-dioxide (**2q**)

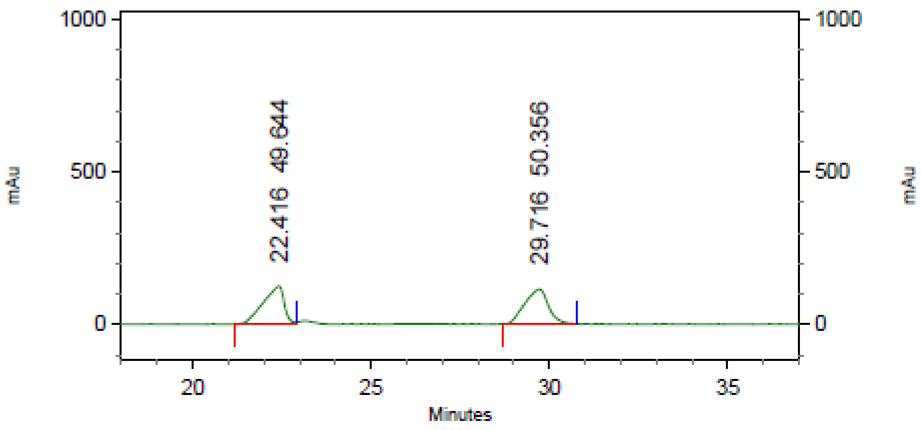

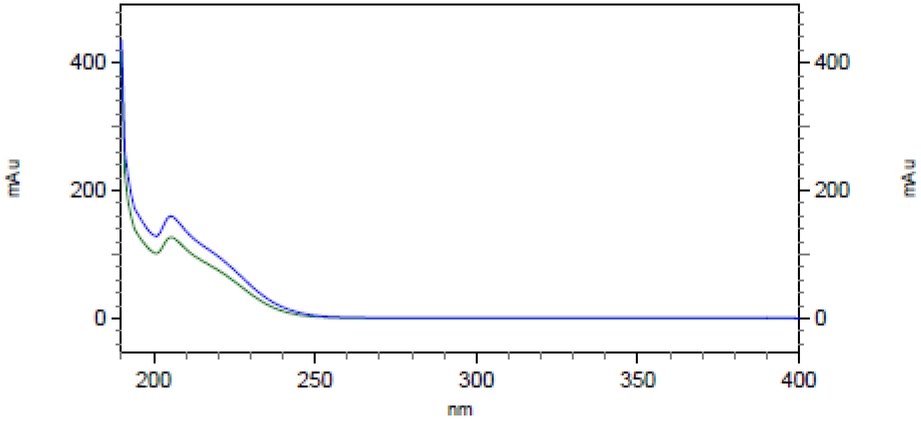

5: 220 nm, 4 nm
Results

Name	Retention Time	Area Percent	Pk #
	22.416	49.644	1
	29.716	50.356	2
Totals		100.000	

methyl 3-(propa-1,2-dien-1-yl)tetrahydrothiophene-2-carboxylate 1,1-dioxide (**2q**)

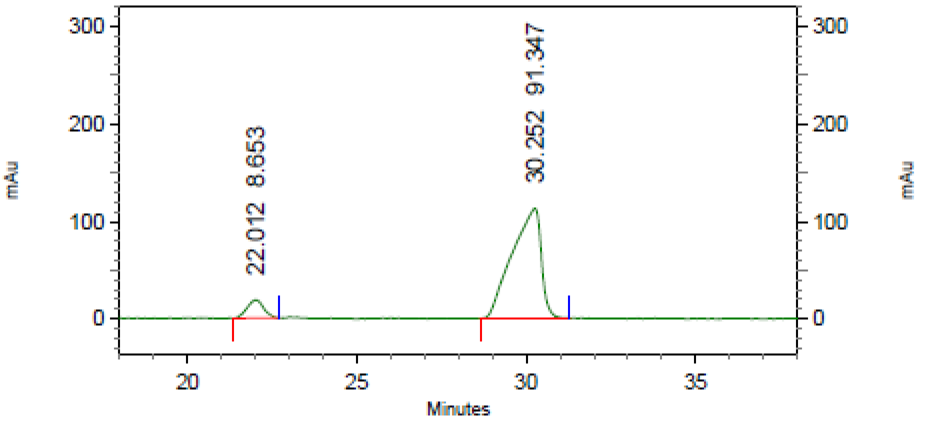

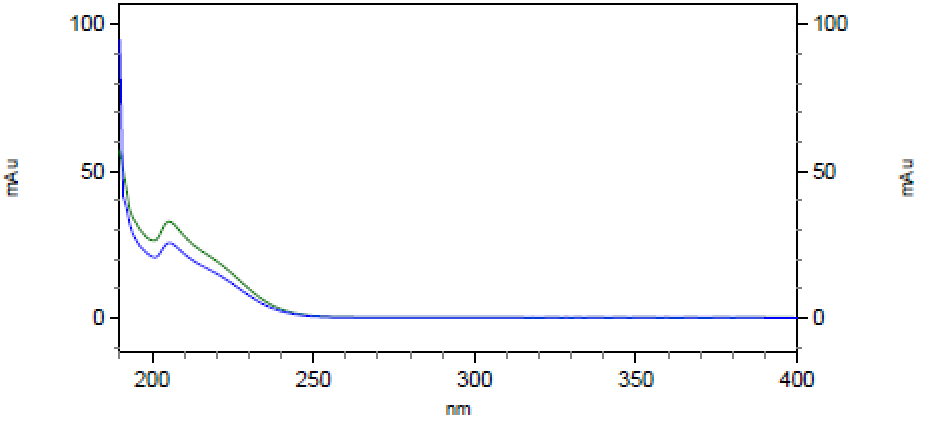

5: 220 nm, 4 nm

Results

Name	Retention Time	Area Percent	Pk #
	22.012	8.653	1
	30.252	91.347	2
Totals		100.000	

methyl (E)-3-(prop-1-en-1-yl)tetrahydrothiophene-2-carboxylate 1,1-dioxide (**2r**)

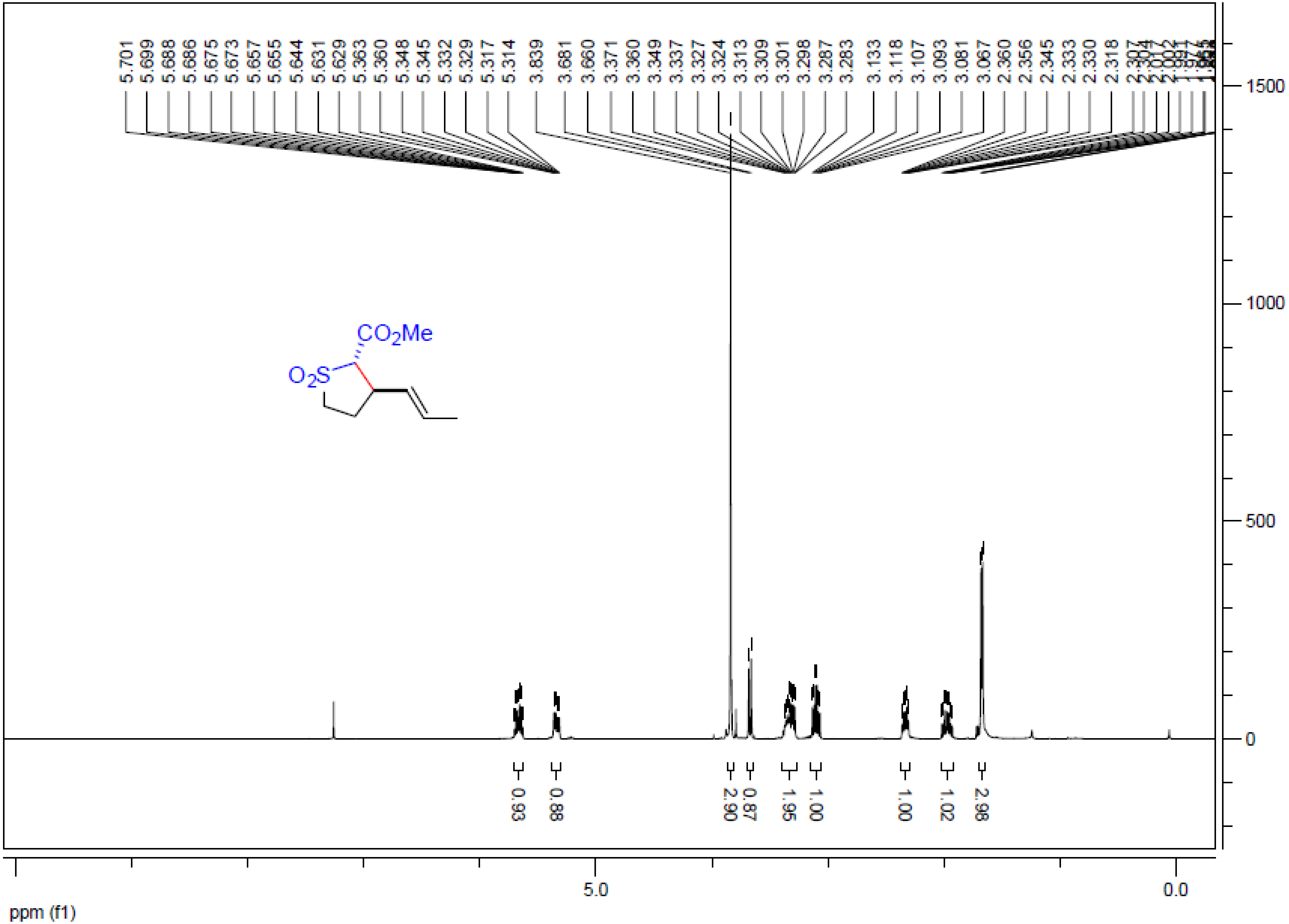

methyl (E)-3-(prop-1-en-1-yl)tetrahydrothiophene-2-carboxylate 1,1-dioxide (**2r**)

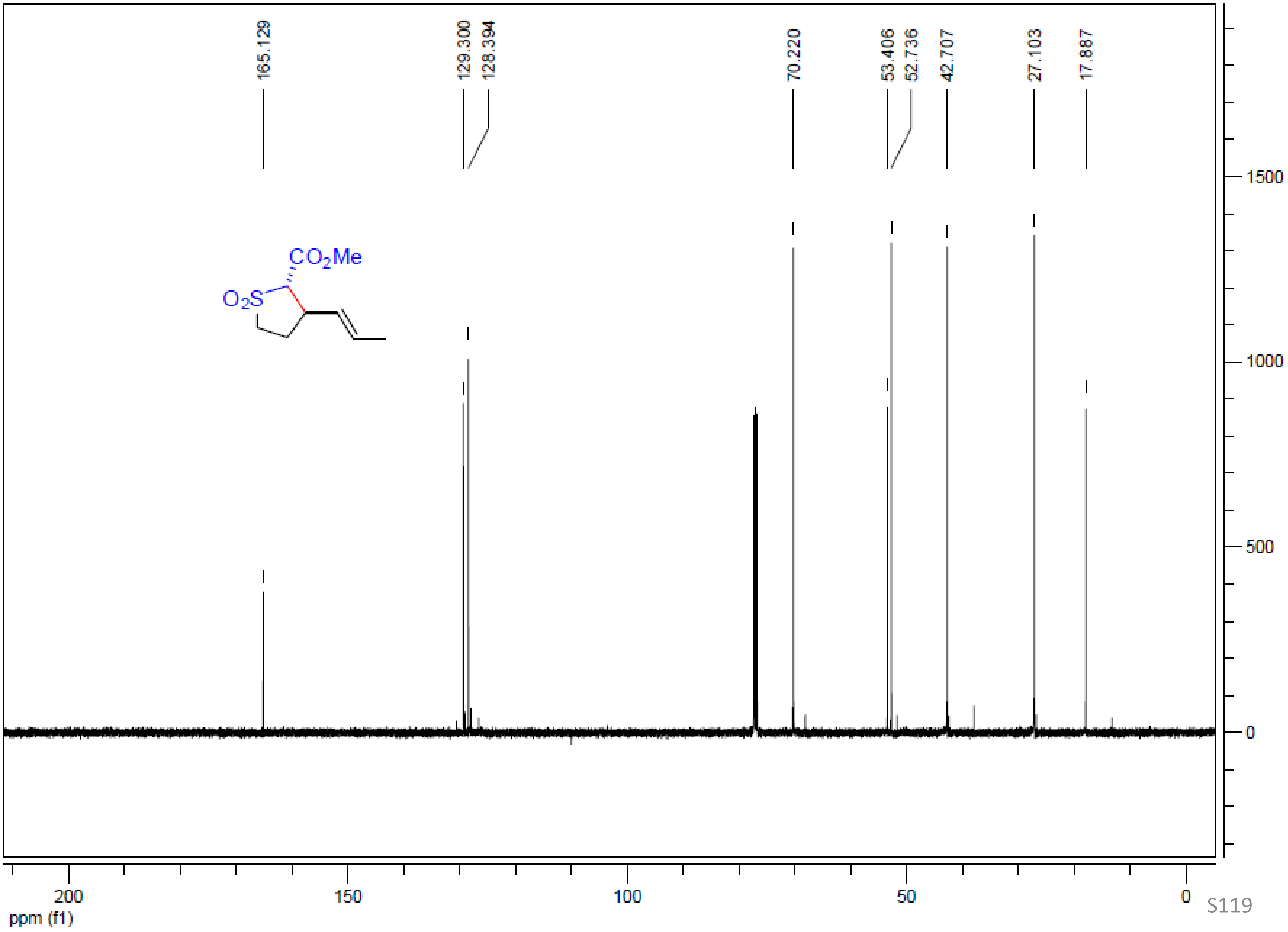

methyl (Z)-3-(prop-1-en-1-yl)tetrahydrothiophene-2-carboxylate 1,1-dioxide (**2s**)

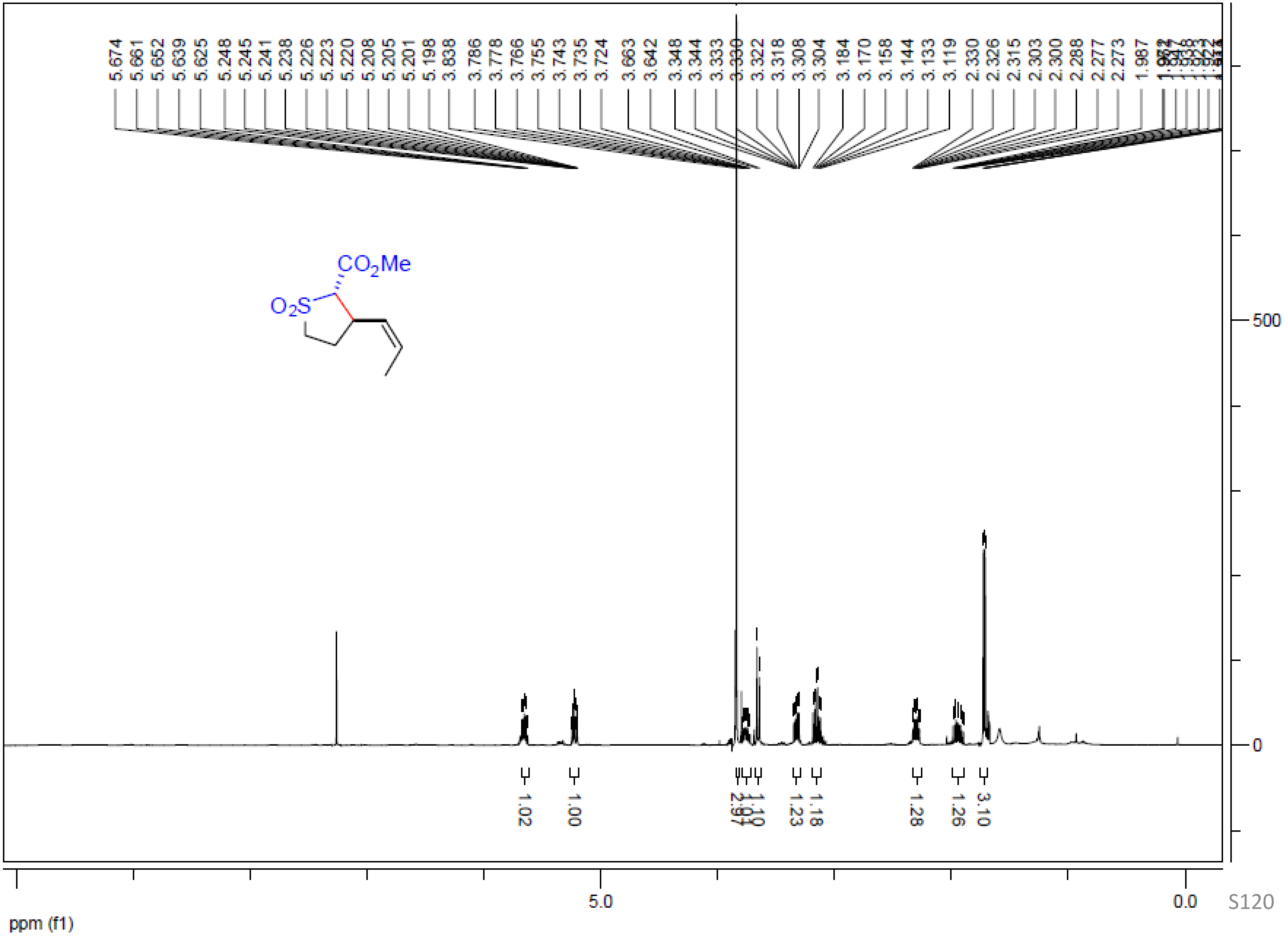

methyl (Z)-3-(prop-1-en-1-yl)tetrahydrothiophene-2-carboxylate 1,1-dioxide (**2s**)

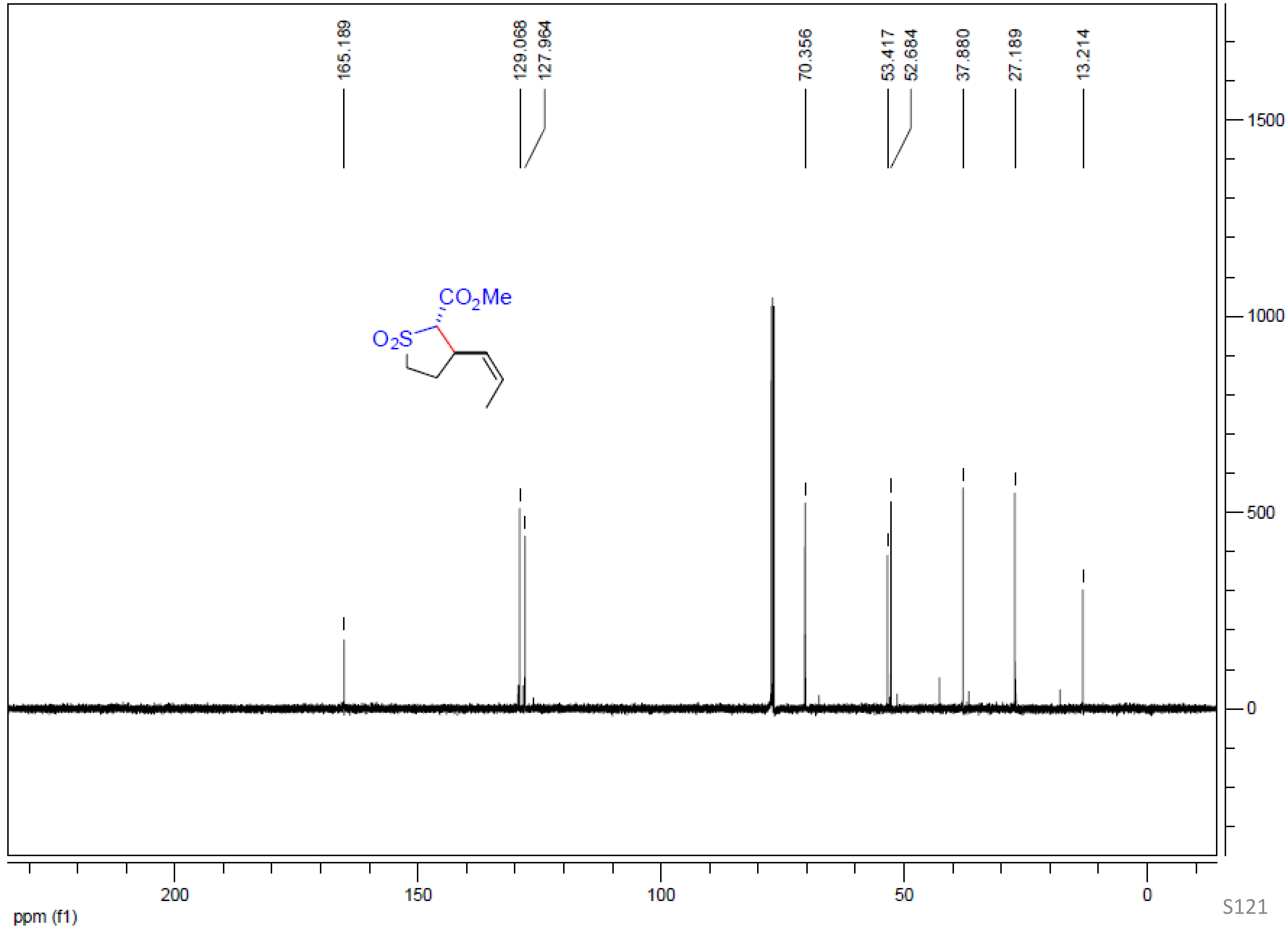

methyl 2-fluoro-3-(4-methoxyphenyl)tetrahydrothiophene-2-carboxylate 1,1-dioxide (**3ia**)

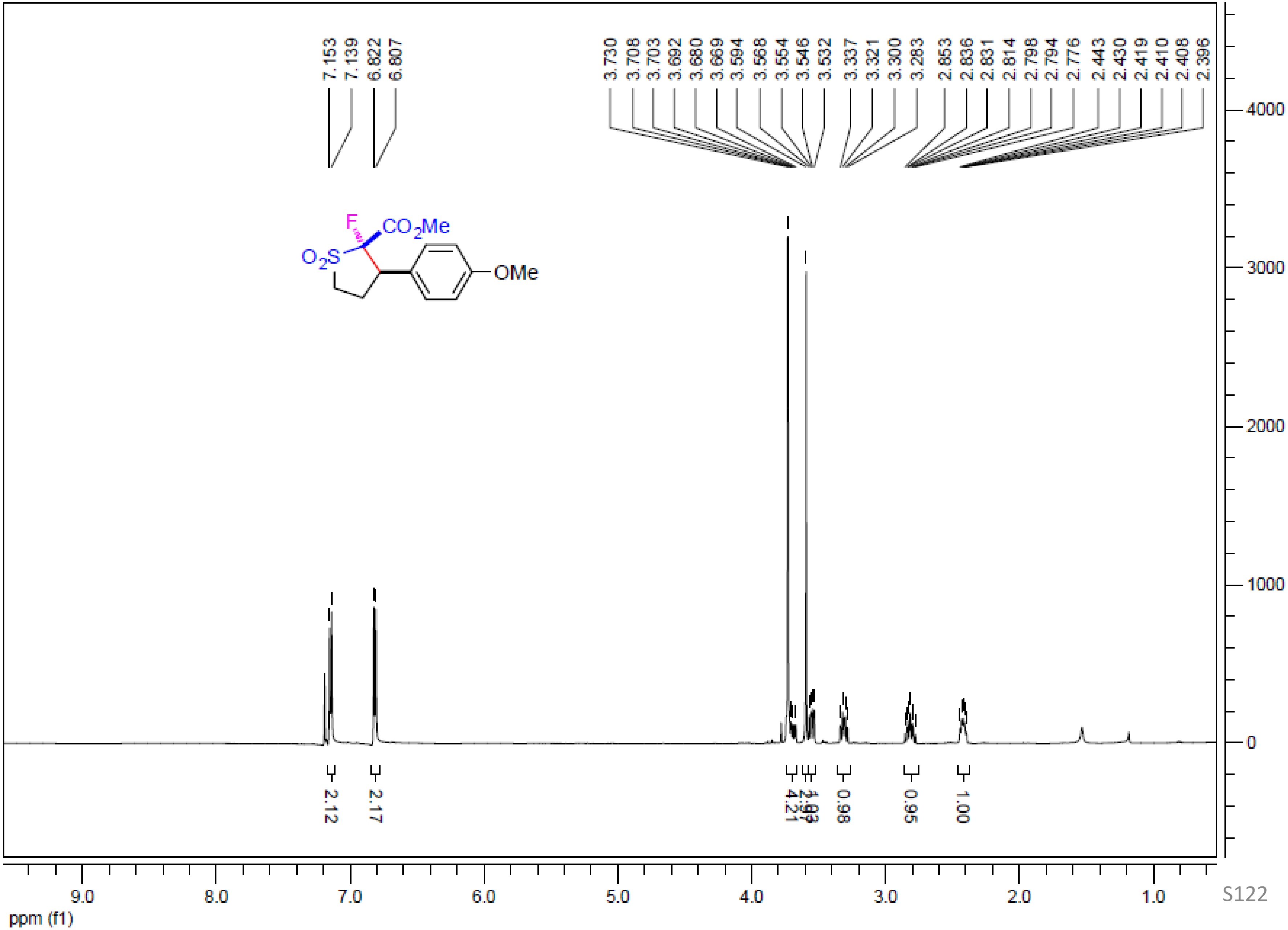

methyl 2-fluoro-3-(4-methoxyphenyl)tetrahydrothiophene-2-carboxylate 1,1-dioxide (**3ia**)

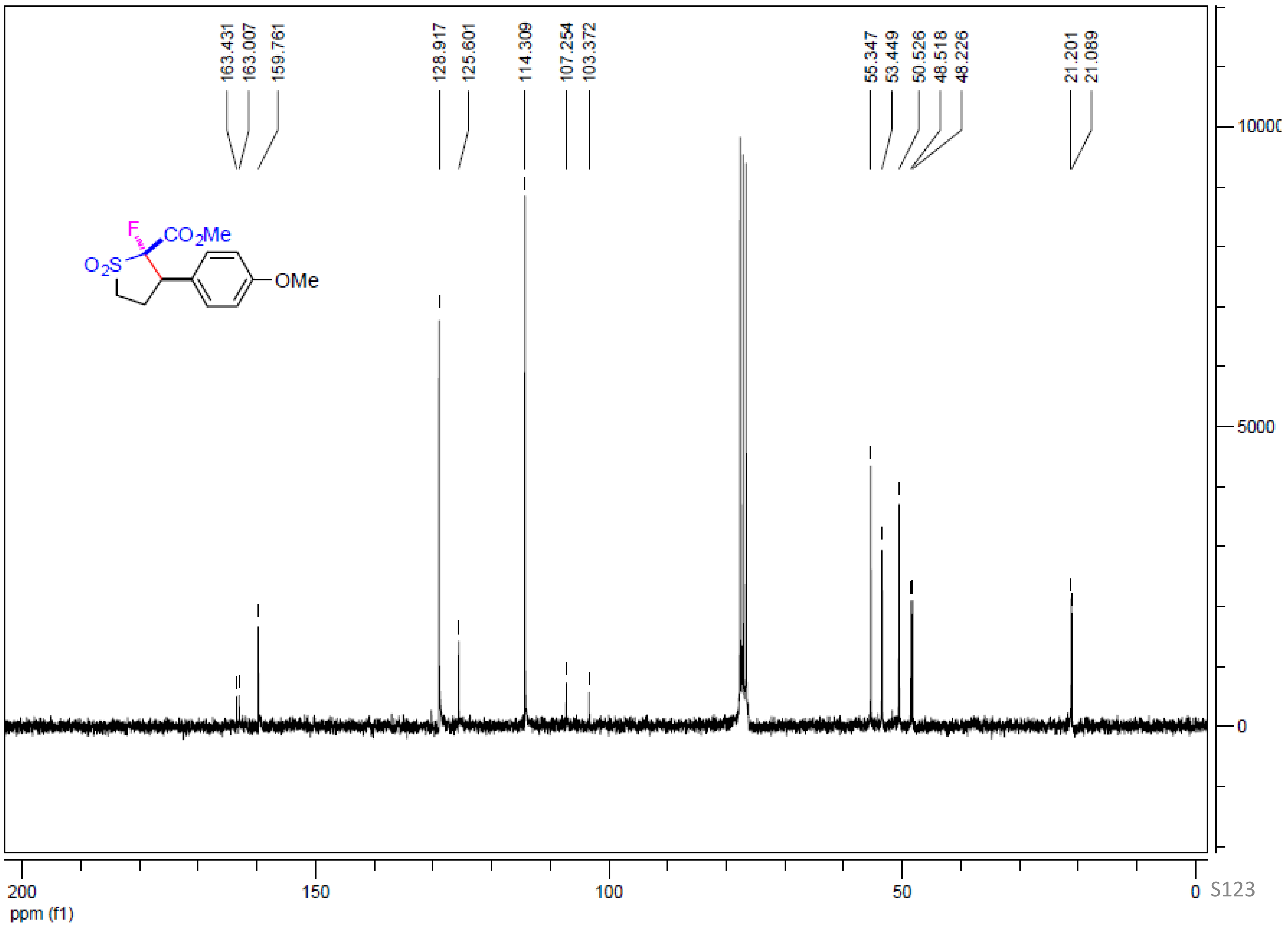

methyl 2-fluoro-3-(4-methoxyphenyl)tetrahydrothiophene-2-carboxylate 1,1-dioxide (**3ia**)

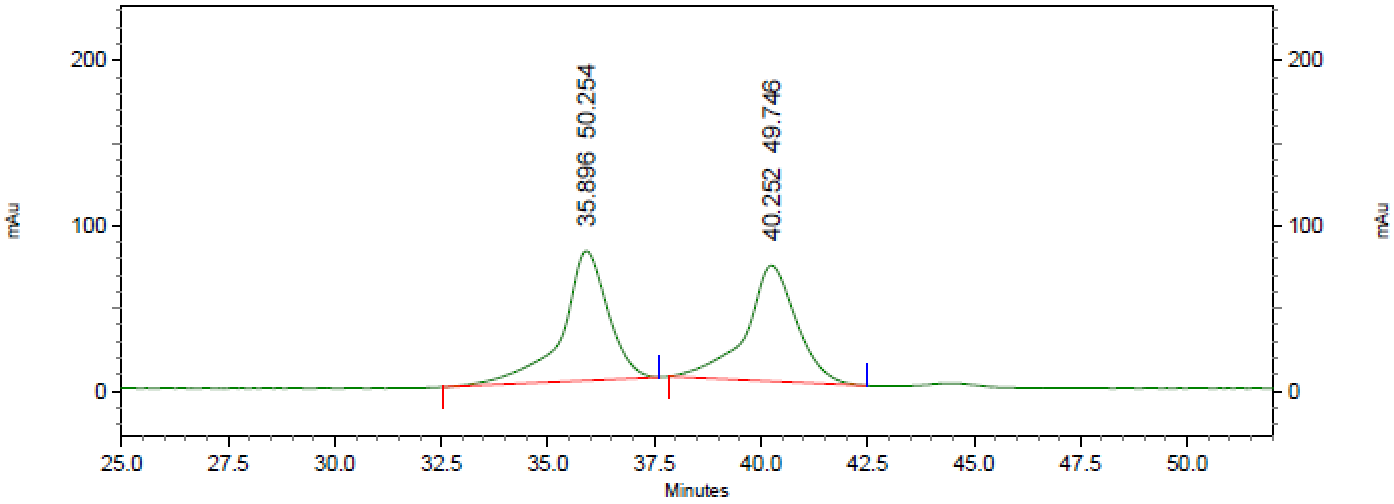

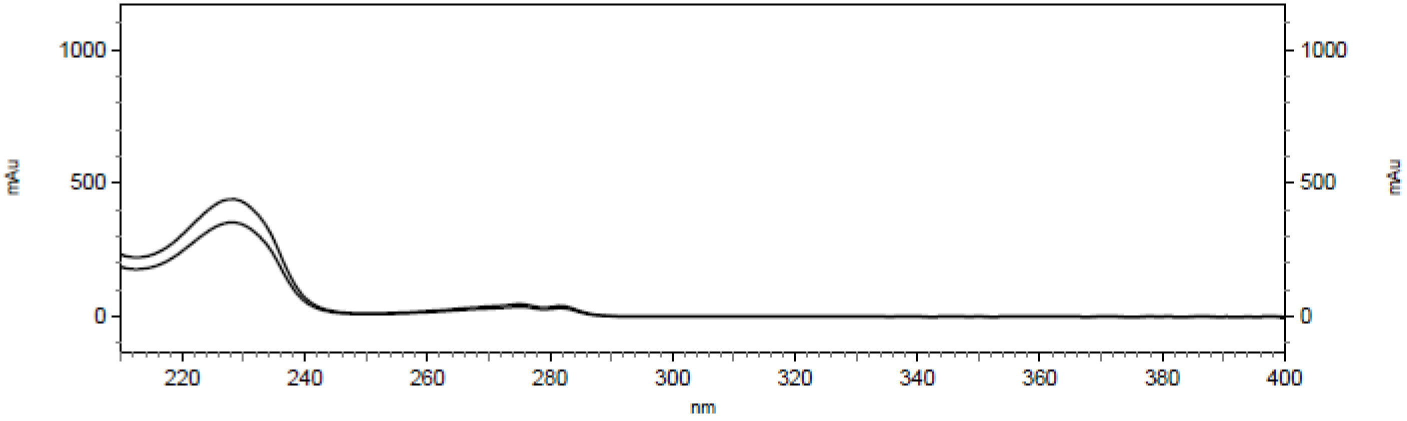

11: 224 nm, 4 nm Results

Pk #	Retention Time	Area Percent
1	35.896	50.254
2	40.252	49.746
Totals		100.000

methyl 2-fluoro-3-(4-methoxyphenyl)tetrahydrothiophene-2-carboxylate 1,1-dioxide (**3ia**)

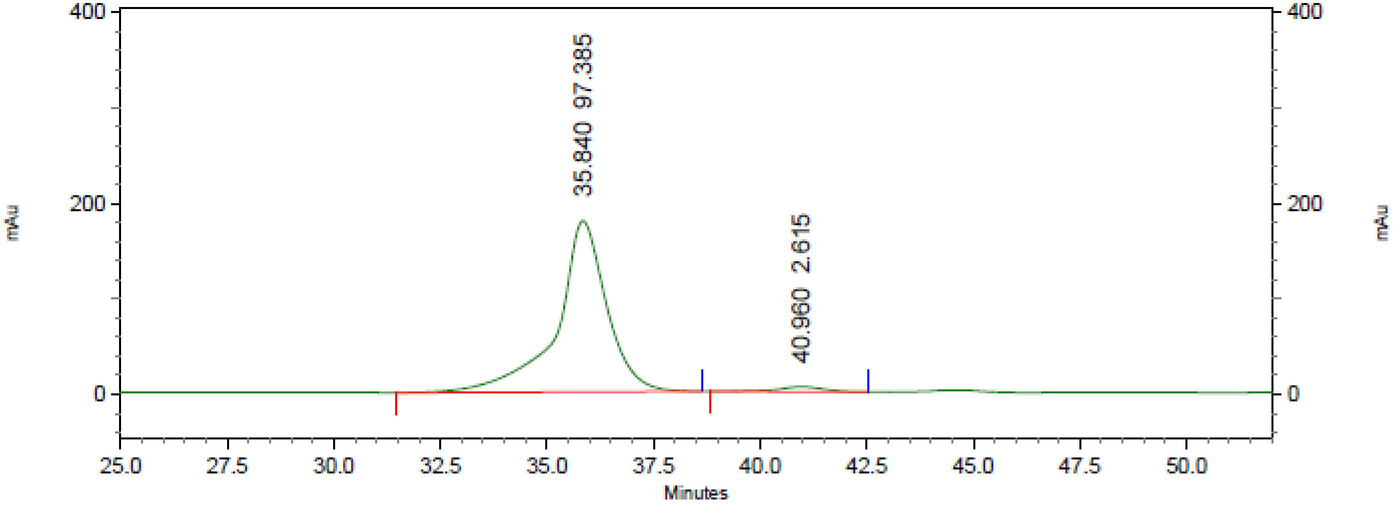

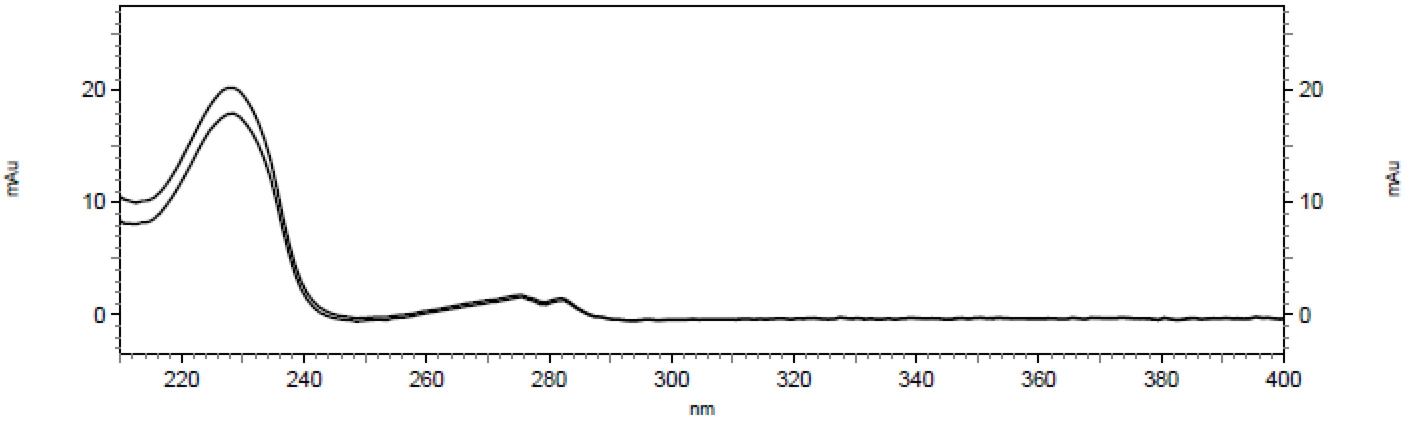

8: 232 nm, 4 nm Results

Pk #	Retention Time	Area Percent
1	35.840	97.385
2	40.960	2.615
Totals		100.000

(2R,3R)-methyl 3-(3,5-dichlorophenyl)-2-fluorotetrahydrothiophene-2-carboxylate 1,1-dioxide (**3ea**)

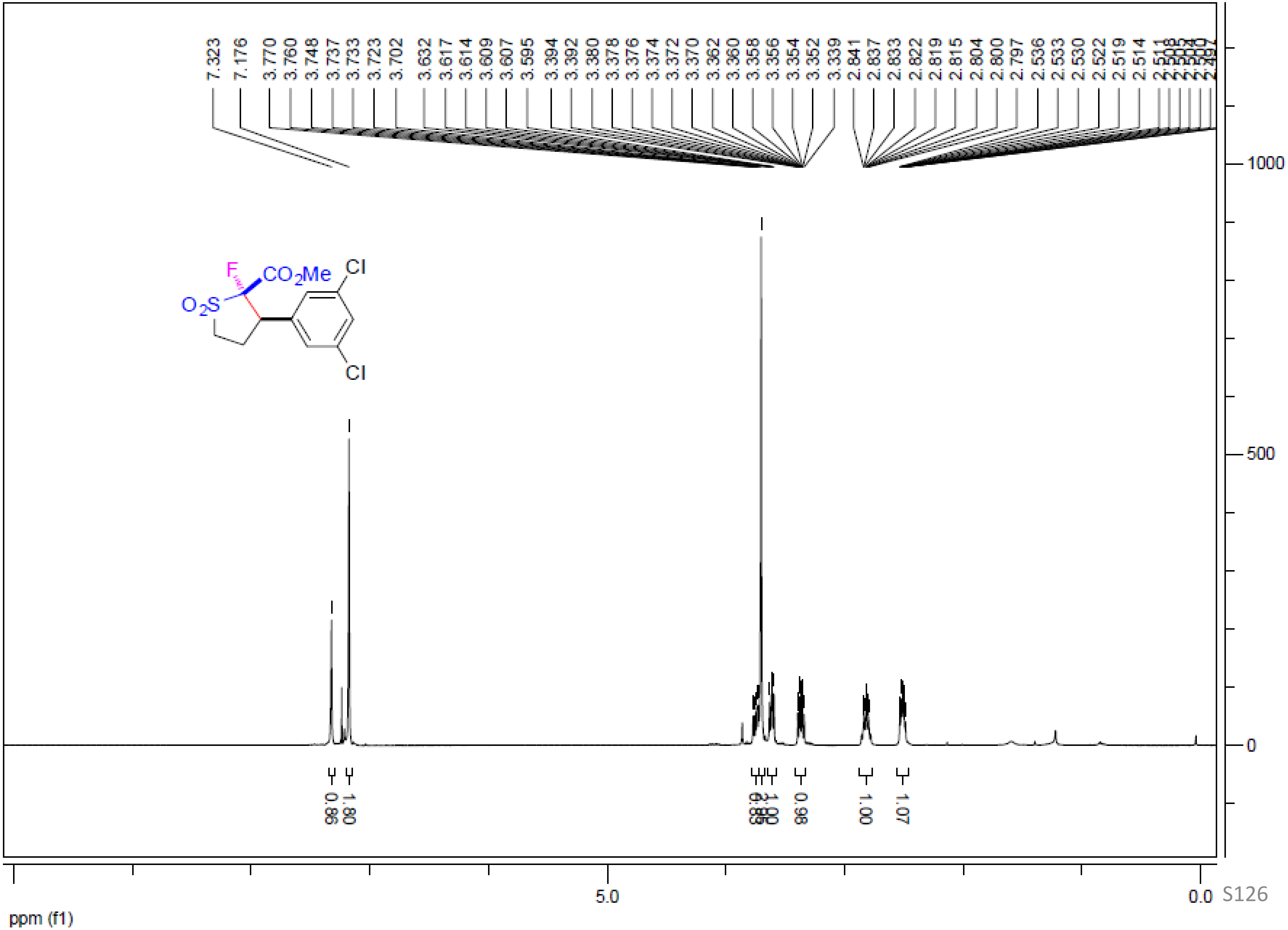

(2R,3R)-methyl 3-(3,5-dichlorophenyl)-2-fluorotetrahydrothiophene-2-carboxylate 1,1-dioxide (**3ea**)

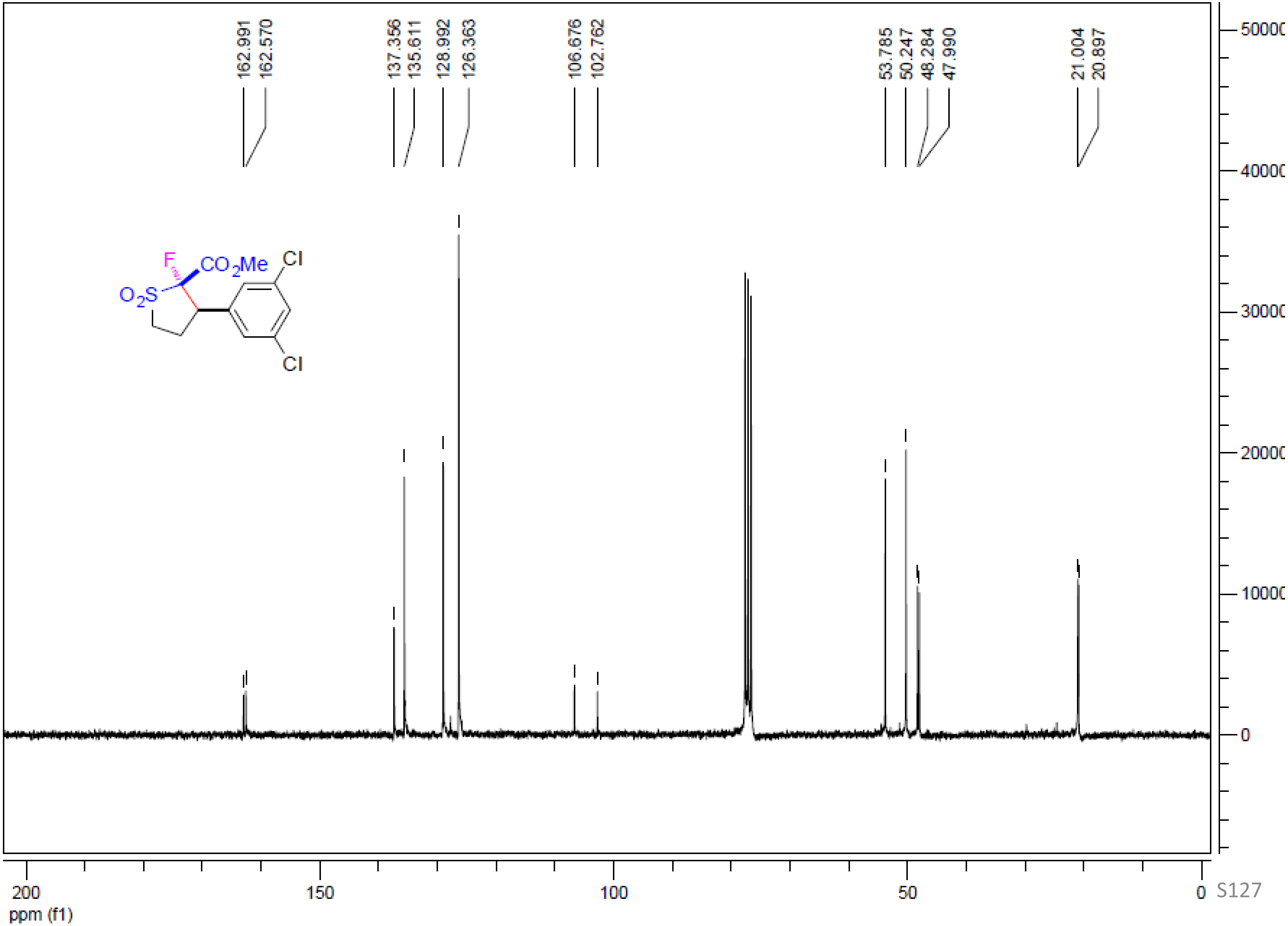

(2R,3R)-methyl 3-(3,5-dichlorophenyl)-2-fluorotetrahydrothiophene-2-carboxylate 1,1-dioxide (**3ea**)

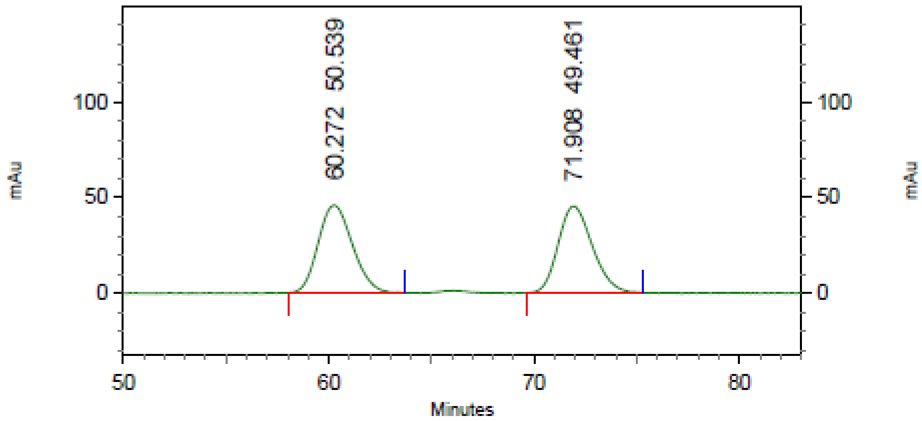

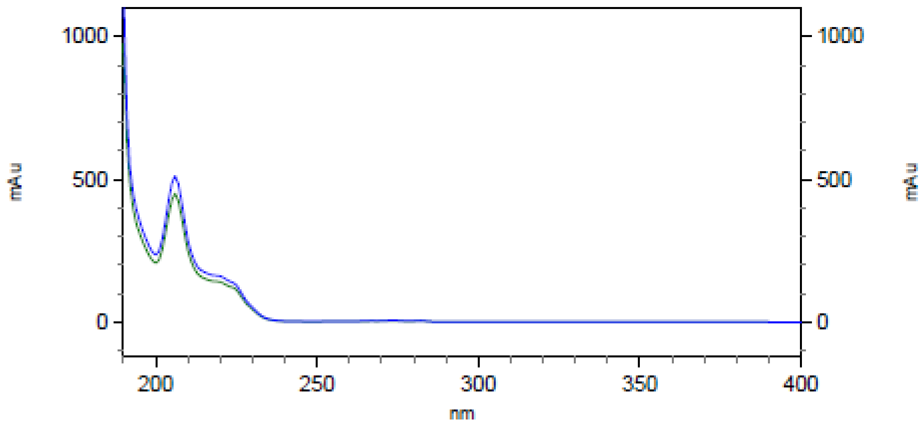

4: 223 nm, 4 nm
Results

Name	Retention Time	Area Percent	Pk #
	60.272	50.539	1
	71.908	49.461	2
Totals		100.000	

(2R,3R)-methyl 3-(3,5-dichlorophenyl)-2-fluorotetrahydrothiophene-2-carboxylate 1,1-dioxide (**3ea**)

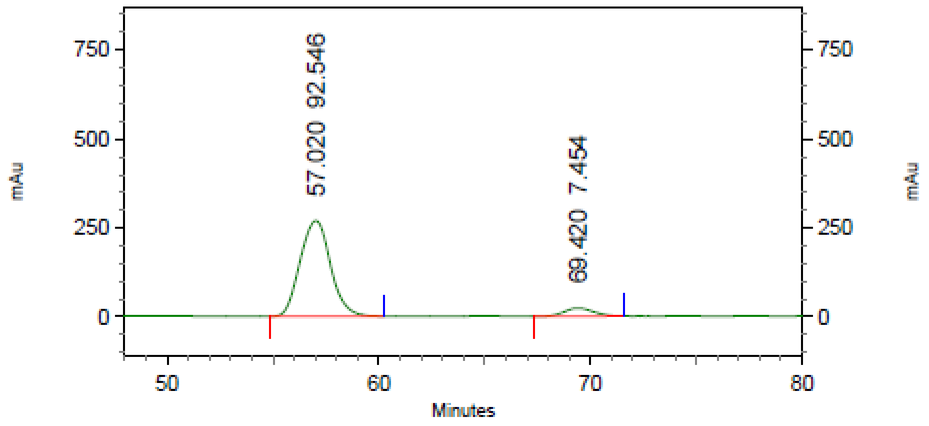

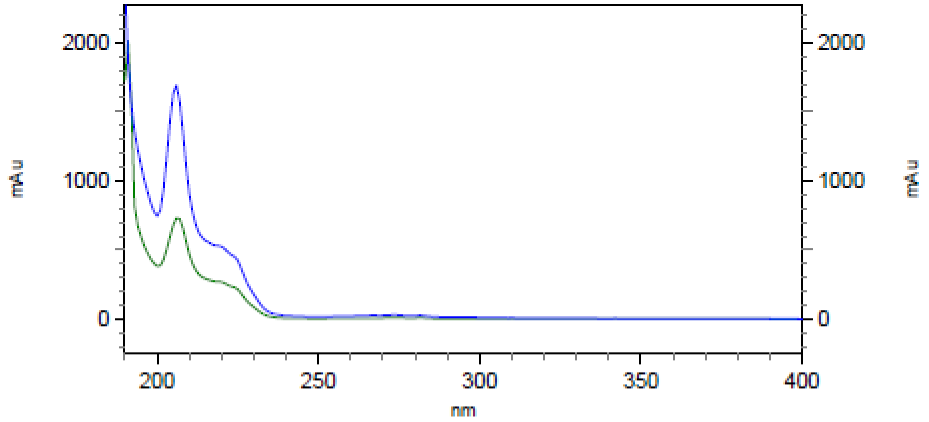

4: 223 nm, 4 nm
Results

Name	Retention Time	Area Percent	Pk #
	57.020	92.546	1
	69.420	7.454	2
Totals		100.000	

methyl 2-chloro-3-(4-methoxyphenyl)tetrahydrothiophene-2-carboxylate 1,1-dioxide (**3ib**)

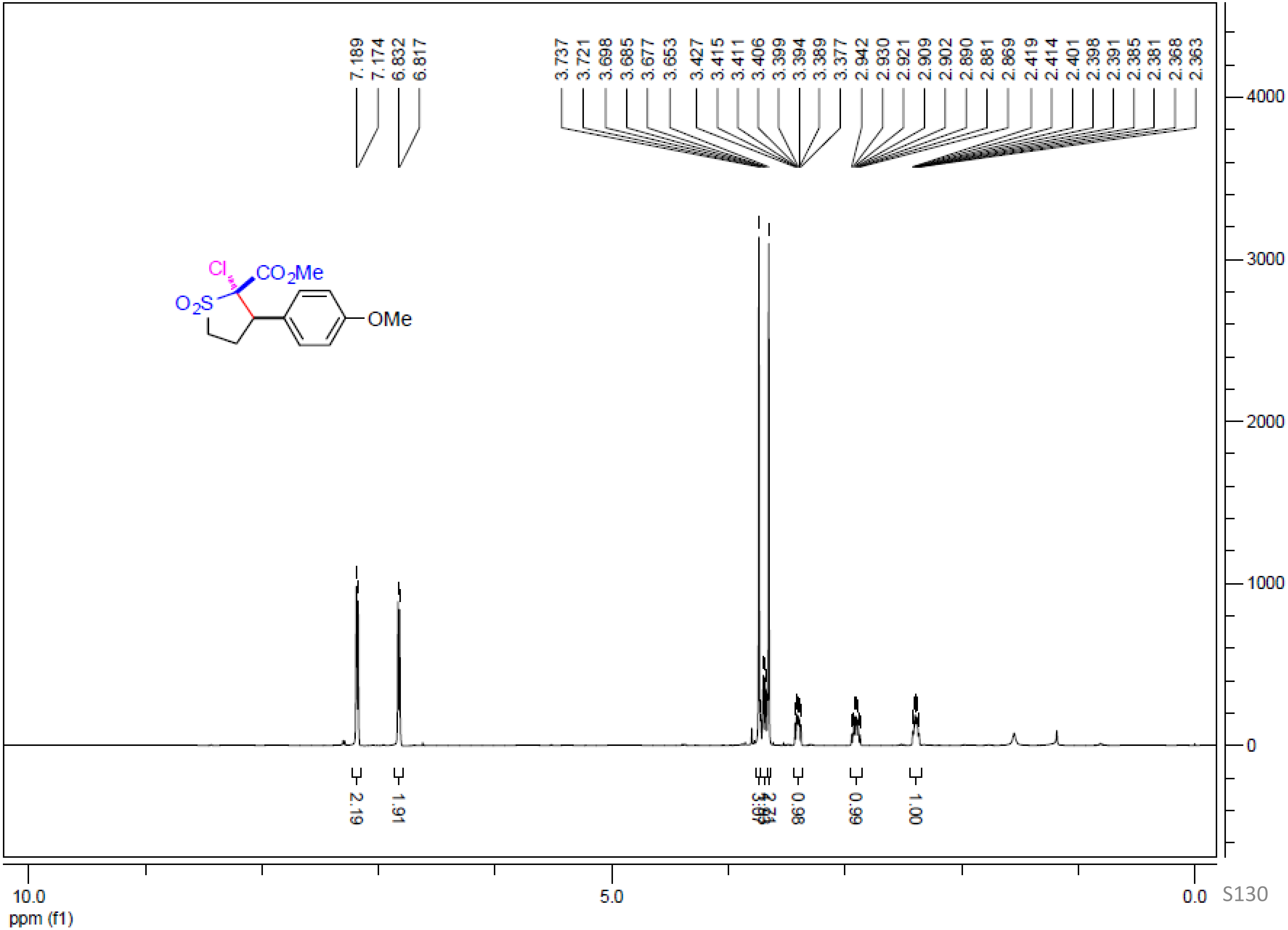

methyl 2-chloro-3-(4-methoxyphenyl)tetrahydrothiophene-2-carboxylate 1,1-dioxide

(3ib)

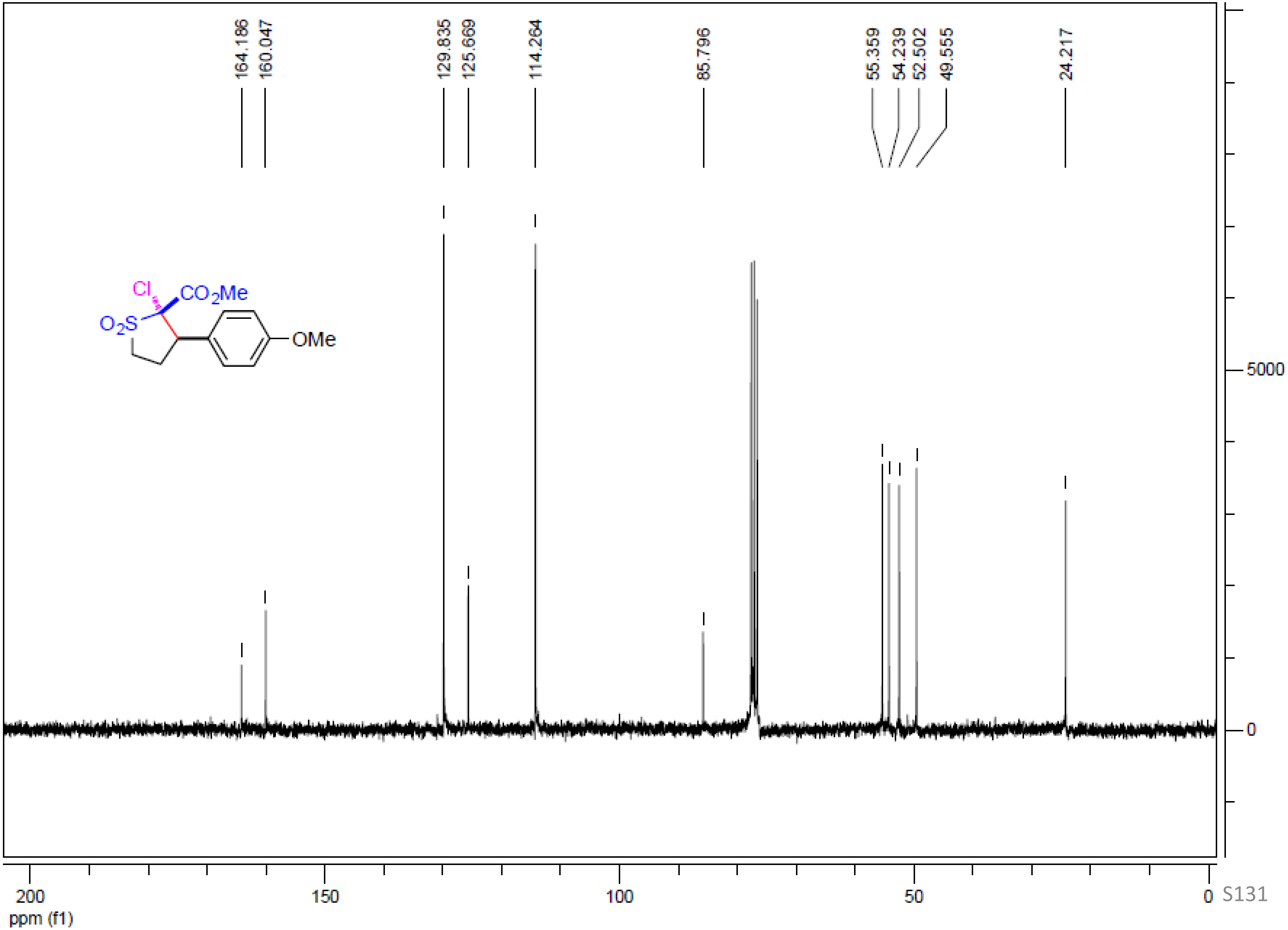

methyl 2-chloro-3-(4-methoxyphenyl)tetrahydrothiophene-2-carboxylate 1,1-dioxide (**3ib**)

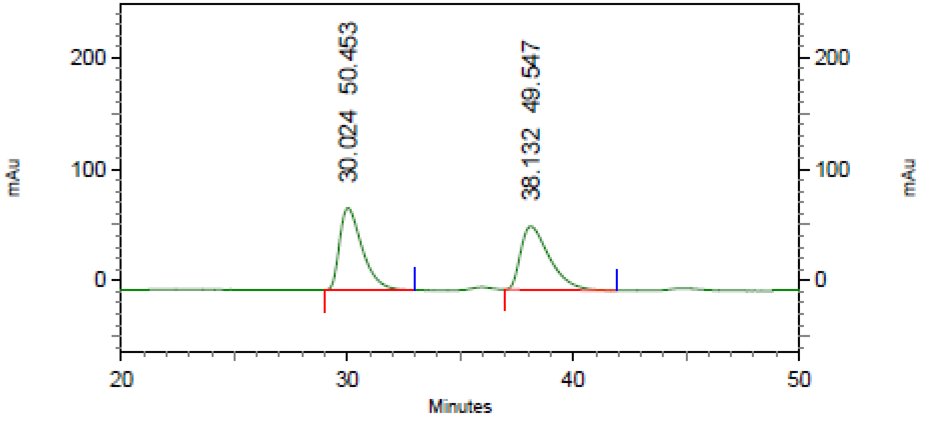

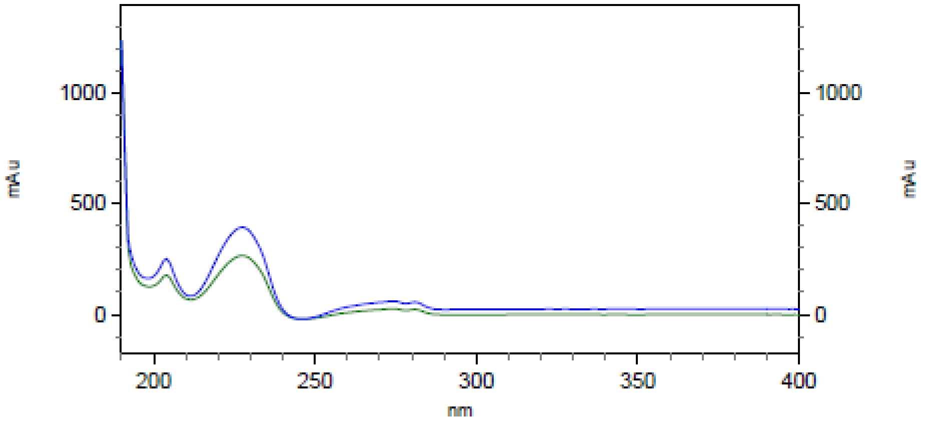

6: 232 nm, 4 nm
Results

Name	Retention Time	Area Percent	Pk #
	30.024	50.453	1
	38.132	49.547	2
Totals		100.000	

methyl 2-chloro-3-(4-methoxyphenyl)tetrahydrothiophene-2-carboxylate 1,1-dioxide (**3ib**)

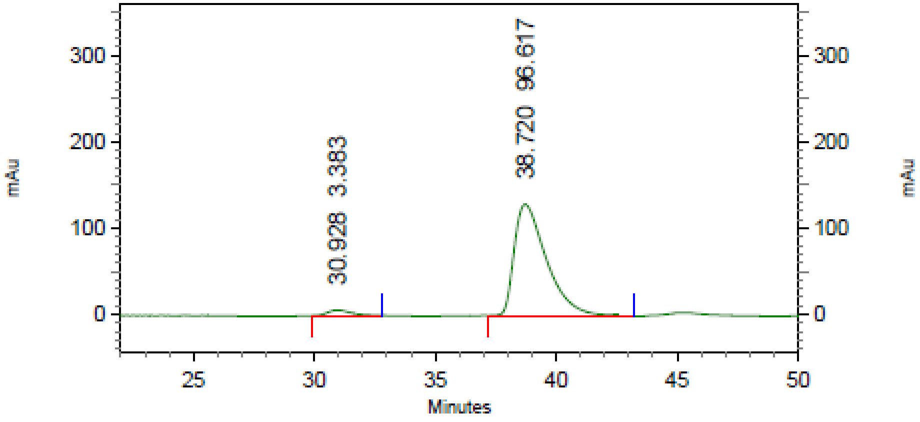

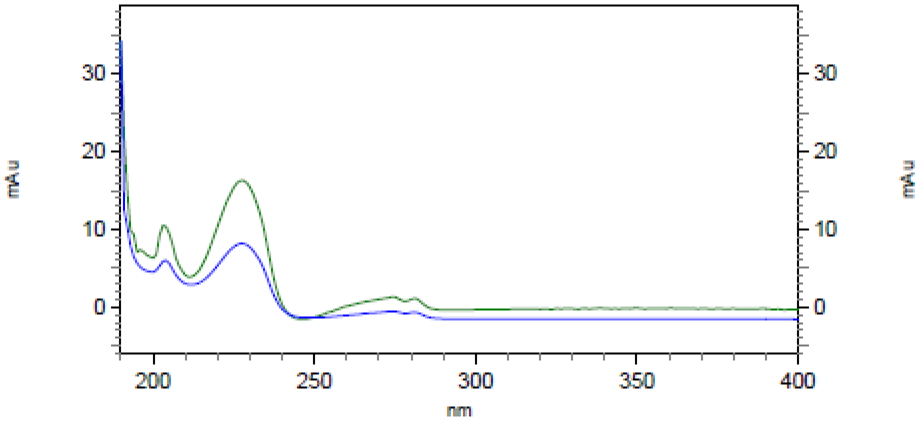

6: 232 nm, 4 nm

Results

Name	Retention Time	Area Percent	Pk #
	30.928	3.383	1
	38.720	96.617	2
Totals		100.000	

methyl 3-(4-methoxyphenyl)-2-methyltetrahydrothiophene-2-carboxylate 1,1-dioxide (**3ic**)

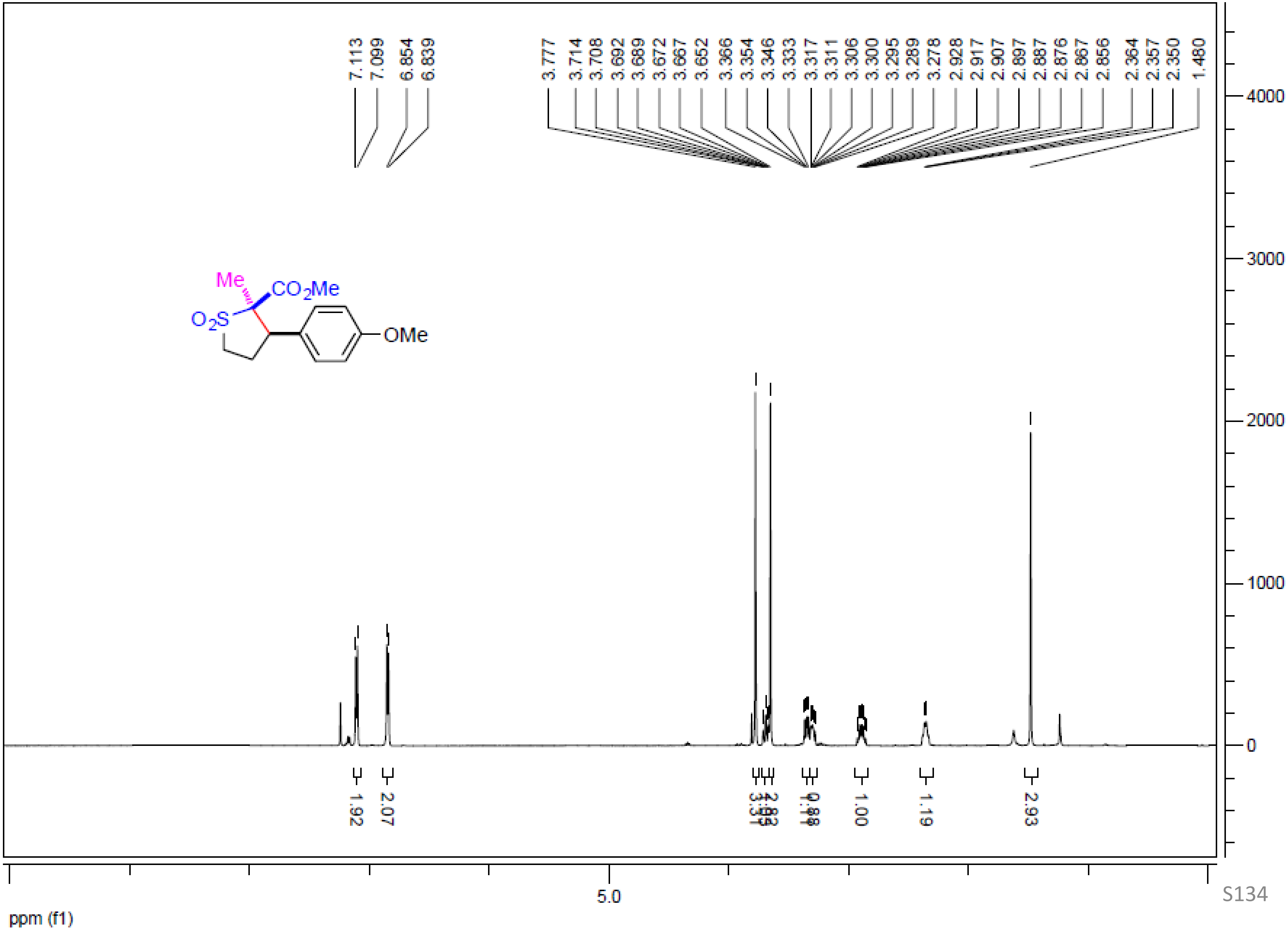

methyl 3-(4-methoxyphenyl)-2-methyltetrahydrothiophene-2-carboxylate 1,1-dioxide (**3ic**)

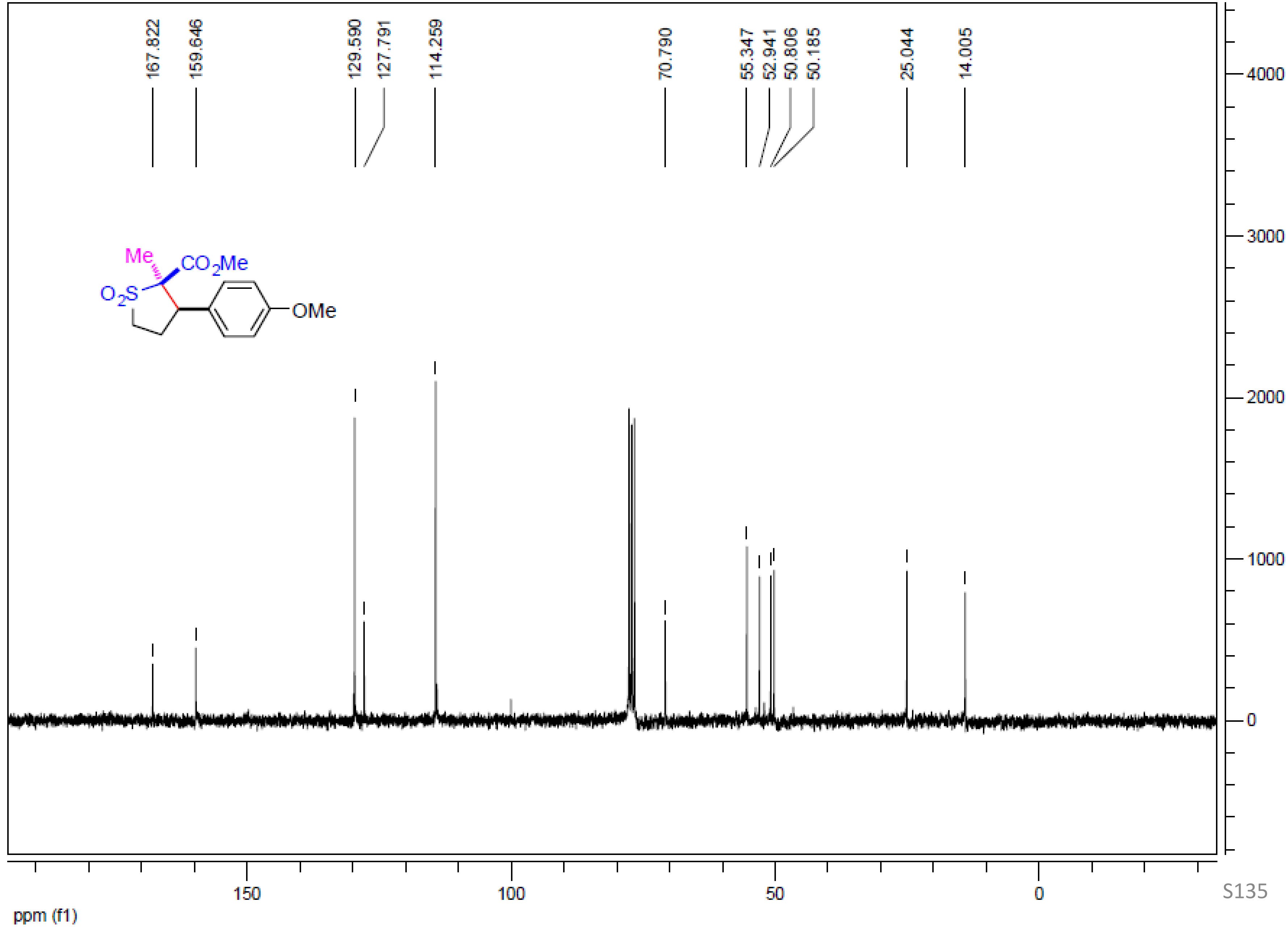

methyl 3-(4-methoxyphenyl)-2-methyltetrahydrothiophene-2-carboxylate 1,1-dioxide (**3ic**)

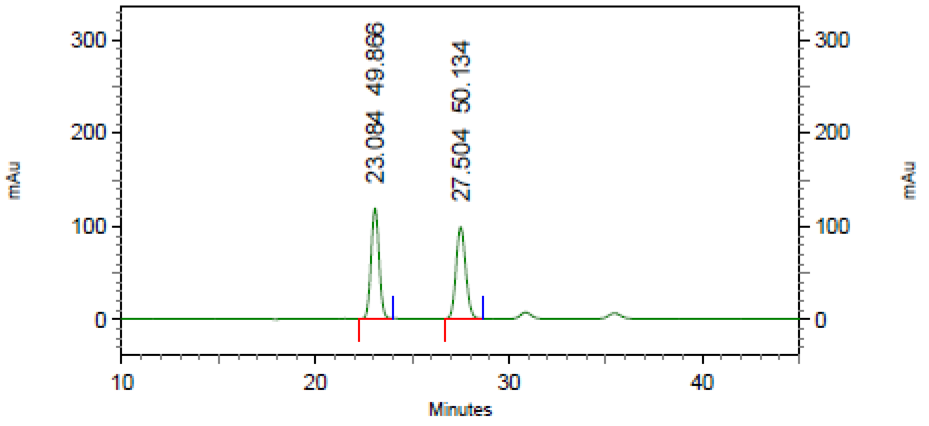

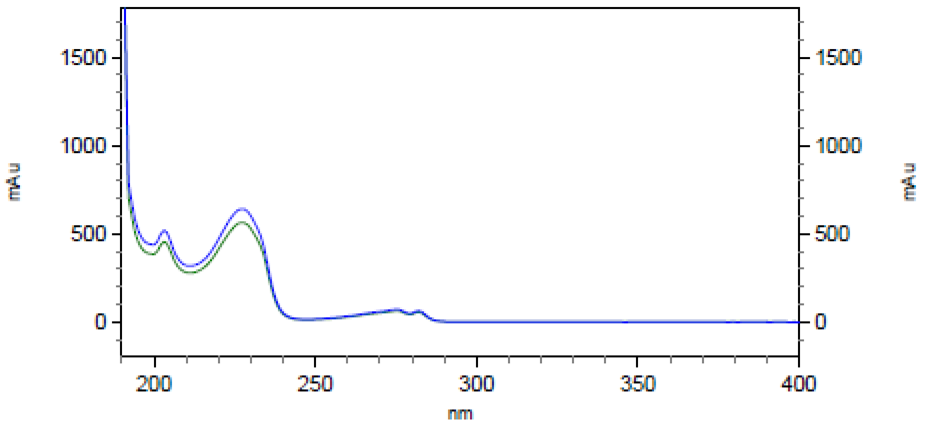

4: 229 nm, 4 nm

Results

Name	Retention Time	Area Percent	Pk #
	23.084	49.866	1
	27.504	50.134	2
Totals		100.000	

methyl 3-(4-methoxyphenyl)-2-methyltetrahydrothiophene-2-carboxylate 1,1-dioxide (**3ic**)

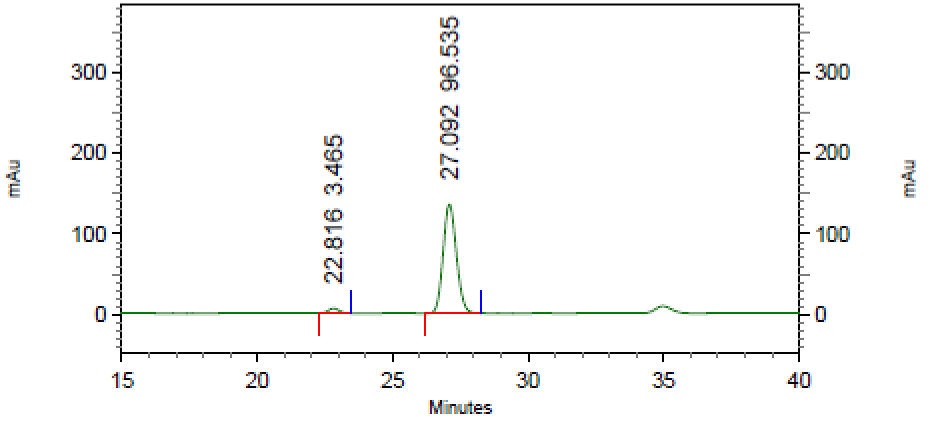

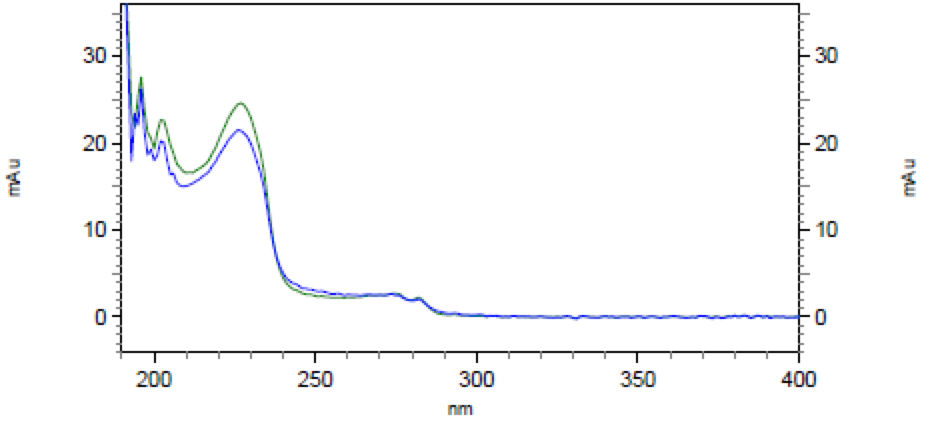

4: 229 nm, 4 nm

Results

Name	Retention Time	Area Percent	Pk #
	22.816	3.465	1
	27.092	96.535	2

Totals	
100.000	

methyl 2-(3-ethoxy-3-oxopropyl)-3-(4-methoxyphenyl)tetrahydrothiophene-2-carboxylate 1,1-dioxide (**3id**)

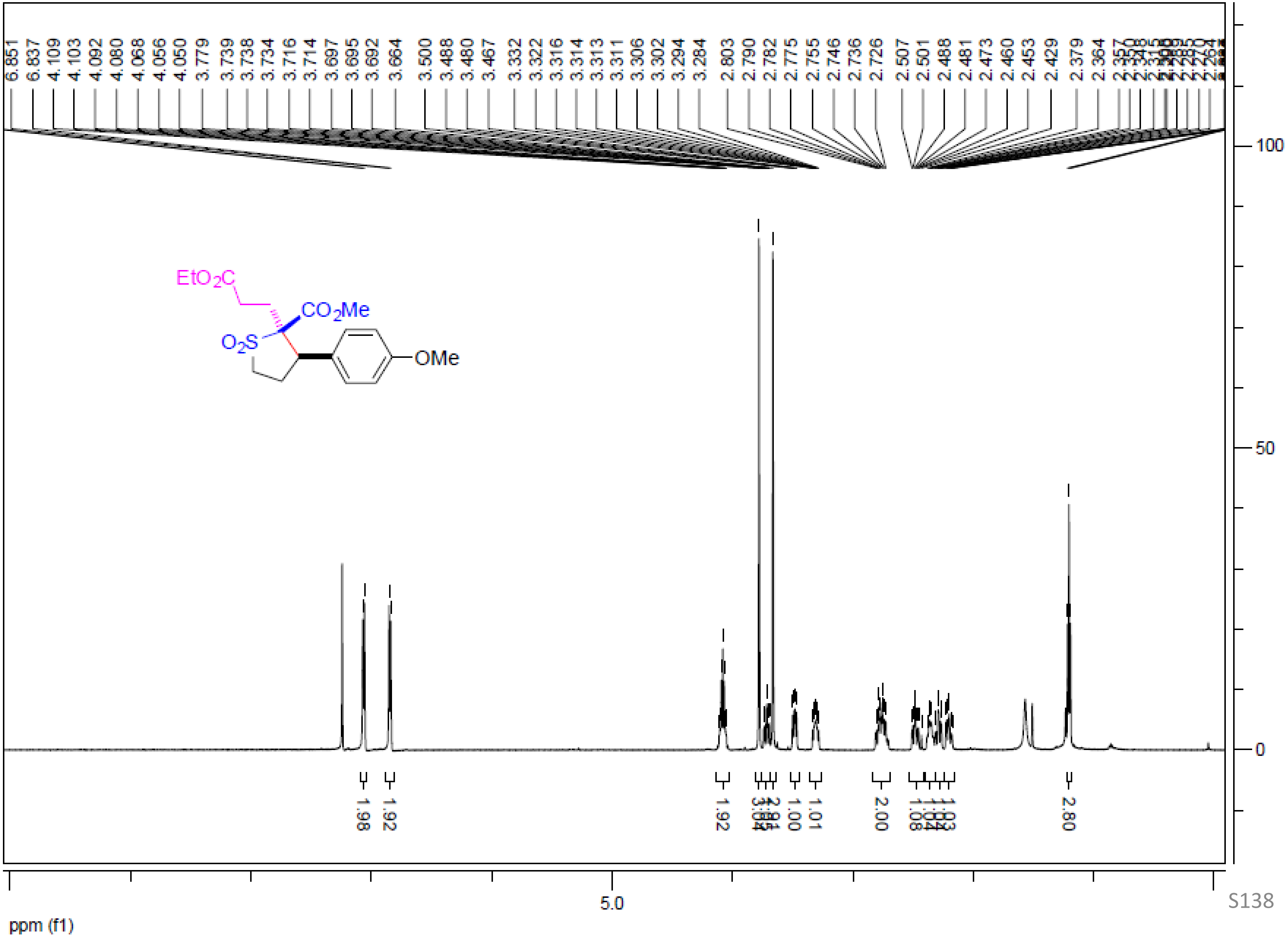

methyl 2-(3-ethoxy-3-oxopropyl)-3-(4-methoxyphenyl)tetrahydrothiophene-2-carboxylate 1,1-dioxide (**3id**)

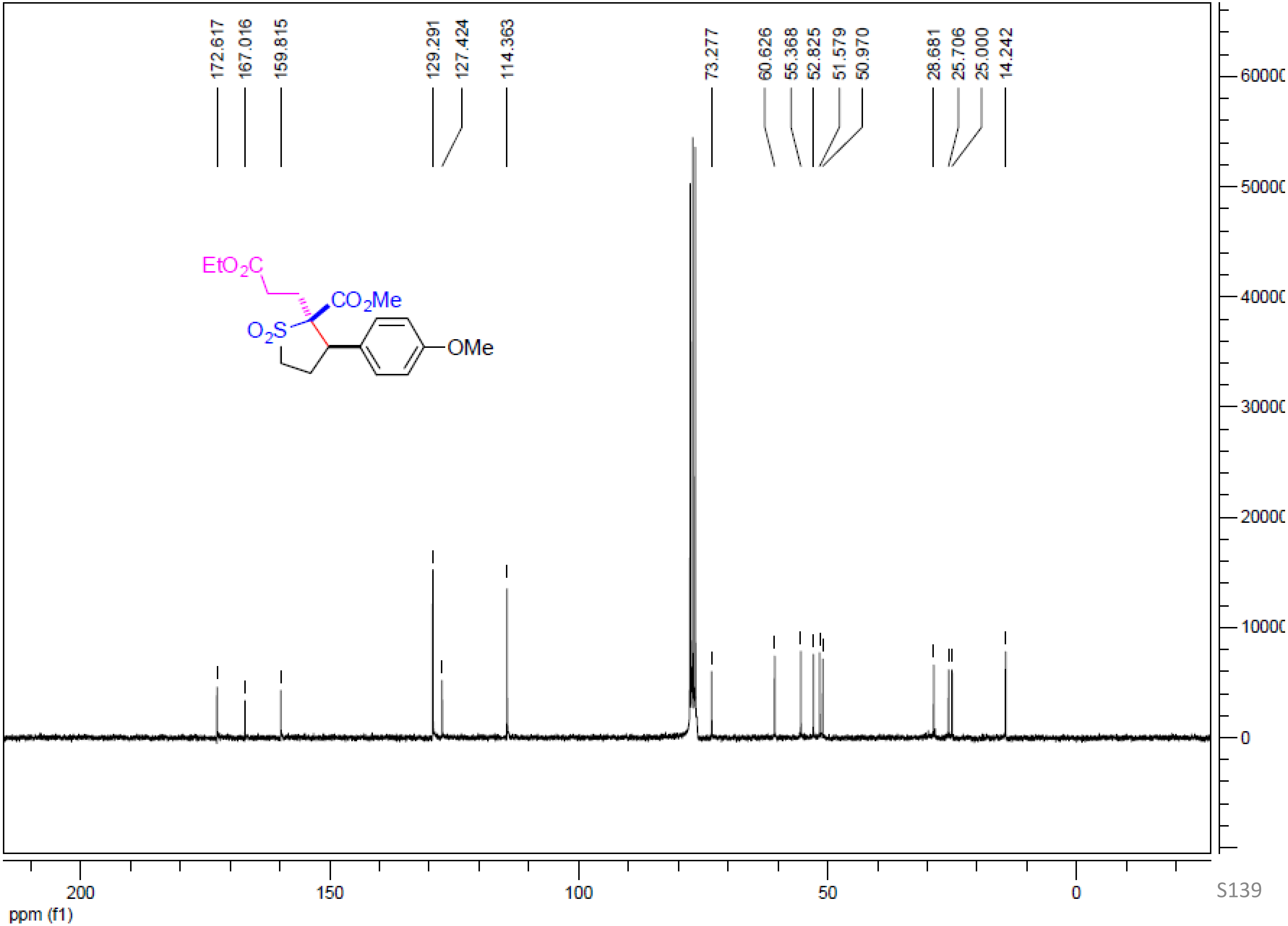

methyl 2-(3-ethoxy-3-oxopropyl)-3-(4-methoxyphenyl)tetrahydrothiophene-2-carboxylate 1,1-dioxide (**3id**)

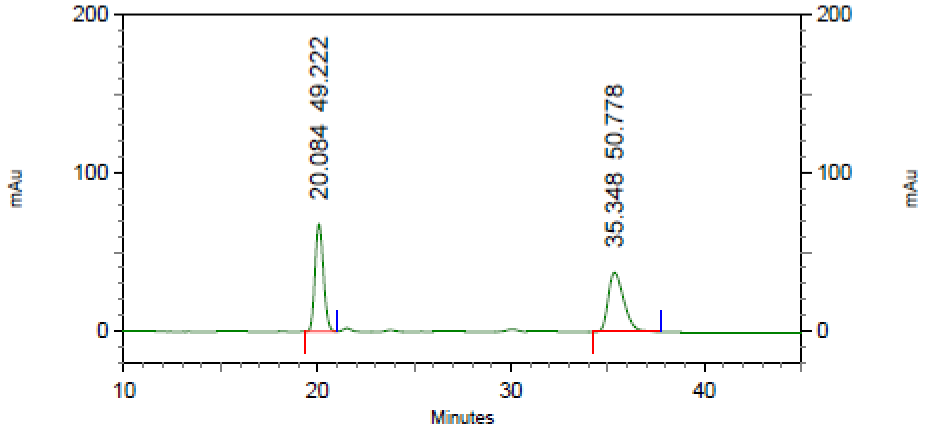

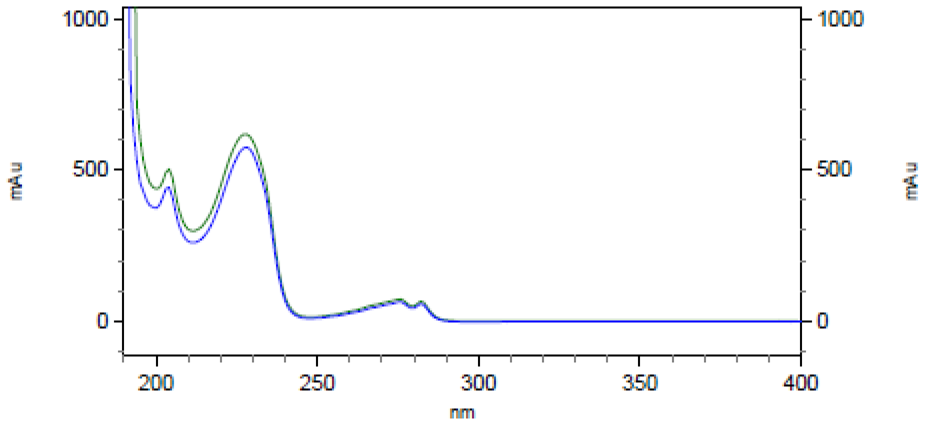

3: 237 nm, 4 nm
Results

Name	Retention Time	Area Percent	Pk #
	20.084	49.222	1
	35.348	50.778	2
Totals		100.000	

methyl 2-(3-ethoxy-3-oxopropyl)-3-(4-methoxyphenyl)tetrahydrothiophene-2-carboxylate 1,1-dioxide (**3id**)

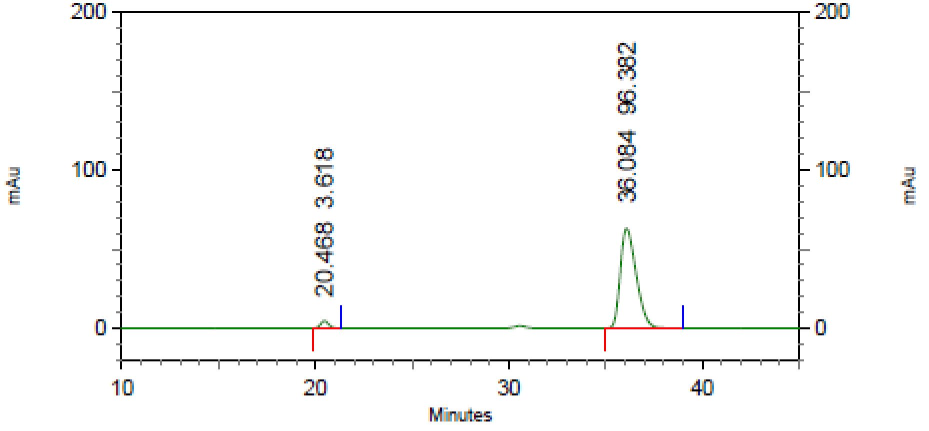

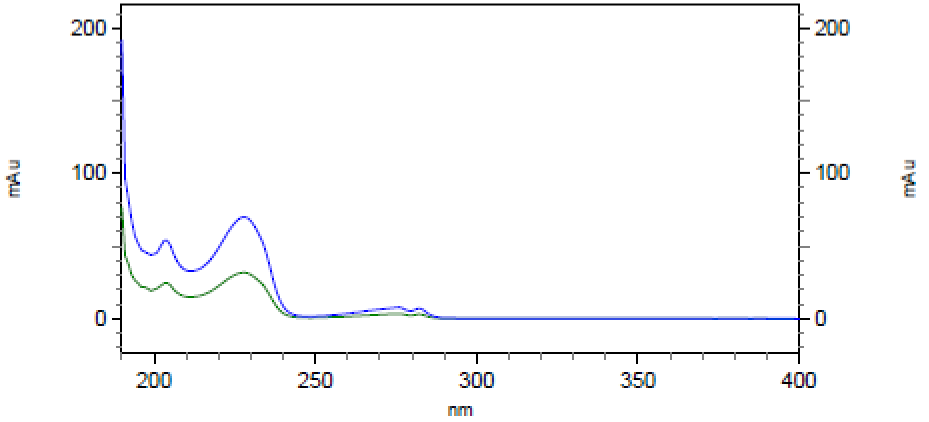

3: 229 nm, 4 nm

Results

Name	Retention Time	Area Percent	Pk #
	20.468	3.618	1
	36.084	96.382	2
Totals		100.000	
